# Hydrochemical composition and potentially toxic elements in the Kyrgyzstan portion of the transboundary Chu-Talas river basin, Central Asia

**DOI:** 10.1038/s41598-020-71880-4

**Published:** 2020-09-11

**Authors:** Long Ma, Yaoming Li, Jilili Abuduwaili, Salamat Abdyzhapar uulu, Wen Liu

**Affiliations:** 1 State Key Laboratory of Desert and Oasis Ecology, Xinjiang Institute of Ecology and Geography, Chinese Academy of Sciences, Urumqi, 830011 China; 2 Research Center for Ecology and Environment of Central Asia, Chinese Academy of Sciences, Urumqi, 830011 China; 3 University of Chinese Academy of Sciences, Beijing, 100049 China; 4 Institute of Geology, National Academy of Sciences of Kyrgyzstan, 720461 Bishkek, Kyrgyzstan

**Keywords:** Environmental monitoring, Environmental impact

## Abstract

Water chemistry and the assessment of health risks of potentially toxic elements have important research significance for water resource utilization and human health. However, not enough attention has been paid to the study of surface water environments in many parts of Central Asia. Sixty water samples were collected from the transboundary river basin of Chu-Talas during periods of high and low river flow, and the hydrochemical composition, including major ions and potentially toxic elements (Zn, Pb, Cu, Cr, and As), was used to determine the status of irrigation suitability and risks to human health. The results suggest that major ions in river water throughout the entire basin are mainly affected by water–rock interactions, resulting in the dissolution and weathering of carbonate and silicate rocks. The concentrations of major ions change to some extent with different hydrological periods; however, the hydrochemical type of calcium carbonate remains unchanged. Based on the water-quality assessment, river water in the basin is classified as excellent/good for irrigation. The relationship between potentially toxic elements (Zn, Pb, Cu, Cr, and As) and major ions is basically the same between periods of high and low river flow. There are significant differences between the sources of potentially toxic elements (Zn, Pb, Cu, and As) and major ions; however, Cr may share the same rock source as major ions. The risk assessment revealed low non-carcinogenic and carcinogenic risks for human health; however, the maximum carcinogenic risk for As exceeded the allowable value, which requires further consideration. These results provide a scientific basis for the management of agricultural irrigation uses and also infill existing gaps regarding the hydrochemical composition in the Chu-Talas river basin, Central Asia.

## Introduction

Water is important for the movement and transformation of material in nature, and water resources are important for the sustenance of life and social and economic development. Central Asia, located in the hinterland of Eurasia, has a semi-arid and arid continental climate, with scarce precipitation and extensive evaporation^1^. The shortage of water resources has seriously restricted the development of Central Asia^2,3^. To date, most studies on water resources in Central Asia have focused on the quantity of water under the influence of recent climatic and anthropogenic factors^4–7^. In particular, in view of the Aral Sea disaster^8^, systematic studies have been carried out on the quantity and management of water in the Syr Darya and Amu Darya rivers and reasons for changing water resources^9–12^. However, with the over-development of water resources and other human activities, a shortage in water resources has resulted, and at the same time, water resources in Central Asia have become seriously polluted^13^. Increased concentrations of toxic elements have been found in humans in the Aral Sea basin^14^. Nuclear tailing dumps^15^ and gold mining^16^ had important impacts on the water environment of Kyrgyzstan. Quality has a direct impact on the safe use of water for industrial, agricultural and drinking purposes. Among research studies examining water resources, relatively few are concerned with water quality. At present, most such studies are focused on water quality in the Syr Darya River of the Aral Sea Basin, especially with respect to radioactive pollution^17,18^. However, not enough attention has been paid to the study of surface water environments in other parts of Central Asia.

The Chu and Talas rivers originate in the Tianshan Mountains and dissipate in the Muyunkum Desert. Precipitation mainly occurs from October to April^19^. The main source of flowing water is from the melting of snowfields and glaciers^19^. In the Talas River and Chu River sub-basins, bedrocks are mainly composed of acid plutonic rocks, siliciclastic sedimentary rocks, carbonate sedimentary rocks, mixed sedimentary rocks, and Quaternary unconsolidated sediments^20^. Based on information from the Harmonized World Soil Database (v 1.2)^21^, soils in the Talas River sub-basin are composed of leptosol and Calcisols, and soils in the study area of the Chu River are composed of leptosol, Calcisols, and Kastanozem^21^. The sub-basin of the Chu River covers an area of 71,600 km^2^, and the river has a maximum length of 1,067 km ^22^. The sub-basin of the Talas River covers an area of 52,700 km^2^, and the river has a maximum length of 661 km ^22^. The importance of studying the Chu and Talas rivers lies in the fact that these two rivers are international rivers flowing through the territories of Kyrgyzstan and Kazakhstan and changes of water quality and quantity in the upstream countries have important impacts on the lower reaches. Transboundary water conflicts in Central Asia have become a very serious problem in the world^23–25^. In addition, the water resources of the Chu River and Talas River are mainly used for agricultural irrigation^22^, and changes in water quality have a significant impact on the safe use of water in agriculture. In addition, the pollution of potentially toxic elements in water is a hot issue in current researches^26,27^. The increased content of Cu and As can lead to certain inflammatory diseases and cardiac dysfunction^28^, and As has significant carcinogenicity^29^. Moderate or high zinc intake may be associated with the risk of prostate cancer^30^. Chronic toxicity of Pb is manifested as liver toxicity, nephrotoxicity and neurotoxicity, which can cause growth delay, neuronal defects and anemia in children^31,32^. Cr has been shown to be related to certain diseases such as nasal septum defect, liver and kidney damage, dermatitis, internal haemorrhage, and respiratory^33,34^.

Based on the above considerations, 60 water samples were collected from the transboundary Chu-Talas river basin during low river flow (n = 30) in May and high river flow (n = 30) in July and August, and the river waters were analyzed for hydrochemical composition and potentially toxic elements (Pb, Zn, Cu, Cr, and As). The study of water quality in this region can provide a scientific basis for the management of water for agricultural irrigation uses, as well as infill existing gaps regarding the hydrochemical composition of Chu and Talas river water. In addition, an important supplement to the study is the role of human activities in reforming the surface water chemical composition of Central Asia.

## Results

During the period of low river flow, the pH value ranged from 7.33 to 8.84, with an average value of 8.24. Conductivity ranged between 65.0 and 521.90 μS cm^−1^, with an average value of 213.22 μS cm^−1^. TDS varied between 80.21 and 474.79 mg L^−1^, with an average value of 229.74 mg L^−1^ (Table 1).

**Table 1:** Statistical summary of environmental indicators of river water quality in the Chu-Talas Basin during periods of low river flow (L, n = 30) and high river flow (H, n = 30).

Indicators	Period	Minimum	Maximum	Mean	Median	SD	SE
Ca (mg L^−1^)	L	10.27	63.75	29.41	24.16	12.93	2.36
	H	9.66	59.65	26.66	22.36	12.33	2.25
Mg (mg L^−1^)	L	1.10	22.42	7.05	5.15	5.28	0.96
	H	0.75	24.64	6.30	4.23	5.15	0.94
K (mg L^−1^)	L	0.62	2.24	1.34	1.23	0.46	0.08
	H	0.54	2.35	1.15	1.06	0.45	0.08
Na (mg L^−1^)	L	0.40	15.43	4.25	2.78	3.98	0.73
	H	0.37	15.46	3.27	1.66	3.90	0.71
HCO_3_ (mg L^−1^)	L	43.97	311.40	156.31	140.19	66.75	12.19
	H	56.75	284.28	143.58	128.09	65.27	11.92
SO_4_ (mg L^−1^)	L	4.77	79.38	24.95	22.22	17.10	3.12
	H	4.02	41.80	19.81	16.90	11.49	2.10
Cl (mg L^−1^)	L	0.48	12.77	2.25	1.37	2.61	0.48
	H	0.25	12.61	1.80	0.75	2.73	0.50
NO_3_ (mg L^−1^)	L	2.19	6.92	3.73	3.57	1.22	0.22
	H	1.17	6.12	2.84	2.68	1.10	0.20
pH	L	7.33	8.84	8.24	8.47	0.55	0.10
	H	7.14	8.99	8.58	8.73	0.42	0.08
EC (μS cm^−1^)	L	65.00	521.90	213.22	193.30	102.23	18.66
	H	36.00	542.00	195.93	145.15	132.92	24.27
TDS (mg L^−1^)	L	80.21	474.79	229.74	194.72	100.59	18.37
	H	76.11	414.84	205.76	171.61	94.10	17.18
Pb (μg L^−1^)	L	0.046	2.976	0.929	0.710	0.782	0.143
	H	0.054	4.655	1.096	0.665	1.175	0.215
Zn (μg L^−1^)	L	1.104	9.855	4.972	5.097	2.250	0.411
	H	0.950	22.664	5.047	3.106	5.150	0.940
Cu (μg L^−1^)	L	0.376	9.570	2.298	1.792	2.217	0.405
	H	0.154	11.853	2.210	1.023	3.113	0.568
Cr (mg L^−1^)	L	0.020	0.060	0.034	0.030	0.009	0.002
	H	0.020	0.080	0.037	0.040	0.012	0.002
As (μg L^−1^)	L	0.323	3.417	1.335	0.878	1.020	0.186
	H	0.271	3.316	1.258	0.904	0.996	0.182

During the period of low river flow, the Ca^2+^ varied from 10.27 to 63.75 mg L^−1^ with a mean value of 29.41 mg L^−1^, and the Mg^2+^ had a range of 1.10 to 22.42 mg L^−1^ with a mean value of 7.05 mg L^−1^. The minimum, mean, and maximum values for Na^+^ were 0.40, 4.25, and 15.43 mg L^−1^, respectively. The K^+^ values varied from 0.62 to 2.24 mg L^−1^, with an average value of 1.34 mg L^−1^. The HCO_3_^−^ varied from 43.97 to 311.40 mg L^−1^ with a mean value of 156.31 mg L^−1^, and SO_4_^2−^ had a range of 4.77 to 79.38 mg L^−1^ with a mean value of 24.95 mg L^−1^. The minimum, mean, and maximum values for Cl^−^ were 0.48, 12.77, and 2.25 mg L^−1^, respectively. The NO_3_^−^ values varied from 2.19 to 6.92 mg L^−1^, with an average value of 3.73 mg L^−1^.

With respect to potentially toxic elements during low river flow, the concentration of Zn ranged from 1.104 to 9.855 μg L^−1^, with an average value of 4.972 μg L^−1^; Pb ranged between 0.046 and 2.976 μg L^−1^, with an average of 0.929 μg L^−1^; and, Cu varied between 0.376 and 9.570 μg L^−1^, with an average value of 2.298 μg L^−1^; The minimum, mean, and maximum values for Cr were 0.20, 0.034, and 0.060 mg L^−1^, respectively. The As concentration varied from 0.323 to 3.417 μg L^−1^, with an average value of 1.335 μg L^−1^.

During the period of high river flow, the pH value ranged from 7.14 to 8.99, with an average value of 8.58. Conductivity values ranged between 36–542 μS cm^−1^, with an average value of 195.93 μS cm^−1^. TDS varied between 76.11 and 414.84 mg L^−1^, with an average value of 205.76 mg L^−1^.

For major ions, Ca^2+^ varied from 9.66 to 59.65 mg L^−1^ with a mean value of 26.66 mg L^−1^, and Mg^2+^ had a range of 0.75 to 24.64 mg L^−1^ with a mean value of 6.30 mg L^−1^. The minimum, mean, and maximum values for Na^+^ were 0.37, 3.27, and 15.46 mg L^−1^, respectively. The K^+^ values varied from 0.54 to 2.35 mg L^−1^, with an average value of 1.15 mg L^−1^. The HCO_3_^−^ concentration varied from 56.75 to 284.28 mg L^−1^ with a mean value of 143.58 mg L^−1^, and SO_4_^2−^ had a range of 4.02 to 41.80 mg L^−1^ with a mean value of 19.81 mg L^−1^. The minimum, mean, and maximum values for Cl^−^ were 0.25, 1.8, and 12.61 mg L^−1^, respectively. The NO_3_^−^ values varied from 1.17 to 6.12 mg L^−1^, with an average value of 2.84 mg L^−1^.

With respect to potentially toxic elements, the Zn concentration ranged from 0.95 to 22.664 μg L^−1^, with an average value of 5.047 μg L^−1^. The range in Pb concentrations was from 0.054 to 4.655 μg L^−1^, with an average of 1.096 μg L^−1^, while Cu varied between 0.154– 11.853 μg L^−1^ with an average value of 2.210 μg L^−1^. The minimum, mean, and maximum values for Cr were 0.020, 0.037 and 0.080 mg L^−1^, respectively. The As concentrations ranged from 0.271 to 3.316 μg L^−1^, with an average value of 1.258 μg L^−1^.

According to independent sample tests of different water chemistry parameters at the two periods (Table S1 in supplementary information, there were some significant differences for NO_3_^−^ (t = -2.958, *p* = 0.004 < 0.05) and Cd (t = − 2.377, *p* = 0.026 < 0.05) at the two periods. For other hydrochemical parameters for the same sampling point at different periods, there were no statistically significant differences.

## Discussion

### Factors influencing the chemical composition of river waters in the Chu- Talas Basin

**Figure 1:**
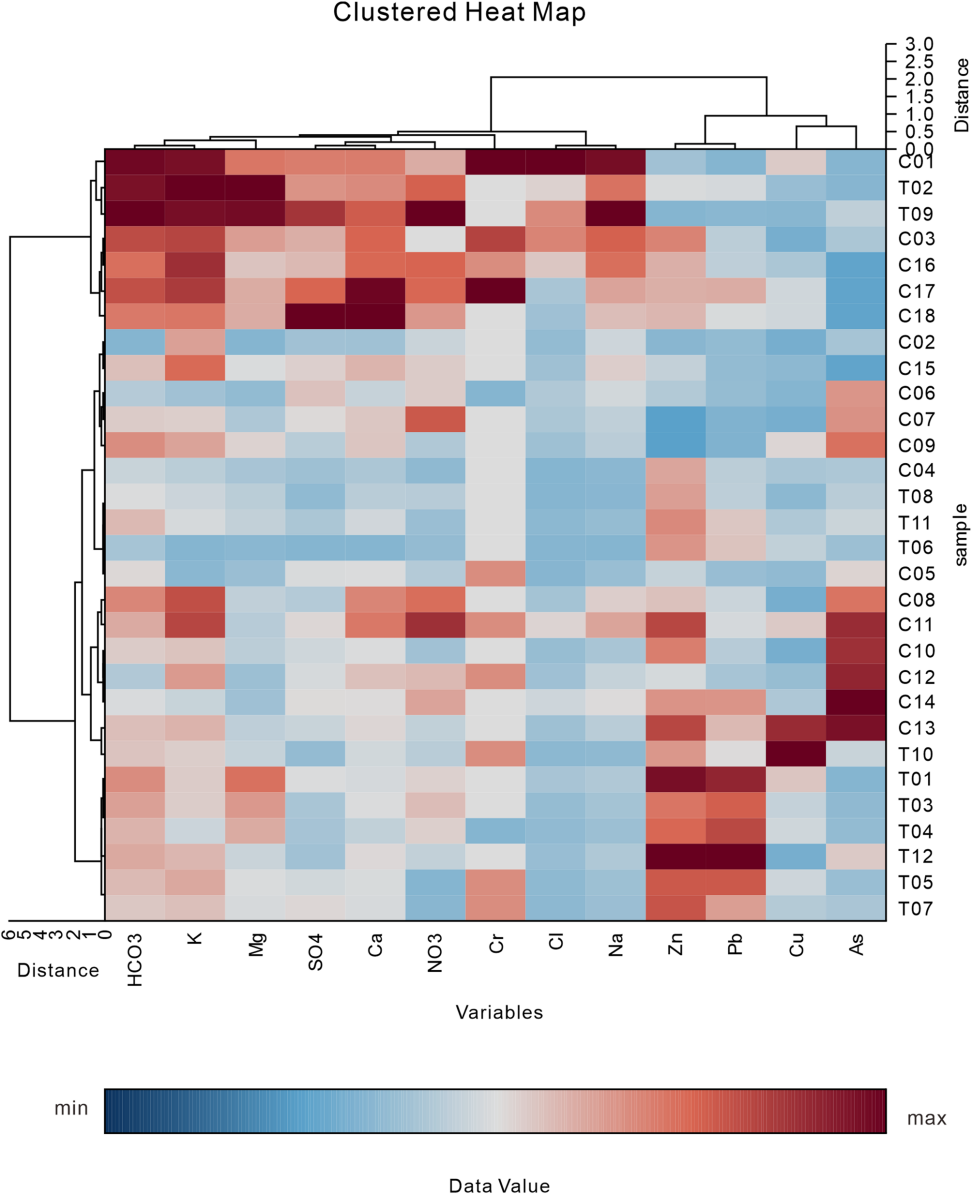
A heat map of the two-dimensional hierarchical cluster analysis suggesting differential clustering of the influencing factors on potentially toxic elements in river waters during the period of low river flow.

**Figure 2:**
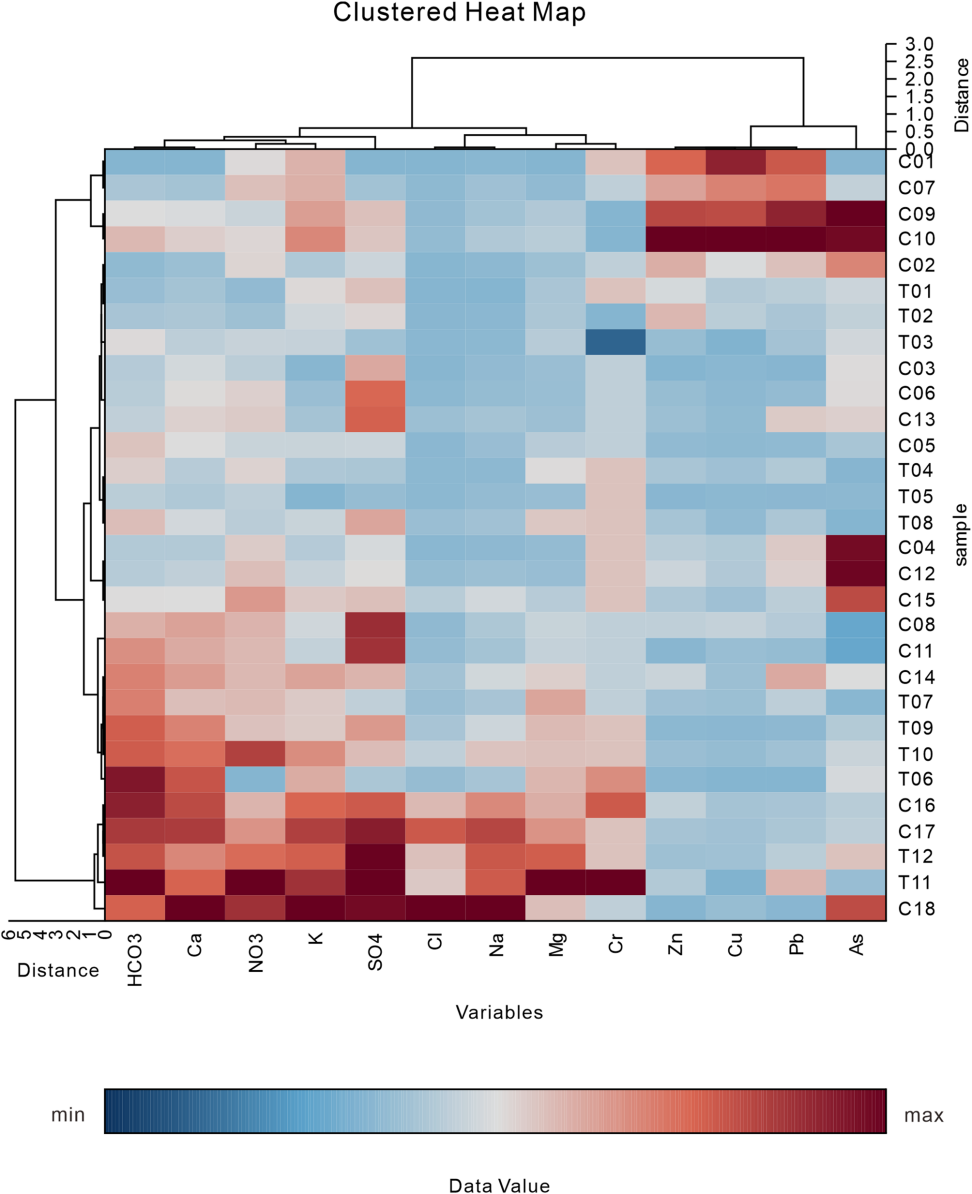
A heat map of the two-dimensional hierarchical cluster analysis suggesting differential clustering of the influencing factors on potentially toxic elements in river waters during the period of high river flow.

Cluster analysis^35–39^ was used to reveal the differences between potentially toxic elements and major ions, and differences among water samples from the Chu-Talas Basin. The method of Euclidean distance was used in the cluster analysis for the similarity measure, and the method of Ward’s minimum variance was used for the clustering of variables and row dendrograms. The results are represented as heat maps in Figs. 1 and 2 for low and high flow periods, respectively. The top dendrogram shows the similarity between major ions and potentially toxic elements, while the left dendrogram shows clustering of the river water samples (the samples are shown in Table S1).

As noted from the top dendrogram in both Figs. 1 and 2, Cr was strongly linked with major ions indicating a strong influence from water rock interactions. The relationship between potentially toxic elements and major ions was generally the same during periods of low and high river flow. However, an obvious linkage with Zn, Pb, Cu, and As was also observed during the low flow period. Furthermore, human activities may have influenced the potentially toxic elements Zn, Pb, Cu, and As. The left dendrogram provided detailed information about the water samples from which spatial differences were inferred. No spatial accumulation of sampling locations was observed during either the dry season or the rainy season. It can thus be concluded that the cluster analysis of major ions and potentially toxic elements indicates no significant differences between the two sub-basins; however, differences were observed in the relationship between the major ions reflecting differences between the different hydrological periods. Whether this significantly impacts the hydrochemistry is discussed through the following analysis.

**Figure 3:**
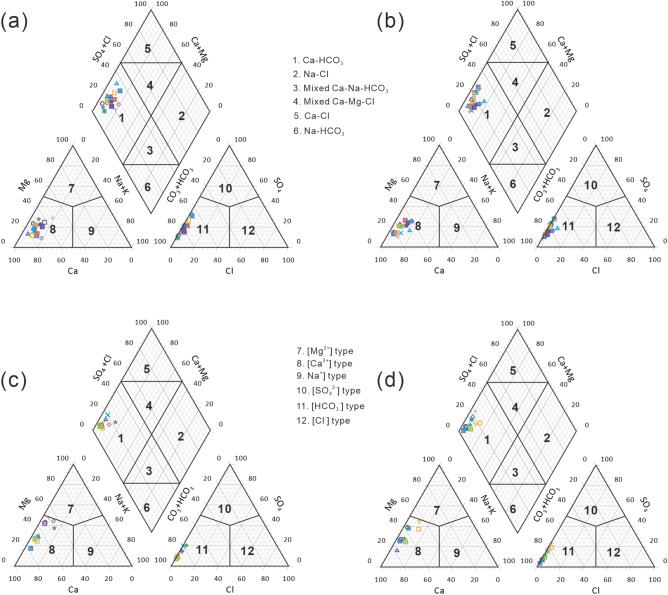
Piper diagrams for water samples from the Chu-Talas river basin. (**A**) Chu River system during low river flow; (**B**) Chu River system during high river flow; (**C**) Talas River system during low river flow; (**D**) Talas River system during high river flow.

**Figure 4:**
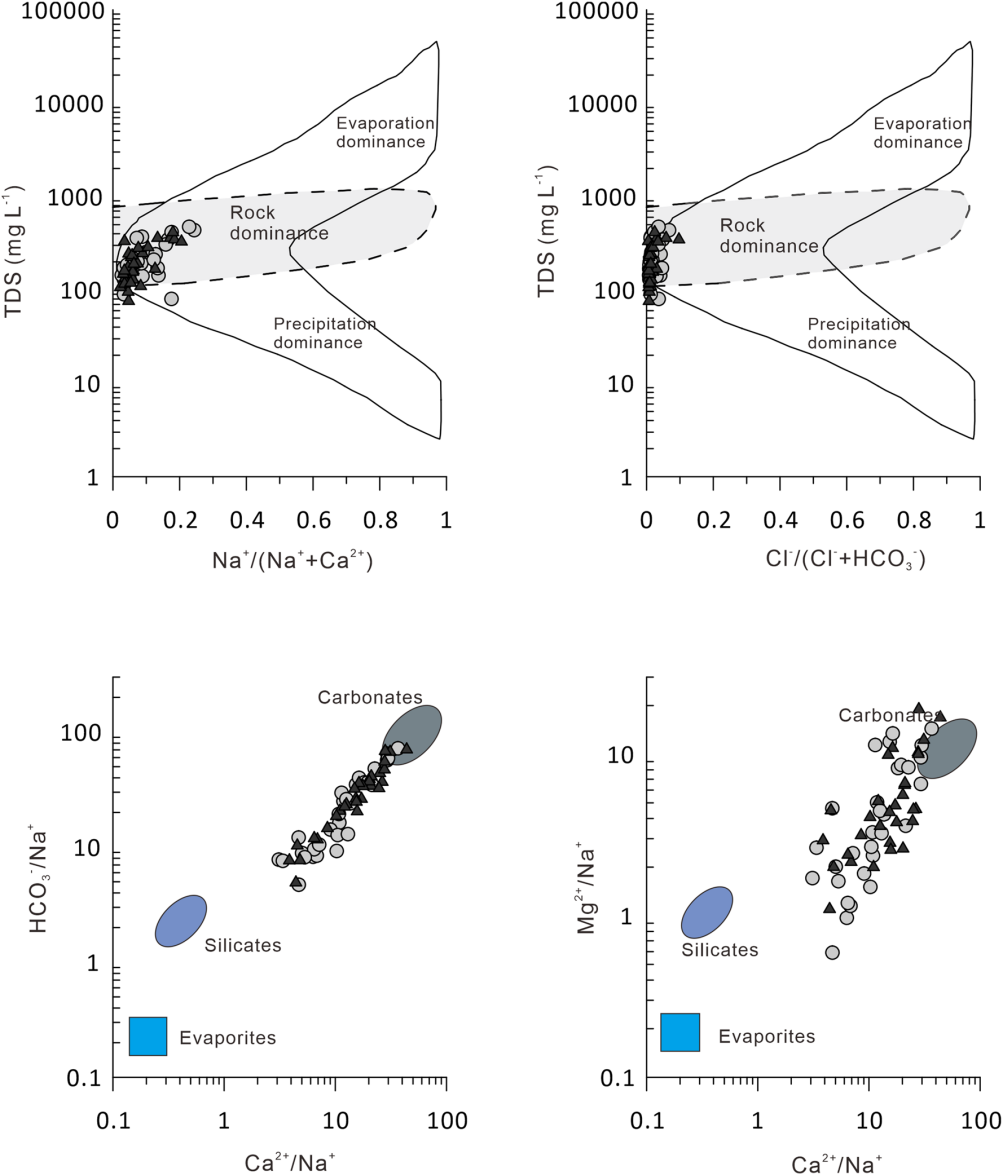
Gibbs (top) and mixing (bottom) diagrams for river water samples from the Chu-Talas river basin. The gray dots represent samples collected during low river flow and the black triangles represent samples collected during high river flow.

Piper diagrams are widely used to display dominant hydrochemical ions, and are useful for revealing the source of the hydrochemical composition^40–42^. All of the water samples were of the calcium bicarbonate type (Ca-HCO_3_) (Fig. 3), which suggests that there is no difference in the classification of water chemistry between water samples collected from the Chu-Talas river basin during low flow and high flow periods. However, the relative content of Mg^2+^ ions in the Talas River sub-basin was higher than that in the Chu River sub-basin, as seen from Fig. 3. On the Gibbs diagrams presented in Fig. 4, the river water samples mostly plotted within the area of rock dominance located between the two end-members of Carbonate and Silicate, which suggests that major ions have mainly been influenced by the process of water–rock interactions. This reflects the fact that the two rivers are adjacent to each other sharing the same geological background and source rocks under the same climatic conditions^43^. This leads to insignificant differences in major ions between the two sub-basins.

**Figure 5:**
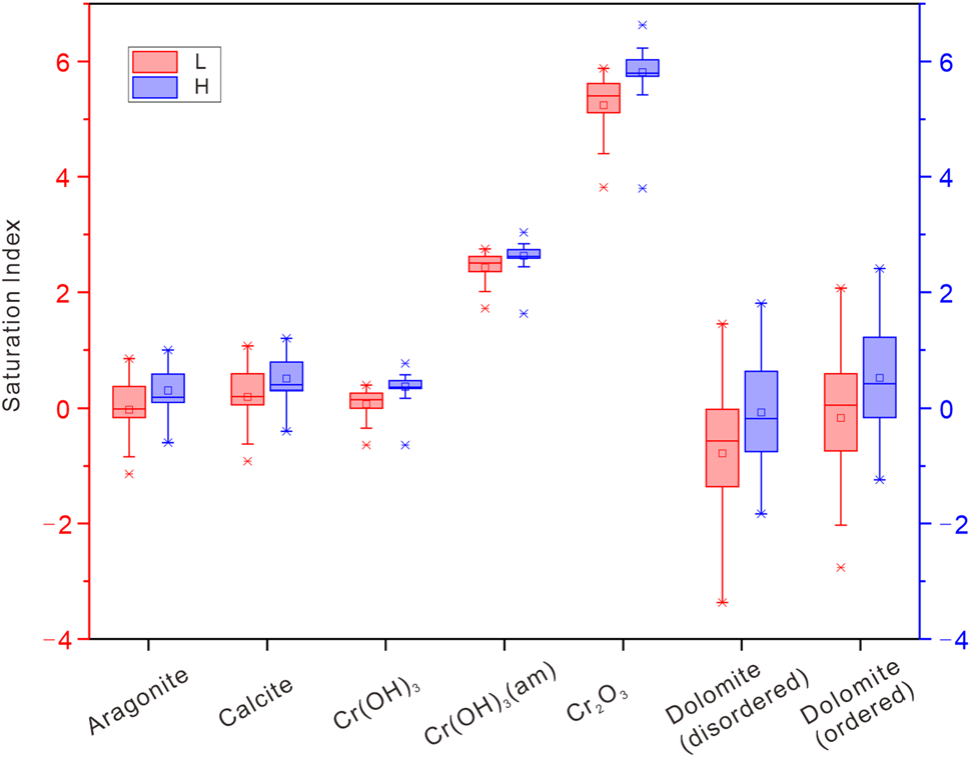
Saturation indices for the phases: Aragonite, Calcite, Cr(OH)_3_, Cr(OH)_3_(am), Cr_2_O_3_, Dolomite (disordered), and Dolomite (ordered) at different periods: low river flow (L) and high river flow (H).

Based on the simulation for distribution of species and calculation of Saturation indices (see the Supplementary Information for simulation results), the results are basically consistent with the above-mentioned discussion. Saturation indices for Aragonite, Calcite, Cr(OH)_3_, Dolomite (disordered), and Dolomite (ordered) fluctuated around 0 (Fig. 5). The phases Cr(OH)_3_(am) and Cr_2_O_3_ are in a supersaturated state. The Saturation indices of the remaining mineral phases are far less than zero. The bedrock of the Lake watershed contains a lot of carbonates and evaporative salts, and the saturation index of surface water bodies of Aragonite and Calcite is nearly saturated^44^. The Saturation indices in the low-flow period are smaller than that in the high-flow period, reflecting the enhancement of watershed weathering and water–rock interaction during the latter period.

### Human health risk assessment and water irrigation evaluation

**Figure 6:**
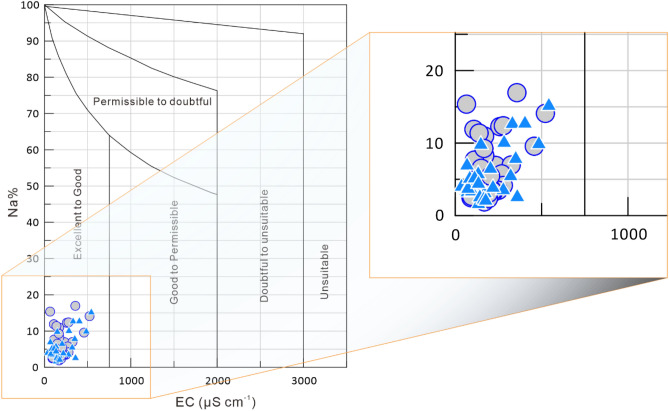
Wilcox diagram (%Na vs. EC) for river water samples. The dots represent samples collected during low river flow and the triangles represent samples collected during high river flow.

Wilcox diagrams are widely used to assess water suitable for irrigation^45–49^. In the Wilcox diagram shown in Fig. 6, the sodium percentage (%Na) versus EC was plotted to assess water quality for irrigation, which shows that river water in the Chu-Talas Basin is excellent/good for irrigation use.

The evaluation of irrigation water noted above is based on the composition of major ions, but harmful effects on human health from potentially toxic elements in water are also of great concern. Research studies have shown that in developed countries and regions, potentially toxic elements have already had a significant impact on human health^50,51^. There is a particular gap in the study and evaluation of potentially toxic elements in the surface waters of Central Asia. Research on the arid regions of Central Asia is important for the future environmental protection and sustainable development of water resources. The daily average exposure doses from the ingestion and dermal absorption of potentially toxic elements in Chu-Talas river water were calculated, and the results of the human health risk assessment are presented in Table 2. With the calculation of the threshold value for the carcinogenic (CI = 10–4) and non-carcinogenic risks (HI = 1), the threshold values (non-carcinogenic) for Pb, Zn, Cu, Cr, and As were 124 μg L^−1^, 10.8 mg L^−1^, 1.44 mg L^−1^, 61.5 ug L^−1^, and 484 μg L^−1^, respectively. The carcinogenic threshold value for As was 2.41 μg L^−1^. And then, the margin of safety was derived from the difference between the threshold value for the carcinogenic and non-carcinogenic risks and the current value for potentially toxic element in the study area. For non-carcinogenic risks, except for Cr, the content of other elements is far below the risk threshold. However, during the low river flow stage, the Cr content of two sampling points was close to the risk threshold, resulting in HI close to 1 (HI = 0.977). In the stage of high river flow, there is a point close to the risk threshold and a point above the risk threshold (HI = 1.30). Based on the analysis presented in this study, the potentially toxic elements Zn, Pb, Cu, and As may have been affected by human activities. However, according to the results of the human health risk assessment, both non-carcinogenic and carcinogenic risks are low. This might be because as a traditional agricultural and pastoral area, the Kyrgyz region has relatively low point source and non-point source pollution, and the level of pollution from human activities is very low. It should be noted that for both the dry season and the rainy season, the maximum carcinogenic risk index for As exceeded the allowable value and requires further consideration. Due to the carcinogenicity of the element As, during the low water flow, the As content of five sampling points out of 30 sampling points was higher than the carcinogenicity threshold. Correspondingly, during the high river flow, the As content of six sampling points was higher than the carcinogenicity threshold. Compared with the low river flow period, the increase in flow did not cause the concentration dilution effect to occur. On the contrary, the pollution degree of heavy metals increased to a certain extent, which may reflect the difference of the intensity of human social activities in different periods.

**Table 2:** Statistical summary of the human health risk assessment results for the ingestion of potentially toxic elements (PTE) in river waters of the Chu-Talas Basin during low river flow (L) and high river flow (H) periods.

Stage	PTE	Minimum	Maximum	Mean	Median	Stage
L	Pb	HQ_ing_	3.60E−04	2.33E−02	7.27E−03	5.56E−03
		HQ_derm_	1.25E−05	8.11E−04	2.53E−04	1.93E−04
		HI	3.73E−04	2.41E−02	7.52E−03	5.75E−03
	Zn	HQ_ing_	1.01E−04	9.00E−04	4.54E−04	4.65E−04
		HQ_derm_	1.58E−06	1.41E−05	7.11E−06	7.29E−06
		HI	1.03E−04	9.14E−04	4.61E−04	4.72E−04
	Cu	HQ_ing_	2.58E−04	6.55E−03	1.57E−03	1.23E−03
		HQ_derm_	4.48E−06	1.14E−04	2.74E−05	2.14E−05
		HI	2.62E−04	6.66E−03	1.60E−03	1.25E−03
	Cr	HQ_ing_	1.83E−01	5.48E−01	3.11E−01	2.74E−01
		HQ_derm_	1.43E−01	4.29E−01	2.43E−01	2.15E−01
		HI	3.26E−01	9.77E−01	5.54E−01	4.89E−01
	As	HQ_ing_	2.95E−04	3.12E−03	1.22E−03	8.02E−04
		HQ_derm_	3.76E−04	3.97E−03	1.55E−03	1.02E−03
		HI	6.71E−04	7.09E−03	2.77E−03	1.82E−03
		CR_ing_	1.33E−05	1.40E−04	5.49E−05	3.61E−05
		CR_derm_	1.69E−07	1.79E−06	6.99E−07	4.60E−07
		CI	1.35E−05	1.42E−04	5.56E−05	3.66E−05
H	Pb	HQ_ing_	4.23E−04	3.64E−02	8.58E−03	5.21E−03
		HQ_derm_	1.47E−05	1.27E−03	2.99E−04	1.81E−04
		HI	4.38E−04	3.77E−02	8.88E−03	5.39E−03
	Zn	HQ_ing_	8.68E−05	2.07E−03	4.61E−04	2.84E−04
		HQ_derm_	1.36E−06	3.24E−05	7.22E−06	4.44E−06
		HI	8.82E−05	2.10E−03	4.68E−04	2.88E−04
	Cu	HQ_ing_	1.05E−04	8.12E−03	1.51E−03	7.01E−04
		HQ_derm_	1.84E−06	1.41E−04	2.63E−05	1.22E−05
		HI	1.07E−04	8.26E−03	1.54E−03	7.13E−04
	Cr	HQ_ing_	1.83E−01	7.31E−01	3.38E−01	3.65E−01
		HQ_derm_	1.43E−01	5.72E−01	2.65E−01	2.86E−01
		HI	3.26E−01	1.30E+00	6.03E−01	6.51E−01
	As	HQ_ing_	2.47E−04	3.03E−03	1.15E−03	8.26E−04
		HQ_derm_	3.15E−04	3.86E−03	1.46E−03	1.05E−03
		HI	5.62E−04	6.89E−03	2.61E−03	1.88E−03
		CR_ing_	1.11E−05	1.36E−04	5.17E−05	3.72E−05
		CR_derm_	1.42E−07	1.74E−06	6.58E−07	4.73E−07
		CI	1.12E−05	1.38E−04	5.24E−05	3.77E−05

## Conclusions

Based on the analysis of major ions and potentially toxic elements in river water of the Chu-Talas Basin, the factors affecting the main chemical composition were studied, and the irrigation suitability of river water and the human health risk of potentially toxic elements was evaluated. The main conclusions are as follows:

1. Although major ions in the river bodies of the basin are mainly affected by carbonate dissolution and silicate weathering, the sources of major ions change to some extent during different hydrological periods, but the hydrochemical type remains unchanged. Rivers in the basin are of the calcium carbonate hydrochemical type.
2. The relationship between the potentially toxic elements Zn, Pb, Cu, Cr, and As and major ions is basically the same under low and high river flow periods. There are significant differences in the sources of potentially toxic elements (Zn, Pb, Cu, and As) and major ions; however, the heavy metal Cr may share the same rock source as the major ions.
3. River water in the basin is classified as excellent/good for irrigation use based on the water quality assessment. According to the risk assessment, both non-carcinogenic and carcinogenic risks for human health are low. However, the maximum carcinogenic risk index for As exceeds the allowable during periods of both low and high river flow, which requires further consideration.

## Materials and methods

### Sampling and analysis

**Figure 7:**
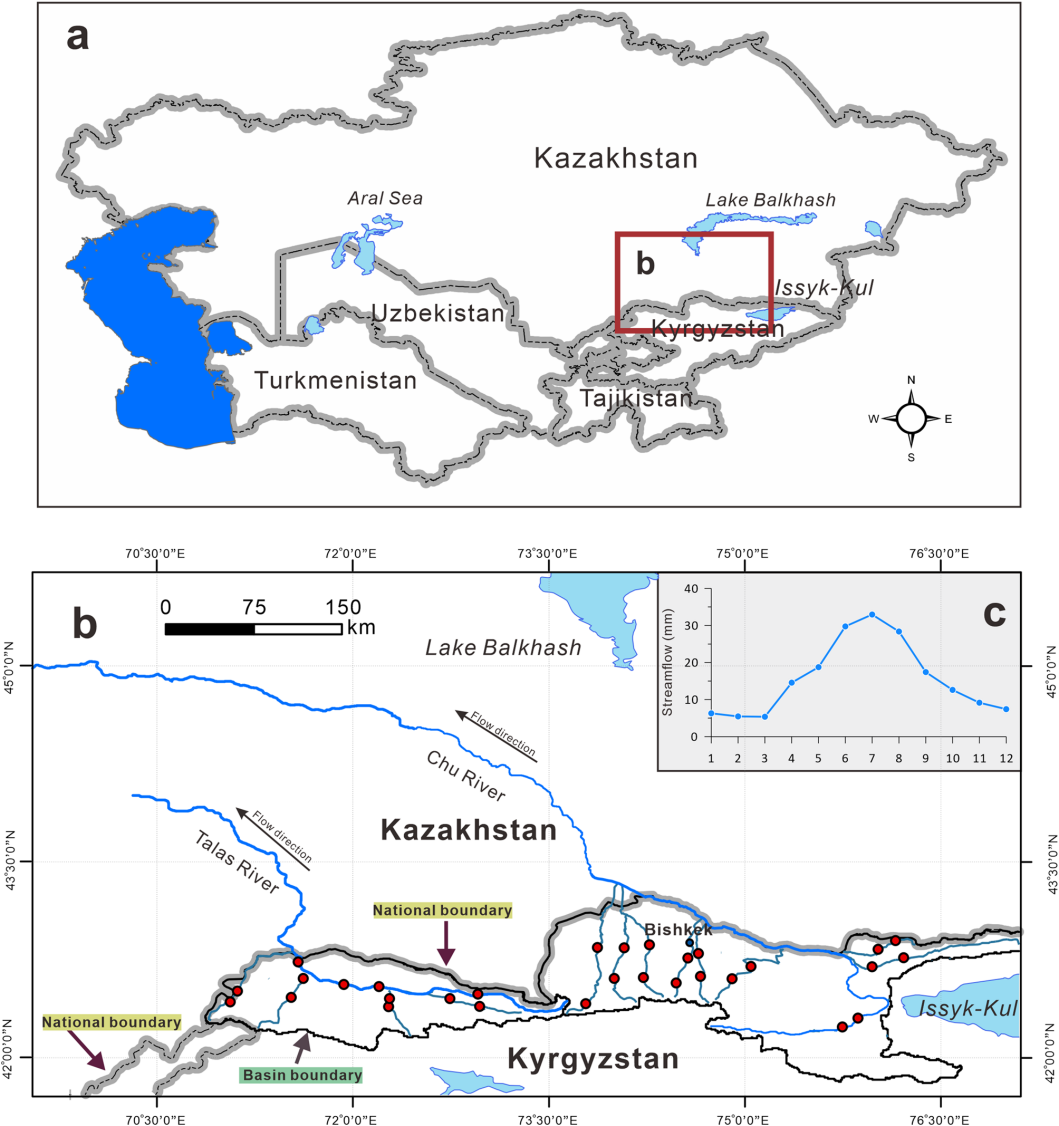
Geographical location map of the research area in Central Asia (**a**), the distribution of sampling site (**b**) with red dot, and the monthly distribution curve for the streamflow of River Chu (**c**). The diagrams of a and b were generated by QGIS 3.14 (https://www.qgis.org/) with the GADM Database of Global Administrative Areas (https://gadm.org), the Product of the Transboundary Freshwater Dispute Database (https://transboundarywaters.science.oregonstate.edu), and the Global Lakes and Wetlands Database (https://www.worldwildlife.org/pages/global-lakes-and-wetlands-database). The monthly distribution curve was generated by OriginPro 2020 (64-bit) SR1 9.7.0.188 (Learning Edition, https://www.originlab.com) with the data of streamflow of River Chu (1966–1955)^52^. The combination of three diagrams a, b, and c was conducted with the software Inkscape 1.0 (https://inkscape.org).

Sampling locations are shown in Fig. 7 and the geographic information for sampling points of surface rivers in Chu and Talas Basin during dry period with low river flow (May, 2017) and wet period with high river flow (July and August, 2017) were summarized in Table 3. Water samples from the Talas and Chu river basins were collected from 60 sites (Chu River sub-basin: n = 18, C01–C18; Talas River sub-basin: n = 12, T01–T12) during the low river flow period (May 2017) and again during the high river flow period (July and August 2017). In order to prevent contamination, river water samples were stored in clean 1.5-L polyethylene terephthalate bottles. Sub-samples were filtered through a 0.45-μm filter and stored in a high-density polyethylene tube until the analysis. Prior to the analysis of cations and potentially toxic elements, water samples were acidified with HNO_3_ (65%) (pH < 2). During the collection of river water, the temperature, pH, electrical conductivity (EC), and total dissolved solids (TDS) were measured in situ with an HI 9828 multi-parameter water quality meter (Hanna Instruments, Italy).

**Table 3:** The geographic information for sampling points of surface rivers in Chu and Talas Basin during dry period with low river flow and wet period with high river flow.

Sampling sites	Elavation (m)	Latitude (° N)	Longitude (° E)	River name	River type	Sampling date		Basin
						Low river flow	High river flow	
C01	1,370	42.601229	74.006862	Ak-Suu (Chu)	Tributary	2017/5/5	2017/7/25	Chu
C02	734	42.836333	74.083778	Ak-Suu (Chu)	Tributary	2017/5/5	2017/7/25	Chu
C03	2,310	42.892347	76.157715	Ak-Tuz	Tributary	2017/5/27	2017/7/26	Chu
C04	1664	42.825144	76.024209	Ak-Tuz	Tributary	2017/5/27	2017/7/26	Chu
C05	1703	42.760964	76.22061	Chon-Kemin	Tributary	2017/5/25	2017/7/27	Chu
C06	1,401	42.691826	75.980247	Chon-Kemin	Tributary	2017/5/25	2017/7/27	Chu
C07	1802	42.226087	75.749446	Kochkor	Main Stream	2017/5/13	2017/8/12	Chu
C08	1,750	42.297176	75.872212	Kochkor	Main Stream	2017/5/13	2017/8/12	Chu
C09	1501	42.607957	74.229199	Sokuluk	Tributary	2017/5/5	2017/8/15	Chu
C10	719	42.859702	74.275902	Sokuluk	Tributary	2017/5/5	2017/8/15	Chu
C11	2,131	42.566487	74.4794752	Ala-Archa	Tributary	2017/5/6	2017/8/15	Chu
C12	1,077	42.758265	74.570389	Ala-Archa	Tributary	2017/5/6	2017/8/15	Chu
C13	1,490	42.618211	74.665793	Alamedin	Tributary	2017/5/7	2017/8/16	Chu
C14	1,008	42.791352	74.649447	Alamedin	Tributary	2017/5/7	2017/8/16	Chu
C15	1862	42.599819	74.907476	Isik-Ata	Tributary	2017/5/28	2017/8/16	Chu
C16	1,161	42.693147	75.052709	Isik-Ata	Tributary	2017/5/28	2017/8/16	Chu
C17	2070	42.40834	73.788627	Kara-Balta	Tributary	2017/5/29	2017/7/24	Chu
C18	742	42.839239	73.879112	Kara-Balta	Tributary	2017/5/29	2017/7/24	Chu
T01	1958	42.483125	72.963804	Talas	Main Stream	2017/5/27	2017/7/25	Tals
T02	1716	42.44727	72.749625	Uch-Koshoi	Tributary	2017/5/27	2017/7/25	Tals
T03	1,195	42.540373	72.206252	Talas	Main Stream	2017/5/25	2017/7/26	Tals
T04	1558	42.385749	72.28057	Besh-Tash	Tributary	2017/5/26	2017/7/26	Tals
T05	1,411	42.447463	72.286084	Besh-Tash	Tributary	2017/5/26	2017/7/26	Tals
T06	1,304	42.388164	72.977375	Urmaral	Tributary	2017/5/24	2017/7/27	Tals
T07	1,036	42.556107	71.938244	Talas	Main Stream	2017/5/24	2017/7/27	Tals
T08	1,247	42.45683	71.535456	Kara-Buura	Tributary	2017/5/23	2017/7/28	Tals
T09	928	42.601067	71.628187	Kara-Buura	Tributary	2017/5/23	2017/7/28	Tals
T10	772	42.728007	71.589141	Talas	Main Stream	2017/5/21	2017/7/29	Tals
T11	1637	42.422344	71.067557	Kurkuroo	Tributary	2017/5/22	2017/7/30	Tals
T12	1,215	42.505295	71.122779	Kurkuroo	Tributary	2017/5/22	2017/7/30	Tals

All of the laboratory analyses were performed at the Research Center for Ecology and Environment of Central Asia (Bishkek), Kyrgyzstan. The river water in the study area is weakly alkaline. According to the equilibrium relationship between the dissolved CO_3_^2−^ and HCO_3_^−^ in water, the content of ion CO_3_^2−^ is very small, accounting for less than 5% of total. Therefore, the content of CO_3_^2−^ was ignored in this study. HCO_3_^−^ was measured by potentiometric titration with a G20 compact titrator (Mettler Toledo AG, Switzerland). Cations Ca^2+^, K^+^, Mg^2+^, and Na^+^ and anions Cl^−^, NO_3_^−^, and SO_4_^2−^ were determined using a Dionex ICS 900 ionic chromatography system (Thermo Fisher Scientific Inc., USA). The charge balance error percentage (CBE)^53,54^ was less than 5%. Potentially toxic elements Zn, Cu, Pb, Cr, and As were measured with an Agilent 8,800 inductively coupled plasma mass spectrometer (Agilent Technologies, USA).

### Hydrochemical diagram and chemical speciation modeling

A Wilcox (1948) diagram with EC and sodium percentage (Na%)^49,55,56^ was used for assessing the irrigation suitability of river waters. Piper diagrams^57–59^, Gibbs diagrams^60–64^, and mixing diagrams^65–67^ were used to determine influences of the river hydrochemistry. By using the software Phreeqc Interactive 3.6.2 with database file (minteq.v4.dat)^68^, chemical speciation modeling was conducted with major ions and the whole potentially toxic elements.

### Human health risk assessment

For potentially toxic elements in aqueous systems, there are two common human exposure pathways: (a) direct ingestion and (b) dermal absorption^69–71^. The human health risks for non-carcinogenic and carcinogenic elements were calculated using Eqs. (1)–(8) below. Parameters used in the equations are defined in Table 4.

**Table 4:** Definitions and values of parameters used in Eqs. (1)–(8) for human health risk calculations for Potentially toxic element (PTE).

Parameters	Meaning	Value	PTE	RfD_ing_^74^	RfD_derm_^74^	CSF_ing_^74^	CSF_derm_^74^
C_hm_	Concentration of potentially toxic elements	mg L^−1^	Zn	3.00E−01	6.00E−02	–	–
IngR	Ingestion rate	2.0 L day^−1^^75^	Cu	4.00E−02	1.20E−02	–	–
EF	Exposure frequency	350 day year^−1^^75^	Pb	3.50E−03	5.25E−04	–	–
ED	Exposure duration	70 year^75^	Cr	3.00E−03	6.00E−05	–	–
BW	Body weight	70 kg^76^	As	3.00E−04	1.23E−04	1.50E+00	3.66E+00
AT	average life time	25,550 days^75^					
SA	Drinking water exposed skin area	18,000 cm^2^^75^					
K_p_	Coefficient for dermal permeability	0.001 cm h^−1^^75^; Cr 0.003^75^; Zn 0.0006^75^					
ET	exposure time	0.58 h day^−1^^75^					
F_1_	Factor for unit conversion	L 1,000 cm^−1^^77^					

Daily exposure dose through oral intake (ADD_ing_):

1 $$ADD_{ing} = C_{hm} \times \frac{IngR \times EF \times ED}{{BW \times AT}}$$

Daily exposure dose through dermal contact (ADD_derm_):

2 $$ADD_{derm} = C_{hm} \times \frac{{SA \times K_{p} \times ET \times EF \times ED \times f_{1} }}{BW \times AT}$$

Hazard quotient for oral exposure (HQ_ing_):

3 $$HQ_{ing} = ADD_{ing} /RfD_{ing}$$

Hazard quotient for dermal exposure (HQ_derm_):

4 $${\text{HQ}}_{{{\text{derm}}}} {\text{ } = \text{ ADD}}_{{{\text{derm}}}} {\text{/RfD}}_{{{\text{derm}}}}$$

Hazard index (HI):

$$HI = HQ_{ing} + HQ_{derm}$$

Carcinogenic risks for oral exposure (CR_ing_):

6 $$CR_{ing} = ADD_{ing} \times CSF_{ing}$$

Carcinogenic risks for dermal exposure (CR_derm_):

7 $$CR_{derm} = ADD_{derm} \times CSF_{derm}$$

Total potential carcinogenic risks (CI):

8 $$CI = CR_{ing} + CR_{derm}$$

The values for Oral reference value (RfD_ing_, mg kg^−1^ day^−1^), dermal reference value (RfD_derm_), oral cancer slope factor (CSF_ing_, (mg kg^−1^ day^−1^)^−1^) and dermal cancer slope factor (CSF_derm_) were also shown in Table 4. HI < 1 or HQ < 1: no non-carcinogenic risks; HI > 1 or HQ > 1: there is potential risk for non-carcinogenic effects^72^; Acceptable or tolerable range of carcinogenic risks: 10^−6^–10^−4^^73^.

## Supplementary information

Supplementary Information.
