# Supplementary material for: Hydrochemical composition and potentially toxic elements in the Kyrgyzstan portion of the transboundary Chu-Talas river basin, Central Asia
Source: Sci Rep. 2020 Sep 11;10:14972. doi: 10.1038/s41598-020-71880-4 (PMC7486924; doi:10.1038/s41598-020-71880-4)
Supplement: Supplementary file 1 — Supplementary Information. [file 41598_2020_71880_MOESM1_ESM.docx]

**Supplementary information**

Title of manuscript:

**Hydrochemical composition and** **potentially toxic elements in the Kyrgyzstan portion of the transboundary Chu-Talas river basin, Central Asia**

Names of authors:

Long Ma, Yaoming Li, Jilili Abuduwaili, Salamat Abdyzhapar uulu, Wen Liu

Table S1. **Independent Samples Test**

|  |  | Levene's Test for Equality of Variances | | t-test for Equality of Means | | | | | | |
| --- | --- | --- | --- | --- | --- | --- | --- | --- | --- | --- |
|  |  | F | Sig. | t | df | Sig. (2-tailed) | Mean Difference | Std. Error Difference | 95% Confidence Interval of the Difference | |
|  |  |  |  |  |  |  |  |  | Lower | Upper |
| Ca | Equal variances assumed | 0.000 | 0.987 | -0.842 | 58 | 0.403 | -2.746 | 3.262 | -9.276 | 3.784 |
|  | Equal variances not assumed |  |  | -0.842 | 58 | 0.403 | -2.746 | 3.262 | -9.276 | 3.784 |
| Cl | Equal variances assumed | 0.001 | 0.980 | -0.656 | 58 | 0.514 | -0.453 | 0.690 | -1.834 | 0.928 |
|  | Equal variances not assumed |  |  | -0.656 | 58 | 0.514 | -0.453 | 0.690 | -1.834 | 0.928 |
| As | Equal variances assumed | 0.019 | 0.892 | -0.293 | 58 | 0.771 | -0.077 | 0.264 | -0.607 | 0.453 |
|  | Equal variances not assumed |  |  | -0.293 | 58 | 0.771 | -0.077 | 0.264 | -0.607 | 0.453 |
| Cd | Equal variances assumed | 8.933 | 0.004 | -2.377 | 58 | 0.022 | -2.536 | 1.067 | -4.681 | -0.391 |
|  | Equal variances not assumed |  |  | -2.377 | 58 | 0.026 | -2.536 | 1.067 | -4.738 | -0.334 |
| Cu | Equal variances assumed | 3.236 | 0.078 | -0.287 | 58 | 0.775 | -0.201 | 0.699 | -1.602 | 1.200 |
|  | Equal variances not assumed |  |  | -0.287 | 58 | 0.775 | -0.201 | 0.699 | -1.608 | 1.206 |
| HCO3 | Equal variances assumed | 0.192 | 0.663 | -0.746 | 58 | 0.458 | -12.723 | 17.045 | -46.841 | 21.395 |
|  | Equal variances not assumed |  |  | -0.746 | 58 | 0.458 | -12.723 | 17.045 | -46.842 | 21.396 |
| K | Equal variances assumed | 0.085 | 0.772 | -1.645 | 58 | 0.105 | -0.192 | 0.117 | -0.425 | 0.042 |
|  | Equal variances not assumed |  |  | -1.645 | 58 | 0.105 | -0.192 | 0.117 | -0.425 | 0.042 |
| Mg | Equal variances assumed | 0.003 | 0.955 | -0.554 | 58 | 0.582 | -0.746 | 1.347 | -3.442 | 1.950 |
|  | Equal variances not assumed |  |  | -0.554 | 58 | 0.582 | -0.746 | 1.347 | -3.442 | 1.950 |
| Na | Equal variances assumed | 0.060 | 0.807 | -0.966 | 58 | 0.338 | -0.983 | 1.018 | -3.019 | 1.054 |
|  | Equal variances not assumed |  |  | -0.966 | 58 | 0.338 | -0.983 | 1.018 | -3.019 | 1.054 |
| NO_3_ | Equal variances assumed | 1.353 | 0.249 | -2.958 | 58 | 0.004 | -0.886 | 0.299 | -1.485 | -0.286 |
|  | Equal variances not assumed |  |  | -2.958 | 58 | 0.004 | -0.886 | 0.299 | -1.485 | -0.286 |
| Pb | Equal variances assumed | 3.647 | 0.061 | 0.654 | 58 | 0.515 | 0.167 | 0.255 | -0.344 | 0.677 |
|  | Equal variances not assumed |  |  | 0.654 | 58 | 0.516 | 0.167 | 0.255 | -0.345 | 0.679 |
| pH | Equal variances assumed | 5.096 | 0.031 | 2.659 | 33.000 | 0.012 | 0.427 | 0.161 | 0.100 | 0.754 |
|  | Equal variances not assumed |  |  | 2.642 | 30.566 | 0.013 | 0.427 | 0.162 | 0.097 | 0.758 |
| SO4 | Equal variances assumed | 1.107 | 0.297 | -1.367 | 58.000 | 0.177 | -5.141 | 3.760 | -12.668 | 2.385 |
|  | Equal variances not assumed |  |  | -1.367 | 50.748 | 0.178 | -5.141 | 3.760 | -12.691 | 2.408 |
| Zn | Equal variances assumed | 7.567 | 0.008 | 0.074 | 58.000 | 0.941 | 0.076 | 1.020 | -1.967 | 2.118 |
|  | Equal variances not assumed |  |  | 0.074 | 38.993 | 0.941 | 0.076 | 1.020 | -1.988 | 2.140 |
| Cr | Equal variances assumed | 0.352 | 0.555 | 0.727 | 56.000 | 0.470 | 0.002 | 0.003 | -0.004 | 0.008 |
|  | Equal variances not assumed |  |  | 0.727 | 53.093 | 0.470 | 0.002 | 0.003 | -0.004 | 0.008 |
| EC | Equal variances assumed | 3.364 | 0.072 | -0.565 | 58.000 | 0.575 | -17.283 | 30.615 | -78.567 | 44.000 |
|  | Equal variances not assumed |  |  | -0.565 | 54.416 | 0.575 | -17.283 | 30.615 | -78.653 | 44.086 |
| TDS | Equal variances assumed | 0.018 | 0.894 | -0.953 | 58.000 | 0.344 | -23.977 | 25.149 | -74.318 | 26.363 |
|  | Equal variances not assumed |  |  | -0.953 | 57.744 | 0.344 | -23.977 | 25.149 | -74.323 | 26.368 |

# PART A: Chemical speciation modeling with the software Phreeqc Interactive 3.6.2 with database file (minteq.v4.dat) for river waters at the stage with low river flow

Initial solution 1.

----------------------------Distribution of species----------------------------

Log Log Log mole V

Species Molality Activity Molality Activity Gamma cm?mol

OH- 1.489e-07 1.365e-07 -6.827 -6.865 -0.038 (0)

H+ 2.439e-08 2.239e-08 -7.613 -7.650 -0.037 0.00

H2O 5.551e+01 9.999e-01 1.744 -0.000 0.000 18.02

As(3) 4.476e-18

H3AsO3 4.414e-18 4.414e-18 -17.355 -17.355 0.000 (0)

H2AsO3- 6.193e-20 5.629e-20 -19.208 -19.250 -0.041 (0)

HAsO3-2 1.696e-24 1.157e-24 -23.771 -23.937 -0.166 (0)

H4AsO3+ 5.386e-26 4.896e-26 -25.269 -25.310 -0.041 (0)

AsO3-3 2.741e-30 1.160e-30 -29.562 -29.935 -0.373 (0)

As(5) 1.782e-08

HAsO4-2 1.531e-08 1.045e-08 -7.815 -7.981 -0.166 (0)

H2AsO4- 2.503e-09 2.275e-09 -8.601 -8.643 -0.041 (0)

AsO4-3 2.425e-12 1.027e-12 -11.615 -11.989 -0.373 (0)

H3AsO4 7.593e-15 7.605e-15 -14.120 -14.119 0.001 (0)

C(4) 3.342e-03

HCO3- 3.108e-03 2.860e-03 -2.508 -2.544 -0.036 (0)

H2CO3 1.751e-04 1.751e-04 -3.757 -3.757 0.000 (0)

CaHCO3+ 3.866e-05 3.561e-05 -4.413 -4.448 -0.036 (0)

MgHCO3+ 7.643e-06 7.012e-06 -5.117 -5.154 -0.037 (0)

CO3-2 6.178e-06 4.383e-06 -5.209 -5.358 -0.149 (0)

CaCO3 5.060e-06 5.060e-06 -5.296 -5.296 0.000 (0)

MgCO3 7.364e-07 7.364e-07 -6.133 -6.133 0.000 (0)

NaHCO3 4.825e-07 4.825e-07 -6.316 -6.316 0.000 (0)

CuCO3 3.277e-08 3.277e-08 -7.485 -7.485 0.000 (0)

NaCO3- 3.067e-08 2.822e-08 -7.513 -7.550 -0.036 (0)

ZnCO3 9.027e-09 9.027e-09 -8.044 -8.044 0.000 (0)

CdCO3 4.107e-09 4.107e-09 -8.386 -8.386 0.000 (0)

ZnHCO3+ 2.606e-09 2.369e-09 -8.584 -8.625 -0.041 (0)

PbCO3 2.599e-09 2.599e-09 -8.585 -8.585 0.000 (0)

Cu(CO3)2-2 5.665e-10 3.866e-10 -9.247 -9.413 -0.166 (0)

PbHCO3+ 3.375e-10 3.067e-10 -9.472 -9.513 -0.041 (0)

CdHCO3+ 2.155e-10 1.959e-10 -9.666 -9.708 -0.041 (0)

CuHCO3+ 1.845e-10 1.677e-10 -9.734 -9.776 -0.041 (0)

Pb(CO3)2-2 4.814e-11 3.285e-11 -10.317 -10.483 -0.166 (0)

Cd(CO3)2-2 1.956e-11 1.335e-11 -10.709 -10.875 -0.166 (0)

Ca 1.591e-03

Ca+2 1.445e-03 1.025e-03 -2.840 -2.989 -0.149 (0)

CaSO4 1.008e-04 1.008e-04 -3.997 -3.997 0.000 (0)

CaHCO3+ 3.866e-05 3.561e-05 -4.413 -4.448 -0.036 (0)

CaCO3 5.060e-06 5.060e-06 -5.296 -5.296 0.000 (0)

CaNO3+ 1.150e-06 1.045e-06 -5.939 -5.981 -0.041 (0)

CaOH+ 2.538e-09 2.338e-09 -8.595 -8.631 -0.036 (0)

Cd 6.659e-08

Cd+2 5.794e-08 4.111e-08 -7.237 -7.386 -0.149 (0)

CdCO3 4.107e-09 4.107e-09 -8.386 -8.386 0.000 (0)

CdSO4 3.996e-09 3.996e-09 -8.398 -8.398 0.000 (0)

CdHCO3+ 2.155e-10 1.959e-10 -9.666 -9.708 -0.041 (0)

CdCl+ 1.506e-10 1.369e-10 -9.822 -9.864 -0.041 (0)

CdNO3+ 6.435e-11 5.849e-11 -10.191 -10.233 -0.041 (0)

CdOH+ 5.009e-11 4.552e-11 -10.300 -10.342 -0.041 (0)

Cd(SO4)2-2 4.751e-11 3.242e-11 -10.323 -10.489 -0.166 (0)

Cd(CO3)2-2 1.956e-11 1.335e-11 -10.709 -10.875 -0.166 (0)

CdOHCl 1.748e-12 1.748e-12 -11.757 -11.757 0.000 (0)

Cd(OH)2 4.167e-13 4.167e-13 -12.380 -12.380 0.000 (0)

CdCl2 1.948e-14 1.948e-14 -13.710 -13.710 0.000 (0)

Cd(NO3)2 5.376e-15 5.376e-15 -14.270 -14.270 0.000 (0)

Cd2OH+3 2.686e-17 1.137e-17 -16.571 -16.944 -0.373 (0)

Cd(OH)3- 1.260e-17 1.145e-17 -16.900 -16.941 -0.041 (0)

CdCl3- 4.147e-19 3.769e-19 -18.382 -18.424 -0.041 (0)

Cd(OH)4-2 1.235e-24 8.429e-25 -23.908 -24.074 -0.166 (0)

Cl 3.881e-05

Cl- 3.881e-05 3.562e-05 -4.411 -4.448 -0.037 (0)

CdCl+ 1.506e-10 1.369e-10 -9.822 -9.864 -0.041 (0)

ZnCl+ 3.110e-12 2.853e-12 -11.507 -11.545 -0.037 (0)

CuCl 2.406e-12 2.406e-12 -11.619 -11.619 0.000 (0)

ZnOHCl 1.885e-12 1.885e-12 -11.725 -11.725 0.000 (0)

CdOHCl 1.748e-12 1.748e-12 -11.757 -11.757 0.000 (0)

PbCl+ 2.277e-13 2.070e-13 -12.643 -12.684 -0.041 (0)

CuCl+ 6.543e-14 6.003e-14 -13.184 -13.222 -0.037 (0)

CuCl2- 2.027e-14 1.859e-14 -13.693 -13.731 -0.037 (0)

CdCl2 1.948e-14 1.948e-14 -13.710 -13.710 0.000 (0)

CrCl+2 4.666e-16 3.185e-16 -15.331 -15.497 -0.166 (0)

ZnCl2 8.199e-17 8.199e-17 -16.086 -16.086 0.000 (0)

PbCl2 3.069e-17 3.069e-17 -16.513 -16.513 0.000 (0)

CdCl3- 4.147e-19 3.769e-19 -18.382 -18.424 -0.041 (0)

CuCl2 3.444e-19 3.444e-19 -18.463 -18.463 0.000 (0)

CuCl3-2 1.865e-19 1.333e-19 -18.729 -18.875 -0.146 (0)

CrOHCl2 1.059e-20 1.059e-20 -19.975 -19.975 0.000 (0)

ZnCl3- 2.372e-21 2.176e-21 -20.625 -20.662 -0.037 (0)

PbCl3- 5.682e-22 5.164e-22 -21.246 -21.287 -0.041 (0)

CrCl2+ 2.991e-22 2.718e-22 -21.524 -21.566 -0.041 (0)

CuCl3- 9.433e-26 8.654e-26 -25.025 -25.063 -0.037 (0)

ZnCl4-2 4.787e-26 3.420e-26 -25.320 -25.466 -0.146 (0)

PbCl4-2 9.788e-27 6.680e-27 -26.009 -26.175 -0.166 (0)

CrO3Cl- 6.007e-30 5.459e-30 -29.221 -29.263 -0.041 (0)

CuCl4-2 3.669e-32 2.621e-32 -31.435 -31.582 -0.146 (0)

Cr(2) 2.306e-23

Cr+2 2.306e-23 1.574e-23 -22.637 -22.803 -0.166 (0)

Cr(3) 5.770e-07

Cr(OH)2+ 4.470e-07 4.063e-07 -6.350 -6.391 -0.041 (0)

Cr(OH)3 6.863e-08 6.863e-08 -7.163 -7.163 0.000 (0)

Cr(OH)+2 5.749e-08 3.924e-08 -7.240 -7.406 -0.166 (0)

CrO2- 1.602e-09 1.456e-09 -8.795 -8.837 -0.041 (0)

Cr(OH)4- 1.352e-09 1.229e-09 -8.869 -8.911 -0.041 (0)

CrOHSO4 8.798e-10 8.798e-10 -9.056 -9.056 0.000 (0)

Cr+3 2.844e-11 1.204e-11 -10.546 -10.919 -0.373 (0)

CrSO4+ 7.965e-12 7.239e-12 -11.099 -11.140 -0.041 (0)

Cr2(OH)2SO4+2 8.651e-16 5.904e-16 -15.063 -15.229 -0.166 (0)

CrCl+2 4.666e-16 3.185e-16 -15.331 -15.497 -0.166 (0)

CrNO3+2 5.620e-17 3.836e-17 -16.250 -16.416 -0.166 (0)

Cr2(OH)2(SO4)2 1.751e-17 1.751e-17 -16.757 -16.757 0.000 (0)

CrOHCl2 1.059e-20 1.059e-20 -19.975 -19.975 0.000 (0)

CrCl2+ 2.991e-22 2.718e-22 -21.524 -21.566 -0.041 (0)

Cr(6) 2.234e-17

CrO4-2 2.117e-17 1.502e-17 -16.674 -16.823 -0.149 (0)

HCrO4- 1.147e-18 1.043e-18 -17.940 -17.982 -0.041 (0)

NaCrO4- 1.838e-20 1.671e-20 -19.736 -19.777 -0.041 (0)

KCrO4- 2.222e-21 2.020e-21 -20.653 -20.695 -0.041 (0)

H2CrO4 8.583e-27 8.583e-27 -26.066 -26.066 0.000 (0)

CrO3SO4-2 5.431e-27 3.707e-27 -26.265 -26.431 -0.166 (0)

CrO3Cl- 6.007e-30 5.459e-30 -29.221 -29.263 -0.041 (0)

Cr2O7-2 8.294e-35 5.660e-35 -34.081 -34.247 -0.166 (0)

Cu(1) 6.147e-11

Cu+ 5.904e-11 5.366e-11 -10.229 -10.270 -0.041 (0)

CuCl 2.406e-12 2.406e-12 -11.619 -11.619 0.000 (0)

CuCl2- 2.027e-14 1.859e-14 -13.693 -13.731 -0.037 (0)

CuCl3-2 1.865e-19 1.333e-19 -18.729 -18.875 -0.146 (0)

Cu(2) 3.651e-08

CuCO3 3.277e-08 3.277e-08 -7.485 -7.485 0.000 (0)

Cu+2 1.789e-09 1.270e-09 -8.747 -8.896 -0.149 (0)

CuOH+ 9.156e-10 8.400e-10 -9.038 -9.076 -0.037 (0)

Cu(CO3)2-2 5.665e-10 3.866e-10 -9.247 -9.413 -0.166 (0)

CuHCO3+ 1.845e-10 1.677e-10 -9.734 -9.776 -0.041 (0)

Cu(OH)2 1.620e-10 1.620e-10 -9.790 -9.790 0.000 (0)

CuSO4 1.206e-10 1.206e-10 -9.919 -9.919 0.000 (0)

CuNO3+ 1.385e-12 1.259e-12 -11.859 -11.900 -0.041 (0)

Cu(OH)3- 1.644e-13 1.495e-13 -12.784 -12.825 -0.041 (0)

CuCl+ 6.543e-14 6.003e-14 -13.184 -13.222 -0.037 (0)

Cu2(OH)2+2 2.334e-14 1.593e-14 -13.632 -13.798 -0.166 (0)

Cu(NO3)2 4.170e-17 4.170e-17 -16.380 -16.380 0.000 (0)

Cu(OH)4-2 7.752e-19 5.290e-19 -18.111 -18.277 -0.166 (0)

CuCl2 3.444e-19 3.444e-19 -18.463 -18.463 0.000 (0)

CuCl3- 9.433e-26 8.654e-26 -25.025 -25.063 -0.037 (0)

CuCl4-2 3.669e-32 2.621e-32 -31.435 -31.582 -0.146 (0)

H(0) 8.292e-27

H2 4.146e-27 4.152e-27 -26.382 -26.382 0.001 (0)

K 3.955e-05

K+ 3.943e-05 3.619e-05 -4.404 -4.441 -0.037 (0)

KSO4- 1.274e-07 1.172e-07 -6.895 -6.931 -0.036 (0)

KCrO4- 2.222e-21 2.020e-21 -20.653 -20.695 -0.041 (0)

Mg 3.972e-04

Mg+2 3.679e-04 2.610e-04 -3.434 -3.583 -0.149 (0)

MgSO4 2.095e-05 2.095e-05 -4.679 -4.679 0.000 (0)

MgHCO3+ 7.643e-06 7.012e-06 -5.117 -5.154 -0.037 (0)

MgCO3 7.364e-07 7.364e-07 -6.133 -6.133 0.000 (0)

MgOH+ 1.190e-08 1.097e-08 -7.924 -7.960 -0.035 (0)

N(5) 3.141e-04

NO3- 3.130e-04 2.872e-04 -3.504 -3.542 -0.037 (0)

CaNO3+ 1.150e-06 1.045e-06 -5.939 -5.981 -0.041 (0)

CdNO3+ 6.435e-11 5.849e-11 -10.191 -10.233 -0.041 (0)

ZnNO3+ 3.134e-11 2.849e-11 -10.504 -10.545 -0.041 (0)

CuNO3+ 1.385e-12 1.259e-12 -11.859 -11.900 -0.041 (0)

PbNO3+ 8.834e-13 8.029e-13 -12.054 -12.095 -0.041 (0)

Cd(NO3)2 5.376e-15 5.376e-15 -14.270 -14.270 0.000 (0)

Zn(NO3)2 1.480e-15 1.480e-15 -14.830 -14.830 0.000 (0)

Pb(NO3)2 4.707e-16 4.707e-16 -15.327 -15.327 0.000 (0)

CrNO3+2 5.620e-17 3.836e-17 -16.250 -16.416 -0.166 (0)

Cu(NO3)2 4.170e-17 4.170e-17 -16.380 -16.380 0.000 (0)

Na 2.450e-04

Na+ 2.438e-04 2.238e-04 -3.613 -3.650 -0.037 (0)

NaSO4- 6.385e-07 5.875e-07 -6.195 -6.231 -0.036 (0)

NaHCO3 4.825e-07 4.825e-07 -6.316 -6.316 0.000 (0)

NaCO3- 3.067e-08 2.822e-08 -7.513 -7.550 -0.036 (0)

NaCrO4- 1.838e-20 1.671e-20 -19.736 -19.777 -0.041 (0)

O(0) 0.000e+00

O2 0.000e+00 0.000e+00 -43.860 -43.859 0.001 (0)

Pb 3.560e-09

PbCO3 2.599e-09 2.599e-09 -8.585 -8.585 0.000 (0)

PbHCO3+ 3.375e-10 3.067e-10 -9.472 -9.513 -0.041 (0)

Pb+2 2.780e-10 1.972e-10 -9.556 -9.705 -0.149 (0)

PbOH+ 2.451e-10 2.228e-10 -9.611 -9.652 -0.041 (0)

PbSO4 4.824e-11 4.824e-11 -10.317 -10.317 0.000 (0)

Pb(CO3)2-2 4.814e-11 3.285e-11 -10.317 -10.483 -0.166 (0)

Pb(OH)2 3.169e-12 3.169e-12 -11.499 -11.499 0.000 (0)

PbNO3+ 8.834e-13 8.029e-13 -12.054 -12.095 -0.041 (0)

PbCl+ 2.277e-13 2.070e-13 -12.643 -12.684 -0.041 (0)

Pb(SO4)2-2 2.127e-13 1.452e-13 -12.672 -12.838 -0.166 (0)

Pb(OH)3- 1.568e-15 1.425e-15 -14.805 -14.846 -0.041 (0)

Pb(NO3)2 4.707e-16 4.707e-16 -15.327 -15.327 0.000 (0)

PbCl2 3.069e-17 3.069e-17 -16.513 -16.513 0.000 (0)

Pb2OH+3 1.645e-18 6.965e-19 -17.784 -18.157 -0.373 (0)

Pb(OH)4-2 2.300e-19 1.570e-19 -18.638 -18.804 -0.166 (0)

PbCl3- 5.682e-22 5.164e-22 -21.246 -21.287 -0.041 (0)

Pb3(OH)4+2 4.934e-24 3.367e-24 -23.307 -23.473 -0.166 (0)

PbCl4-2 9.788e-27 6.680e-27 -26.009 -26.175 -0.166 (0)

Pb4(OH)4+4 4.330e-29 9.393e-30 -28.363 -29.027 -0.664 (0)

S(6) 8.263e-04

SO4-2 7.038e-04 4.994e-04 -3.153 -3.302 -0.149 (0)

CaSO4 1.008e-04 1.008e-04 -3.997 -3.997 0.000 (0)

MgSO4 2.095e-05 2.095e-05 -4.679 -4.679 0.000 (0)

NaSO4- 6.385e-07 5.875e-07 -6.195 -6.231 -0.036 (0)

KSO4- 1.274e-07 1.172e-07 -6.895 -6.931 -0.036 (0)

CdSO4 3.996e-09 3.996e-09 -8.398 -8.398 0.000 (0)

ZnSO4 3.425e-09 3.425e-09 -8.465 -8.465 0.000 (0)

CrOHSO4 8.798e-10 8.798e-10 -9.056 -9.056 0.000 (0)

HSO4- 7.434e-10 6.828e-10 -9.129 -9.166 -0.037 (0)

CuSO4 1.206e-10 1.206e-10 -9.919 -9.919 0.000 (0)

PbSO4 4.824e-11 4.824e-11 -10.317 -10.317 0.000 (0)

Cd(SO4)2-2 4.751e-11 3.242e-11 -10.323 -10.489 -0.166 (0)

Zn(SO4)2-2 2.492e-11 1.701e-11 -10.603 -10.769 -0.166 (0)

CrSO4+ 7.965e-12 7.239e-12 -11.099 -11.140 -0.041 (0)

Pb(SO4)2-2 2.127e-13 1.452e-13 -12.672 -12.838 -0.166 (0)

Cr2(OH)2SO4+2 8.651e-16 5.904e-16 -15.063 -15.229 -0.166 (0)

Cr2(OH)2(SO4)2 1.751e-17 1.751e-17 -16.757 -16.757 0.000 (0)

CrO3SO4-2 5.431e-27 3.707e-27 -26.265 -26.431 -0.166 (0)

Zn 6.621e-08

Zn+2 5.044e-08 3.579e-08 -7.297 -7.446 -0.149 (0)

ZnCO3 9.027e-09 9.027e-09 -8.044 -8.044 0.000 (0)

ZnSO4 3.425e-09 3.425e-09 -8.465 -8.465 0.000 (0)

ZnHCO3+ 2.606e-09 2.369e-09 -8.584 -8.625 -0.041 (0)

ZnOH+ 5.373e-10 4.883e-10 -9.270 -9.311 -0.041 (0)

Zn(OH)2 1.147e-10 1.147e-10 -9.940 -9.940 0.000 (0)

ZnNO3+ 3.134e-11 2.849e-11 -10.504 -10.545 -0.041 (0)

Zn(SO4)2-2 2.492e-11 1.701e-11 -10.603 -10.769 -0.166 (0)

ZnCl+ 3.110e-12 2.853e-12 -11.507 -11.545 -0.037 (0)

ZnOHCl 1.885e-12 1.885e-12 -11.725 -11.725 0.000 (0)

Zn(OH)3- 2.845e-13 2.586e-13 -12.546 -12.587 -0.041 (0)

Zn(NO3)2 1.480e-15 1.480e-15 -14.830 -14.830 0.000 (0)

ZnCl2 8.199e-17 8.199e-17 -16.086 -16.086 0.000 (0)

Zn(OH)4-2 6.783e-18 4.629e-18 -17.169 -17.334 -0.166 (0)

ZnCl3- 2.372e-21 2.176e-21 -20.625 -20.662 -0.037 (0)

ZnCl4-2 4.787e-26 3.420e-26 -25.320 -25.466 -0.146 (0)

------------------------------Saturation indices-------------------------------

Phase SI** log IAP log K(283 K, 1 atm)

Anglesite -5.11 -13.01 -7.90 PbSO4

Anhydrite -2.00 -6.29 -4.29 CaSO4

Antlerite -8.18 0.61 8.79 Cu3(OH)4SO4

Aragonite -0.16 -8.35 -8.19 CaCO3

Arsenolite -66.10 -69.42 -3.32 As4O6

Artinite -7.94 2.77 10.72 MgCO3:Mg(OH)2:3H2O

As2O5 -35.15 -28.24 6.92 As2O5

Atacamite -7.55 0.71 8.26 Cu2(OH)3Cl

Azurite -6.08 -22.11 -16.02 Cu3(OH)2(CO3)2

Bianchite -8.99 -10.75 -1.76 ZnSO4:6H2O

Brochantite -10.09 7.01 17.10 Cu4(OH)6SO4

Brucite -6.19 11.72 17.90 Mg(OH)2

Ca3(AsO4)2:4H2O -13.61 8.69 22.30 Ca3(AsO4)2:4H2O

CaCrO4 -17.80 -19.81 -2.02 CaCrO4

Calcite 0.06 -8.35 -8.41 CaCO3

Cd(OH)2 -6.61 7.91 14.52 Cd(OH)2

Cd(OH)2(am) -6.62 7.91 14.54 Cd(OH)2

Cd3(OH)2(SO4)2 -20.17 -13.46 6.71 Cd3(OH)2(SO4)2

Cd3(OH)4SO4 -17.42 5.14 22.56 Cd3(OH)4SO4

Cd4(OH)6SO4 -15.35 13.05 28.40 Cd4(OH)6SO4

CdCl2 -15.80 -16.28 -0.49 CdCl2

CdCl2:1H2O -14.66 -16.28 -1.62 CdCl2:1H2O

CdCl2:2.5H2O -14.30 -16.28 -1.98 CdCl2:2.5H2O

Cdmetal(alpha) -29.60 -15.39 14.21 Cd

Cdmetal(gamma) -29.71 -15.39 14.32 Cd

CdOHCl -8.01 -4.18 3.82 CdOHCl

CdSO4 -11.00 -10.69 0.31 CdSO4

CdSO4:1H2O -9.25 -10.69 -1.43 CdSO4:1H2O

CdSO4:2.67H2O -8.98 -10.69 -1.71 CdSO4:2.67H2O

Cerussite -1.70 -15.06 -13.36 PbCO3

CH4(g) -70.43 -113.86 -43.43 CH4

Chalcanthite -9.50 -12.20 -2.70 CuSO4:5H2O

Claudetite -65.84 -69.42 -3.58 As4O6

CO2(g) -2.47 -20.66 -18.18 CO2

Cotunnite -13.58 -18.60 -5.02 PbCl2

Cr(OH)2 -18.65 -7.50 11.15 Cr(OH)2

Cr(OH)3 -0.35 1.26 1.61 Cr(OH)3

Cr(OH)3(am) 2.01 1.26 -0.75 Cr(OH)3

Cr2O3 4.40 2.52 -1.89 Cr2O3

CrCl2 -46.82 -31.70 15.12 CrCl2

CrCl3 -51.27 -35.04 16.24 CrCl3

Crmetal -62.88 -30.80 32.08 Cr

CrO3 -28.96 -32.12 -3.16 CrO3

Cu(OH)2 -2.79 6.40 9.20 Cu(OH)2

Cu2(OH)3NO3 -8.31 1.62 9.92 Cu2(OH)3NO3

Cu2SO4 -22.07 -23.84 -1.77 Cu2SO4

Cu3(AsO4)2:2H2O -15.13 -9.03 6.10 Cu3(AsO4)2:2H2O

CuCO3 -2.75 -14.25 -11.50 CuCO3

CuCrO4 -20.28 -25.72 -5.44 CuCrO4

Cumetal -4.85 -14.27 -9.42 Cu

CuOCuSO4 -17.38 -5.79 11.58 CuOCuSO4

Cuprite -4.99 -5.24 -0.26 Cu2O

CuSO4 -15.82 -12.20 3.62 CuSO4

Dolomite(disordered) -1.18 -17.29 -16.11 CaMg(CO3)2

Dolomite(ordered) -0.57 -17.29 -16.72 CaMg(CO3)2

Epsomite -4.65 -6.89 -2.23 MgSO4:7H2O

Goslarite -8.61 -10.75 -2.14 ZnSO4:7H2O

Gypsum -1.67 -6.29 -4.62 CaSO4:2H2O

Halite -9.67 -8.10 1.57 NaCl

Huntite -6.20 -35.17 -28.97 CaMg3(CO3)4

Hydrocerussite -5.76 -24.53 -18.77 Pb3(OH)2(CO3)2

Hydromagnesite -17.31 -24.05 -6.74 Mg5(CO3)4(OH)2:4H2O

K2Cr2O7 -39.84 -57.83 -17.99 K2Cr2O7

K2CrO4 -25.02 -25.71 -0.68 K2CrO4

Langite -12.01 7.01 19.03 Cu4(OH)6SO4:H2O

Larnakite -7.18 -7.41 -0.23 PbO:PbSO4

Laurionite -7.13 -6.50 0.62 PbOHCl

Lime -22.19 12.31 34.50 CaO

Litharge -7.71 5.59 13.30 PbO

Magnesite -1.30 -8.94 -7.65 MgCO3

Malachite -1.84 -7.85 -6.01 Cu2(OH)2CO3

Massicot -7.92 5.59 13.51 PbO

Melanothallite -24.64 -17.79 6.85 CuCl2

Mg(OH)2(active) -7.08 11.72 18.79 Mg(OH)2

MgCr2O4 -3.63 14.23 17.87 MgCr2O4

MgCrO4 -26.61 -20.41 6.21 MgCrO4

Minium -37.35 40.08 77.44 Pb3O4

Mirabilite -8.75 -10.60 -1.85 Na2SO4:10H2O

Monteponite -8.15 7.91 16.06 CdO

Na2Cr2O7 -46.15 -56.25 -10.10 Na2Cr2O7

Na2CrO4 -27.24 -24.12 3.11 Na2CrO4

Nantokite -7.59 -14.72 -7.13 CuCl

Natron -10.74 -12.66 -1.92 Na2CO3:10H2O

Nesquehonite -4.50 -8.94 -4.45 MgCO3:3H2O

O2(g) -41.79 46.60 88.39 O2

Otavite -0.75 -12.74 -11.99 CdCO3

Pb(OH)2 -3.10 5.59 8.69 Pb(OH)2

Pb10(OH)6O(CO3)6 -59.24 -68.00 -8.76 Pb10(OH)6O(CO3)6

Pb2(OH)3Cl -9.70 -0.91 8.79 Pb2(OH)3Cl

Pb2O(OH)2 -15.00 11.19 26.19 Pb2O(OH)2

Pb2O3 -26.55 34.49 61.04 Pb2O3

Pb2OCO3 -9.29 -9.47 -0.18 Pb2OCO3

Pb3(AsO4)2 -17.25 -11.45 5.80 Pb3(AsO4)2

Pb3O2CO3 -15.92 -3.87 12.05 Pb3O2CO3

Pb3O2SO4 -13.24 -1.82 11.42 Pb3O2SO4

Pb4(OH)6SO4 -17.32 3.78 21.10 Pb4(OH)6SO4

Pb4O3SO4 -19.37 3.78 23.14 Pb4O3SO4

PbCrO4 -13.52 -26.53 -13.01 PbCrO4

Pbmetal -21.94 -17.71 4.24 Pb

PbO:0.3H2O -7.39 5.59 12.98 PbO:0.33H2O

Periclase -11.27 11.72 22.99 MgO

Phosgenite -13.85 -33.66 -19.81 PbCl2:PbCO3

Plattnerite -23.45 28.89 52.35 PbO2

Portlandite -11.69 12.31 24.00 Ca(OH)2

Smithsonite -2.95 -12.80 -9.85 ZnCO3

Tenorite -1.84 6.40 8.25 CuO

Thenardite -11.01 -10.60 0.41 Na2SO4

Thermonatrite -13.39 -12.66 0.73 Na2CO3:H2O

Zincite -4.31 7.85 12.17 ZnO

Zincosite -15.44 -10.75 4.70 ZnSO4

Zn(NO3)2:6H2O -17.62 -14.53 3.09 Zn(NO3)2:6H2O

Zn(OH)2 -4.35 7.85 12.20 Zn(OH)2

Zn(OH)2(am) -5.37 7.85 13.22 Zn(OH)2

Zn(OH)2(beta) -4.67 7.85 12.53 Zn(OH)2

Zn(OH)2(epsilon) -4.44 7.85 12.29 Zn(OH)2

Zn(OH)2(gamma) -3.88 7.85 11.73 Zn(OH)2

Zn2(OH)2SO4 -10.39 -2.89 7.50 Zn2(OH)2SO4

Zn2(OH)3Cl -11.58 3.61 15.19 Zn2(OH)3Cl

Zn3(AsO4)2:2.5H2O -18.33 -4.68 13.65 Zn3(AsO4)2:2.5H2O

Zn3O(SO4)2 -34.95 -13.64 21.31 Zn3O(SO4)2

Zn4(OH)6SO4 -15.59 12.81 28.40 Zn4(OH)6SO4

Zn5(OH)8Cl2 -23.43 15.07 38.50 Zn5(OH)8Cl2

ZnCl2 -24.07 -16.34 7.72 ZnCl2

ZnCO3:1H2O -2.54 -12.80 -10.26 ZnCO3:1H2O

Znmetal -42.66 -15.45 27.21 Zn

ZnO(active) -4.16 7.85 12.01 ZnO

ZnSO4:1H2O -10.52 -10.75 -0.23 ZnSO4:1H2O

**For a gas, SI = log10(fugacity). Fugacity = pressure * phi / 1 atm.

For ideal gases, phi = 1.

Initial solution 2.

----------------------------Distribution of species----------------------------

Log Log Log mole V

Species Molality Activity Molality Activity Gamma cm?mol

OH- 1.670e-07 1.531e-07 -6.777 -6.815 -0.038 (0)

H+ 2.173e-08 1.995e-08 -7.663 -7.700 -0.037 0.00

H2O 5.551e+01 9.999e-01 1.744 -0.000 0.000 18.02

As(3) 2.881e-18

H3AsO3 2.836e-18 2.836e-18 -17.547 -17.547 0.000 (0)

H2AsO3- 4.462e-20 4.058e-20 -19.351 -19.392 -0.041 (0)

HAsO3-2 1.368e-24 9.362e-25 -23.864 -24.029 -0.165 (0)

H4AsO3+ 3.082e-26 2.804e-26 -25.511 -25.552 -0.041 (0)

AsO3-3 2.470e-30 1.053e-30 -29.607 -29.978 -0.370 (0)

As(5) 1.782e-08

HAsO4-2 1.555e-08 1.064e-08 -7.808 -7.973 -0.165 (0)

H2AsO4- 2.270e-09 2.065e-09 -8.644 -8.685 -0.041 (0)

AsO4-3 2.752e-12 1.173e-12 -11.560 -11.931 -0.370 (0)

H3AsO4 6.143e-15 6.152e-15 -14.212 -14.211 0.001 (0)

C(4) 3.968e-03

HCO3- 3.709e-03 3.415e-03 -2.431 -2.467 -0.036 (0)

H2CO3 1.864e-04 1.864e-04 -3.730 -3.730 0.000 (0)

CaHCO3+ 4.627e-05 4.264e-05 -4.335 -4.370 -0.035 (0)

MgHCO3+ 9.299e-06 8.537e-06 -5.032 -5.069 -0.037 (0)

CO3-2 8.256e-06 5.873e-06 -5.083 -5.231 -0.148 (0)

CaCO3 6.800e-06 6.800e-06 -5.168 -5.168 0.000 (0)

MgCO3 1.006e-06 1.006e-06 -5.997 -5.997 0.000 (0)

NaHCO3 7.021e-07 7.021e-07 -6.154 -6.154 0.000 (0)

NaCO3- 5.003e-08 4.606e-08 -7.301 -7.337 -0.036 (0)

CuCO3 3.333e-08 3.333e-08 -7.477 -7.477 0.000 (0)

ZnCO3 1.227e-08 1.227e-08 -7.911 -7.911 0.000 (0)

PbCO3 4.487e-09 4.487e-09 -8.348 -8.348 0.000 (0)

ZnHCO3+ 3.155e-09 2.870e-09 -8.501 -8.542 -0.041 (0)

Cu(CO3)2-2 7.695e-10 5.268e-10 -9.114 -9.278 -0.165 (0)

PbHCO3+ 5.189e-10 4.720e-10 -9.285 -9.326 -0.041 (0)

CuHCO3+ 1.671e-10 1.520e-10 -9.777 -9.818 -0.041 (0)

Pb(CO3)2-2 1.110e-10 7.599e-11 -9.955 -10.119 -0.165 (0)

CdCO3 6.489e-11 6.489e-11 -10.188 -10.188 0.000 (0)

CdHCO3+ 3.033e-12 2.759e-12 -11.518 -11.559 -0.041 (0)

Cd(CO3)2-2 4.127e-13 2.825e-13 -12.384 -12.549 -0.165 (0)

Ca 1.566e-03

Ca+2 1.446e-03 1.028e-03 -2.840 -2.988 -0.148 (0)

CaSO4 6.550e-05 6.550e-05 -4.184 -4.184 0.000 (0)

CaHCO3+ 4.627e-05 4.264e-05 -4.335 -4.370 -0.035 (0)

CaCO3 6.800e-06 6.800e-06 -5.168 -5.168 0.000 (0)

CaNO3+ 1.340e-06 1.219e-06 -5.873 -5.914 -0.041 (0)

CaOH+ 2.854e-09 2.631e-09 -8.544 -8.580 -0.035 (0)

Cd 7.844e-10

Cd+2 6.815e-10 4.848e-10 -9.167 -9.314 -0.148 (0)

CdCO3 6.489e-11 6.489e-11 -10.188 -10.188 0.000 (0)

CdSO4 3.053e-11 3.053e-11 -10.515 -10.515 0.000 (0)

CdHCO3+ 3.033e-12 2.759e-12 -11.518 -11.559 -0.041 (0)

CdCl+ 2.235e-12 2.033e-12 -11.651 -11.692 -0.041 (0)

CdNO3+ 8.818e-13 8.021e-13 -12.055 -12.096 -0.041 (0)

CdOH+ 6.622e-13 6.023e-13 -12.179 -12.220 -0.041 (0)

Cd(CO3)2-2 4.127e-13 2.825e-13 -12.384 -12.549 -0.165 (0)

Cd(SO4)2-2 2.345e-13 1.605e-13 -12.630 -12.794 -0.165 (0)

CdOHCl 2.912e-14 2.912e-14 -13.536 -13.536 0.000 (0)

Cd(OH)2 6.186e-15 6.186e-15 -14.209 -14.209 0.000 (0)

CdCl2 3.641e-16 3.641e-16 -15.439 -15.439 0.000 (0)

Cd(NO3)2 8.572e-17 8.572e-17 -16.067 -16.067 0.000 (0)

Cd(OH)3- 2.097e-19 1.907e-19 -18.678 -18.720 -0.041 (0)

CdCl3- 9.755e-21 8.874e-21 -20.011 -20.052 -0.041 (0)

Cd2OH+3 4.161e-21 1.774e-21 -20.381 -20.751 -0.370 (0)

Cd(OH)4-2 2.301e-26 1.575e-26 -25.638 -25.803 -0.165 (0)

Cl 4.884e-05

Cl- 4.884e-05 4.485e-05 -4.311 -4.348 -0.037 (0)

ZnCl+ 3.970e-12 3.645e-12 -11.401 -11.438 -0.037 (0)

ZnOHCl 2.702e-12 2.702e-12 -11.568 -11.568 0.000 (0)

CuCl 2.300e-12 2.300e-12 -11.638 -11.638 0.000 (0)

CdCl+ 2.235e-12 2.033e-12 -11.651 -11.692 -0.041 (0)

PbCl+ 3.692e-13 3.358e-13 -12.433 -12.474 -0.041 (0)

CuCl+ 6.249e-14 5.737e-14 -13.204 -13.241 -0.037 (0)

CdOHCl 2.912e-14 2.912e-14 -13.536 -13.536 0.000 (0)

CuCl2- 2.437e-14 2.238e-14 -13.613 -13.650 -0.037 (0)

CrCl+2 9.274e-16 6.349e-16 -15.033 -15.197 -0.165 (0)

CdCl2 3.641e-16 3.641e-16 -15.439 -15.439 0.000 (0)

ZnCl2 1.319e-16 1.319e-16 -15.880 -15.880 0.000 (0)

PbCl2 6.270e-17 6.270e-17 -16.203 -16.203 0.000 (0)

CuCl2 4.144e-19 4.144e-19 -18.383 -18.383 0.000 (0)

CuCl3-2 2.820e-19 2.019e-19 -18.550 -18.695 -0.145 (0)

CrOHCl2 2.983e-20 2.983e-20 -19.525 -19.525 0.000 (0)

CdCl3- 9.755e-21 8.874e-21 -20.011 -20.052 -0.041 (0)

ZnCl3- 4.800e-21 4.407e-21 -20.319 -20.356 -0.037 (0)

PbCl3- 1.460e-21 1.328e-21 -20.836 -20.877 -0.041 (0)

CrCl2+ 7.501e-22 6.823e-22 -21.125 -21.166 -0.041 (0)

CuCl3- 1.428e-25 1.311e-25 -24.845 -24.882 -0.037 (0)

ZnCl4-2 1.218e-25 8.721e-26 -24.914 -25.059 -0.145 (0)

PbCl4-2 3.160e-26 2.164e-26 -25.500 -25.665 -0.165 (0)

CrO3Cl- 2.387e-29 2.171e-29 -28.622 -28.663 -0.041 (0)

CuCl4-2 6.983e-32 5.000e-32 -31.156 -31.301 -0.145 (0)

Cr(2) 3.639e-23

Cr+2 3.639e-23 2.491e-23 -22.439 -22.604 -0.165 (0)

Cr(3) 1.154e-06

Cr(OH)2+ 8.902e-07 8.097e-07 -6.051 -6.092 -0.041 (0)

Cr(OH)3 1.535e-07 1.535e-07 -6.814 -6.814 0.000 (0)

Cr(OH)+2 1.018e-07 6.970e-08 -6.992 -7.157 -0.165 (0)

CrO2- 4.017e-09 3.654e-09 -8.396 -8.437 -0.041 (0)

Cr(OH)4- 3.390e-09 3.083e-09 -8.470 -8.511 -0.041 (0)

CrOHSO4 1.013e-09 1.013e-09 -8.995 -8.995 0.000 (0)

Cr+3 4.472e-11 1.906e-11 -10.350 -10.720 -0.370 (0)

CrSO4+ 8.164e-12 7.426e-12 -11.088 -11.129 -0.041 (0)

Cr2(OH)2SO4+2 1.763e-15 1.207e-15 -14.754 -14.918 -0.165 (0)

CrCl+2 9.274e-16 6.349e-16 -15.033 -15.197 -0.165 (0)

CrNO3+2 1.032e-16 7.062e-17 -15.987 -16.151 -0.165 (0)

Cr2(OH)2(SO4)2 2.320e-17 2.320e-17 -16.634 -16.634 0.000 (0)

CrOHCl2 2.983e-20 2.983e-20 -19.525 -19.525 0.000 (0)

CrCl2+ 7.501e-22 6.823e-22 -21.125 -21.166 -0.041 (0)

Cr(6) 8.816e-17

CrO4-2 8.399e-17 5.975e-17 -16.076 -16.224 -0.148 (0)

HCrO4- 4.063e-18 3.696e-18 -17.391 -17.432 -0.041 (0)

NaCrO4- 8.900e-20 8.096e-20 -19.051 -19.092 -0.041 (0)

KCrO4- 1.078e-20 9.801e-21 -19.968 -20.009 -0.041 (0)

H2CrO4 2.711e-26 2.711e-26 -25.567 -25.567 0.000 (0)

CrO3SO4-2 1.108e-26 7.587e-27 -25.955 -26.120 -0.165 (0)

CrO3Cl- 2.387e-29 2.171e-29 -28.622 -28.663 -0.041 (0)

Cr2O7-2 1.039e-33 7.110e-34 -32.984 -33.148 -0.165 (0)

Cu(1) 4.710e-11

Cu+ 4.478e-11 4.073e-11 -10.349 -10.390 -0.041 (0)

CuCl 2.300e-12 2.300e-12 -11.638 -11.638 0.000 (0)

CuCl2- 2.437e-14 2.238e-14 -13.613 -13.650 -0.037 (0)

CuCl3-2 2.820e-19 2.019e-19 -18.550 -18.695 -0.145 (0)

Cu(2) 3.661e-08

CuCO3 3.333e-08 3.333e-08 -7.477 -7.477 0.000 (0)

Cu+2 1.355e-09 9.637e-10 -8.868 -9.016 -0.148 (0)

CuOH+ 7.793e-10 7.154e-10 -9.108 -9.145 -0.037 (0)

Cu(CO3)2-2 7.695e-10 5.268e-10 -9.114 -9.278 -0.165 (0)

CuHCO3+ 1.671e-10 1.520e-10 -9.777 -9.818 -0.041 (0)

Cu(OH)2 1.548e-10 1.548e-10 -9.810 -9.810 0.000 (0)

CuSO4 5.932e-11 5.932e-11 -10.227 -10.227 0.000 (0)

CuNO3+ 1.222e-12 1.111e-12 -11.913 -11.954 -0.041 (0)

Cu(OH)3- 1.762e-13 1.602e-13 -12.754 -12.795 -0.041 (0)

CuCl+ 6.249e-14 5.737e-14 -13.204 -13.241 -0.037 (0)

Cu2(OH)2+2 1.688e-14 1.155e-14 -13.773 -13.937 -0.165 (0)

Cu(NO3)2 4.281e-17 4.281e-17 -16.369 -16.369 0.000 (0)

Cu(OH)4-2 9.296e-19 6.364e-19 -18.032 -18.196 -0.165 (0)

CuCl2 4.144e-19 4.144e-19 -18.383 -18.383 0.000 (0)

CuCl3- 1.428e-25 1.311e-25 -24.845 -24.882 -0.037 (0)

CuCl4-2 6.983e-32 5.000e-32 -31.156 -31.301 -0.145 (0)

H(0) 6.586e-27

H2 3.293e-27 3.298e-27 -26.482 -26.482 0.001 (0)

K 4.818e-05

K+ 4.808e-05 4.415e-05 -4.318 -4.355 -0.037 (0)

KSO4- 1.007e-07 9.266e-08 -6.997 -7.033 -0.036 (0)

KCrO4- 1.078e-20 9.801e-21 -19.968 -20.009 -0.041 (0)

Mg 3.983e-04

Mg+2 3.741e-04 2.661e-04 -3.427 -3.575 -0.148 (0)

MgSO4 1.384e-05 1.384e-05 -4.859 -4.859 0.000 (0)

MgHCO3+ 9.299e-06 8.537e-06 -5.032 -5.069 -0.037 (0)

MgCO3 1.006e-06 1.006e-06 -5.997 -5.997 0.000 (0)

MgOH+ 1.361e-08 1.255e-08 -7.866 -7.901 -0.035 (0)

N(5) 3.651e-04

NO3- 3.637e-04 3.340e-04 -3.439 -3.476 -0.037 (0)

CaNO3+ 1.340e-06 1.219e-06 -5.873 -5.914 -0.041 (0)

ZnNO3+ 3.695e-11 3.361e-11 -10.432 -10.474 -0.041 (0)

PbNO3+ 1.323e-12 1.203e-12 -11.879 -11.920 -0.041 (0)

CuNO3+ 1.222e-12 1.111e-12 -11.913 -11.954 -0.041 (0)

CdNO3+ 8.818e-13 8.021e-13 -12.055 -12.096 -0.041 (0)

Zn(NO3)2 2.030e-15 2.030e-15 -14.692 -14.692 0.000 (0)

Pb(NO3)2 8.202e-16 8.202e-16 -15.086 -15.086 0.000 (0)

CrNO3+2 1.032e-16 7.062e-17 -15.987 -16.151 -0.165 (0)

Cd(NO3)2 8.572e-17 8.572e-17 -16.067 -16.067 0.000 (0)

Cu(NO3)2 4.281e-17 4.281e-17 -16.369 -16.369 0.000 (0)

Na 2.982e-04

Na+ 2.969e-04 2.727e-04 -3.527 -3.564 -0.037 (0)

NaHCO3 7.021e-07 7.021e-07 -6.154 -6.154 0.000 (0)

NaSO4- 5.038e-07 4.638e-07 -6.298 -6.334 -0.036 (0)

NaCO3- 5.003e-08 4.606e-08 -7.301 -7.337 -0.036 (0)

NaCrO4- 8.900e-20 8.096e-20 -19.051 -19.092 -0.041 (0)

O(0) 0.000e+00

O2 0.000e+00 0.000e+00 -43.660 -43.659 0.001 (0)

Pb 5.875e-09

PbCO3 4.487e-09 4.487e-09 -8.348 -8.348 0.000 (0)

PbHCO3+ 5.189e-10 4.720e-10 -9.285 -9.326 -0.041 (0)

Pb+2 3.573e-10 2.542e-10 -9.447 -9.595 -0.148 (0)

PbOH+ 3.541e-10 3.221e-10 -9.451 -9.492 -0.041 (0)

Pb(CO3)2-2 1.110e-10 7.599e-11 -9.955 -10.119 -0.165 (0)

PbSO4 4.028e-11 4.028e-11 -10.395 -10.395 0.000 (0)

Pb(OH)2 5.140e-12 5.140e-12 -11.289 -11.289 0.000 (0)

PbNO3+ 1.323e-12 1.203e-12 -11.879 -11.920 -0.041 (0)

PbCl+ 3.692e-13 3.358e-13 -12.433 -12.474 -0.041 (0)

Pb(SO4)2-2 1.147e-13 7.854e-14 -12.940 -13.105 -0.165 (0)

Pb(OH)3- 2.852e-15 2.594e-15 -14.545 -14.586 -0.041 (0)

Pb(NO3)2 8.202e-16 8.202e-16 -15.086 -15.086 0.000 (0)

PbCl2 6.270e-17 6.270e-17 -16.203 -16.203 0.000 (0)

Pb2OH+3 3.044e-18 1.298e-18 -17.517 -17.887 -0.370 (0)

Pb(OH)4-2 4.682e-19 3.205e-19 -18.330 -18.494 -0.165 (0)

PbCl3- 1.460e-21 1.328e-21 -20.836 -20.877 -0.041 (0)

Pb3(OH)4+2 1.668e-23 1.142e-23 -22.778 -22.942 -0.165 (0)

PbCl4-2 3.160e-26 2.164e-26 -25.500 -25.665 -0.165 (0)

Pb4(OH)4+4 1.869e-28 4.104e-29 -27.728 -28.387 -0.658 (0)

S(6) 5.348e-04

SO4-2 4.549e-04 3.236e-04 -3.342 -3.490 -0.148 (0)

CaSO4 6.550e-05 6.550e-05 -4.184 -4.184 0.000 (0)

MgSO4 1.384e-05 1.384e-05 -4.859 -4.859 0.000 (0)

NaSO4- 5.038e-07 4.638e-07 -6.298 -6.334 -0.036 (0)

KSO4- 1.007e-07 9.266e-08 -6.997 -7.033 -0.036 (0)

ZnSO4 2.251e-09 2.251e-09 -8.648 -8.648 0.000 (0)

CrOHSO4 1.013e-09 1.013e-09 -8.995 -8.995 0.000 (0)

HSO4- 4.290e-10 3.943e-10 -9.367 -9.404 -0.037 (0)

CuSO4 5.932e-11 5.932e-11 -10.227 -10.227 0.000 (0)

PbSO4 4.028e-11 4.028e-11 -10.395 -10.395 0.000 (0)

CdSO4 3.053e-11 3.053e-11 -10.515 -10.515 0.000 (0)

Zn(SO4)2-2 1.058e-11 7.244e-12 -10.975 -11.140 -0.165 (0)

CrSO4+ 8.164e-12 7.426e-12 -11.088 -11.129 -0.041 (0)

Cd(SO4)2-2 2.345e-13 1.605e-13 -12.630 -12.794 -0.165 (0)

Pb(SO4)2-2 1.147e-13 7.854e-14 -12.940 -13.105 -0.165 (0)

Cr2(OH)2SO4+2 1.763e-15 1.207e-15 -14.754 -14.918 -0.165 (0)

Cr2(OH)2(SO4)2 2.320e-17 2.320e-17 -16.634 -16.634 0.000 (0)

CrO3SO4-2 1.108e-26 7.587e-27 -25.955 -26.120 -0.165 (0)

Zn 6.953e-08

Zn+2 5.104e-08 3.631e-08 -7.292 -7.440 -0.148 (0)

ZnCO3 1.227e-08 1.227e-08 -7.911 -7.911 0.000 (0)

ZnHCO3+ 3.155e-09 2.870e-09 -8.501 -8.542 -0.041 (0)

ZnSO4 2.251e-09 2.251e-09 -8.648 -8.648 0.000 (0)

ZnOH+ 6.111e-10 5.559e-10 -9.214 -9.255 -0.041 (0)

Zn(OH)2 1.465e-10 1.465e-10 -9.834 -9.834 0.000 (0)

ZnNO3+ 3.695e-11 3.361e-11 -10.432 -10.474 -0.041 (0)

Zn(SO4)2-2 1.058e-11 7.244e-12 -10.975 -11.140 -0.165 (0)

ZnCl+ 3.970e-12 3.645e-12 -11.401 -11.438 -0.037 (0)

ZnOHCl 2.702e-12 2.702e-12 -11.568 -11.568 0.000 (0)

Zn(OH)3- 4.074e-13 3.705e-13 -12.390 -12.431 -0.041 (0)

Zn(NO3)2 2.030e-15 2.030e-15 -14.692 -14.692 0.000 (0)

ZnCl2 1.319e-16 1.319e-16 -15.880 -15.880 0.000 (0)

Zn(OH)4-2 1.087e-17 7.443e-18 -16.964 -17.128 -0.165 (0)

ZnCl3- 4.800e-21 4.407e-21 -20.319 -20.356 -0.037 (0)

ZnCl4-2 1.218e-25 8.721e-26 -24.914 -25.059 -0.145 (0)

------------------------------Saturation indices-------------------------------

Phase SI** log IAP log K(283 K, 1 atm)

Anglesite -5.18 -13.08 -7.90 PbSO4

Anhydrite -2.18 -6.48 -4.29 CaSO4

Antlerite -8.53 0.26 8.79 Cu3(OH)4SO4

Aragonite -0.03 -8.22 -8.19 CaCO3

Arsenolite -66.87 -70.19 -3.32 As4O6

Artinite -7.70 3.02 10.72 MgCO3:Mg(OH)2:3H2O

As2O5 -35.34 -28.42 6.92 As2O5

Atacamite -7.54 0.72 8.26 Cu2(OH)3Cl

Azurite -6.09 -22.11 -16.02 Cu3(OH)2(CO3)2

Bianchite -9.17 -10.93 -1.76 ZnSO4:6H2O

Brochantite -10.46 6.65 17.10 Cu4(OH)6SO4

Brucite -6.08 11.82 17.90 Mg(OH)2

Ca3(AsO4)2:4H2O -13.49 8.81 22.30 Ca3(AsO4)2:4H2O

CaCrO4 -17.20 -19.21 -2.02 CaCrO4

Calcite 0.19 -8.22 -8.41 CaCO3

Cd(OH)2 -8.44 6.09 14.52 Cd(OH)2

Cd(OH)2(am) -8.45 6.09 14.54 Cd(OH)2

Cd3(OH)2(SO4)2 -26.23 -19.52 6.71 Cd3(OH)2(SO4)2

Cd3(OH)4SO4 -23.19 -0.63 22.56 Cd3(OH)4SO4

Cd4(OH)6SO4 -22.95 5.45 28.40 Cd4(OH)6SO4

CdCl2 -17.52 -18.01 -0.49 CdCl2

CdCl2:1H2O -16.39 -18.01 -1.62 CdCl2:1H2O

CdCl2:2.5H2O -16.03 -18.01 -1.98 CdCl2:2.5H2O

Cdmetal(alpha) -31.53 -17.31 14.21 Cd

Cdmetal(gamma) -31.64 -17.31 14.32 Cd

CdOHCl -9.79 -5.96 3.82 CdOHCl

CdSO4 -13.11 -12.80 0.31 CdSO4

CdSO4:1H2O -11.37 -12.80 -1.43 CdSO4:1H2O

CdSO4:2.67H2O -11.10 -12.80 -1.71 CdSO4:2.67H2O

Cerussite -1.47 -14.83 -13.36 PbCO3

CH4(g) -70.80 -114.23 -43.43 CH4

Chalcanthite -9.81 -12.51 -2.70 CuSO4:5H2O

Claudetite -66.61 -70.19 -3.58 As4O6

CO2(g) -2.45 -20.63 -18.18 CO2

Cotunnite -13.27 -18.29 -5.02 PbCl2

Cr(OH)2 -18.35 -7.20 11.15 Cr(OH)2

Cr(OH)3 -0.00 1.61 1.61 Cr(OH)3

Cr(OH)3(am) 2.36 1.61 -0.75 Cr(OH)3

Cr2O3 5.10 3.22 -1.89 Cr2O3

CrCl2 -46.42 -31.30 15.12 CrCl2

CrCl3 -50.77 -34.54 16.24 CrCl3

Crmetal -62.68 -30.60 32.08 Cr

CrO3 -28.46 -31.62 -3.16 CrO3

Cu(OH)2 -2.81 6.38 9.20 Cu(OH)2

Cu2(OH)3NO3 -8.33 1.59 9.92 Cu2(OH)3NO3

Cu2SO4 -22.50 -24.27 -1.77 Cu2SO4

Cu3(AsO4)2:2H2O -15.37 -9.27 6.10 Cu3(AsO4)2:2H2O

CuCO3 -2.75 -14.25 -11.50 CuCO3

CuCrO4 -19.80 -25.24 -5.44 CuCrO4

Cumetal -4.97 -14.39 -9.42 Cu

CuOCuSO4 -17.70 -6.12 11.58 CuOCuSO4

Cuprite -5.13 -5.38 -0.26 Cu2O

CuSO4 -16.12 -12.51 3.62 CuSO4

Dolomite(disordered) -0.92 -17.03 -16.11 CaMg(CO3)2

Dolomite(ordered) -0.30 -17.03 -16.72 CaMg(CO3)2

Epsomite -4.83 -7.07 -2.23 MgSO4:7H2O

Goslarite -8.79 -10.93 -2.14 ZnSO4:7H2O

Gypsum -1.86 -6.48 -4.62 CaSO4:2H2O

Halite -9.48 -7.91 1.57 NaCl

Huntite -5.67 -34.64 -28.97 CaMg3(CO3)4

Hydrocerussite -5.08 -23.85 -18.77 Pb3(OH)2(CO3)2

Hydromagnesite -16.66 -23.40 -6.74 Mg5(CO3)4(OH)2:4H2O

K2Cr2O7 -38.57 -56.56 -17.99 K2Cr2O7

K2CrO4 -24.25 -24.93 -0.68 K2CrO4

Langite -12.38 6.65 19.03 Cu4(OH)6SO4:H2O

Larnakite -7.05 -7.28 -0.23 PbO:PbSO4

Laurionite -6.87 -6.24 0.62 PbOHCl

Lime -22.09 12.41 34.50 CaO

Litharge -7.50 5.81 13.30 PbO

Magnesite -1.16 -8.81 -7.65 MgCO3

Malachite -1.85 -7.86 -6.01 Cu2(OH)2CO3

Massicot -7.71 5.81 13.51 PbO

Melanothallite -24.56 -17.71 6.85 CuCl2

Mg(OH)2(active) -6.97 11.82 18.79 Mg(OH)2

MgCr2O4 -2.82 15.04 17.87 MgCr2O4

MgCrO4 -26.00 -19.80 6.21 MgCrO4

Minium -36.62 40.82 77.44 Pb3O4

Mirabilite -8.77 -10.62 -1.85 Na2SO4:10H2O

Monteponite -9.98 6.09 16.06 CdO

Na2Cr2O7 -44.87 -54.98 -10.10 Na2Cr2O7

Na2CrO4 -26.46 -23.35 3.11 Na2CrO4

Nantokite -7.61 -14.74 -7.13 CuCl

Natron -10.44 -12.36 -1.92 Na2CO3:10H2O

Nesquehonite -4.36 -8.81 -4.45 MgCO3:3H2O

O2(g) -41.59 46.80 88.39 O2

Otavite -2.55 -14.55 -11.99 CdCO3

Pb(OH)2 -2.89 5.80 8.69 Pb(OH)2

Pb10(OH)6O(CO3)6 -56.98 -65.74 -8.76 Pb10(OH)6O(CO3)6

Pb2(OH)3Cl -9.23 -0.44 8.79 Pb2(OH)3Cl

Pb2O(OH)2 -14.58 11.61 26.19 Pb2O(OH)2

Pb2O3 -26.03 35.01 61.04 Pb2O3

Pb2OCO3 -8.84 -9.02 -0.18 Pb2OCO3

Pb3(AsO4)2 -16.81 -11.01 5.80 Pb3(AsO4)2

Pb3O2CO3 -15.26 -3.22 12.05 Pb3O2CO3

Pb3O2SO4 -12.90 -1.47 11.42 Pb3O2SO4

Pb4(OH)6SO4 -16.77 4.33 21.10 Pb4(OH)6SO4

Pb4O3SO4 -18.81 4.33 23.14 Pb4O3SO4

PbCrO4 -12.81 -25.82 -13.01 PbCrO4

Pbmetal -21.83 -17.59 4.24 Pb

PbO:0.3H2O -7.17 5.81 12.98 PbO:0.33H2O

Periclase -11.16 11.83 22.99 MgO

Phosgenite -13.31 -33.12 -19.81 PbCl2:PbCO3

Plattnerite -23.14 29.20 52.35 PbO2

Portlandite -11.59 12.41 24.00 Ca(OH)2

Smithsonite -2.82 -12.67 -9.85 ZnCO3

Tenorite -1.86 6.38 8.25 CuO

Thenardite -11.03 -10.62 0.41 Na2SO4

Thermonatrite -13.09 -12.36 0.73 Na2CO3:H2O

Zincite -4.21 7.96 12.17 ZnO

Zincosite -15.63 -10.93 4.70 ZnSO4

Zn(NO3)2:6H2O -17.48 -14.39 3.09 Zn(NO3)2:6H2O

Zn(OH)2 -4.24 7.96 12.20 Zn(OH)2

Zn(OH)2(am) -5.26 7.96 13.22 Zn(OH)2

Zn(OH)2(beta) -4.57 7.96 12.53 Zn(OH)2

Zn(OH)2(epsilon) -4.33 7.96 12.29 Zn(OH)2

Zn(OH)2(gamma) -3.77 7.96 11.73 Zn(OH)2

Zn2(OH)2SO4 -10.47 -2.97 7.50 Zn2(OH)2SO4

Zn2(OH)3Cl -11.32 3.87 15.19 Zn2(OH)3Cl

Zn3(AsO4)2:2.5H2O -18.19 -4.54 13.65 Zn3(AsO4)2:2.5H2O

Zn3O(SO4)2 -35.21 -13.90 21.31 Zn3O(SO4)2

Zn4(OH)6SO4 -15.45 12.95 28.40 Zn4(OH)6SO4

Zn5(OH)8Cl2 -22.80 15.70 38.50 Zn5(OH)8Cl2

ZnCl2 -23.86 -16.14 7.72 ZnCl2

ZnCO3:1H2O -2.41 -12.67 -10.26 ZnCO3:1H2O

Znmetal -42.65 -15.44 27.21 Zn

ZnO(active) -4.05 7.96 12.01 ZnO

ZnSO4:1H2O -10.70 -10.93 -0.23 ZnSO4:1H2O

**For a gas, SI = log10(fugacity). Fugacity = pressure * phi / 1 atm.

For ideal gases, phi = 1.

Initial solution 3.

----------------------------Distribution of species----------------------------

Log Log Log mole V

Species Molality Activity Molality Activity Gamma cm?mol

OH- 1.849e-07 1.758e-07 -6.733 -6.755 -0.022 (0)

H+ 1.827e-08 1.738e-08 -7.738 -7.760 -0.022 0.00

H2O 5.551e+01 1.000e+00 1.744 -0.000 0.000 18.02

As(3) 9.979e-19

H3AsO3 9.809e-19 9.809e-19 -18.008 -18.008 0.000 (0)

H2AsO3- 1.699e-20 1.612e-20 -19.770 -19.793 -0.023 (0)

HAsO3-2 5.276e-25 4.268e-25 -24.278 -24.370 -0.092 (0)

H4AsO3+ 8.905e-27 8.445e-27 -26.050 -26.073 -0.023 (0)

AsO3-3 8.881e-31 5.512e-31 -30.052 -30.259 -0.207 (0)

As(5) 9.049e-09

HAsO4-2 7.908e-09 6.397e-09 -8.102 -8.194 -0.092 (0)

H2AsO4- 1.140e-09 1.081e-09 -8.943 -8.966 -0.023 (0)

AsO4-3 1.304e-12 8.095e-13 -11.885 -12.092 -0.207 (0)

H3AsO4 2.804e-15 2.805e-15 -14.552 -14.552 0.000 (0)

C(4) 1.633e-03

HCO3- 1.551e-03 1.477e-03 -2.809 -2.831 -0.021 (0)

H2CO3 7.019e-05 7.019e-05 -4.154 -4.154 0.000 (0)

CaHCO3+ 6.029e-06 5.743e-06 -5.220 -5.241 -0.021 (0)

CO3-2 3.560e-06 2.916e-06 -5.448 -5.535 -0.087 (0)

MgHCO3+ 1.537e-06 1.462e-06 -5.813 -5.835 -0.022 (0)

CaCO3 1.051e-06 1.051e-06 -5.978 -5.978 0.000 (0)

MgCO3 1.978e-07 1.978e-07 -6.704 -6.704 0.000 (0)

NaHCO3 2.915e-08 2.915e-08 -7.535 -7.535 0.000 (0)

CuCO3 1.802e-08 1.802e-08 -7.744 -7.744 0.000 (0)

ZnCO3 8.430e-09 8.430e-09 -8.074 -8.074 0.000 (0)

CdCO3 2.496e-09 2.496e-09 -8.603 -8.603 0.000 (0)

NaCO3- 2.306e-09 2.196e-09 -8.637 -8.658 -0.021 (0)

ZnHCO3+ 1.811e-09 1.717e-09 -8.742 -8.765 -0.023 (0)

PbCO3 1.681e-09 1.681e-09 -8.774 -8.774 0.000 (0)

Cu(CO3)2-2 1.748e-10 1.414e-10 -9.757 -9.850 -0.092 (0)

PbHCO3+ 1.624e-10 1.540e-10 -9.789 -9.812 -0.023 (0)

CdHCO3+ 9.745e-11 9.242e-11 -10.011 -10.034 -0.023 (0)

CuHCO3+ 7.546e-11 7.156e-11 -10.122 -10.145 -0.023 (0)

Pb(CO3)2-2 1.747e-11 1.413e-11 -10.758 -10.850 -0.092 (0)

Cd(CO3)2-2 6.670e-12 5.395e-12 -11.176 -11.268 -0.092 (0)

Ca 4.033e-04

Ca+2 3.910e-04 3.202e-04 -3.408 -3.495 -0.087 (0)

CaHCO3+ 6.029e-06 5.743e-06 -5.220 -5.241 -0.021 (0)

CaSO4 5.073e-06 5.073e-06 -5.295 -5.295 0.000 (0)

CaCO3 1.051e-06 1.051e-06 -5.978 -5.978 0.000 (0)

CaNO3+ 1.890e-07 1.792e-07 -6.724 -6.747 -0.023 (0)

CaOH+ 9.875e-10 9.407e-10 -9.005 -9.027 -0.021 (0)

Cd 4.918e-08

Cd+2 4.585e-08 3.755e-08 -7.339 -7.425 -0.087 (0)

CdCO3 2.496e-09 2.496e-09 -8.603 -8.603 0.000 (0)

CdSO4 5.883e-10 5.883e-10 -9.230 -9.230 0.000 (0)

CdHCO3+ 9.745e-11 9.242e-11 -10.011 -10.034 -0.023 (0)

CdOH+ 5.649e-11 5.358e-11 -10.248 -10.271 -0.023 (0)

CdCl+ 4.727e-11 4.483e-11 -10.325 -10.348 -0.023 (0)

CdNO3+ 3.093e-11 2.933e-11 -10.510 -10.533 -0.023 (0)

Cd(CO3)2-2 6.670e-12 5.395e-12 -11.176 -11.268 -0.092 (0)

Cd(SO4)2-2 9.509e-13 7.692e-13 -12.022 -12.114 -0.092 (0)

CdOHCl 7.374e-13 7.374e-13 -12.132 -12.132 0.000 (0)

Cd(OH)2 6.319e-13 6.319e-13 -12.199 -12.199 0.000 (0)

CdCl2 2.287e-15 2.287e-15 -14.641 -14.641 0.000 (0)

Cd(NO3)2 1.480e-15 1.480e-15 -14.830 -14.830 0.000 (0)

Cd(OH)3- 2.358e-17 2.237e-17 -16.627 -16.650 -0.023 (0)

Cd2OH+3 1.969e-17 1.222e-17 -16.706 -16.913 -0.207 (0)

CdCl3- 1.673e-20 1.586e-20 -19.777 -19.800 -0.023 (0)

Cd(OH)4-2 2.622e-24 2.121e-24 -23.581 -23.673 -0.092 (0)

Cl 1.342e-05

Cl- 1.342e-05 1.277e-05 -4.872 -4.894 -0.022 (0)

CdCl+ 4.727e-11 4.483e-11 -10.325 -10.348 -0.023 (0)

ZnCl+ 1.509e-12 1.436e-12 -11.821 -11.843 -0.022 (0)

ZnOHCl 1.222e-12 1.222e-12 -11.913 -11.913 0.000 (0)

CdOHCl 7.374e-13 7.374e-13 -12.132 -12.132 0.000 (0)

CuCl 7.130e-13 7.130e-13 -12.147 -12.147 0.000 (0)

PbCl+ 7.606e-14 7.214e-14 -13.119 -13.142 -0.023 (0)

CuCl+ 1.870e-14 1.779e-14 -13.728 -13.750 -0.022 (0)

CdCl2 2.287e-15 2.287e-15 -14.641 -14.641 0.000 (0)

CuCl2- 2.076e-15 1.975e-15 -14.683 -14.704 -0.022 (0)

CrCl+2 8.772e-17 7.096e-17 -16.057 -16.149 -0.092 (0)

ZnCl2 1.479e-17 1.479e-17 -16.830 -16.830 0.000 (0)

PbCl2 3.835e-18 3.835e-18 -17.416 -17.416 0.000 (0)

CuCl2 3.658e-20 3.658e-20 -19.437 -19.437 0.000 (0)

CdCl3- 1.673e-20 1.586e-20 -19.777 -19.800 -0.023 (0)

CuCl3-2 6.179e-21 5.074e-21 -20.209 -20.295 -0.086 (0)

CrOHCl2 1.090e-21 1.090e-21 -20.963 -20.963 0.000 (0)

ZnCl3- 1.479e-22 1.407e-22 -21.830 -21.852 -0.022 (0)

PbCl3- 2.439e-23 2.313e-23 -22.613 -22.636 -0.023 (0)

CrCl2+ 2.290e-23 2.171e-23 -22.640 -22.663 -0.023 (0)

CuCl3- 3.464e-27 3.295e-27 -26.460 -26.482 -0.022 (0)

ZnCl4-2 9.656e-28 7.928e-28 -27.015 -27.101 -0.086 (0)

PbCl4-2 1.326e-28 1.072e-28 -27.878 -27.970 -0.092 (0)

CrO3Cl- 5.864e-30 5.561e-30 -29.232 -29.255 -0.023 (0)

CuCl4-2 4.357e-34 3.577e-34 -33.361 -33.446 -0.086 (0)

Cr(2) 1.209e-23

Cr+2 1.209e-23 9.780e-24 -22.918 -23.010 -0.092 (0)

Cr(3) 5.770e-07

Cr(OH)2+ 4.419e-07 4.191e-07 -6.355 -6.378 -0.023 (0)

Cr(OH)3 9.123e-08 9.123e-08 -7.040 -7.040 0.000 (0)

Cr(OH)+2 3.884e-08 3.142e-08 -7.411 -7.503 -0.092 (0)

CrO2- 2.629e-09 2.493e-09 -8.580 -8.603 -0.023 (0)

Cr(OH)4- 2.219e-09 2.104e-09 -8.654 -8.677 -0.023 (0)

CrOHSO4 1.135e-10 1.135e-10 -9.945 -9.945 0.000 (0)

Cr+3 1.206e-11 7.484e-12 -10.919 -11.126 -0.207 (0)

CrSO4+ 7.646e-13 7.251e-13 -12.117 -12.140 -0.023 (0)

CrCl+2 8.772e-17 7.096e-17 -16.057 -16.149 -0.092 (0)

Cr2(OH)2SO4+2 7.542e-17 6.101e-17 -16.123 -16.215 -0.092 (0)

CrNO3+2 1.618e-17 1.309e-17 -16.791 -16.883 -0.092 (0)

Cr2(OH)2(SO4)2 2.917e-19 2.917e-19 -18.535 -18.535 0.000 (0)

CrOHCl2 1.090e-21 1.090e-21 -20.963 -20.963 0.000 (0)

CrCl2+ 2.290e-23 2.171e-23 -22.640 -22.663 -0.023 (0)

Cr(6) 9.056e-17

CrO4-2 8.652e-17 7.086e-17 -16.063 -16.150 -0.087 (0)

HCrO4- 4.026e-18 3.818e-18 -17.395 -17.418 -0.023 (0)

NaCrO4- 9.719e-21 9.217e-21 -20.012 -20.035 -0.023 (0)

KCrO4- 5.853e-21 5.551e-21 -20.233 -20.256 -0.023 (0)

H2CrO4 2.439e-26 2.439e-26 -25.613 -25.613 0.000 (0)

CrO3SO4-2 2.098e-27 1.698e-27 -26.678 -26.770 -0.092 (0)

CrO3Cl- 5.864e-30 5.561e-30 -29.232 -29.255 -0.023 (0)

Cr2O7-2 9.377e-34 7.586e-34 -33.028 -33.120 -0.092 (0)

Cu(1) 4.748e-11

Cu+ 4.676e-11 4.435e-11 -10.330 -10.353 -0.023 (0)

CuCl 7.130e-13 7.130e-13 -12.147 -12.147 0.000 (0)

CuCl2- 2.076e-15 1.975e-15 -14.683 -14.704 -0.022 (0)

CuCl3-2 6.179e-21 5.074e-21 -20.209 -20.295 -0.086 (0)

Cu(2) 2.073e-08

CuCO3 1.802e-08 1.802e-08 -7.744 -7.744 0.000 (0)

Cu+2 1.281e-09 1.049e-09 -8.892 -8.979 -0.087 (0)

CuOH+ 9.403e-10 8.945e-10 -9.027 -9.048 -0.022 (0)

Cu(OH)2 2.223e-10 2.223e-10 -9.653 -9.653 0.000 (0)

Cu(CO3)2-2 1.748e-10 1.414e-10 -9.757 -9.850 -0.092 (0)

CuHCO3+ 7.546e-11 7.156e-11 -10.122 -10.145 -0.023 (0)

CuSO4 1.606e-11 1.606e-11 -10.794 -10.794 0.000 (0)

CuNO3+ 6.023e-13 5.712e-13 -12.220 -12.243 -0.023 (0)

Cu(OH)3- 2.785e-13 2.642e-13 -12.555 -12.578 -0.023 (0)

Cu2(OH)2+2 2.232e-14 1.806e-14 -13.651 -13.743 -0.092 (0)

CuCl+ 1.870e-14 1.779e-14 -13.728 -13.750 -0.022 (0)

Cu(NO3)2 1.039e-17 1.039e-17 -16.983 -16.983 0.000 (0)

Cu(OH)4-2 1.489e-18 1.205e-18 -17.827 -17.919 -0.092 (0)

CuCl2 3.658e-20 3.658e-20 -19.437 -19.437 0.000 (0)

CuCl3- 3.464e-27 3.295e-27 -26.460 -26.482 -0.022 (0)

CuCl4-2 4.357e-34 3.577e-34 -33.361 -33.446 -0.086 (0)

H(0) 5.002e-27

H2 2.501e-27 2.502e-27 -26.602 -26.602 0.000 (0)

K 2.218e-05

K+ 2.217e-05 2.109e-05 -4.654 -4.676 -0.022 (0)

KSO4- 1.156e-08 1.101e-08 -7.937 -7.958 -0.021 (0)

KCrO4- 5.853e-21 5.551e-21 -20.233 -20.256 -0.023 (0)

Mg 1.318e-04

Mg+2 1.287e-04 1.054e-04 -3.891 -3.977 -0.087 (0)

MgHCO3+ 1.537e-06 1.462e-06 -5.813 -5.835 -0.022 (0)

MgSO4 1.363e-06 1.363e-06 -5.865 -5.865 0.000 (0)

MgCO3 1.978e-07 1.978e-07 -6.704 -6.704 0.000 (0)

MgOH+ 5.989e-09 5.707e-09 -8.223 -8.244 -0.021 (0)

N(5) 1.659e-04

NO3- 1.658e-04 1.577e-04 -3.781 -3.802 -0.022 (0)

CaNO3+ 1.890e-07 1.792e-07 -6.724 -6.747 -0.023 (0)

CdNO3+ 3.093e-11 2.933e-11 -10.510 -10.533 -0.023 (0)

ZnNO3+ 2.315e-11 2.195e-11 -10.635 -10.658 -0.023 (0)

CuNO3+ 6.023e-13 5.712e-13 -12.220 -12.243 -0.023 (0)

PbNO3+ 4.518e-13 4.285e-13 -12.345 -12.368 -0.023 (0)

Cd(NO3)2 1.480e-15 1.480e-15 -14.830 -14.830 0.000 (0)

Zn(NO3)2 6.261e-16 6.261e-16 -15.203 -15.203 0.000 (0)

Pb(NO3)2 1.379e-16 1.379e-16 -15.860 -15.860 0.000 (0)

CrNO3+2 1.618e-17 1.309e-17 -16.791 -16.883 -0.092 (0)

Cu(NO3)2 1.039e-17 1.039e-17 -16.983 -16.983 0.000 (0)

Na 2.756e-05

Na+ 2.752e-05 2.618e-05 -4.560 -4.582 -0.022 (0)

NaHCO3 2.915e-08 2.915e-08 -7.535 -7.535 0.000 (0)

NaSO4- 1.163e-08 1.107e-08 -7.934 -7.956 -0.021 (0)

NaCO3- 2.306e-09 2.196e-09 -8.637 -8.658 -0.021 (0)

NaCrO4- 9.719e-21 9.217e-21 -20.012 -20.035 -0.023 (0)

O(0) 0.000e+00

O2 0.000e+00 0.000e+00 -43.419 -43.419 0.000 (0)

Pb 2.402e-09

PbCO3 1.681e-09 1.681e-09 -8.774 -8.774 0.000 (0)

PbOH+ 2.943e-10 2.791e-10 -9.531 -9.554 -0.023 (0)

Pb+2 2.341e-10 1.917e-10 -9.631 -9.717 -0.087 (0)

PbHCO3+ 1.624e-10 1.540e-10 -9.789 -9.812 -0.023 (0)

Pb(CO3)2-2 1.747e-11 1.413e-11 -10.758 -10.850 -0.092 (0)

PbSO4 7.558e-12 7.558e-12 -11.122 -11.122 0.000 (0)

Pb(OH)2 5.113e-12 5.113e-12 -11.291 -11.291 0.000 (0)

PbNO3+ 4.518e-13 4.285e-13 -12.345 -12.368 -0.023 (0)

PbCl+ 7.606e-14 7.214e-14 -13.119 -13.142 -0.023 (0)

Pb(SO4)2-2 4.531e-15 3.665e-15 -14.344 -14.436 -0.092 (0)

Pb(OH)3- 3.124e-15 2.963e-15 -14.505 -14.528 -0.023 (0)

Pb(NO3)2 1.379e-16 1.379e-16 -15.860 -15.860 0.000 (0)

PbCl2 3.835e-18 3.835e-18 -17.416 -17.416 0.000 (0)

Pb2OH+3 1.366e-18 8.481e-19 -17.864 -18.072 -0.207 (0)

Pb(OH)4-2 5.197e-19 4.204e-19 -18.284 -18.376 -0.092 (0)

PbCl3- 2.439e-23 2.313e-23 -22.613 -22.636 -0.023 (0)

Pb3(OH)4+2 1.054e-23 8.523e-24 -22.977 -23.069 -0.092 (0)

PbCl4-2 1.326e-28 1.072e-28 -27.878 -27.970 -0.092 (0)

Pb4(OH)4+4 5.397e-29 2.312e-29 -28.268 -28.636 -0.368 (0)

S(6) 1.047e-04

SO4-2 9.827e-05 8.048e-05 -4.008 -4.094 -0.087 (0)

CaSO4 5.073e-06 5.073e-06 -5.295 -5.295 0.000 (0)

MgSO4 1.363e-06 1.363e-06 -5.865 -5.865 0.000 (0)

NaSO4- 1.163e-08 1.107e-08 -7.934 -7.956 -0.021 (0)

KSO4- 1.156e-08 1.101e-08 -7.937 -7.958 -0.021 (0)

ZnSO4 7.748e-10 7.748e-10 -9.111 -9.111 0.000 (0)

CdSO4 5.883e-10 5.883e-10 -9.230 -9.230 0.000 (0)

CrOHSO4 1.135e-10 1.135e-10 -9.945 -9.945 0.000 (0)

HSO4- 8.976e-11 8.541e-11 -10.047 -10.068 -0.022 (0)

CuSO4 1.606e-11 1.606e-11 -10.794 -10.794 0.000 (0)

PbSO4 7.558e-12 7.558e-12 -11.122 -11.122 0.000 (0)

Cd(SO4)2-2 9.509e-13 7.692e-13 -12.022 -12.114 -0.092 (0)

Zn(SO4)2-2 7.665e-13 6.201e-13 -12.115 -12.208 -0.092 (0)

CrSO4+ 7.646e-13 7.251e-13 -12.117 -12.140 -0.023 (0)

Pb(SO4)2-2 4.531e-15 3.665e-15 -14.344 -14.436 -0.092 (0)

Cr2(OH)2SO4+2 7.542e-17 6.101e-17 -16.123 -16.215 -0.092 (0)

Cr2(OH)2(SO4)2 2.917e-19 2.917e-19 -18.535 -18.535 0.000 (0)

CrO3SO4-2 2.098e-27 1.698e-27 -26.678 -26.770 -0.092 (0)

Zn 7.358e-08

Zn+2 6.134e-08 5.024e-08 -7.212 -7.299 -0.087 (0)

ZnCO3 8.430e-09 8.430e-09 -8.074 -8.074 0.000 (0)

ZnHCO3+ 1.811e-09 1.717e-09 -8.742 -8.765 -0.023 (0)

ZnOH+ 9.313e-10 8.832e-10 -9.031 -9.054 -0.023 (0)

ZnSO4 7.748e-10 7.748e-10 -9.111 -9.111 0.000 (0)

Zn(OH)2 2.673e-10 2.673e-10 -9.573 -9.573 0.000 (0)

ZnNO3+ 2.315e-11 2.195e-11 -10.635 -10.658 -0.023 (0)

ZnCl+ 1.509e-12 1.436e-12 -11.821 -11.843 -0.022 (0)

ZnOHCl 1.222e-12 1.222e-12 -11.913 -11.913 0.000 (0)

Zn(OH)3- 8.185e-13 7.762e-13 -12.087 -12.110 -0.023 (0)

Zn(SO4)2-2 7.665e-13 6.201e-13 -12.115 -12.208 -0.092 (0)

Zn(NO3)2 6.261e-16 6.261e-16 -15.203 -15.203 0.000 (0)

Zn(OH)4-2 2.213e-17 1.790e-17 -16.655 -16.747 -0.092 (0)

ZnCl2 1.479e-17 1.479e-17 -16.830 -16.830 0.000 (0)

ZnCl3- 1.479e-22 1.407e-22 -21.830 -21.852 -0.022 (0)

ZnCl4-2 9.656e-28 7.928e-28 -27.015 -27.101 -0.086 (0)

------------------------------Saturation indices-------------------------------

Phase SI** log IAP log K(283 K, 1 atm)

Anglesite -5.91 -13.81 -7.90 PbSO4

Anhydrite -3.30 -7.59 -4.29 CaSO4

Antlerite -8.78 0.01 8.79 Cu3(OH)4SO4

Aragonite -0.84 -9.03 -8.19 CaCO3

Arsenolite -68.72 -72.03 -3.32 As4O6

Artinite -8.69 2.03 10.72 MgCO3:Mg(OH)2:3H2O

As2O5 -36.02 -29.10 6.92 As2O5

Atacamite -7.83 0.43 8.26 Cu2(OH)3Cl

Azurite -6.47 -22.49 -16.02 Cu3(OH)2(CO3)2

Bianchite -9.63 -11.39 -1.76 ZnSO4:6H2O

Brochantite -10.56 6.55 17.10 Cu4(OH)6SO4

Brucite -6.36 11.54 17.90 Mg(OH)2

Ca3(AsO4)2:4H2O -15.33 6.97 22.30 Ca3(AsO4)2:4H2O

CaCrO4 -17.63 -19.64 -2.02 CaCrO4

Calcite -0.62 -9.03 -8.41 CaCO3

Cd(OH)2 -6.43 8.09 14.52 Cd(OH)2

Cd(OH)2(am) -6.44 8.09 14.54 Cd(OH)2

Cd3(OH)2(SO4)2 -21.65 -14.94 6.71 Cd3(OH)2(SO4)2

Cd3(OH)4SO4 -17.89 4.67 22.56 Cd3(OH)4SO4

Cd4(OH)6SO4 -15.64 12.76 28.40 Cd4(OH)6SO4

CdCl2 -16.73 -17.21 -0.49 CdCl2

CdCl2:1H2O -15.59 -17.21 -1.62 CdCl2:1H2O

CdCl2:2.5H2O -15.23 -17.21 -1.98 CdCl2:2.5H2O

Cdmetal(alpha) -29.64 -15.43 14.21 Cd

Cdmetal(gamma) -29.75 -15.43 14.32 Cd

CdOHCl -8.38 -4.56 3.82 CdOHCl

CdSO4 -11.83 -11.52 0.31 CdSO4

CdSO4:1H2O -10.09 -11.52 -1.43 CdSO4:1H2O

CdSO4:2.67H2O -9.81 -11.52 -1.71 CdSO4:2.67H2O

Cerussite -1.89 -15.25 -13.36 PbCO3

CH4(g) -71.70 -115.14 -43.43 CH4

Chalcanthite -10.38 -13.07 -2.70 CuSO4:5H2O

Claudetite -68.45 -72.03 -3.58 As4O6

CO2(g) -2.87 -21.06 -18.18 CO2

Cotunnite -14.48 -19.50 -5.02 PbCl2

Cr(OH)2 -18.64 -7.49 11.15 Cr(OH)2

Cr(OH)3 -0.23 1.38 1.61 Cr(OH)3

Cr(OH)3(am) 2.13 1.38 -0.75 Cr(OH)3

Cr2O3 4.65 2.76 -1.89 Cr2O3

CrCl2 -47.92 -32.80 15.12 CrCl2

CrCl3 -52.82 -36.58 16.24 CrCl3

Crmetal -63.09 -31.01 32.08 Cr

CrO3 -28.51 -31.67 -3.16 CrO3

Cu(OH)2 -2.66 6.54 9.20 Cu(OH)2

Cu2(OH)3NO3 -8.41 1.52 9.92 Cu2(OH)3NO3

Cu2SO4 -23.03 -24.80 -1.77 Cu2SO4

Cu3(AsO4)2:2H2O -15.58 -9.48 6.10 Cu3(AsO4)2:2H2O

CuCO3 -3.01 -14.51 -11.50 CuCO3

CuCrO4 -19.69 -25.13 -5.44 CuCrO4

Cumetal -4.93 -14.35 -9.42 Cu

CuOCuSO4 -18.11 -6.53 11.58 CuOCuSO4

Cuprite -4.93 -5.19 -0.26 Cu2O

CuSO4 -16.69 -13.07 3.62 CuSO4

Dolomite(disordered) -2.43 -18.54 -16.11 CaMg(CO3)2

Dolomite(ordered) -1.82 -18.54 -16.72 CaMg(CO3)2

Epsomite -5.84 -8.07 -2.23 MgSO4:7H2O

Goslarite -9.25 -11.39 -2.14 ZnSO4:7H2O

Gypsum -2.97 -7.59 -4.62 CaSO4:2H2O

Halite -11.04 -9.48 1.57 NaCl

Huntite -8.60 -37.57 -28.97 CaMg3(CO3)4

Hydrocerussite -5.93 -24.70 -18.77 Pb3(OH)2(CO3)2

Hydromagnesite -19.77 -26.51 -6.74 Mg5(CO3)4(OH)2:4H2O

K2Cr2O7 -39.18 -57.17 -17.99 K2Cr2O7

K2CrO4 -24.82 -25.50 -0.68 K2CrO4

Langite -12.48 6.55 19.03 Cu4(OH)6SO4:H2O

Larnakite -7.78 -8.01 -0.23 PbO:PbSO4

Laurionite -7.47 -6.85 0.62 PbOHCl

Lime -22.47 12.03 34.50 CaO

Litharge -7.50 5.80 13.30 PbO

Magnesite -1.87 -9.51 -7.65 MgCO3

Malachite -1.96 -7.97 -6.01 Cu2(OH)2CO3

Massicot -7.71 5.80 13.51 PbO

Melanothallite -25.61 -18.77 6.85 CuCl2

Mg(OH)2(active) -7.25 11.54 18.79 Mg(OH)2

MgCr2O4 -3.56 14.31 17.87 MgCr2O4

MgCrO4 -26.33 -20.13 6.21 MgCrO4

Minium -36.51 40.93 77.44 Pb3O4

Mirabilite -11.41 -13.26 -1.85 Na2SO4:10H2O

Monteponite -7.97 8.09 16.06 CdO

Na2Cr2O7 -46.88 -56.98 -10.10 Na2Cr2O7

Na2CrO4 -28.43 -25.31 3.11 Na2CrO4

Nantokite -8.12 -15.25 -7.13 CuCl

Natron -12.78 -14.70 -1.92 Na2CO3:10H2O

Nesquehonite -5.07 -9.51 -4.45 MgCO3:3H2O

O2(g) -41.35 47.04 88.39 O2

Otavite -0.97 -12.96 -11.99 CdCO3

Pb(OH)2 -2.89 5.80 8.69 Pb(OH)2

Pb10(OH)6O(CO3)6 -59.54 -68.30 -8.76 Pb10(OH)6O(CO3)6

Pb2(OH)3Cl -9.84 -1.05 8.79 Pb2(OH)3Cl

Pb2O(OH)2 -14.58 11.61 26.19 Pb2O(OH)2

Pb2O3 -25.91 35.13 61.04 Pb2O3

Pb2OCO3 -9.27 -9.45 -0.18 Pb2OCO3

Pb3(AsO4)2 -17.50 -11.70 5.80 Pb3(AsO4)2

Pb3O2CO3 -15.69 -3.65 12.05 Pb3O2CO3

Pb3O2SO4 -13.63 -2.21 11.42 Pb3O2SO4

Pb4(OH)6SO4 -17.50 3.60 21.10 Pb4(OH)6SO4

Pb4O3SO4 -19.55 3.60 23.14 Pb4O3SO4

PbCrO4 -12.86 -25.87 -13.01 PbCrO4

Pbmetal -21.95 -17.72 4.24 Pb

PbO:0.3H2O -7.18 5.80 12.98 PbO:0.33H2O

Periclase -11.44 11.54 22.99 MgO

Phosgenite -14.95 -34.76 -19.81 PbCl2:PbCO3

Plattnerite -23.03 29.32 52.35 PbO2

Portlandite -11.97 12.03 24.00 Ca(OH)2

Smithsonite -2.98 -12.83 -9.85 ZnCO3

Tenorite -1.71 6.54 8.25 CuO

Thenardite -13.66 -13.26 0.41 Na2SO4

Thermonatrite -15.43 -14.70 0.73 Na2CO3:H2O

Zincite -3.94 8.22 12.17 ZnO

Zincosite -16.09 -11.39 4.70 ZnSO4

Zn(NO3)2:6H2O -17.99 -14.90 3.09 Zn(NO3)2:6H2O

Zn(OH)2 -3.98 8.22 12.20 Zn(OH)2

Zn(OH)2(am) -5.00 8.22 13.22 Zn(OH)2

Zn(OH)2(beta) -4.30 8.22 12.53 Zn(OH)2

Zn(OH)2(epsilon) -4.07 8.22 12.29 Zn(OH)2

Zn(OH)2(gamma) -3.51 8.22 11.73 Zn(OH)2

Zn2(OH)2SO4 -10.67 -3.17 7.50 Zn2(OH)2SO4

Zn2(OH)3Cl -11.40 3.79 15.19 Zn2(OH)3Cl

Zn3(AsO4)2:2.5H2O -18.09 -4.44 13.65 Zn3(AsO4)2:2.5H2O

Zn3O(SO4)2 -35.87 -14.57 21.31 Zn3O(SO4)2

Zn4(OH)6SO4 -15.13 13.27 28.40 Zn4(OH)6SO4

Zn5(OH)8Cl2 -22.70 15.80 38.50 Zn5(OH)8Cl2

ZnCl2 -24.81 -17.09 7.72 ZnCl2

ZnCO3:1H2O -2.57 -12.83 -10.26 ZnCO3:1H2O

Znmetal -42.51 -15.30 27.21 Zn

ZnO(active) -3.79 8.22 12.01 ZnO

ZnSO4:1H2O -11.16 -11.39 -0.23 ZnSO4:1H2O

**For a gas, SI = log10(fugacity). Fugacity = pressure * phi / 1 atm.

For ideal gases, phi = 1.

Initial solution 4.

----------------------------Distribution of species----------------------------

Log Log Log mole V

Species Molality Activity Molality Activity Gamma cm?mol

OH- 6.077e-07 5.689e-07 -6.216 -6.245 -0.029 (0)

H+ 5.733e-09 5.370e-09 -8.242 -8.270 -0.028 0.00

H2O 5.551e+01 9.999e-01 1.744 -0.000 0.000 18.02

As(3) 3.048e-20

H3AsO3 2.883e-20 2.883e-20 -19.540 -19.540 0.000 (0)

H2AsO3- 1.645e-21 1.533e-21 -20.784 -20.815 -0.031 (0)

HAsO3-2 1.744e-25 1.314e-25 -24.759 -24.881 -0.123 (0)

H4AsO3+ 8.234e-29 7.671e-29 -28.084 -28.115 -0.031 (0)

AsO3-3 1.038e-30 5.490e-31 -29.984 -30.260 -0.277 (0)

As(5) 2.853e-08

HAsO4-2 2.736e-08 2.062e-08 -7.563 -7.686 -0.123 (0)

H2AsO4- 1.156e-09 1.077e-09 -8.937 -8.968 -0.031 (0)

AsO4-3 1.596e-11 8.442e-12 -10.797 -11.074 -0.277 (0)

H3AsO4 8.626e-16 8.633e-16 -15.064 -15.064 0.000 (0)

C(4) 2.912e-03

HCO3- 2.816e-03 2.642e-03 -2.550 -2.578 -0.028 (0)

H2CO3 3.881e-05 3.881e-05 -4.411 -4.411 0.000 (0)

CO3-2 2.192e-05 1.688e-05 -4.659 -4.773 -0.113 (0)

CaHCO3+ 1.706e-05 1.601e-05 -4.768 -4.796 -0.027 (0)

CaCO3 9.487e-06 9.487e-06 -5.023 -5.023 0.000 (0)

MgHCO3+ 5.707e-06 5.345e-06 -5.244 -5.272 -0.028 (0)

MgCO3 2.340e-06 2.340e-06 -5.631 -5.631 0.000 (0)

NaHCO3 2.157e-07 2.157e-07 -6.666 -6.666 0.000 (0)

NaCO3- 5.604e-08 5.258e-08 -7.251 -7.279 -0.028 (0)

CuCO3 4.016e-08 4.016e-08 -7.396 -7.396 0.000 (0)

ZnCO3 2.983e-08 2.983e-08 -7.525 -7.525 0.000 (0)

PbCO3 3.655e-09 3.655e-09 -8.437 -8.437 0.000 (0)

Cu(CO3)2-2 2.422e-09 1.825e-09 -8.616 -8.739 -0.123 (0)

ZnHCO3+ 2.016e-09 1.878e-09 -8.696 -8.726 -0.031 (0)

CdCO3 3.916e-10 3.916e-10 -9.407 -9.407 0.000 (0)

Pb(CO3)2-2 2.362e-10 1.780e-10 -9.627 -9.750 -0.123 (0)

PbHCO3+ 1.111e-10 1.035e-10 -9.954 -9.985 -0.031 (0)

CuHCO3+ 5.291e-11 4.929e-11 -10.276 -10.307 -0.031 (0)

Cd(CO3)2-2 6.504e-12 4.900e-12 -11.187 -11.310 -0.123 (0)

CdHCO3+ 4.809e-12 4.480e-12 -11.318 -11.349 -0.031 (0)

Ca 6.863e-04

Ca+2 6.482e-04 4.991e-04 -3.188 -3.302 -0.113 (0)

CaHCO3+ 1.706e-05 1.601e-05 -4.768 -4.796 -0.027 (0)

CaSO4 1.124e-05 1.124e-05 -4.949 -4.949 0.000 (0)

CaCO3 9.487e-06 9.487e-06 -5.023 -5.023 0.000 (0)

CaNO3+ 3.522e-07 3.281e-07 -6.453 -6.484 -0.031 (0)

CaOH+ 5.053e-09 4.744e-09 -8.296 -8.324 -0.027 (0)

Cd 1.757e-09

Cd+2 1.322e-09 1.018e-09 -8.879 -8.992 -0.113 (0)

CdCO3 3.916e-10 3.916e-10 -9.407 -9.407 0.000 (0)

CdSO4 2.266e-11 2.266e-11 -10.645 -10.645 0.000 (0)

Cd(CO3)2-2 6.504e-12 4.900e-12 -11.187 -11.310 -0.123 (0)

CdOH+ 5.042e-12 4.698e-12 -11.297 -11.328 -0.031 (0)

CdHCO3+ 4.809e-12 4.480e-12 -11.318 -11.349 -0.031 (0)

CdCl+ 3.689e-12 3.437e-12 -11.433 -11.464 -0.031 (0)

CdNO3+ 1.002e-12 9.336e-13 -11.999 -12.030 -0.031 (0)

CdOHCl 1.830e-13 1.830e-13 -12.738 -12.738 0.000 (0)

Cd(OH)2 1.793e-13 1.793e-13 -12.746 -12.746 0.000 (0)

Cd(SO4)2-2 5.590e-14 4.212e-14 -13.253 -13.376 -0.123 (0)

CdCl2 4.961e-16 4.961e-16 -15.304 -15.304 0.000 (0)

Cd(NO3)2 5.533e-17 5.533e-17 -16.257 -16.257 0.000 (0)

Cd(OH)3- 2.204e-17 2.053e-17 -16.657 -16.688 -0.031 (0)

Cd2OH+3 5.490e-20 2.904e-20 -19.260 -19.537 -0.277 (0)

CdCl3- 1.045e-20 9.738e-21 -19.981 -20.012 -0.031 (0)

Cd(OH)4-2 8.363e-24 6.302e-24 -23.078 -23.201 -0.123 (0)

Cl 3.857e-05

Cl- 3.857e-05 3.613e-05 -4.414 -4.442 -0.028 (0)

ZnOHCl 6.841e-12 6.841e-12 -11.165 -11.165 0.000 (0)

CdCl+ 3.689e-12 3.437e-12 -11.433 -11.464 -0.031 (0)

ZnCl+ 2.651e-12 2.483e-12 -11.577 -11.605 -0.028 (0)

CuCl 7.767e-13 7.767e-13 -12.110 -12.110 0.000 (0)

CdOHCl 1.830e-13 1.830e-13 -12.738 -12.738 0.000 (0)

PbCl+ 8.230e-14 7.668e-14 -13.085 -13.115 -0.031 (0)

CuCl+ 2.069e-14 1.937e-14 -13.684 -13.713 -0.028 (0)

CuCl2- 6.500e-15 6.088e-15 -14.187 -14.216 -0.028 (0)

CdCl2 4.961e-16 4.961e-16 -15.304 -15.304 0.000 (0)

ZnCl2 7.239e-17 7.239e-17 -16.140 -16.140 0.000 (0)

CrCl+2 1.814e-17 1.367e-17 -16.741 -16.864 -0.123 (0)

PbCl2 1.153e-17 1.153e-17 -16.938 -16.938 0.000 (0)

CuCl2 1.128e-19 1.128e-19 -18.948 -18.948 0.000 (0)

CuCl3-2 5.723e-20 4.425e-20 -19.242 -19.354 -0.112 (0)

CdCl3- 1.045e-20 9.738e-21 -19.981 -20.012 -0.031 (0)

ZnCl3- 2.081e-21 1.949e-21 -20.682 -20.710 -0.028 (0)

CrOHCl2 1.922e-21 1.922e-21 -20.716 -20.716 0.000 (0)

PbCl3- 2.113e-22 1.968e-22 -21.675 -21.706 -0.031 (0)

CrCl2+ 1.270e-23 1.183e-23 -22.896 -22.927 -0.031 (0)

ZnCl4-2 4.017e-26 3.106e-26 -25.396 -25.508 -0.112 (0)

CuCl3- 3.068e-26 2.874e-26 -25.513 -25.542 -0.028 (0)

PbCl4-2 3.427e-27 2.582e-27 -26.465 -26.588 -0.123 (0)

CrO3Cl- 1.320e-27 1.230e-27 -26.880 -26.910 -0.031 (0)

CuCl4-2 1.142e-32 8.828e-33 -31.942 -32.054 -0.112 (0)

Cr(2) 8.834e-25

Cr+2 8.834e-25 6.656e-25 -24.054 -24.177 -0.123 (0)

Cr(3) 5.770e-07

Cr(OH)2+ 3.206e-07 2.987e-07 -6.494 -6.525 -0.031 (0)

Cr(OH)3 2.104e-07 2.104e-07 -6.677 -6.677 0.000 (0)

CrO2- 1.997e-08 1.860e-08 -7.700 -7.730 -0.031 (0)

Cr(OH)4- 1.685e-08 1.570e-08 -7.773 -7.804 -0.031 (0)

Cr(OH)+2 9.183e-09 6.920e-09 -8.037 -8.160 -0.123 (0)

CrOHSO4 3.554e-11 3.554e-11 -10.449 -10.449 0.000 (0)

Cr+3 9.629e-13 5.093e-13 -12.016 -12.293 -0.277 (0)

CrSO4+ 7.530e-14 7.015e-14 -13.123 -13.154 -0.031 (0)

CrCl+2 1.814e-17 1.367e-17 -16.741 -16.864 -0.123 (0)

Cr2(OH)2SO4+2 5.582e-18 4.206e-18 -17.253 -17.376 -0.123 (0)

CrNO3+2 1.389e-18 1.046e-18 -17.857 -17.980 -0.123 (0)

Cr2(OH)2(SO4)2 2.858e-20 2.858e-20 -19.544 -19.544 0.000 (0)

CrOHCl2 1.922e-21 1.922e-21 -20.716 -20.716 0.000 (0)

CrCl2+ 1.270e-23 1.183e-23 -22.896 -22.927 -0.031 (0)

Cr(6) 7.636e-14

CrO4-2 7.529e-14 5.797e-14 -13.123 -13.237 -0.113 (0)

HCrO4- 1.036e-15 9.653e-16 -14.985 -15.015 -0.031 (0)

NaCrO4- 3.348e-17 3.119e-17 -16.475 -16.506 -0.031 (0)

KCrO4- 7.293e-18 6.794e-18 -17.137 -17.168 -0.031 (0)

H2CrO4 1.906e-24 1.906e-24 -23.720 -23.720 0.000 (0)

CrO3SO4-2 2.502e-25 1.885e-25 -24.602 -24.725 -0.123 (0)

CrO3Cl- 1.320e-27 1.230e-27 -26.880 -26.910 -0.031 (0)

Cr2O7-2 6.436e-29 4.849e-29 -28.191 -28.314 -0.123 (0)

Cu(1) 1.911e-11

Cu+ 1.833e-11 1.707e-11 -10.737 -10.768 -0.031 (0)

CuCl 7.767e-13 7.767e-13 -12.110 -12.110 0.000 (0)

CuCl2- 6.500e-15 6.088e-15 -14.187 -14.216 -0.028 (0)

CuCl3-2 5.723e-20 4.425e-20 -19.242 -19.354 -0.112 (0)

Cu(2) 4.526e-08

CuCO3 4.016e-08 4.016e-08 -7.396 -7.396 0.000 (0)

Cu(CO3)2-2 2.422e-09 1.825e-09 -8.616 -8.739 -0.123 (0)

CuOH+ 1.190e-09 1.114e-09 -8.925 -8.953 -0.028 (0)

Cu(OH)2 8.960e-10 8.960e-10 -9.048 -9.048 0.000 (0)

Cu+2 5.246e-10 4.040e-10 -9.280 -9.394 -0.113 (0)

CuHCO3+ 5.291e-11 4.929e-11 -10.276 -10.307 -0.031 (0)

CuSO4 8.792e-12 8.792e-12 -11.056 -11.056 0.000 (0)

Cu(OH)3- 3.698e-12 3.446e-12 -11.432 -11.463 -0.031 (0)

CuNO3+ 2.772e-13 2.583e-13 -12.557 -12.588 -0.031 (0)

Cu2(OH)2+2 3.720e-14 2.803e-14 -13.429 -13.552 -0.123 (0)

CuCl+ 2.069e-14 1.937e-14 -13.684 -13.713 -0.028 (0)

Cu(OH)4-2 6.748e-17 5.084e-17 -16.171 -16.294 -0.123 (0)

Cu(NO3)2 5.518e-18 5.518e-18 -17.258 -17.258 0.000 (0)

CuCl2 1.128e-19 1.128e-19 -18.948 -18.948 0.000 (0)

CuCl3- 3.068e-26 2.874e-26 -25.513 -25.542 -0.028 (0)

CuCl4-2 1.142e-32 8.828e-33 -31.942 -32.054 -0.112 (0)

H(0) 4.775e-28

H2 2.387e-28 2.389e-28 -27.622 -27.622 0.000 (0)

K 3.370e-05

K+ 3.367e-05 3.154e-05 -4.473 -4.501 -0.028 (0)

KSO4- 2.495e-08 2.341e-08 -7.603 -7.631 -0.028 (0)

KCrO4- 7.293e-18 6.794e-18 -17.137 -17.168 -0.031 (0)

Mg 2.917e-04

Mg+2 2.797e-04 2.154e-04 -3.553 -3.667 -0.113 (0)

MgHCO3+ 5.707e-06 5.345e-06 -5.244 -5.272 -0.028 (0)

MgSO4 3.961e-06 3.961e-06 -5.402 -5.402 0.000 (0)

MgCO3 2.340e-06 2.340e-06 -5.631 -5.631 0.000 (0)

MgOH+ 4.018e-08 3.774e-08 -7.396 -7.423 -0.027 (0)

N(5) 1.981e-04

NO3- 1.977e-04 1.852e-04 -3.704 -3.732 -0.028 (0)

CaNO3+ 3.522e-07 3.281e-07 -6.453 -6.484 -0.031 (0)

ZnNO3+ 1.692e-11 1.576e-11 -10.772 -10.802 -0.031 (0)

CdNO3+ 1.002e-12 9.336e-13 -11.999 -12.030 -0.031 (0)

CuNO3+ 2.772e-13 2.583e-13 -12.557 -12.588 -0.031 (0)

PbNO3+ 2.029e-13 1.891e-13 -12.693 -12.723 -0.031 (0)

Zn(NO3)2 5.280e-16 5.280e-16 -15.277 -15.277 0.000 (0)

Pb(NO3)2 7.148e-17 7.148e-17 -16.146 -16.146 0.000 (0)

Cd(NO3)2 5.533e-17 5.533e-17 -16.257 -16.257 0.000 (0)

Cu(NO3)2 5.518e-18 5.518e-18 -17.258 -17.258 0.000 (0)

CrNO3+2 1.389e-18 1.046e-18 -17.857 -17.980 -0.123 (0)

Na 1.159e-04

Na+ 1.156e-04 1.083e-04 -3.937 -3.965 -0.028 (0)

NaHCO3 2.157e-07 2.157e-07 -6.666 -6.666 0.000 (0)

NaSO4- 6.940e-08 6.511e-08 -7.159 -7.186 -0.028 (0)

NaCO3- 5.604e-08 5.258e-08 -7.251 -7.279 -0.028 (0)

NaCrO4- 3.348e-17 3.119e-17 -16.475 -16.506 -0.031 (0)

O(0) 0.000e+00

O2 0.000e+00 0.000e+00 -41.379 -41.379 0.000 (0)

Pb 4.485e-09

PbCO3 3.655e-09 3.655e-09 -8.437 -8.437 0.000 (0)

PbOH+ 3.641e-10 3.392e-10 -9.439 -9.470 -0.031 (0)

Pb(CO3)2-2 2.362e-10 1.780e-10 -9.627 -9.750 -0.123 (0)

PbHCO3+ 1.111e-10 1.035e-10 -9.954 -9.985 -0.031 (0)

Pb+2 9.354e-11 7.203e-11 -10.029 -10.143 -0.113 (0)

Pb(OH)2 2.011e-11 2.011e-11 -10.697 -10.697 0.000 (0)

PbSO4 4.036e-12 4.036e-12 -11.394 -11.394 0.000 (0)

PbNO3+ 2.029e-13 1.891e-13 -12.693 -12.723 -0.031 (0)

PbCl+ 8.230e-14 7.668e-14 -13.085 -13.115 -0.031 (0)

Pb(OH)3- 4.047e-14 3.771e-14 -13.393 -13.424 -0.031 (0)

Pb(SO4)2-2 3.692e-15 2.782e-15 -14.433 -14.556 -0.123 (0)

Pb(NO3)2 7.148e-17 7.148e-17 -16.146 -16.146 0.000 (0)

Pb(OH)4-2 2.298e-17 1.731e-17 -16.639 -16.762 -0.123 (0)

PbCl2 1.153e-17 1.153e-17 -16.938 -16.938 0.000 (0)

Pb2OH+3 7.320e-19 3.872e-19 -18.135 -18.412 -0.277 (0)

PbCl3- 2.113e-22 1.968e-22 -21.675 -21.706 -0.031 (0)

Pb3(OH)4+2 6.573e-23 4.953e-23 -22.182 -22.305 -0.123 (0)

PbCl4-2 3.427e-27 2.582e-27 -26.465 -26.588 -0.123 (0)

Pb4(OH)4+4 1.565e-28 5.046e-29 -27.805 -28.297 -0.492 (0)

S(6) 1.639e-04

SO4-2 1.486e-04 1.144e-04 -3.828 -3.942 -0.113 (0)

CaSO4 1.124e-05 1.124e-05 -4.949 -4.949 0.000 (0)

MgSO4 3.961e-06 3.961e-06 -5.402 -5.402 0.000 (0)

NaSO4- 6.940e-08 6.511e-08 -7.159 -7.186 -0.028 (0)

KSO4- 2.495e-08 2.341e-08 -7.603 -7.631 -0.028 (0)

ZnSO4 6.732e-10 6.732e-10 -9.172 -9.172 0.000 (0)

HSO4- 4.004e-11 3.752e-11 -10.398 -10.426 -0.028 (0)

CrOHSO4 3.554e-11 3.554e-11 -10.449 -10.449 0.000 (0)

CdSO4 2.266e-11 2.266e-11 -10.645 -10.645 0.000 (0)

CuSO4 8.792e-12 8.792e-12 -11.056 -11.056 0.000 (0)

PbSO4 4.036e-12 4.036e-12 -11.394 -11.394 0.000 (0)

Zn(SO4)2-2 1.016e-12 7.658e-13 -11.993 -12.116 -0.123 (0)

CrSO4+ 7.530e-14 7.015e-14 -13.123 -13.154 -0.031 (0)

Cd(SO4)2-2 5.590e-14 4.212e-14 -13.253 -13.376 -0.123 (0)

Pb(SO4)2-2 3.692e-15 2.782e-15 -14.433 -14.556 -0.123 (0)

Cr2(OH)2SO4+2 5.582e-18 4.206e-18 -17.253 -17.376 -0.123 (0)

Cr2(OH)2(SO4)2 2.858e-20 2.858e-20 -19.544 -19.544 0.000 (0)

CrO3SO4-2 2.502e-25 1.885e-25 -24.602 -24.725 -0.123 (0)

Zn 7.603e-08

Zn+2 3.988e-08 3.071e-08 -7.399 -7.513 -0.113 (0)

ZnCO3 2.983e-08 2.983e-08 -7.525 -7.525 0.000 (0)

ZnHCO3+ 2.016e-09 1.878e-09 -8.696 -8.726 -0.031 (0)

ZnOH+ 1.875e-09 1.747e-09 -8.727 -8.758 -0.031 (0)

Zn(OH)2 1.711e-09 1.711e-09 -8.767 -8.767 0.000 (0)

ZnSO4 6.732e-10 6.732e-10 -9.172 -9.172 0.000 (0)

Zn(OH)3- 1.725e-11 1.607e-11 -10.763 -10.794 -0.031 (0)

ZnNO3+ 1.692e-11 1.576e-11 -10.772 -10.802 -0.031 (0)

ZnOHCl 6.841e-12 6.841e-12 -11.165 -11.165 0.000 (0)

ZnCl+ 2.651e-12 2.483e-12 -11.577 -11.605 -0.028 (0)

Zn(SO4)2-2 1.016e-12 7.658e-13 -11.993 -12.116 -0.123 (0)

Zn(OH)4-2 1.592e-15 1.200e-15 -14.798 -14.921 -0.123 (0)

Zn(NO3)2 5.280e-16 5.280e-16 -15.277 -15.277 0.000 (0)

ZnCl2 7.239e-17 7.239e-17 -16.140 -16.140 0.000 (0)

ZnCl3- 2.081e-21 1.949e-21 -20.682 -20.710 -0.028 (0)

ZnCl4-2 4.017e-26 3.106e-26 -25.396 -25.508 -0.112 (0)

------------------------------Saturation indices-------------------------------

Phase SI** log IAP log K(283 K, 1 atm)

Anglesite -6.18 -14.08 -7.90 PbSO4

Anhydrite -2.95 -7.24 -4.29 CaSO4

Antlerite -7.83 0.96 8.79 Cu3(OH)4SO4

Aragonite 0.11 -8.07 -8.19 CaCO3

Arsenolite -74.84 -78.16 -3.32 As4O6

Artinite -6.28 4.43 10.72 MgCO3:Mg(OH)2:3H2O

As2O5 -37.04 -30.13 6.92 As2O5

Atacamite -6.68 1.58 8.26 Cu2(OH)3Cl

Azurite -5.16 -21.19 -16.02 Cu3(OH)2(CO3)2

Bianchite -9.70 -11.45 -1.76 ZnSO4:6H2O

Brochantite -9.00 8.10 17.10 Cu4(OH)6SO4

Brucite -5.03 12.87 17.90 Mg(OH)2

Ca3(AsO4)2:4H2O -12.71 9.59 22.30 Ca3(AsO4)2:4H2O

CaCrO4 -14.52 -16.54 -2.02 CaCrO4

Calcite 0.33 -8.07 -8.41 CaCO3

Cd(OH)2 -6.97 7.55 14.52 Cd(OH)2

Cd(OH)2(am) -6.99 7.55 14.54 Cd(OH)2

Cd3(OH)2(SO4)2 -25.03 -18.32 6.71 Cd3(OH)2(SO4)2

Cd3(OH)4SO4 -20.40 2.16 22.56 Cd3(OH)4SO4

Cd4(OH)6SO4 -18.69 9.71 28.40 Cd4(OH)6SO4

CdCl2 -17.39 -17.88 -0.49 CdCl2

CdCl2:1H2O -16.25 -17.88 -1.62 CdCl2:1H2O

CdCl2:2.5H2O -15.90 -17.88 -1.98 CdCl2:2.5H2O

Cdmetal(alpha) -31.21 -16.99 14.21 Cd

Cdmetal(gamma) -31.32 -16.99 14.32 Cd

CdOHCl -8.99 -5.16 3.82 CdOHCl

CdSO4 -13.24 -12.93 0.31 CdSO4

CdSO4:1H2O -11.50 -12.93 -1.43 CdSO4:1H2O

CdSO4:2.67H2O -11.23 -12.93 -1.71 CdSO4:2.67H2O

Cerussite -1.56 -14.92 -13.36 PbCO3

CH4(g) -76.04 -119.47 -43.43 CH4

Chalcanthite -10.64 -13.34 -2.70 CuSO4:5H2O

Claudetite -74.58 -78.16 -3.58 As4O6

CO2(g) -3.13 -21.31 -18.18 CO2

Cotunnite -14.00 -19.03 -5.02 PbCl2

Cr(OH)2 -18.79 -7.64 11.15 Cr(OH)2

Cr(OH)3 0.13 1.75 1.61 Cr(OH)3

Cr(OH)3(am) 2.50 1.75 -0.75 Cr(OH)3

Cr2O3 5.38 3.49 -1.89 Cr2O3

CrCl2 -48.18 -33.06 15.12 CrCl2

CrCl3 -52.63 -36.39 16.24 CrCl3

Crmetal -64.26 -32.18 32.08 Cr

CrO3 -26.61 -29.78 -3.16 CrO3

Cu(OH)2 -2.05 7.15 9.20 Cu(OH)2

Cu2(OH)3NO3 -7.63 2.29 9.92 Cu2(OH)3NO3

Cu2SO4 -23.70 -25.48 -1.77 Cu2SO4

Cu3(AsO4)2:2H2O -14.79 -8.69 6.10 Cu3(AsO4)2:2H2O

CuCO3 -2.67 -14.17 -11.50 CuCO3

CuCrO4 -17.19 -22.63 -5.44 CuCrO4

Cumetal -5.35 -14.77 -9.42 Cu

CuOCuSO4 -17.77 -6.19 11.58 CuOCuSO4

Cuprite -4.74 -5.00 -0.26 Cu2O

CuSO4 -16.95 -13.34 3.62 CuSO4

Dolomite(disordered) -0.40 -16.51 -16.11 CaMg(CO3)2

Dolomite(ordered) 0.21 -16.51 -16.72 CaMg(CO3)2

Epsomite -5.37 -7.61 -2.23 MgSO4:7H2O

Goslarite -9.31 -11.45 -2.14 ZnSO4:7H2O

Gypsum -2.62 -7.24 -4.62 CaSO4:2H2O

Halite -9.98 -8.41 1.57 NaCl

Huntite -4.42 -33.39 -28.97 CaMg3(CO3)4

Hydrocerussite -4.66 -23.43 -18.77 Pb3(OH)2(CO3)2

Hydromagnesite -14.15 -20.88 -6.74 Mg5(CO3)4(OH)2:4H2O

K2Cr2O7 -34.02 -52.02 -17.99 K2Cr2O7

K2CrO4 -21.56 -22.24 -0.68 K2CrO4

Langite -10.92 8.10 19.03 Cu4(OH)6SO4:H2O

Larnakite -7.45 -7.69 -0.23 PbO:PbSO4

Laurionite -6.94 -6.31 0.62 PbOHCl

Lime -21.26 13.24 34.50 CaO

Litharge -6.90 6.40 13.30 PbO

Magnesite -0.79 -8.44 -7.65 MgCO3

Malachite -1.01 -7.02 -6.01 Cu2(OH)2CO3

Massicot -7.12 6.40 13.51 PbO

Melanothallite -25.12 -18.28 6.85 CuCl2

Mg(OH)2(active) -5.92 12.87 18.79 Mg(OH)2

MgCr2O4 -1.50 16.36 17.87 MgCr2O4

MgCrO4 -23.11 -16.90 6.21 MgCrO4

Minium -33.70 43.73 77.44 Pb3O4

Mirabilite -10.02 -11.87 -1.85 Na2SO4:10H2O

Monteponite -8.52 7.55 16.06 CdO

Na2Cr2O7 -40.84 -50.94 -10.10 Na2Cr2O7

Na2CrO4 -24.28 -21.17 3.11 Na2CrO4

Nantokite -8.08 -15.21 -7.13 CuCl

Natron -10.78 -12.70 -1.92 Na2CO3:10H2O

Nesquehonite -3.99 -8.44 -4.45 MgCO3:3H2O

O2(g) -39.31 49.08 88.39 O2

Otavite -1.77 -13.76 -11.99 CdCO3

Pb(OH)2 -2.30 6.40 8.69 Pb(OH)2

Pb10(OH)6O(CO3)6 -55.14 -63.90 -8.76 Pb10(OH)6O(CO3)6

Pb2(OH)3Cl -8.71 0.08 8.79 Pb2(OH)3Cl

Pb2O(OH)2 -13.39 12.79 26.19 Pb2O(OH)2

Pb2O3 -23.71 37.33 61.04 Pb2O3

Pb2OCO3 -8.34 -8.52 -0.18 Pb2OCO3

Pb3(AsO4)2 -16.74 -10.94 5.80 Pb3(AsO4)2

Pb3O2CO3 -14.17 -2.12 12.05 Pb3O2CO3

Pb3O2SO4 -12.71 -1.29 11.42 Pb3O2SO4

Pb4(OH)6SO4 -15.99 5.11 21.10 Pb4(OH)6SO4

Pb4O3SO4 -18.04 5.11 23.14 Pb4O3SO4

PbCrO4 -10.37 -23.38 -13.01 PbCrO4

Pbmetal -22.38 -18.14 4.24 Pb

PbO:0.3H2O -6.58 6.40 12.98 PbO:0.33H2O

Periclase -10.11 12.87 22.99 MgO

Phosgenite -14.13 -33.94 -19.81 PbCl2:PbCO3

Plattnerite -21.41 30.94 52.35 PbO2

Portlandite -10.76 13.24 24.00 Ca(OH)2

Smithsonite -2.43 -12.29 -9.85 ZnCO3

Tenorite -1.10 7.15 8.25 CuO

Thenardite -12.28 -11.87 0.41 Na2SO4

Thermonatrite -13.44 -12.70 0.73 Na2CO3:H2O

Zincite -3.14 9.03 12.17 ZnO

Zincosite -16.15 -11.45 4.70 ZnSO4

Zn(NO3)2:6H2O -18.06 -14.98 3.09 Zn(NO3)2:6H2O

Zn(OH)2 -3.17 9.03 12.20 Zn(OH)2

Zn(OH)2(am) -4.20 9.03 13.22 Zn(OH)2

Zn(OH)2(beta) -3.50 9.03 12.53 Zn(OH)2

Zn(OH)2(epsilon) -3.27 9.03 12.29 Zn(OH)2

Zn(OH)2(gamma) -2.71 9.03 11.73 Zn(OH)2

Zn2(OH)2SO4 -9.93 -2.43 7.50 Zn2(OH)2SO4

Zn2(OH)3Cl -9.85 5.34 15.19 Zn2(OH)3Cl

Zn3(AsO4)2:2.5H2O -16.70 -3.05 13.65 Zn3(AsO4)2:2.5H2O

Zn3O(SO4)2 -35.19 -13.88 21.31 Zn3O(SO4)2

Zn4(OH)6SO4 -12.77 15.63 28.40 Zn4(OH)6SO4

Zn5(OH)8Cl2 -18.79 19.71 38.50 Zn5(OH)8Cl2

ZnCl2 -24.12 -16.40 7.72 ZnCl2

ZnCO3:1H2O -2.03 -12.29 -10.26 ZnCO3:1H2O

Znmetal -42.72 -15.51 27.21 Zn

ZnO(active) -2.98 9.03 12.01 ZnO

ZnSO4:1H2O -11.23 -11.45 -0.23 ZnSO4:1H2O

**For a gas, SI = log10(fugacity). Fugacity = pressure * phi / 1 atm.

For ideal gases, phi = 1.

Initial solution 5.

----------------------------Distribution of species----------------------------

Log Log Log mole V

Species Molality Activity Molality Activity Gamma cm?mol

OH- 5.552e-07 5.315e-07 -6.256 -6.275 -0.019 (0)

H+ 6.003e-09 5.749e-09 -8.222 -8.240 -0.019 0.00

H2O 5.551e+01 1.000e+00 1.744 -0.000 0.000 18.02

As(3) 1.266e-20

H3AsO3 1.204e-20 1.204e-20 -19.919 -19.919 0.000 (0)

H2AsO3- 6.258e-22 5.979e-22 -21.204 -21.223 -0.020 (0)

HAsO3-2 5.745e-26 4.787e-26 -25.241 -25.320 -0.079 (0)

H4AsO3+ 3.589e-29 3.429e-29 -28.445 -28.465 -0.020 (0)

AsO3-3 2.817e-31 1.869e-31 -30.550 -30.728 -0.178 (0)

As(5) 8.257e-09

HAsO4-2 7.869e-09 6.557e-09 -8.104 -8.183 -0.079 (0)

H2AsO4- 3.836e-10 3.665e-10 -9.416 -9.436 -0.020 (0)

AsO4-3 3.781e-12 2.508e-12 -11.422 -11.601 -0.178 (0)

H3AsO4 3.145e-16 3.146e-16 -15.502 -15.502 0.000 (0)

C(4) 7.241e-04

HCO3- 7.044e-04 6.750e-04 -3.152 -3.171 -0.019 (0)

H2CO3 1.061e-05 1.061e-05 -4.974 -4.974 0.000 (0)

CO3-2 4.792e-06 4.029e-06 -5.320 -5.395 -0.075 (0)

CaHCO3+ 2.513e-06 2.409e-06 -5.600 -5.618 -0.018 (0)

CaCO3 1.333e-06 1.333e-06 -5.875 -5.875 0.000 (0)

MgHCO3+ 2.468e-07 2.364e-07 -6.608 -6.626 -0.019 (0)

MgCO3 9.666e-08 9.666e-08 -7.015 -7.015 0.000 (0)

NaHCO3 7.441e-08 7.441e-08 -7.128 -7.128 0.000 (0)

CuCO3 2.864e-08 2.864e-08 -7.543 -7.543 0.000 (0)

NaCO3- 1.768e-08 1.694e-08 -7.752 -7.771 -0.019 (0)

ZnCO3 2.716e-09 2.716e-09 -8.566 -8.566 0.000 (0)

CdCO3 1.435e-09 1.435e-09 -8.843 -8.843 0.000 (0)

PbCO3 4.880e-10 4.880e-10 -9.312 -9.312 0.000 (0)

Cu(CO3)2-2 3.728e-10 3.106e-10 -9.429 -9.508 -0.079 (0)

ZnHCO3+ 1.915e-10 1.830e-10 -9.718 -9.738 -0.020 (0)

CuHCO3+ 3.939e-11 3.763e-11 -10.405 -10.424 -0.020 (0)

CdHCO3+ 1.840e-11 1.758e-11 -10.735 -10.755 -0.020 (0)

PbHCO3+ 1.548e-11 1.479e-11 -10.810 -10.830 -0.020 (0)

Pb(CO3)2-2 6.805e-12 5.671e-12 -11.167 -11.246 -0.079 (0)

Cd(CO3)2-2 5.145e-12 4.287e-12 -11.289 -11.368 -0.079 (0)

Ca 3.587e-04

Ca+2 3.495e-04 2.939e-04 -3.457 -3.532 -0.075 (0)

CaSO4 5.196e-06 5.196e-06 -5.284 -5.284 0.000 (0)

CaHCO3+ 2.513e-06 2.409e-06 -5.600 -5.618 -0.018 (0)

CaCO3 1.333e-06 1.333e-06 -5.875 -5.875 0.000 (0)

CaNO3+ 2.320e-07 2.216e-07 -6.635 -6.654 -0.020 (0)

CaOH+ 2.722e-09 2.610e-09 -8.565 -8.583 -0.018 (0)

Cd 2.045e-08

Cd+2 1.858e-08 1.563e-08 -7.731 -7.806 -0.075 (0)

CdCO3 1.435e-09 1.435e-09 -8.843 -8.843 0.000 (0)

CdSO4 2.733e-10 2.733e-10 -9.563 -9.563 0.000 (0)

CdOH+ 7.054e-11 6.740e-11 -10.152 -10.171 -0.020 (0)

CdCl+ 3.966e-11 3.790e-11 -10.402 -10.421 -0.020 (0)

CdHCO3+ 1.840e-11 1.758e-11 -10.735 -10.755 -0.020 (0)

CdNO3+ 1.722e-11 1.645e-11 -10.764 -10.784 -0.020 (0)

Cd(CO3)2-2 5.145e-12 4.287e-12 -11.289 -11.368 -0.079 (0)

Cd(OH)2 2.403e-12 2.403e-12 -11.619 -11.619 0.000 (0)

CdOHCl 1.884e-12 1.884e-12 -11.725 -11.725 0.000 (0)

Cd(SO4)2-2 4.787e-13 3.988e-13 -12.320 -12.399 -0.079 (0)

CdCl2 3.927e-15 3.927e-15 -14.406 -14.406 0.000 (0)

Cd(NO3)2 1.118e-15 1.118e-15 -14.951 -14.951 0.000 (0)

Cd(OH)3- 2.691e-16 2.571e-16 -15.570 -15.590 -0.020 (0)

Cd2OH+3 9.645e-18 6.398e-18 -17.016 -17.194 -0.178 (0)

CdCl3- 5.792e-20 5.534e-20 -19.237 -19.257 -0.020 (0)

Cd(OH)4-2 8.847e-23 7.372e-23 -22.053 -22.132 -0.079 (0)

Cl 2.709e-05

Cl- 2.709e-05 2.594e-05 -4.567 -4.586 -0.019 (0)

CdCl+ 3.966e-11 3.790e-11 -10.402 -10.421 -0.020 (0)

CdOHCl 1.884e-12 1.884e-12 -11.725 -11.725 0.000 (0)

ZnOHCl 1.750e-12 1.750e-12 -11.757 -11.757 0.000 (0)

CuCl 1.666e-12 1.666e-12 -11.778 -11.778 0.000 (0)

ZnCl+ 7.101e-13 6.800e-13 -12.149 -12.167 -0.019 (0)

CuCl+ 4.340e-14 4.156e-14 -13.362 -13.381 -0.019 (0)

PbCl+ 3.223e-14 3.079e-14 -13.492 -13.512 -0.020 (0)

CuCl2- 9.791e-15 9.375e-15 -14.009 -14.028 -0.019 (0)

CdCl2 3.927e-15 3.927e-15 -14.406 -14.406 0.000 (0)

ZnCl2 1.423e-17 1.423e-17 -16.847 -16.847 0.000 (0)

CrCl+2 1.417e-17 1.180e-17 -16.849 -16.928 -0.079 (0)

PbCl2 3.325e-18 3.325e-18 -17.478 -17.478 0.000 (0)

CuCl2 1.736e-19 1.736e-19 -18.760 -18.760 0.000 (0)

CuCl3-2 5.807e-20 4.893e-20 -19.236 -19.310 -0.074 (0)

CdCl3- 5.792e-20 5.534e-20 -19.237 -19.257 -0.020 (0)

CrOHCl2 1.114e-21 1.114e-21 -20.953 -20.953 0.000 (0)

ZnCl3- 2.872e-22 2.750e-22 -21.542 -21.561 -0.019 (0)

PbCl3- 4.264e-23 4.074e-23 -22.370 -22.390 -0.020 (0)

CrCl2+ 7.681e-24 7.338e-24 -23.115 -23.134 -0.020 (0)

CuCl3- 3.318e-26 3.178e-26 -25.479 -25.498 -0.019 (0)

ZnCl4-2 3.736e-27 3.148e-27 -26.428 -26.502 -0.074 (0)

CrO3Cl- 7.391e-28 7.061e-28 -27.131 -27.151 -0.020 (0)

PbCl4-2 4.605e-28 3.837e-28 -27.337 -27.416 -0.079 (0)

CuCl4-2 8.317e-33 7.008e-33 -32.080 -32.154 -0.074 (0)

Cr(2) 9.612e-25

Cr+2 9.612e-25 8.009e-25 -24.017 -24.096 -0.079 (0)

Cr(3) 5.770e-07

Cr(OH)2+ 3.283e-07 3.137e-07 -6.484 -6.504 -0.020 (0)

Cr(OH)3 2.064e-07 2.064e-07 -6.685 -6.685 0.000 (0)

CrO2- 1.785e-08 1.705e-08 -7.748 -7.768 -0.020 (0)

Cr(OH)4- 1.506e-08 1.439e-08 -7.822 -7.842 -0.020 (0)

Cr(OH)+2 9.335e-09 7.779e-09 -8.030 -8.109 -0.079 (0)

CrOHSO4 3.138e-11 3.138e-11 -10.503 -10.503 0.000 (0)

Cr+3 9.240e-13 6.129e-13 -12.034 -12.213 -0.178 (0)

CrSO4+ 6.938e-14 6.629e-14 -13.159 -13.179 -0.020 (0)

CrCl+2 1.417e-17 1.180e-17 -16.849 -16.928 -0.079 (0)

Cr2(OH)2SO4+2 5.009e-18 4.174e-18 -17.300 -17.379 -0.079 (0)

CrNO3+2 1.733e-18 1.444e-18 -17.761 -17.840 -0.079 (0)

Cr2(OH)2(SO4)2 2.227e-20 2.227e-20 -19.652 -19.652 0.000 (0)

CrOHCl2 1.114e-21 1.114e-21 -20.953 -20.953 0.000 (0)

CrCl2+ 7.681e-24 7.338e-24 -23.115 -23.134 -0.020 (0)

Cr(6) 4.892e-14

CrO4-2 4.813e-14 4.047e-14 -13.318 -13.393 -0.075 (0)

HCrO4- 7.550e-16 7.214e-16 -15.122 -15.142 -0.020 (0)

NaCrO4- 3.077e-17 2.940e-17 -16.512 -16.532 -0.020 (0)

KCrO4- 5.123e-18 4.894e-18 -17.290 -17.310 -0.020 (0)

H2CrO4 1.525e-24 1.525e-24 -23.817 -23.817 0.000 (0)

CrO3SO4-2 1.421e-25 1.184e-25 -24.847 -24.927 -0.079 (0)

CrO3Cl- 7.391e-28 7.061e-28 -27.131 -27.151 -0.020 (0)

Cr2O7-2 3.250e-29 2.708e-29 -28.488 -28.567 -0.079 (0)

Cu(1) 5.507e-11

Cu+ 5.340e-11 5.102e-11 -10.272 -10.292 -0.020 (0)

CuCl 1.666e-12 1.666e-12 -11.778 -11.778 0.000 (0)

CuCl2- 9.791e-15 9.375e-15 -14.009 -14.028 -0.019 (0)

CuCl3-2 5.807e-20 4.893e-20 -19.236 -19.310 -0.074 (0)

Cu(2) 3.610e-08

CuCO3 2.864e-08 2.864e-08 -7.543 -7.543 0.000 (0)

CuOH+ 3.248e-09 3.110e-09 -8.488 -8.507 -0.019 (0)

Cu(OH)2 2.337e-09 2.337e-09 -8.631 -8.631 0.000 (0)

Cu+2 1.435e-09 1.207e-09 -8.843 -8.918 -0.075 (0)

Cu(CO3)2-2 3.728e-10 3.106e-10 -9.429 -9.508 -0.079 (0)

CuHCO3+ 3.939e-11 3.763e-11 -10.405 -10.424 -0.020 (0)

CuSO4 2.063e-11 2.063e-11 -10.686 -10.686 0.000 (0)

Cu(OH)3- 8.786e-12 8.395e-12 -11.056 -11.076 -0.020 (0)

CuNO3+ 9.267e-13 8.854e-13 -12.033 -12.053 -0.020 (0)

Cu2(OH)2+2 2.621e-13 2.184e-13 -12.582 -12.661 -0.079 (0)

CuCl+ 4.340e-14 4.156e-14 -13.362 -13.381 -0.019 (0)

Cu(OH)4-2 1.389e-16 1.157e-16 -15.857 -15.937 -0.079 (0)

Cu(NO3)2 2.170e-17 2.170e-17 -16.664 -16.664 0.000 (0)

CuCl2 1.736e-19 1.736e-19 -18.760 -18.760 0.000 (0)

CuCl3- 3.318e-26 3.178e-26 -25.479 -25.498 -0.019 (0)

CuCl4-2 8.317e-33 7.008e-33 -32.080 -32.154 -0.074 (0)

H(0) 5.474e-28

H2 2.737e-28 2.738e-28 -27.563 -27.563 0.000 (0)

K 3.401e-05

K+ 3.399e-05 3.255e-05 -4.469 -4.487 -0.019 (0)

KSO4- 1.979e-08 1.897e-08 -7.704 -7.722 -0.019 (0)

KCrO4- 5.123e-18 4.894e-18 -17.290 -17.310 -0.020 (0)

Mg 4.521e-05

Mg+2 4.432e-05 3.727e-05 -4.353 -4.429 -0.075 (0)

MgSO4 5.383e-07 5.383e-07 -6.269 -6.269 0.000 (0)

MgHCO3+ 2.468e-07 2.364e-07 -6.608 -6.626 -0.019 (0)

MgCO3 9.666e-08 9.666e-08 -7.015 -7.015 0.000 (0)

MgOH+ 6.364e-09 6.102e-09 -8.196 -8.215 -0.018 (0)

N(5) 2.221e-04

NO3- 2.219e-04 2.125e-04 -3.654 -3.673 -0.019 (0)

CaNO3+ 2.320e-07 2.216e-07 -6.635 -6.654 -0.020 (0)

CdNO3+ 1.722e-11 1.645e-11 -10.764 -10.784 -0.020 (0)

ZnNO3+ 7.219e-12 6.897e-12 -11.142 -11.161 -0.020 (0)

CuNO3+ 9.267e-13 8.854e-13 -12.033 -12.053 -0.020 (0)

PbNO3+ 1.270e-13 1.213e-13 -12.896 -12.916 -0.020 (0)

Cd(NO3)2 1.118e-15 1.118e-15 -14.951 -14.951 0.000 (0)

Zn(NO3)2 2.650e-16 2.650e-16 -15.577 -15.577 0.000 (0)

Pb(NO3)2 5.261e-17 5.261e-17 -16.279 -16.279 0.000 (0)

Cu(NO3)2 2.170e-17 2.170e-17 -16.664 -16.664 0.000 (0)

CrNO3+2 1.733e-18 1.444e-18 -17.761 -17.840 -0.079 (0)

Na 1.528e-04

Na+ 1.527e-04 1.462e-04 -3.816 -3.835 -0.019 (0)

NaHCO3 7.441e-08 7.441e-08 -7.128 -7.128 0.000 (0)

NaSO4- 7.205e-08 6.904e-08 -7.142 -7.161 -0.019 (0)

NaCO3- 1.768e-08 1.694e-08 -7.752 -7.771 -0.019 (0)

NaCrO4- 3.077e-17 2.940e-17 -16.512 -16.532 -0.020 (0)

O(0) 0.000e+00

O2 0.000e+00 0.000e+00 -41.497 -41.497 0.000 (0)

Pb 7.555e-10

PbCO3 4.880e-10 4.880e-10 -9.312 -9.312 0.000 (0)

PbOH+ 1.855e-10 1.772e-10 -9.732 -9.751 -0.020 (0)

Pb+2 4.791e-11 4.029e-11 -10.320 -10.395 -0.075 (0)

PbHCO3+ 1.548e-11 1.479e-11 -10.810 -10.830 -0.020 (0)

Pb(OH)2 9.818e-12 9.818e-12 -11.008 -11.008 0.000 (0)

Pb(CO3)2-2 6.805e-12 5.671e-12 -11.167 -11.246 -0.079 (0)

PbSO4 1.773e-12 1.773e-12 -11.751 -11.751 0.000 (0)

PbNO3+ 1.270e-13 1.213e-13 -12.896 -12.916 -0.020 (0)

PbCl+ 3.223e-14 3.079e-14 -13.492 -13.512 -0.020 (0)

Pb(OH)3- 1.800e-14 1.720e-14 -13.745 -13.765 -0.020 (0)

Pb(SO4)2-2 1.152e-15 9.596e-16 -14.939 -15.018 -0.079 (0)

Pb(NO3)2 5.261e-17 5.261e-17 -16.279 -16.279 0.000 (0)

Pb(OH)4-2 8.853e-18 7.377e-18 -17.053 -17.132 -0.079 (0)

PbCl2 3.325e-18 3.325e-18 -17.478 -17.478 0.000 (0)

Pb2OH+3 1.706e-19 1.132e-19 -18.768 -18.946 -0.178 (0)

PbCl3- 4.264e-23 4.074e-23 -22.370 -22.390 -0.020 (0)

Pb3(OH)4+2 7.923e-24 6.602e-24 -23.101 -23.180 -0.079 (0)

PbCl4-2 4.605e-28 3.837e-28 -27.337 -27.416 -0.079 (0)

Pb4(OH)4+4 7.805e-30 3.762e-30 -29.108 -29.425 -0.317 (0)

S(6) 1.127e-04

SO4-2 1.068e-04 8.984e-05 -3.971 -4.047 -0.075 (0)

CaSO4 5.196e-06 5.196e-06 -5.284 -5.284 0.000 (0)

MgSO4 5.383e-07 5.383e-07 -6.269 -6.269 0.000 (0)

NaSO4- 7.205e-08 6.904e-08 -7.142 -7.161 -0.019 (0)

KSO4- 1.979e-08 1.897e-08 -7.704 -7.722 -0.019 (0)

CdSO4 2.733e-10 2.733e-10 -9.563 -9.563 0.000 (0)

ZnSO4 2.016e-10 2.016e-10 -9.695 -9.695 0.000 (0)

HSO4- 3.293e-11 3.154e-11 -10.482 -10.501 -0.019 (0)

CrOHSO4 3.138e-11 3.138e-11 -10.503 -10.503 0.000 (0)

CuSO4 2.063e-11 2.063e-11 -10.686 -10.686 0.000 (0)

PbSO4 1.773e-12 1.773e-12 -11.751 -11.751 0.000 (0)

Cd(SO4)2-2 4.787e-13 3.988e-13 -12.320 -12.399 -0.079 (0)

Zn(SO4)2-2 2.162e-13 1.801e-13 -12.665 -12.744 -0.079 (0)

CrSO4+ 6.938e-14 6.629e-14 -13.159 -13.179 -0.020 (0)

Pb(SO4)2-2 1.152e-15 9.596e-16 -14.939 -15.018 -0.079 (0)

Cr2(OH)2SO4+2 5.009e-18 4.174e-18 -17.300 -17.379 -0.079 (0)

Cr2(OH)2(SO4)2 2.227e-20 2.227e-20 -19.652 -19.652 0.000 (0)

CrO3SO4-2 1.421e-25 1.184e-25 -24.847 -24.927 -0.079 (0)

Zn 1.827e-08

Zn+2 1.393e-08 1.171e-08 -7.856 -7.931 -0.075 (0)

ZnCO3 2.716e-09 2.716e-09 -8.566 -8.566 0.000 (0)

ZnOH+ 6.515e-10 6.225e-10 -9.186 -9.206 -0.020 (0)

Zn(OH)2 5.695e-10 5.695e-10 -9.245 -9.245 0.000 (0)

ZnSO4 2.016e-10 2.016e-10 -9.695 -9.695 0.000 (0)

ZnHCO3+ 1.915e-10 1.830e-10 -9.718 -9.738 -0.020 (0)

ZnNO3+ 7.219e-12 6.897e-12 -11.142 -11.161 -0.020 (0)

Zn(OH)3- 5.233e-12 4.999e-12 -11.281 -11.301 -0.020 (0)

ZnOHCl 1.750e-12 1.750e-12 -11.757 -11.757 0.000 (0)

ZnCl+ 7.101e-13 6.800e-13 -12.149 -12.167 -0.019 (0)

Zn(SO4)2-2 2.162e-13 1.801e-13 -12.665 -12.744 -0.079 (0)

Zn(OH)4-2 4.184e-16 3.486e-16 -15.378 -15.458 -0.079 (0)

Zn(NO3)2 2.650e-16 2.650e-16 -15.577 -15.577 0.000 (0)

ZnCl2 1.423e-17 1.423e-17 -16.847 -16.847 0.000 (0)

ZnCl3- 2.872e-22 2.750e-22 -21.542 -21.561 -0.019 (0)

ZnCl4-2 3.736e-27 3.148e-27 -26.428 -26.502 -0.074 (0)

------------------------------Saturation indices-------------------------------

Phase SI** log IAP log K(283 K, 1 atm)

Anglesite -6.54 -14.44 -7.90 PbSO4

Anhydrite -3.29 -7.58 -4.29 CaSO4

Antlerite -6.63 2.16 8.79 Cu3(OH)4SO4

Aragonite -0.74 -8.93 -8.19 CaCO3

Arsenolite -76.36 -79.68 -3.32 As4O6

Artinite -8.49 2.23 10.72 MgCO3:Mg(OH)2:3H2O

As2O5 -37.92 -31.00 6.92 As2O5

Atacamite -5.96 2.30 8.26 Cu2(OH)3Cl

Azurite -5.04 -21.06 -16.02 Cu3(OH)2(CO3)2

Bianchite -10.22 -11.98 -1.76 ZnSO4:6H2O

Brochantite -7.38 9.72 17.10 Cu4(OH)6SO4

Brucite -5.85 12.05 17.90 Mg(OH)2

Ca3(AsO4)2:4H2O -14.46 7.84 22.30 Ca3(AsO4)2:4H2O

CaCrO4 -14.91 -16.92 -2.02 CaCrO4

Calcite -0.52 -8.93 -8.41 CaCO3

Cd(OH)2 -5.85 8.67 14.52 Cd(OH)2

Cd(OH)2(am) -5.86 8.67 14.54 Cd(OH)2

Cd3(OH)2(SO4)2 -21.74 -15.03 6.71 Cd3(OH)2(SO4)2

Cd3(OH)4SO4 -17.06 5.50 22.56 Cd3(OH)4SO4

Cd4(OH)6SO4 -14.23 14.17 28.40 Cd4(OH)6SO4

CdCl2 -16.49 -16.98 -0.49 CdCl2

CdCl2:1H2O -15.35 -16.98 -1.62 CdCl2:1H2O

CdCl2:2.5H2O -15.00 -16.98 -1.98 CdCl2:2.5H2O

Cdmetal(alpha) -30.02 -15.81 14.21 Cd

Cdmetal(gamma) -30.13 -15.81 14.32 Cd

CdOHCl -7.98 -4.15 3.82 CdOHCl

CdSO4 -12.16 -11.85 0.31 CdSO4

CdSO4:1H2O -10.42 -11.85 -1.43 CdSO4:1H2O

CdSO4:2.67H2O -10.15 -11.85 -1.71 CdSO4:2.67H2O

Cerussite -2.43 -15.79 -13.36 PbCO3

CH4(g) -76.37 -119.80 -43.43 CH4

Chalcanthite -10.27 -12.96 -2.70 CuSO4:5H2O

Claudetite -76.10 -79.68 -3.58 As4O6

CO2(g) -3.69 -21.88 -18.18 CO2

Cotunnite -14.54 -19.57 -5.02 PbCl2

Cr(OH)2 -18.76 -7.62 11.15 Cr(OH)2

Cr(OH)3 0.13 1.74 1.61 Cr(OH)3

Cr(OH)3(am) 2.49 1.74 -0.75 Cr(OH)3

Cr2O3 5.36 3.47 -1.89 Cr2O3

CrCl2 -48.39 -33.27 15.12 CrCl2

CrCl3 -52.98 -36.74 16.24 CrCl3

Crmetal -64.18 -32.10 32.08 Cr

CrO3 -26.71 -29.87 -3.16 CrO3

Cu(OH)2 -1.64 7.56 9.20 Cu(OH)2

Cu2(OH)3NO3 -6.71 3.21 9.92 Cu2(OH)3NO3

Cu2SO4 -22.86 -24.63 -1.77 Cu2SO4

Cu3(AsO4)2:2H2O -14.42 -8.32 6.10 Cu3(AsO4)2:2H2O

CuCO3 -2.81 -14.31 -11.50 CuCO3

CuCrO4 -16.87 -22.31 -5.44 CuCrO4

Cumetal -4.87 -14.29 -9.42 Cu

CuOCuSO4 -16.98 -5.40 11.58 CuOCuSO4

Cuprite -3.85 -4.10 -0.26 Cu2O

CuSO4 -16.58 -12.96 3.62 CuSO4

Dolomite(disordered) -2.64 -18.75 -16.11 CaMg(CO3)2

Dolomite(ordered) -2.03 -18.75 -16.72 CaMg(CO3)2

Epsomite -6.24 -8.48 -2.23 MgSO4:7H2O

Goslarite -9.83 -11.98 -2.14 ZnSO4:7H2O

Gypsum -2.96 -7.58 -4.62 CaSO4:2H2O

Halite -9.99 -8.42 1.57 NaCl

Huntite -9.43 -38.40 -28.97 CaMg3(CO3)4

Hydrocerussite -6.72 -25.49 -18.77 Pb3(OH)2(CO3)2

Hydromagnesite -20.50 -27.24 -6.74 Mg5(CO3)4(OH)2:4H2O

K2Cr2O7 -34.25 -52.24 -17.99 K2Cr2O7

K2CrO4 -21.68 -22.37 -0.68 K2CrO4

Langite -9.30 9.72 19.03 Cu4(OH)6SO4:H2O

Larnakite -8.12 -8.36 -0.23 PbO:PbSO4

Laurionite -7.36 -6.74 0.62 PbOHCl

Lime -21.55 12.95 34.50 CaO

Litharge -7.22 6.09 13.30 PbO

Magnesite -2.18 -9.82 -7.65 MgCO3

Malachite -0.74 -6.75 -6.01 Cu2(OH)2CO3

Massicot -7.43 6.09 13.51 PbO

Melanothallite -24.94 -18.09 6.85 CuCl2

Mg(OH)2(active) -6.74 12.05 18.79 Mg(OH)2

MgCr2O4 -2.34 15.53 17.87 MgCr2O4

MgCrO4 -24.03 -17.82 6.21 MgCrO4

Minium -34.70 42.74 77.44 Pb3O4

Mirabilite -9.87 -11.72 -1.85 Na2SO4:10H2O

Monteponite -7.39 8.67 16.06 CdO

Na2Cr2O7 -40.84 -50.94 -10.10 Na2Cr2O7

Na2CrO4 -24.18 -21.06 3.11 Na2CrO4

Nantokite -7.75 -14.88 -7.13 CuCl

Natron -11.14 -13.07 -1.92 Na2CO3:10H2O

Nesquehonite -5.38 -9.82 -4.45 MgCO3:3H2O

O2(g) -39.43 48.96 88.39 O2

Otavite -1.21 -13.20 -11.99 CdCO3

Pb(OH)2 -2.61 6.09 8.69 Pb(OH)2

Pb10(OH)6O(CO3)6 -61.63 -70.39 -8.76 Pb10(OH)6O(CO3)6

Pb2(OH)3Cl -9.45 -0.65 8.79 Pb2(OH)3Cl

Pb2O(OH)2 -14.02 12.17 26.19 Pb2O(OH)2

Pb2O3 -24.39 36.65 61.04 Pb2O3

Pb2OCO3 -9.52 -9.70 -0.18 Pb2OCO3

Pb3(AsO4)2 -18.55 -12.75 5.80 Pb3(AsO4)2

Pb3O2CO3 -15.66 -3.62 12.05 Pb3O2CO3

Pb3O2SO4 -13.69 -2.27 11.42 Pb3O2SO4

Pb4(OH)6SO4 -17.28 3.82 21.10 Pb4(OH)6SO4

Pb4O3SO4 -19.33 3.82 23.14 Pb4O3SO4

PbCrO4 -10.78 -23.79 -13.01 PbCrO4

Pbmetal -22.63 -18.39 4.24 Pb

PbO:0.3H2O -6.89 6.09 12.98 PbO:0.33H2O

Periclase -10.94 12.05 22.99 MgO

Phosgenite -15.55 -35.36 -19.81 PbCl2:PbCO3

Plattnerite -21.78 30.57 52.35 PbO2

Portlandite -11.05 12.95 24.00 Ca(OH)2

Smithsonite -3.47 -13.33 -9.85 ZnCO3

Tenorite -0.68 7.56 8.25 CuO

Thenardite -12.12 -11.72 0.41 Na2SO4

Thermonatrite -13.80 -13.06 0.73 Na2CO3:H2O

Zincite -3.62 8.55 12.17 ZnO

Zincosite -16.67 -11.98 4.70 ZnSO4

Zn(NO3)2:6H2O -18.36 -15.28 3.09 Zn(NO3)2:6H2O

Zn(OH)2 -3.65 8.55 12.20 Zn(OH)2

Zn(OH)2(am) -4.67 8.55 13.22 Zn(OH)2

Zn(OH)2(beta) -3.98 8.55 12.53 Zn(OH)2

Zn(OH)2(epsilon) -3.74 8.55 12.29 Zn(OH)2

Zn(OH)2(gamma) -3.18 8.55 11.73 Zn(OH)2

Zn2(OH)2SO4 -10.93 -3.43 7.50 Zn2(OH)2SO4

Zn2(OH)3Cl -10.92 4.27 15.19 Zn2(OH)3Cl

Zn3(AsO4)2:2.5H2O -19.01 -5.36 13.65 Zn3(AsO4)2:2.5H2O

Zn3O(SO4)2 -36.71 -15.41 21.31 Zn3O(SO4)2

Zn4(OH)6SO4 -14.73 13.67 28.40 Zn4(OH)6SO4

Zn5(OH)8Cl2 -21.41 17.09 38.50 Zn5(OH)8Cl2

ZnCl2 -24.83 -17.10 7.72 ZnCl2

ZnCO3:1H2O -3.07 -13.33 -10.26 ZnCO3:1H2O

Znmetal -43.14 -15.93 27.21 Zn

ZnO(active) -3.46 8.55 12.01 ZnO

ZnSO4:1H2O -11.75 -11.98 -0.23 ZnSO4:1H2O

**For a gas, SI = log10(fugacity). Fugacity = pressure * phi / 1 atm.

For ideal gases, phi = 1.

Initial solution 6.

----------------------------Distribution of species----------------------------

Log Log Log mole V

Species Molality Activity Molality Activity Gamma cm?mol

OH- 7.506e-08 6.999e-08 -7.125 -7.155 -0.030 (0)

H+ 4.678e-08 4.365e-08 -7.330 -7.360 -0.030 0.00

H2O 5.551e+01 9.999e-01 1.744 -0.000 0.000 18.02

As(3) 9.576e-17

H3AsO3 9.509e-17 9.509e-17 -16.022 -16.022 0.000 (0)

H2AsO3- 6.706e-19 6.220e-19 -18.174 -18.206 -0.033 (0)

HAsO3-2 8.862e-24 6.558e-24 -23.052 -23.183 -0.131 (0)

H4AsO3+ 2.217e-24 2.057e-24 -23.654 -23.687 -0.033 (0)

AsO3-3 6.637e-30 3.372e-30 -29.178 -29.472 -0.294 (0)

As(5) 2.818e-08

HAsO4-2 2.105e-08 1.558e-08 -7.677 -7.807 -0.131 (0)

H2AsO4- 7.129e-09 6.612e-09 -8.147 -8.180 -0.033 (0)

AsO4-3 1.545e-12 7.848e-13 -11.811 -12.105 -0.294 (0)

H3AsO4 4.306e-14 4.310e-14 -13.366 -13.366 0.000 (0)

C(4) 3.327e-03

HCO3- 2.962e-03 2.769e-03 -2.528 -2.558 -0.029 (0)

H2CO3 3.306e-04 3.306e-04 -3.481 -3.481 0.000 (0)

CaHCO3+ 2.493e-05 2.332e-05 -4.603 -4.632 -0.029 (0)

MgHCO3+ 3.941e-06 3.677e-06 -5.404 -5.434 -0.030 (0)

CO3-2 2.870e-06 2.177e-06 -5.542 -5.662 -0.120 (0)

CaCO3 1.699e-06 1.699e-06 -5.770 -5.770 0.000 (0)

NaHCO3 4.107e-07 4.107e-07 -6.386 -6.386 0.000 (0)

MgCO3 1.981e-07 1.981e-07 -6.703 -6.703 0.000 (0)

CuCO3 3.123e-08 3.123e-08 -7.505 -7.505 0.000 (0)

NaCO3- 1.318e-08 1.232e-08 -7.880 -7.909 -0.029 (0)

ZnCO3 5.013e-09 5.013e-09 -8.300 -8.300 0.000 (0)

ZnHCO3+ 2.766e-09 2.565e-09 -8.558 -8.591 -0.033 (0)

PbCO3 1.932e-09 1.932e-09 -8.714 -8.714 0.000 (0)

CdCO3 7.245e-10 7.245e-10 -9.140 -9.140 0.000 (0)

PbHCO3+ 4.793e-10 4.446e-10 -9.319 -9.352 -0.033 (0)

CuHCO3+ 3.359e-10 3.115e-10 -9.474 -9.506 -0.033 (0)

Cu(CO3)2-2 2.472e-10 1.830e-10 -9.607 -9.738 -0.131 (0)

CdHCO3+ 7.265e-11 6.738e-11 -10.139 -10.171 -0.033 (0)

Pb(CO3)2-2 1.639e-11 1.213e-11 -10.786 -10.916 -0.131 (0)

Cd(CO3)2-2 1.580e-12 1.169e-12 -11.801 -11.932 -0.131 (0)

Ca 9.561e-04

Ca+2 9.143e-04 6.934e-04 -3.039 -3.159 -0.120 (0)

CaHCO3+ 2.493e-05 2.332e-05 -4.603 -4.632 -0.029 (0)

CaSO4 1.430e-05 1.430e-05 -4.845 -4.845 0.000 (0)

CaCO3 1.699e-06 1.699e-06 -5.770 -5.770 0.000 (0)

CaNO3+ 8.847e-07 8.206e-07 -6.053 -6.086 -0.033 (0)

CaOH+ 8.668e-10 8.109e-10 -9.062 -9.091 -0.029 (0)

Cd 2.045e-08

Cd+2 1.926e-08 1.460e-08 -7.715 -7.836 -0.120 (0)

CdCO3 7.245e-10 7.245e-10 -9.140 -9.140 0.000 (0)

CdSO4 2.979e-10 2.979e-10 -9.526 -9.526 0.000 (0)

CdHCO3+ 7.265e-11 6.738e-11 -10.139 -10.171 -0.033 (0)

CdCl+ 6.044e-11 5.606e-11 -10.219 -10.251 -0.033 (0)

CdNO3+ 2.600e-11 2.412e-11 -10.585 -10.618 -0.033 (0)

CdOH+ 8.942e-12 8.294e-12 -11.049 -11.081 -0.033 (0)

Cd(CO3)2-2 1.580e-12 1.169e-12 -11.801 -11.932 -0.131 (0)

Cd(SO4)2-2 6.851e-13 5.071e-13 -12.164 -12.295 -0.131 (0)

CdOHCl 3.671e-13 3.671e-13 -12.435 -12.435 0.000 (0)

Cd(OH)2 3.894e-14 3.894e-14 -13.410 -13.410 0.000 (0)

CdCl2 9.194e-15 9.194e-15 -14.036 -14.036 0.000 (0)

Cd(NO3)2 2.573e-15 2.573e-15 -14.590 -14.590 0.000 (0)

Cd2OH+3 1.448e-18 7.357e-19 -17.839 -18.133 -0.294 (0)

Cd(OH)3- 5.916e-19 5.487e-19 -18.228 -18.261 -0.033 (0)

CdCl3- 2.211e-19 2.051e-19 -18.655 -18.688 -0.033 (0)

Cd(OH)4-2 2.799e-26 2.072e-26 -25.553 -25.684 -0.131 (0)

Cl 4.400e-05

Cl- 4.400e-05 4.106e-05 -4.357 -4.387 -0.030 (0)

CdCl+ 6.044e-11 5.606e-11 -10.219 -10.251 -0.033 (0)

CuCl 5.323e-12 5.323e-12 -11.274 -11.274 0.000 (0)

ZnCl+ 3.942e-12 3.678e-12 -11.404 -11.434 -0.030 (0)

ZnOHCl 1.247e-12 1.247e-12 -11.904 -11.904 0.000 (0)

PbCl+ 3.850e-13 3.571e-13 -12.415 -12.447 -0.033 (0)

CdOHCl 3.671e-13 3.671e-13 -12.435 -12.435 0.000 (0)

CuCl+ 1.423e-13 1.328e-13 -12.847 -12.877 -0.030 (0)

CuCl2- 5.081e-14 4.741e-14 -13.294 -13.324 -0.030 (0)

CdCl2 9.194e-15 9.194e-15 -14.036 -14.036 0.000 (0)

CrCl+2 1.883e-15 1.394e-15 -14.725 -14.856 -0.131 (0)

ZnCl2 1.218e-16 1.218e-16 -15.914 -15.914 0.000 (0)

PbCl2 6.104e-17 6.104e-17 -16.214 -16.214 0.000 (0)

CuCl2 8.781e-19 8.781e-19 -18.056 -18.056 0.000 (0)

CuCl3-2 5.140e-19 3.917e-19 -18.289 -18.407 -0.118 (0)

CdCl3- 2.211e-19 2.051e-19 -18.655 -18.688 -0.033 (0)

CrOHCl2 2.740e-20 2.740e-20 -19.562 -19.562 0.000 (0)

ZnCl3- 3.995e-21 3.728e-21 -20.398 -20.429 -0.030 (0)

CrCl2+ 1.478e-21 1.371e-21 -20.830 -20.863 -0.033 (0)

PbCl3- 1.276e-21 1.184e-21 -20.894 -20.927 -0.033 (0)

CuCl3- 2.726e-25 2.544e-25 -24.564 -24.595 -0.030 (0)

ZnCl4-2 8.863e-26 6.753e-26 -25.052 -25.170 -0.118 (0)

PbCl4-2 2.385e-26 1.765e-26 -25.622 -25.753 -0.131 (0)

CrO3Cl- 4.688e-31 4.348e-31 -30.329 -30.362 -0.033 (0)

CuCl4-2 1.165e-31 8.880e-32 -30.934 -31.052 -0.118 (0)

Cr(2) 8.071e-23

Cr+2 8.071e-23 5.973e-23 -22.093 -22.224 -0.131 (0)

Cr(3) 5.770e-07

Cr(OH)2+ 4.374e-07 4.057e-07 -6.359 -6.392 -0.033 (0)

Cr(OH)+2 1.032e-07 7.640e-08 -6.986 -7.117 -0.131 (0)

Cr(OH)3 3.515e-08 3.515e-08 -7.454 -7.454 0.000 (0)

CrO2- 4.123e-10 3.824e-10 -9.385 -9.417 -0.033 (0)

CrOHSO4 3.594e-10 3.594e-10 -9.444 -9.444 0.000 (0)

Cr(OH)4- 3.480e-10 3.228e-10 -9.458 -9.491 -0.033 (0)

Cr+3 8.998e-11 4.571e-11 -10.046 -10.340 -0.294 (0)

CrSO4+ 6.217e-12 5.766e-12 -11.206 -11.239 -0.033 (0)

CrCl+2 1.883e-15 1.394e-15 -14.725 -14.856 -0.131 (0)

Cr2(OH)2SO4+2 6.345e-16 4.696e-16 -15.198 -15.328 -0.131 (0)

CrNO3+2 2.284e-16 1.690e-16 -15.641 -15.772 -0.131 (0)

Cr2(OH)2(SO4)2 2.923e-18 2.923e-18 -17.534 -17.534 0.000 (0)

CrOHCl2 2.740e-20 2.740e-20 -19.562 -19.562 0.000 (0)

CrCl2+ 1.478e-21 1.371e-21 -20.830 -20.863 -0.033 (0)

Cr(6) 4.002e-19

CrO4-2 3.600e-19 2.730e-19 -18.444 -18.564 -0.120 (0)

HCrO4- 3.984e-20 3.695e-20 -19.400 -19.432 -0.033 (0)

NaCrO4- 2.877e-22 2.669e-22 -21.541 -21.574 -0.033 (0)

KCrO4- 4.602e-23 4.268e-23 -22.337 -22.370 -0.033 (0)

H2CrO4 5.930e-28 5.930e-28 -27.227 -27.227 0.000 (0)

CrO3SO4-2 7.260e-29 5.373e-29 -28.139 -28.270 -0.131 (0)

CrO3Cl- 4.688e-31 4.348e-31 -30.329 -30.362 -0.033 (0)

Cr2O7-2 9.602e-38 7.106e-38 -37.018 -37.148 -0.131 (0)

Cu(1) 1.164e-10

Cu+ 1.110e-10 1.030e-10 -9.955 -9.987 -0.033 (0)

CuCl 5.323e-12 5.323e-12 -11.274 -11.274 0.000 (0)

CuCl2- 5.081e-14 4.741e-14 -13.294 -13.324 -0.030 (0)

CuCl3-2 5.140e-19 3.917e-19 -18.289 -18.407 -0.118 (0)

Cu(2) 3.604e-08

CuCO3 3.123e-08 3.123e-08 -7.505 -7.505 0.000 (0)

Cu+2 3.212e-09 2.436e-09 -8.493 -8.613 -0.120 (0)

CuOH+ 8.860e-10 8.267e-10 -9.053 -9.083 -0.030 (0)

CuHCO3+ 3.359e-10 3.115e-10 -9.474 -9.506 -0.033 (0)

Cu(CO3)2-2 2.472e-10 1.830e-10 -9.607 -9.738 -0.131 (0)

Cu(OH)2 8.178e-11 8.178e-11 -10.087 -10.087 0.000 (0)

CuSO4 4.856e-11 4.856e-11 -10.314 -10.314 0.000 (0)

CuNO3+ 3.023e-12 2.804e-12 -11.520 -11.552 -0.033 (0)

CuCl+ 1.423e-13 1.328e-13 -12.847 -12.877 -0.030 (0)

Cu(OH)3- 4.171e-14 3.869e-14 -13.380 -13.412 -0.033 (0)

Cu2(OH)2+2 2.084e-14 1.543e-14 -13.681 -13.812 -0.131 (0)

Cu(NO3)2 1.078e-16 1.078e-16 -15.967 -15.967 0.000 (0)

CuCl2 8.781e-19 8.781e-19 -18.056 -18.056 0.000 (0)

Cu(OH)4-2 9.490e-20 7.024e-20 -19.023 -19.153 -0.131 (0)

CuCl3- 2.726e-25 2.544e-25 -24.564 -24.595 -0.030 (0)

CuCl4-2 1.165e-31 8.880e-32 -30.934 -31.052 -0.118 (0)

H(0) 3.154e-26

H2 1.577e-26 1.579e-26 -25.802 -25.802 0.000 (0)

K 4.512e-05

K+ 4.509e-05 4.208e-05 -4.346 -4.376 -0.030 (0)

KSO4- 3.059e-08 2.860e-08 -7.514 -7.544 -0.029 (0)

KCrO4- 4.602e-23 4.268e-23 -22.337 -22.370 -0.033 (0)

Mg 1.929e-04

Mg+2 1.864e-04 1.414e-04 -3.730 -3.850 -0.120 (0)

MgHCO3+ 3.941e-06 3.677e-06 -5.404 -5.434 -0.030 (0)

MgSO4 2.381e-06 2.381e-06 -5.623 -5.623 0.000 (0)

MgCO3 1.981e-07 1.981e-07 -6.703 -6.703 0.000 (0)

MgOH+ 3.256e-09 3.048e-09 -8.487 -8.516 -0.029 (0)

N(5) 3.582e-04

NO3- 3.573e-04 3.334e-04 -3.447 -3.477 -0.030 (0)

CaNO3+ 8.847e-07 8.206e-07 -6.053 -6.086 -0.033 (0)

ZnNO3+ 3.987e-11 3.698e-11 -10.399 -10.432 -0.033 (0)

CdNO3+ 2.600e-11 2.412e-11 -10.585 -10.618 -0.033 (0)

CuNO3+ 3.023e-12 2.804e-12 -11.520 -11.552 -0.033 (0)

PbNO3+ 1.504e-12 1.395e-12 -11.823 -11.855 -0.033 (0)

Cd(NO3)2 2.573e-15 2.573e-15 -14.590 -14.590 0.000 (0)

Zn(NO3)2 2.230e-15 2.230e-15 -14.652 -14.652 0.000 (0)

Pb(NO3)2 9.493e-16 9.493e-16 -15.023 -15.023 0.000 (0)

CrNO3+2 2.284e-16 1.690e-16 -15.641 -15.772 -0.131 (0)

Cu(NO3)2 1.078e-16 1.078e-16 -15.967 -15.967 0.000 (0)

Na 2.113e-04

Na+ 2.108e-04 1.967e-04 -3.676 -3.706 -0.030 (0)

NaHCO3 4.107e-07 4.107e-07 -6.386 -6.386 0.000 (0)

NaSO4- 1.159e-07 1.084e-07 -6.936 -6.965 -0.029 (0)

NaCO3- 1.318e-08 1.232e-08 -7.880 -7.909 -0.029 (0)

NaCrO4- 2.877e-22 2.669e-22 -21.541 -21.574 -0.033 (0)

O(0) 0.000e+00

O2 0.000e+00 0.000e+00 -45.020 -45.019 0.000 (0)

Pb 3.019e-09

PbCO3 1.932e-09 1.932e-09 -8.714 -8.714 0.000 (0)

PbHCO3+ 4.793e-10 4.446e-10 -9.319 -9.352 -0.033 (0)

Pb+2 3.892e-10 2.952e-10 -9.410 -9.530 -0.120 (0)

PbOH+ 1.844e-10 1.710e-10 -9.734 -9.767 -0.033 (0)

Pb(CO3)2-2 1.639e-11 1.213e-11 -10.786 -10.916 -0.131 (0)

PbSO4 1.515e-11 1.515e-11 -10.820 -10.820 0.000 (0)

PbNO3+ 1.504e-12 1.395e-12 -11.823 -11.855 -0.033 (0)

Pb(OH)2 1.247e-12 1.247e-12 -11.904 -11.904 0.000 (0)

PbCl+ 3.850e-13 3.571e-13 -12.415 -12.447 -0.033 (0)

Pb(SO4)2-2 1.293e-14 9.566e-15 -13.889 -14.019 -0.131 (0)

Pb(NO3)2 9.493e-16 9.493e-16 -15.023 -15.023 0.000 (0)

Pb(OH)3- 3.102e-16 2.877e-16 -15.508 -15.541 -0.033 (0)

PbCl2 6.104e-17 6.104e-17 -16.214 -16.214 0.000 (0)

Pb2OH+3 1.575e-18 8.002e-19 -17.803 -18.097 -0.294 (0)

Pb(OH)4-2 2.196e-20 1.625e-20 -19.658 -19.789 -0.131 (0)

PbCl3- 1.276e-21 1.184e-21 -20.894 -20.927 -0.033 (0)

Pb3(OH)4+2 1.055e-24 7.811e-25 -23.977 -24.107 -0.131 (0)

PbCl4-2 2.385e-26 1.765e-26 -25.622 -25.753 -0.131 (0)

Pb4(OH)4+4 1.087e-29 3.261e-30 -28.964 -29.487 -0.523 (0)

S(6) 1.550e-04

SO4-2 1.382e-04 1.048e-04 -3.860 -3.980 -0.120 (0)

CaSO4 1.430e-05 1.430e-05 -4.845 -4.845 0.000 (0)

MgSO4 2.381e-06 2.381e-06 -5.623 -5.623 0.000 (0)

NaSO4- 1.159e-07 1.084e-07 -6.936 -6.965 -0.029 (0)

KSO4- 3.059e-08 2.860e-08 -7.514 -7.544 -0.029 (0)

ZnSO4 8.037e-10 8.037e-10 -9.095 -9.095 0.000 (0)

CrOHSO4 3.594e-10 3.594e-10 -9.444 -9.444 0.000 (0)

HSO4- 2.992e-10 2.793e-10 -9.524 -9.554 -0.030 (0)

CdSO4 2.979e-10 2.979e-10 -9.526 -9.526 0.000 (0)

CuSO4 4.856e-11 4.856e-11 -10.314 -10.314 0.000 (0)

PbSO4 1.515e-11 1.515e-11 -10.820 -10.820 0.000 (0)

CrSO4+ 6.217e-12 5.766e-12 -11.206 -11.239 -0.033 (0)

Zn(SO4)2-2 1.131e-12 8.374e-13 -11.946 -12.077 -0.131 (0)

Cd(SO4)2-2 6.851e-13 5.071e-13 -12.164 -12.295 -0.131 (0)

Pb(SO4)2-2 1.293e-14 9.566e-15 -13.889 -14.019 -0.131 (0)

Cr2(OH)2SO4+2 6.345e-16 4.696e-16 -15.198 -15.328 -0.131 (0)

Cr2(OH)2(SO4)2 2.923e-18 2.923e-18 -17.534 -17.534 0.000 (0)

CrO3SO4-2 7.260e-29 5.373e-29 -28.139 -28.270 -0.131 (0)

Zn 6.174e-08

Zn+2 5.277e-08 4.002e-08 -7.278 -7.398 -0.120 (0)

ZnCO3 5.013e-09 5.013e-09 -8.300 -8.300 0.000 (0)

ZnHCO3+ 2.766e-09 2.565e-09 -8.558 -8.591 -0.033 (0)

ZnSO4 8.037e-10 8.037e-10 -9.095 -9.095 0.000 (0)

ZnOH+ 3.020e-10 2.801e-10 -9.520 -9.553 -0.033 (0)

ZnNO3+ 3.987e-11 3.698e-11 -10.399 -10.432 -0.033 (0)

Zn(OH)2 3.375e-11 3.375e-11 -10.472 -10.472 0.000 (0)

ZnCl+ 3.942e-12 3.678e-12 -11.404 -11.434 -0.030 (0)

ZnOHCl 1.247e-12 1.247e-12 -11.904 -11.904 0.000 (0)

Zn(SO4)2-2 1.131e-12 8.374e-13 -11.946 -12.077 -0.131 (0)

Zn(OH)3- 4.206e-14 3.901e-14 -13.376 -13.409 -0.033 (0)

Zn(NO3)2 2.230e-15 2.230e-15 -14.652 -14.652 0.000 (0)

ZnCl2 1.218e-16 1.218e-16 -15.914 -15.914 0.000 (0)

Zn(OH)4-2 4.840e-19 3.582e-19 -18.315 -18.446 -0.131 (0)

ZnCl3- 3.995e-21 3.728e-21 -20.398 -20.429 -0.030 (0)

ZnCl4-2 8.863e-26 6.753e-26 -25.052 -25.170 -0.118 (0)

------------------------------Saturation indices-------------------------------

Phase SI** log IAP log K(283 K, 1 atm)

Anglesite -5.61 -13.51 -7.90 PbSO4

Anhydrite -2.85 -7.14 -4.29 CaSO4

Antlerite -9.17 -0.38 8.79 Cu3(OH)4SO4

Aragonite -0.63 -8.82 -8.19 CaCO3

Arsenolite -60.77 -64.09 -3.32 As4O6

Artinite -9.36 1.36 10.72 MgCO3:Mg(OH)2:3H2O

As2O5 -33.65 -26.73 6.92 As2O5

Atacamite -7.79 0.47 8.26 Cu2(OH)3Cl

Azurite -6.42 -22.44 -16.02 Cu3(OH)2(CO3)2

Bianchite -9.62 -11.38 -1.76 ZnSO4:6H2O

Brochantite -11.38 5.73 17.10 Cu4(OH)6SO4

Brucite -7.03 10.87 17.90 Mg(OH)2

Ca3(AsO4)2:4H2O -14.35 7.95 22.30 Ca3(AsO4)2:4H2O

CaCrO4 -19.71 -21.72 -2.02 CaCrO4

Calcite -0.42 -8.82 -8.41 CaCO3

Cd(OH)2 -7.64 6.88 14.52 Cd(OH)2

Cd(OH)2(am) -7.65 6.88 14.54 Cd(OH)2

Cd3(OH)2(SO4)2 -23.46 -16.75 6.71 Cd3(OH)2(SO4)2

Cd3(OH)4SO4 -20.61 1.95 22.56 Cd3(OH)4SO4

Cd4(OH)6SO4 -19.56 8.84 28.40 Cd4(OH)6SO4

CdCl2 -16.12 -16.61 -0.49 CdCl2

CdCl2:1H2O -14.98 -16.61 -1.62 CdCl2:1H2O

CdCl2:2.5H2O -14.63 -16.61 -1.98 CdCl2:2.5H2O

Cdmetal(alpha) -30.05 -15.84 14.21 Cd

Cdmetal(gamma) -30.16 -15.84 14.32 Cd

CdOHCl -8.69 -4.86 3.82 CdOHCl

CdSO4 -12.13 -11.82 0.31 CdSO4

CdSO4:1H2O -10.38 -11.82 -1.43 CdSO4:1H2O

CdSO4:2.67H2O -10.11 -11.82 -1.71 CdSO4:2.67H2O

Cerussite -1.83 -15.19 -13.36 PbCO3

CH4(g) -67.83 -111.26 -43.43 CH4

Chalcanthite -9.90 -12.59 -2.70 CuSO4:5H2O

Claudetite -60.51 -64.09 -3.58 As4O6

CO2(g) -2.20 -20.38 -18.18 CO2

Cotunnite -13.28 -18.30 -5.02 PbCl2

Cr(OH)2 -18.65 -7.50 11.15 Cr(OH)2

Cr(OH)3 -0.64 0.97 1.61 Cr(OH)3

Cr(OH)3(am) 1.72 0.97 -0.75 Cr(OH)3

Cr2O3 3.82 1.94 -1.89 Cr2O3

CrCl2 -46.12 -31.00 15.12 CrCl2

CrCl3 -50.51 -34.27 16.24 CrCl3

Crmetal -62.30 -30.22 32.08 Cr

CrO3 -30.12 -33.28 -3.16 CrO3

Cu(OH)2 -3.09 6.11 9.20 Cu(OH)2

Cu2(OH)3NO3 -8.55 1.38 9.92 Cu2(OH)3NO3

Cu2SO4 -22.18 -23.95 -1.77 Cu2SO4

Cu3(AsO4)2:2H2O -14.51 -8.41 6.10 Cu3(AsO4)2:2H2O

CuCO3 -2.78 -14.28 -11.50 CuCO3

CuCrO4 -21.74 -27.18 -5.44 CuCrO4

Cumetal -4.57 -13.99 -9.42 Cu

CuOCuSO4 -18.07 -6.49 11.58 CuOCuSO4

Cuprite -5.00 -5.25 -0.26 Cu2O

CuSO4 -16.21 -12.59 3.62 CuSO4

Dolomite(disordered) -2.22 -18.33 -16.11 CaMg(CO3)2

Dolomite(ordered) -1.61 -18.33 -16.72 CaMg(CO3)2

Epsomite -5.60 -7.83 -2.23 MgSO4:7H2O

Goslarite -9.23 -11.38 -2.14 ZnSO4:7H2O

Gypsum -2.52 -7.14 -4.62 CaSO4:2H2O

Halite -9.66 -8.09 1.57 NaCl

Huntite -8.39 -37.36 -28.97 CaMg3(CO3)4

Hydrocerussite -6.42 -25.19 -18.77 Pb3(OH)2(CO3)2

Hydromagnesite -20.44 -27.18 -6.74 Mg5(CO3)4(OH)2:4H2O

K2Cr2O7 -42.61 -60.60 -17.99 K2Cr2O7

K2CrO4 -26.63 -27.32 -0.68 K2CrO4

Langite -13.30 5.73 19.03 Cu4(OH)6SO4:H2O

Larnakite -8.09 -8.32 -0.23 PbO:PbSO4

Laurionite -7.18 -6.56 0.62 PbOHCl

Lime -22.94 11.56 34.50 CaO

Litharge -8.11 5.19 13.30 PbO

Magnesite -1.87 -9.51 -7.65 MgCO3

Malachite -2.15 -8.17 -6.01 Cu2(OH)2CO3

Massicot -8.32 5.19 13.51 PbO

Melanothallite -24.23 -17.39 6.85 CuCl2

Mg(OH)2(active) -7.92 10.87 18.79 Mg(OH)2

MgCr2O4 -5.06 12.81 17.87 MgCr2O4

MgCrO4 -28.62 -22.41 6.21 MgCrO4

Minium -39.15 38.29 77.44 Pb3O4

Mirabilite -9.54 -11.39 -1.85 Na2SO4:10H2O

Monteponite -9.18 6.88 16.06 CdO

Na2Cr2O7 -49.16 -59.26 -10.10 Na2Cr2O7

Na2CrO4 -29.09 -25.98 3.11 Na2CrO4

Nantokite -7.25 -14.37 -7.13 CuCl

Natron -11.15 -13.07 -1.92 Na2CO3:10H2O

Nesquehonite -5.07 -9.51 -4.45 MgCO3:3H2O

O2(g) -42.95 45.44 88.39 O2

Otavite -1.50 -13.50 -11.99 CdCO3

Pb(OH)2 -3.50 5.19 8.69 Pb(OH)2

Pb10(OH)6O(CO3)6 -61.63 -70.39 -8.76 Pb10(OH)6O(CO3)6

Pb2(OH)3Cl -10.16 -1.37 8.79 Pb2(OH)3Cl

Pb2O(OH)2 -15.81 10.38 26.19 Pb2O(OH)2

Pb2O3 -27.94 33.10 61.04 Pb2O3

Pb2OCO3 -9.82 -10.00 -0.18 Pb2OCO3

Pb3(AsO4)2 -16.96 -11.16 5.80 Pb3(AsO4)2

Pb3O2CO3 -16.86 -4.81 12.05 Pb3O2CO3

Pb3O2SO4 -14.55 -3.13 11.42 Pb3O2SO4

Pb4(OH)6SO4 -19.04 2.06 21.10 Pb4(OH)6SO4

Pb4O3SO4 -21.08 2.06 23.14 Pb4O3SO4

PbCrO4 -15.08 -28.09 -13.01 PbCrO4

Pbmetal -21.77 -17.53 4.24 Pb

PbO:0.3H2O -7.79 5.19 12.98 PbO:0.33H2O

Periclase -12.12 10.87 22.99 MgO

Phosgenite -13.69 -33.50 -19.81 PbCl2:PbCO3

Plattnerite -24.44 27.91 52.35 PbO2

Portlandite -12.44 11.56 24.00 Ca(OH)2

Smithsonite -3.21 -13.06 -9.85 ZnCO3

Tenorite -2.14 6.11 8.25 CuO

Thenardite -11.80 -11.39 0.41 Na2SO4

Thermonatrite -13.81 -13.07 0.73 Na2CO3:H2O

Zincite -4.84 7.32 12.17 ZnO

Zincosite -16.07 -11.38 4.70 ZnSO4

Zn(NO3)2:6H2O -17.44 -14.35 3.09 Zn(NO3)2:6H2O

Zn(OH)2 -4.88 7.32 12.20 Zn(OH)2

Zn(OH)2(am) -5.90 7.32 13.22 Zn(OH)2

Zn(OH)2(beta) -5.20 7.32 12.53 Zn(OH)2

Zn(OH)2(epsilon) -4.97 7.32 12.29 Zn(OH)2

Zn(OH)2(gamma) -4.41 7.32 11.73 Zn(OH)2

Zn2(OH)2SO4 -11.56 -4.06 7.50 Zn2(OH)2SO4

Zn2(OH)3Cl -12.29 2.90 15.19 Zn2(OH)3Cl

Zn3(AsO4)2:2.5H2O -18.41 -4.76 13.65 Zn3(AsO4)2:2.5H2O

Zn3O(SO4)2 -36.74 -15.43 21.31 Zn3O(SO4)2

Zn4(OH)6SO4 -17.81 10.59 28.40 Zn4(OH)6SO4

Zn5(OH)8Cl2 -25.38 13.12 38.50 Zn5(OH)8Cl2

ZnCl2 -23.89 -16.17 7.72 ZnCl2

ZnCO3:1H2O -2.80 -13.06 -10.26 ZnCO3:1H2O

Znmetal -42.61 -15.40 27.21 Zn

ZnO(active) -4.69 7.32 12.01 ZnO

ZnSO4:1H2O -11.15 -11.38 -0.23 ZnSO4:1H2O

**For a gas, SI = log10(fugacity). Fugacity = pressure * phi / 1 atm.

For ideal gases, phi = 1.

Initial solution 7.

----------------------------Distribution of species----------------------------

Log Log Log mole V

Species Molality Activity Molality Activity Gamma cm?mol

OH- 6.920e-08 6.532e-08 -7.160 -7.185 -0.025 (0)

H+ 4.953e-08 4.677e-08 -7.305 -7.330 -0.025 0.00

H2O 5.551e+01 1.000e+00 1.744 -0.000 0.000 18.02

As(3) 1.836e-16

H3AsO3 1.824e-16 1.824e-16 -15.739 -15.739 0.000 (0)

H2AsO3- 1.184e-18 1.114e-18 -17.927 -17.953 -0.027 (0)

HAsO3-2 1.400e-23 1.096e-23 -22.854 -22.960 -0.106 (0)

H4AsO3+ 4.495e-24 4.228e-24 -23.347 -23.374 -0.027 (0)

AsO3-3 9.128e-30 5.258e-30 -29.040 -29.279 -0.240 (0)

As(5) 3.994e-08

HAsO4-2 2.897e-08 2.267e-08 -7.538 -7.645 -0.106 (0)

H2AsO4- 1.096e-08 1.031e-08 -7.960 -7.987 -0.027 (0)

AsO4-3 1.851e-12 1.066e-12 -11.733 -11.972 -0.240 (0)

H3AsO4 7.197e-14 7.201e-14 -13.143 -13.143 0.000 (0)

C(4) 1.422e-03

HCO3- 1.259e-03 1.190e-03 -2.900 -2.924 -0.024 (0)

H2CO3 1.523e-04 1.523e-04 -3.817 -3.817 0.000 (0)

CaHCO3+ 8.271e-06 7.825e-06 -5.082 -5.107 -0.024 (0)

CO3-2 1.098e-06 8.733e-07 -5.959 -6.059 -0.099 (0)

MgHCO3+ 9.468e-07 8.940e-07 -6.024 -6.049 -0.025 (0)

CaCO3 5.322e-07 5.322e-07 -6.274 -6.274 0.000 (0)

NaHCO3 1.162e-07 1.162e-07 -6.935 -6.935 0.000 (0)

MgCO3 4.494e-08 4.494e-08 -7.347 -7.347 0.000 (0)

CuCO3 9.926e-09 9.926e-09 -8.003 -8.003 0.000 (0)

NaCO3- 3.440e-09 3.253e-09 -8.463 -8.488 -0.024 (0)

ZnCO3 1.729e-09 1.729e-09 -8.762 -8.762 0.000 (0)

ZnHCO3+ 1.008e-09 9.477e-10 -8.997 -9.023 -0.027 (0)

PbCO3 8.064e-10 8.064e-10 -9.093 -9.093 0.000 (0)

PbHCO3+ 2.114e-10 1.989e-10 -9.675 -9.701 -0.027 (0)

CuHCO3+ 1.128e-10 1.061e-10 -9.948 -9.974 -0.027 (0)

Cu(CO3)2-2 2.981e-11 2.333e-11 -10.526 -10.632 -0.106 (0)

CdCO3 8.855e-12 8.855e-12 -11.053 -11.053 0.000 (0)

Pb(CO3)2-2 2.595e-12 2.031e-12 -11.586 -11.692 -0.106 (0)

CdHCO3+ 9.382e-13 8.824e-13 -12.028 -12.054 -0.027 (0)

Cd(CO3)2-2 7.325e-15 5.732e-15 -14.135 -14.242 -0.106 (0)

Ca 7.074e-04

Ca+2 6.805e-04 5.413e-04 -3.167 -3.267 -0.099 (0)

CaSO4 1.764e-05 1.764e-05 -4.753 -4.753 0.000 (0)

CaHCO3+ 8.271e-06 7.825e-06 -5.082 -5.107 -0.024 (0)

CaNO3+ 5.350e-07 5.032e-07 -6.272 -6.298 -0.027 (0)

CaCO3 5.322e-07 5.322e-07 -6.274 -6.274 0.000 (0)

CaOH+ 6.245e-10 5.908e-10 -9.204 -9.229 -0.024 (0)

Cd 5.859e-10

Cd+2 5.592e-10 4.448e-10 -9.252 -9.352 -0.099 (0)

CdSO4 1.434e-11 1.434e-11 -10.843 -10.843 0.000 (0)

CdCO3 8.855e-12 8.855e-12 -11.053 -11.053 0.000 (0)

CdCl+ 1.626e-12 1.530e-12 -11.789 -11.815 -0.027 (0)

CdHCO3+ 9.382e-13 8.824e-13 -12.028 -12.054 -0.027 (0)

CdNO3+ 6.137e-13 5.772e-13 -12.212 -12.239 -0.027 (0)

CdOH+ 2.507e-13 2.358e-13 -12.601 -12.627 -0.027 (0)

Cd(SO4)2-2 4.931e-14 3.858e-14 -13.307 -13.414 -0.106 (0)

CdOHCl 9.349e-15 9.349e-15 -14.029 -14.029 0.000 (0)

Cd(CO3)2-2 7.325e-15 5.732e-15 -14.135 -14.242 -0.106 (0)

Cd(OH)2 1.033e-15 1.033e-15 -14.986 -14.986 0.000 (0)

CdCl2 2.248e-16 2.248e-16 -15.648 -15.648 0.000 (0)

Cd(NO3)2 4.838e-17 4.838e-17 -16.315 -16.315 0.000 (0)

Cd(OH)3- 1.445e-20 1.359e-20 -19.840 -19.867 -0.027 (0)

CdCl3- 4.777e-21 4.492e-21 -20.321 -20.348 -0.027 (0)

Cd2OH+3 1.106e-21 6.372e-22 -20.956 -21.196 -0.240 (0)

Cd(OH)4-2 6.118e-28 4.788e-28 -27.213 -27.320 -0.106 (0)

Cl 3.895e-05

Cl- 3.895e-05 3.679e-05 -4.409 -4.434 -0.025 (0)

CuCl 3.778e-12 3.778e-12 -11.423 -11.423 0.000 (0)

ZnCl+ 2.999e-12 2.832e-12 -11.523 -11.548 -0.025 (0)

CdCl+ 1.626e-12 1.530e-12 -11.789 -11.815 -0.027 (0)

ZnOHCl 8.958e-13 8.958e-13 -12.048 -12.048 0.000 (0)

PbCl+ 3.540e-13 3.329e-13 -12.451 -12.478 -0.027 (0)

CuCl+ 9.981e-14 9.425e-14 -13.001 -13.026 -0.025 (0)

CuCl2- 3.193e-14 3.015e-14 -13.496 -13.521 -0.025 (0)

CdOHCl 9.349e-15 9.349e-15 -14.029 -14.029 0.000 (0)

CrCl+2 2.472e-15 1.934e-15 -14.607 -14.713 -0.106 (0)

CdCl2 2.248e-16 2.248e-16 -15.648 -15.648 0.000 (0)

ZnCl2 8.405e-17 8.405e-17 -16.075 -16.075 0.000 (0)

PbCl2 5.098e-17 5.098e-17 -16.293 -16.293 0.000 (0)

CuCl2 5.584e-19 5.584e-19 -18.253 -18.253 0.000 (0)

CuCl3-2 2.796e-19 2.231e-19 -18.553 -18.651 -0.098 (0)

CrOHCl2 3.180e-20 3.180e-20 -19.498 -19.498 0.000 (0)

CdCl3- 4.777e-21 4.492e-21 -20.321 -20.348 -0.027 (0)

ZnCl3- 2.439e-21 2.304e-21 -20.613 -20.638 -0.025 (0)

CrCl2+ 1.813e-21 1.705e-21 -20.742 -20.768 -0.027 (0)

PbCl3- 9.419e-22 8.858e-22 -21.026 -21.053 -0.027 (0)

CuCl3- 1.535e-25 1.449e-25 -24.814 -24.839 -0.025 (0)

ZnCl4-2 4.685e-26 3.739e-26 -25.329 -25.427 -0.098 (0)

PbCl4-2 1.512e-26 1.183e-26 -25.820 -25.927 -0.106 (0)

CrO3Cl- 4.239e-31 3.987e-31 -30.373 -30.399 -0.027 (0)

CuCl4-2 5.679e-32 4.532e-32 -31.246 -31.344 -0.098 (0)

Cr(2) 1.183e-22

Cr+2 1.183e-22 9.254e-23 -21.927 -22.034 -0.106 (0)

Cr(3) 7.693e-07

Cr(OH)2+ 5.820e-07 5.474e-07 -6.235 -6.262 -0.027 (0)

Cr(OH)+2 1.412e-07 1.105e-07 -6.850 -6.957 -0.106 (0)

Cr(OH)3 4.427e-08 4.427e-08 -7.354 -7.354 0.000 (0)

CrOHSO4 8.214e-10 8.214e-10 -9.085 -9.085 0.000 (0)

CrO2- 4.779e-10 4.495e-10 -9.321 -9.347 -0.027 (0)

Cr(OH)4- 4.034e-10 3.794e-10 -9.394 -9.421 -0.027 (0)

Cr+3 1.230e-10 7.081e-11 -9.910 -10.150 -0.240 (0)

CrSO4+ 1.501e-11 1.412e-11 -10.824 -10.850 -0.027 (0)

CrCl+2 2.472e-15 1.934e-15 -14.607 -14.713 -0.106 (0)

Cr2(OH)2SO4+2 1.983e-15 1.552e-15 -14.703 -14.809 -0.106 (0)

CrNO3+2 2.629e-16 2.057e-16 -15.580 -15.687 -0.106 (0)

Cr2(OH)2(SO4)2 1.526e-17 1.526e-17 -16.816 -16.816 0.000 (0)

CrOHCl2 3.180e-20 3.180e-20 -19.498 -19.498 0.000 (0)

CrCl2+ 1.813e-21 1.705e-21 -20.742 -20.768 -0.027 (0)

Cr(6) 3.438e-19

CrO4-2 3.060e-19 2.434e-19 -18.514 -18.614 -0.099 (0)

HCrO4- 3.753e-20 3.530e-20 -19.426 -19.452 -0.027 (0)

NaCrO4- 1.665e-22 1.566e-22 -21.778 -21.805 -0.027 (0)

KCrO4- 3.155e-23 2.968e-23 -22.501 -22.528 -0.027 (0)

H2CrO4 6.071e-28 6.071e-28 -27.217 -27.217 0.000 (0)

CrO3SO4-2 1.111e-28 8.693e-29 -27.954 -28.061 -0.106 (0)

CrO3Cl- 4.239e-31 3.987e-31 -30.373 -30.399 -0.027 (0)

Cr2O7-2 8.288e-38 6.486e-38 -37.082 -37.188 -0.106 (0)

Cu(1) 9.055e-11

Cu+ 8.674e-11 8.158e-11 -10.062 -10.088 -0.027 (0)

CuCl 3.778e-12 3.778e-12 -11.423 -11.423 0.000 (0)

CuCl2- 3.193e-14 3.015e-14 -13.496 -13.521 -0.025 (0)

CuCl3-2 2.796e-19 2.231e-19 -18.553 -18.651 -0.098 (0)

Cu(2) 1.326e-08

CuCO3 9.926e-09 9.926e-09 -8.003 -8.003 0.000 (0)

Cu+2 2.427e-09 1.930e-09 -8.615 -8.714 -0.099 (0)

CuOH+ 6.474e-10 6.113e-10 -9.189 -9.214 -0.025 (0)

CuHCO3+ 1.128e-10 1.061e-10 -9.948 -9.974 -0.027 (0)

CuSO4 6.081e-11 6.081e-11 -10.216 -10.216 0.000 (0)

Cu(OH)2 5.644e-11 5.644e-11 -10.248 -10.248 0.000 (0)

Cu(CO3)2-2 2.981e-11 2.333e-11 -10.526 -10.632 -0.106 (0)

CuNO3+ 1.856e-12 1.745e-12 -11.731 -11.758 -0.027 (0)

CuCl+ 9.981e-14 9.425e-14 -13.001 -13.026 -0.025 (0)

Cu(OH)3- 2.650e-14 2.492e-14 -13.577 -13.603 -0.027 (0)

Cu2(OH)2+2 1.078e-14 8.435e-15 -13.967 -14.074 -0.106 (0)

Cu(NO3)2 5.273e-17 5.273e-17 -16.278 -16.278 0.000 (0)

CuCl2 5.584e-19 5.584e-19 -18.253 -18.253 0.000 (0)

Cu(OH)4-2 5.395e-20 4.222e-20 -19.268 -19.374 -0.106 (0)

CuCl3- 1.535e-25 1.449e-25 -24.814 -24.839 -0.025 (0)

CuCl4-2 5.679e-32 4.532e-32 -31.246 -31.344 -0.098 (0)

H(0) 3.623e-26

H2 1.811e-26 1.813e-26 -25.742 -25.742 0.000 (0)

K 3.478e-05

K+ 3.474e-05 3.281e-05 -4.459 -4.484 -0.025 (0)

KSO4- 3.727e-08 3.524e-08 -7.429 -7.453 -0.024 (0)

KCrO4- 3.155e-23 2.968e-23 -22.501 -22.528 -0.027 (0)

Mg 1.036e-04

Mg+2 1.005e-04 7.995e-05 -3.998 -4.097 -0.099 (0)

MgSO4 2.129e-06 2.129e-06 -5.672 -5.672 0.000 (0)

MgHCO3+ 9.468e-07 8.940e-07 -6.024 -6.049 -0.025 (0)

MgCO3 4.494e-08 4.494e-08 -7.347 -7.347 0.000 (0)

MgOH+ 1.700e-09 1.609e-09 -8.770 -8.794 -0.024 (0)

N(5) 2.779e-04

NO3- 2.774e-04 2.620e-04 -3.557 -3.582 -0.025 (0)

CaNO3+ 5.350e-07 5.032e-07 -6.272 -6.298 -0.027 (0)

ZnNO3+ 2.655e-11 2.497e-11 -10.576 -10.603 -0.027 (0)

CuNO3+ 1.856e-12 1.745e-12 -11.731 -11.758 -0.027 (0)

PbNO3+ 1.213e-12 1.140e-12 -11.916 -11.943 -0.027 (0)

CdNO3+ 6.137e-13 5.772e-13 -12.212 -12.239 -0.027 (0)

Zn(NO3)2 1.183e-15 1.183e-15 -14.927 -14.927 0.000 (0)

Pb(NO3)2 6.097e-16 6.097e-16 -15.215 -15.215 0.000 (0)

CrNO3+2 2.629e-16 2.057e-16 -15.580 -15.687 -0.106 (0)

Cu(NO3)2 5.273e-17 5.273e-17 -16.278 -16.278 0.000 (0)

Cd(NO3)2 4.838e-17 4.838e-17 -16.315 -16.315 0.000 (0)

Na 1.374e-04

Na+ 1.371e-04 1.295e-04 -3.863 -3.888 -0.025 (0)

NaSO4- 1.192e-07 1.127e-07 -6.924 -6.948 -0.024 (0)

NaHCO3 1.162e-07 1.162e-07 -6.935 -6.935 0.000 (0)

NaCO3- 3.440e-09 3.253e-09 -8.463 -8.488 -0.024 (0)

NaCrO4- 1.665e-22 1.566e-22 -21.778 -21.805 -0.027 (0)

O(0) 0.000e+00

O2 0.000e+00 0.000e+00 -45.139 -45.139 0.000 (0)

Pb 1.611e-09

PbCO3 8.064e-10 8.064e-10 -9.093 -9.093 0.000 (0)

Pb+2 3.862e-10 3.072e-10 -9.413 -9.513 -0.099 (0)

PbHCO3+ 2.114e-10 1.989e-10 -9.675 -9.701 -0.027 (0)

PbOH+ 1.766e-10 1.661e-10 -9.753 -9.780 -0.027 (0)

PbSO4 2.492e-11 2.492e-11 -10.604 -10.604 0.000 (0)

Pb(CO3)2-2 2.595e-12 2.031e-12 -11.586 -11.692 -0.106 (0)

PbNO3+ 1.213e-12 1.140e-12 -11.916 -11.943 -0.027 (0)

Pb(OH)2 1.131e-12 1.131e-12 -11.947 -11.947 0.000 (0)

PbCl+ 3.540e-13 3.329e-13 -12.451 -12.478 -0.027 (0)

Pb(SO4)2-2 3.177e-14 2.486e-14 -13.498 -13.604 -0.106 (0)

Pb(NO3)2 6.097e-16 6.097e-16 -15.215 -15.215 0.000 (0)

Pb(OH)3- 2.588e-16 2.434e-16 -15.587 -15.614 -0.027 (0)

PbCl2 5.098e-17 5.098e-17 -16.293 -16.293 0.000 (0)

Pb2OH+3 1.404e-18 8.087e-19 -17.853 -18.092 -0.240 (0)

Pb(OH)4-2 1.640e-20 1.283e-20 -19.785 -19.892 -0.106 (0)

PbCl3- 9.419e-22 8.858e-22 -21.026 -21.053 -0.027 (0)

Pb3(OH)4+2 8.533e-25 6.677e-25 -24.069 -24.175 -0.106 (0)

PbCl4-2 1.512e-26 1.183e-26 -25.820 -25.927 -0.106 (0)

Pb4(OH)4+4 7.737e-30 2.901e-30 -29.111 -29.537 -0.426 (0)

S(6) 2.281e-04

SO4-2 2.082e-04 1.656e-04 -3.682 -3.781 -0.099 (0)

CaSO4 1.764e-05 1.764e-05 -4.753 -4.753 0.000 (0)

MgSO4 2.129e-06 2.129e-06 -5.672 -5.672 0.000 (0)

NaSO4- 1.192e-07 1.127e-07 -6.924 -6.948 -0.024 (0)

KSO4- 3.727e-08 3.524e-08 -7.429 -7.453 -0.024 (0)

ZnSO4 1.092e-09 1.092e-09 -8.962 -8.962 0.000 (0)

CrOHSO4 8.214e-10 8.214e-10 -9.085 -9.085 0.000 (0)

HSO4- 5.007e-10 4.731e-10 -9.300 -9.325 -0.025 (0)

CuSO4 6.081e-11 6.081e-11 -10.216 -10.216 0.000 (0)

PbSO4 2.492e-11 2.492e-11 -10.604 -10.604 0.000 (0)

CrSO4+ 1.501e-11 1.412e-11 -10.824 -10.850 -0.027 (0)

CdSO4 1.434e-11 1.434e-11 -10.843 -10.843 0.000 (0)

Zn(SO4)2-2 2.297e-12 1.798e-12 -11.639 -11.745 -0.106 (0)

Cd(SO4)2-2 4.931e-14 3.858e-14 -13.307 -13.414 -0.106 (0)

Pb(SO4)2-2 3.177e-14 2.486e-14 -13.498 -13.604 -0.106 (0)

Cr2(OH)2SO4+2 1.983e-15 1.552e-15 -14.703 -14.809 -0.106 (0)

Cr2(OH)2(SO4)2 1.526e-17 1.526e-17 -16.816 -16.816 0.000 (0)

CrO3SO4-2 1.111e-28 8.693e-29 -27.954 -28.061 -0.106 (0)

Zn 4.737e-08

Zn+2 4.324e-08 3.440e-08 -7.364 -7.463 -0.099 (0)

ZnCO3 1.729e-09 1.729e-09 -8.762 -8.762 0.000 (0)

ZnSO4 1.092e-09 1.092e-09 -8.962 -8.962 0.000 (0)

ZnHCO3+ 1.008e-09 9.477e-10 -8.997 -9.023 -0.027 (0)

ZnOH+ 2.389e-10 2.247e-10 -9.622 -9.648 -0.027 (0)

ZnNO3+ 2.655e-11 2.497e-11 -10.576 -10.603 -0.027 (0)

Zn(OH)2 2.526e-11 2.526e-11 -10.598 -10.598 0.000 (0)

ZnCl+ 2.999e-12 2.832e-12 -11.523 -11.548 -0.025 (0)

Zn(SO4)2-2 2.297e-12 1.798e-12 -11.639 -11.745 -0.106 (0)

ZnOHCl 8.958e-13 8.958e-13 -12.048 -12.048 0.000 (0)

Zn(OH)3- 2.898e-14 2.726e-14 -13.538 -13.565 -0.027 (0)

Zn(NO3)2 1.183e-15 1.183e-15 -14.927 -14.927 0.000 (0)

ZnCl2 8.405e-17 8.405e-17 -16.075 -16.075 0.000 (0)

Zn(OH)4-2 2.985e-19 2.336e-19 -18.525 -18.632 -0.106 (0)

ZnCl3- 2.439e-21 2.304e-21 -20.613 -20.638 -0.025 (0)

ZnCl4-2 4.685e-26 3.739e-26 -25.329 -25.427 -0.098 (0)

------------------------------Saturation indices-------------------------------

Phase SI** log IAP log K(283 K, 1 atm)

Anglesite -5.39 -13.29 -7.90 PbSO4

Anhydrite -2.75 -7.05 -4.29 CaSO4

Antlerite -9.39 -0.60 8.79 Cu3(OH)4SO4

Aragonite -1.14 -9.33 -8.19 CaCO3

Arsenolite -59.64 -62.96 -3.32 As4O6

Artinite -10.31 0.41 10.72 MgCO3:Mg(OH)2:3H2O

As2O5 -33.20 -26.29 6.92 As2O5

Atacamite -8.13 0.13 8.26 Cu2(OH)3Cl

Azurite -7.58 -23.60 -16.02 Cu3(OH)2(CO3)2

Bianchite -9.49 -11.24 -1.76 ZnSO4:6H2O

Brochantite -11.76 5.34 17.10 Cu4(OH)6SO4

Brucite -7.34 10.56 17.90 Mg(OH)2

Ca3(AsO4)2:4H2O -14.40 7.90 22.30 Ca3(AsO4)2:4H2O

CaCrO4 -19.86 -21.88 -2.02 CaCrO4

Calcite -0.92 -9.33 -8.41 CaCO3

Cd(OH)2 -9.21 5.31 14.52 Cd(OH)2

Cd(OH)2(am) -9.23 5.31 14.54 Cd(OH)2

Cd3(OH)2(SO4)2 -27.67 -20.96 6.71 Cd3(OH)2(SO4)2

Cd3(OH)4SO4 -25.08 -2.52 22.56 Cd3(OH)4SO4

Cd4(OH)6SO4 -25.61 2.79 28.40 Cd4(OH)6SO4

CdCl2 -17.73 -18.22 -0.49 CdCl2

CdCl2:1H2O -16.60 -18.22 -1.62 CdCl2:1H2O

CdCl2:2.5H2O -16.24 -18.22 -1.98 CdCl2:2.5H2O

Cdmetal(alpha) -31.57 -17.35 14.21 Cd

Cdmetal(gamma) -31.67 -17.35 14.32 Cd

CdOHCl -10.28 -6.46 3.82 CdOHCl

CdSO4 -13.44 -13.13 0.31 CdSO4

CdSO4:1H2O -11.70 -13.13 -1.43 CdSO4:1H2O

CdSO4:2.67H2O -11.43 -13.13 -1.71 CdSO4:2.67H2O

Cerussite -2.21 -15.57 -13.36 PbCO3

CH4(g) -67.93 -111.36 -43.43 CH4

Chalcanthite -9.80 -12.50 -2.70 CuSO4:5H2O

Claudetite -59.37 -62.96 -3.58 As4O6

CO2(g) -2.53 -20.72 -18.18 CO2

Cotunnite -13.36 -18.38 -5.02 PbCl2

Cr(OH)2 -18.52 -7.37 11.15 Cr(OH)2

Cr(OH)3 -0.54 1.07 1.61 Cr(OH)3

Cr(OH)3(am) 1.82 1.07 -0.75 Cr(OH)3

Cr2O3 4.02 2.14 -1.89 Cr2O3

CrCl2 -46.02 -30.90 15.12 CrCl2

CrCl3 -50.46 -34.22 16.24 CrCl3

Crmetal -62.11 -30.03 32.08 Cr

CrO3 -30.11 -33.27 -3.16 CrO3

Cu(OH)2 -3.25 5.95 9.20 Cu(OH)2

Cu2(OH)3NO3 -8.95 0.98 9.92 Cu2(OH)3NO3

Cu2SO4 -22.18 -23.96 -1.77 Cu2SO4

Cu3(AsO4)2:2H2O -14.55 -8.45 6.10 Cu3(AsO4)2:2H2O

CuCO3 -3.27 -14.77 -11.50 CuCO3

CuCrO4 -21.89 -27.33 -5.44 CuCrO4

Cumetal -4.67 -14.09 -9.42 Cu

CuOCuSO4 -18.13 -6.55 11.58 CuOCuSO4

Cuprite -5.26 -5.52 -0.26 Cu2O

CuSO4 -16.11 -12.50 3.62 CuSO4

Dolomite(disordered) -3.37 -19.48 -16.11 CaMg(CO3)2

Dolomite(ordered) -2.76 -19.48 -16.72 CaMg(CO3)2

Epsomite -5.64 -7.88 -2.23 MgSO4:7H2O

Goslarite -9.10 -11.24 -2.14 ZnSO4:7H2O

Gypsum -2.43 -7.05 -4.62 CaSO4:2H2O

Halite -9.89 -8.32 1.57 NaCl

Huntite -10.83 -39.79 -28.97 CaMg3(CO3)4

Hydrocerussite -7.23 -26.00 -18.77 Pb3(OH)2(CO3)2

Hydromagnesite -23.32 -30.06 -6.74 Mg5(CO3)4(OH)2:4H2O

K2Cr2O7 -42.86 -60.86 -17.99 K2Cr2O7

K2CrO4 -26.90 -27.58 -0.68 K2CrO4

Langite -13.68 5.34 19.03 Cu4(OH)6SO4:H2O

Larnakite -7.91 -8.15 -0.23 PbO:PbSO4

Laurionite -7.24 -6.62 0.62 PbOHCl

Lime -23.11 11.39 34.50 CaO

Litharge -8.15 5.15 13.30 PbO

Magnesite -2.51 -10.16 -7.65 MgCO3

Malachite -2.81 -8.83 -6.01 Cu2(OH)2CO3

Massicot -8.37 5.15 13.51 PbO

Melanothallite -24.43 -17.58 6.85 CuCl2

Mg(OH)2(active) -8.23 10.56 18.79 Mg(OH)2

MgCr2O4 -5.17 12.70 17.87 MgCr2O4

MgCrO4 -28.92 -22.71 6.21 MgCrO4

Minium -39.34 38.10 77.44 Pb3O4

Mirabilite -9.71 -11.56 -1.85 Na2SO4:10H2O

Monteponite -10.75 5.31 16.06 CdO

Na2Cr2O7 -49.56 -59.66 -10.10 Na2Cr2O7

Na2CrO4 -29.50 -26.39 3.11 Na2CrO4

Nantokite -7.40 -14.52 -7.13 CuCl

Natron -11.91 -13.83 -1.92 Na2CO3:10H2O

Nesquehonite -5.71 -10.16 -4.45 MgCO3:3H2O

O2(g) -43.07 45.32 88.39 O2

Otavite -3.42 -15.41 -11.99 CdCO3

Pb(OH)2 -3.55 5.15 8.69 Pb(OH)2

Pb10(OH)6O(CO3)6 -64.08 -72.84 -8.76 Pb10(OH)6O(CO3)6

Pb2(OH)3Cl -10.26 -1.47 8.79 Pb2(OH)3Cl

Pb2O(OH)2 -15.89 10.29 26.19 Pb2O(OH)2

Pb2O3 -28.09 32.95 61.04 Pb2O3

Pb2OCO3 -10.25 -10.42 -0.18 Pb2OCO3

Pb3(AsO4)2 -16.64 -10.84 5.80 Pb3(AsO4)2

Pb3O2CO3 -17.32 -5.28 12.05 Pb3O2CO3

Pb3O2SO4 -14.42 -3.00 11.42 Pb3O2SO4

Pb4(OH)6SO4 -18.95 2.15 21.10 Pb4(OH)6SO4

Pb4O3SO4 -20.99 2.15 23.14 Pb4O3SO4

PbCrO4 -15.12 -28.13 -13.01 PbCrO4

Pbmetal -21.75 -17.51 4.24 Pb

PbO:0.3H2O -7.83 5.15 12.98 PbO:0.33H2O

Periclase -12.42 10.56 22.99 MgO

Phosgenite -14.14 -33.95 -19.81 PbCl2:PbCO3

Plattnerite -24.54 27.81 52.35 PbO2

Portlandite -12.60 11.39 24.00 Ca(OH)2

Smithsonite -3.67 -13.52 -9.85 ZnCO3

Tenorite -2.30 5.95 8.25 CuO

Thenardite -11.96 -11.56 0.41 Na2SO4

Thermonatrite -14.57 -13.83 0.73 Na2CO3:H2O

Zincite -4.97 7.20 12.17 ZnO

Zincosite -15.94 -11.24 4.70 ZnSO4

Zn(NO3)2:6H2O -17.71 -14.63 3.09 Zn(NO3)2:6H2O

Zn(OH)2 -5.00 7.20 12.20 Zn(OH)2

Zn(OH)2(am) -6.03 7.20 13.22 Zn(OH)2

Zn(OH)2(beta) -5.33 7.20 12.53 Zn(OH)2

Zn(OH)2(epsilon) -5.10 7.20 12.29 Zn(OH)2

Zn(OH)2(gamma) -4.54 7.20 11.73 Zn(OH)2

Zn2(OH)2SO4 -11.55 -4.05 7.50 Zn2(OH)2SO4

Zn2(OH)3Cl -12.56 2.63 15.19 Zn2(OH)3Cl

Zn3(AsO4)2:2.5H2O -18.35 -4.70 13.65 Zn3(AsO4)2:2.5H2O

Zn3O(SO4)2 -36.60 -15.29 21.31 Zn3O(SO4)2

Zn4(OH)6SO4 -18.05 10.35 28.40 Zn4(OH)6SO4

Zn5(OH)8Cl2 -26.05 12.45 38.50 Zn5(OH)8Cl2

ZnCl2 -24.05 -16.33 7.72 ZnCl2

ZnCO3:1H2O -3.26 -13.52 -10.26 ZnCO3:1H2O

Znmetal -42.68 -15.46 27.21 Zn

ZnO(active) -4.82 7.20 12.01 ZnO

ZnSO4:1H2O -11.02 -11.24 -0.23 ZnSO4:1H2O

**For a gas, SI = log10(fugacity). Fugacity = pressure * phi / 1 atm.

For ideal gases, phi = 1.

Initial solution 8.

----------------------------Distribution of species----------------------------

Log Log Log mole V

Species Molality Activity Molality Activity Gamma cm?mol

OH- 8.049e-08 7.499e-08 -7.094 -7.125 -0.031 (0)

H+ 4.369e-08 4.074e-08 -7.360 -7.390 -0.030 0.00

H2O 5.551e+01 9.999e-01 1.744 -0.000 0.000 18.02

As(3) 1.018e-16

H3AsO3 1.011e-16 1.011e-16 -15.995 -15.995 0.000 (0)

H2AsO3- 7.642e-19 7.082e-19 -18.117 -18.150 -0.033 (0)

HAsO3-2 1.085e-23 8.002e-24 -22.965 -23.097 -0.132 (0)

H4AsO3+ 2.201e-24 2.040e-24 -23.657 -23.690 -0.033 (0)

AsO3-3 8.744e-30 4.408e-30 -29.058 -29.356 -0.297 (0)

As(5) 3.892e-08

HAsO4-2 2.959e-08 2.182e-08 -7.529 -7.661 -0.132 (0)

H2AsO4- 9.328e-09 8.645e-09 -8.030 -8.063 -0.033 (0)

AsO4-3 2.337e-12 1.178e-12 -11.631 -11.929 -0.297 (0)

H3AsO4 5.253e-14 5.258e-14 -13.280 -13.279 0.000 (0)

C(4) 2.753e-03

HCO3- 2.467e-03 2.304e-03 -2.608 -2.637 -0.030 (0)

H2CO3 2.568e-04 2.568e-04 -3.590 -3.590 0.000 (0)

CaHCO3+ 2.178e-05 2.037e-05 -4.662 -4.691 -0.029 (0)

MgHCO3+ 2.884e-06 2.689e-06 -5.540 -5.570 -0.030 (0)

CO3-2 2.567e-06 1.941e-06 -5.591 -5.712 -0.121 (0)

CaCO3 1.590e-06 1.590e-06 -5.798 -5.798 0.000 (0)

NaHCO3 4.796e-07 4.796e-07 -6.319 -6.319 0.000 (0)

MgCO3 1.552e-07 1.552e-07 -6.809 -6.809 0.000 (0)

CuCO3 4.295e-08 4.295e-08 -7.367 -7.367 0.000 (0)

NaCO3- 1.650e-08 1.541e-08 -7.783 -7.812 -0.030 (0)

ZnCO3 8.386e-09 8.386e-09 -8.076 -8.076 0.000 (0)

CdCO3 7.318e-09 7.318e-09 -8.136 -8.136 0.000 (0)

ZnHCO3+ 4.321e-09 4.005e-09 -8.364 -8.397 -0.033 (0)

PbCO3 2.143e-09 2.143e-09 -8.669 -8.669 0.000 (0)

CdHCO3+ 6.854e-10 6.352e-10 -9.164 -9.197 -0.033 (0)

PbHCO3+ 4.967e-10 4.603e-10 -9.304 -9.337 -0.033 (0)

CuHCO3+ 4.315e-10 3.999e-10 -9.365 -9.398 -0.033 (0)

Cu(CO3)2-2 3.043e-10 2.244e-10 -9.517 -9.649 -0.132 (0)

Pb(CO3)2-2 1.627e-11 1.200e-11 -10.789 -10.921 -0.132 (0)

Cd(CO3)2-2 1.428e-11 1.053e-11 -10.845 -10.978 -0.132 (0)

Ca 1.012e-03

Ca+2 9.623e-04 7.277e-04 -3.017 -3.138 -0.121 (0)

CaSO4 2.489e-05 2.489e-05 -4.604 -4.604 0.000 (0)

CaHCO3+ 2.178e-05 2.037e-05 -4.662 -4.691 -0.029 (0)

CaCO3 1.590e-06 1.590e-06 -5.798 -5.798 0.000 (0)

CaNO3+ 1.126e-06 1.043e-06 -5.949 -5.982 -0.033 (0)

CaOH+ 9.754e-10 9.119e-10 -9.011 -9.040 -0.029 (0)

Cd 2.345e-07

Cd+2 2.187e-07 1.654e-07 -6.660 -6.781 -0.121 (0)

CdCO3 7.318e-09 7.318e-09 -8.136 -8.136 0.000 (0)

CdSO4 5.596e-09 5.596e-09 -8.252 -8.252 0.000 (0)

CdCl+ 1.703e-09 1.578e-09 -8.769 -8.802 -0.033 (0)

CdHCO3+ 6.854e-10 6.352e-10 -9.164 -9.197 -0.033 (0)

CdNO3+ 3.571e-10 3.309e-10 -9.447 -9.480 -0.033 (0)

CdOH+ 1.086e-10 1.007e-10 -9.964 -9.997 -0.033 (0)

Cd(SO4)2-2 2.142e-11 1.580e-11 -10.669 -10.801 -0.132 (0)

Cd(CO3)2-2 1.428e-11 1.053e-11 -10.845 -10.978 -0.132 (0)

CdOHCl 1.107e-11 1.107e-11 -10.956 -10.956 0.000 (0)

CdCl2 6.434e-13 6.434e-13 -12.192 -12.192 0.000 (0)

Cd(OH)2 5.064e-13 5.064e-13 -12.296 -12.296 0.000 (0)

Cd(NO3)2 4.277e-14 4.277e-14 -13.369 -13.369 0.000 (0)

Cd2OH+3 2.006e-16 1.011e-16 -15.698 -15.995 -0.297 (0)

CdCl3- 3.850e-17 3.568e-17 -16.415 -16.448 -0.033 (0)

Cd(OH)3- 8.251e-18 7.646e-18 -17.084 -17.117 -0.033 (0)

Cd(OH)4-2 4.194e-25 3.093e-25 -24.377 -24.510 -0.132 (0)

Cl 1.095e-04

Cl- 1.095e-04 1.021e-04 -3.961 -3.991 -0.030 (0)

CdCl+ 1.703e-09 1.578e-09 -8.769 -8.802 -0.033 (0)

CuCl 2.041e-11 2.041e-11 -10.690 -10.690 0.000 (0)

ZnCl+ 1.839e-11 1.715e-11 -10.735 -10.766 -0.030 (0)

CdOHCl 1.107e-11 1.107e-11 -10.956 -10.956 0.000 (0)

ZnOHCl 6.228e-12 6.228e-12 -11.206 -11.206 0.000 (0)

PbCl+ 1.192e-12 1.104e-12 -11.924 -11.957 -0.033 (0)

CdCl2 6.434e-13 6.434e-13 -12.192 -12.192 0.000 (0)

CuCl+ 5.460e-13 5.090e-13 -12.263 -12.293 -0.030 (0)

CuCl2- 4.846e-13 4.518e-13 -12.315 -12.345 -0.030 (0)

CrCl+2 5.486e-15 4.046e-15 -14.261 -14.393 -0.132 (0)

ZnCl2 1.412e-15 1.412e-15 -14.850 -14.850 0.000 (0)

PbCl2 4.692e-16 4.692e-16 -15.329 -15.329 0.000 (0)

CdCl3- 3.850e-17 3.568e-17 -16.415 -16.448 -0.033 (0)

CuCl3-2 1.221e-17 9.277e-18 -16.913 -17.033 -0.119 (0)

CuCl2 8.368e-18 8.368e-18 -17.077 -17.077 0.000 (0)

CrOHCl2 2.119e-19 2.119e-19 -18.674 -18.674 0.000 (0)

ZnCl3- 1.152e-19 1.074e-19 -18.939 -18.969 -0.030 (0)

PbCl3- 2.441e-20 2.262e-20 -19.612 -19.646 -0.033 (0)

CrCl2+ 1.068e-20 9.897e-21 -19.971 -20.005 -0.033 (0)

CuCl3- 6.462e-24 6.025e-24 -23.190 -23.220 -0.030 (0)

ZnCl4-2 6.365e-24 4.836e-24 -23.196 -23.316 -0.119 (0)

PbCl4-2 1.137e-24 8.383e-25 -23.944 -24.077 -0.132 (0)

CuCl4-2 6.881e-30 5.228e-30 -29.162 -29.282 -0.119 (0)

CrO3Cl- 2.062e-30 1.911e-30 -29.686 -29.719 -0.033 (0)

Cr(2) 9.461e-23

Cr+2 9.461e-23 6.978e-23 -22.024 -22.156 -0.132 (0)

Cr(3) 7.693e-07

Cr(OH)2+ 5.871e-07 5.441e-07 -6.231 -6.264 -0.033 (0)

Cr(OH)+2 1.296e-07 9.562e-08 -6.887 -7.019 -0.132 (0)

Cr(OH)3 5.051e-08 5.051e-08 -7.297 -7.297 0.000 (0)

CrOHSO4 7.462e-10 7.462e-10 -9.127 -9.127 0.000 (0)

CrO2- 6.355e-10 5.889e-10 -9.197 -9.230 -0.033 (0)

Cr(OH)4- 5.363e-10 4.970e-10 -9.271 -9.304 -0.033 (0)

Cr+3 1.059e-10 5.339e-11 -9.975 -10.273 -0.297 (0)

CrSO4+ 1.206e-11 1.117e-11 -10.919 -10.952 -0.033 (0)

CrCl+2 5.486e-15 4.046e-15 -14.261 -14.393 -0.132 (0)

Cr2(OH)2SO4+2 1.654e-15 1.220e-15 -14.781 -14.914 -0.132 (0)

CrNO3+2 3.243e-16 2.392e-16 -15.489 -15.621 -0.132 (0)

Cr2(OH)2(SO4)2 1.260e-17 1.260e-17 -16.900 -16.900 0.000 (0)

CrOHCl2 2.119e-19 2.119e-19 -18.674 -18.674 0.000 (0)

CrCl2+ 1.068e-20 9.897e-21 -19.971 -20.005 -0.033 (0)

Cr(6) 8.093e-19

CrO4-2 7.329e-19 5.542e-19 -18.135 -18.256 -0.121 (0)

HCrO4- 7.554e-20 7.000e-20 -19.122 -19.155 -0.033 (0)

NaCrO4- 8.202e-22 7.601e-22 -21.086 -21.119 -0.033 (0)

KCrO4- 9.616e-23 8.911e-23 -22.017 -22.050 -0.033 (0)

H2CrO4 1.048e-27 1.048e-27 -26.979 -26.979 0.000 (0)

CrO3SO4-2 2.136e-28 1.576e-28 -27.670 -27.803 -0.132 (0)

CrO3Cl- 2.062e-30 1.911e-30 -29.686 -29.719 -0.033 (0)

Cr2O7-2 3.458e-37 2.550e-37 -36.461 -36.593 -0.132 (0)

Cu(1) 1.922e-10

Cu+ 1.714e-10 1.588e-10 -9.766 -9.799 -0.033 (0)

CuCl 2.041e-11 2.041e-11 -10.690 -10.690 0.000 (0)

CuCl2- 4.846e-13 4.518e-13 -12.315 -12.345 -0.030 (0)

CuCl3-2 1.221e-17 9.277e-18 -16.913 -17.033 -0.119 (0)

Cu(2) 5.039e-08

CuCO3 4.295e-08 4.295e-08 -7.367 -7.367 0.000 (0)

Cu+2 4.969e-09 3.757e-09 -8.304 -8.425 -0.121 (0)

CuOH+ 1.465e-09 1.366e-09 -8.834 -8.864 -0.030 (0)

CuHCO3+ 4.315e-10 3.999e-10 -9.365 -9.398 -0.033 (0)

Cu(CO3)2-2 3.043e-10 2.244e-10 -9.517 -9.649 -0.132 (0)

Cu(OH)2 1.448e-10 1.448e-10 -9.839 -9.839 0.000 (0)

CuSO4 1.242e-10 1.242e-10 -9.906 -9.906 0.000 (0)

CuNO3+ 5.653e-12 5.239e-12 -11.248 -11.281 -0.033 (0)

CuCl+ 5.460e-13 5.090e-13 -12.263 -12.293 -0.030 (0)

Cu(OH)3- 7.922e-14 7.341e-14 -13.101 -13.134 -0.033 (0)

Cu2(OH)2+2 5.713e-14 4.213e-14 -13.243 -13.375 -0.132 (0)

Cu(NO3)2 2.440e-16 2.440e-16 -15.613 -15.613 0.000 (0)

CuCl2 8.368e-18 8.368e-18 -17.077 -17.077 0.000 (0)

Cu(OH)4-2 1.936e-19 1.428e-19 -18.713 -18.845 -0.132 (0)

CuCl3- 6.462e-24 6.025e-24 -23.190 -23.220 -0.030 (0)

CuCl4-2 6.881e-30 5.228e-30 -29.162 -29.282 -0.119 (0)

H(0) 2.747e-26

H2 1.374e-26 1.375e-26 -25.862 -25.862 0.000 (0)

K 4.646e-05

K+ 4.641e-05 4.328e-05 -4.333 -4.364 -0.030 (0)

KSO4- 5.222e-08 4.878e-08 -7.282 -7.312 -0.030 (0)

KCrO4- 9.616e-23 8.911e-23 -22.017 -22.050 -0.033 (0)

Mg 1.708e-04

Mg+2 1.643e-04 1.242e-04 -3.784 -3.906 -0.121 (0)

MgSO4 3.471e-06 3.471e-06 -5.460 -5.460 0.000 (0)

MgHCO3+ 2.884e-06 2.689e-06 -5.540 -5.570 -0.030 (0)

MgCO3 1.552e-07 1.552e-07 -6.809 -6.809 0.000 (0)

MgOH+ 3.068e-09 2.870e-09 -8.513 -8.542 -0.029 (0)

N(5) 4.343e-04

NO3- 4.331e-04 4.039e-04 -3.363 -3.394 -0.030 (0)

CaNO3+ 1.126e-06 1.043e-06 -5.949 -5.982 -0.033 (0)

CdNO3+ 3.571e-10 3.309e-10 -9.447 -9.480 -0.033 (0)

ZnNO3+ 9.069e-11 8.404e-11 -10.042 -10.076 -0.033 (0)

CuNO3+ 5.653e-12 5.239e-12 -11.248 -11.281 -0.033 (0)

PbNO3+ 2.268e-12 2.102e-12 -11.644 -11.677 -0.033 (0)

Cd(NO3)2 4.277e-14 4.277e-14 -13.369 -13.369 0.000 (0)

Zn(NO3)2 6.139e-15 6.139e-15 -14.212 -14.212 0.000 (0)

Pb(NO3)2 1.733e-15 1.733e-15 -14.761 -14.761 0.000 (0)

CrNO3+2 3.243e-16 2.392e-16 -15.489 -15.621 -0.132 (0)

Cu(NO3)2 2.440e-16 2.440e-16 -15.613 -15.613 0.000 (0)

Na 2.967e-04

Na+ 2.960e-04 2.760e-04 -3.529 -3.559 -0.030 (0)

NaHCO3 4.796e-07 4.796e-07 -6.319 -6.319 0.000 (0)

NaSO4- 2.699e-07 2.521e-07 -6.569 -6.598 -0.030 (0)

NaCO3- 1.650e-08 1.541e-08 -7.783 -7.812 -0.030 (0)

NaCrO4- 8.202e-22 7.601e-22 -21.086 -21.119 -0.033 (0)

O(0) 0.000e+00

O2 0.000e+00 0.000e+00 -44.900 -44.899 0.000 (0)

Pb 3.424e-09

PbCO3 2.143e-09 2.143e-09 -8.669 -8.669 0.000 (0)

PbHCO3+ 4.967e-10 4.603e-10 -9.304 -9.337 -0.033 (0)

Pb+2 4.856e-10 3.672e-10 -9.314 -9.435 -0.121 (0)

PbOH+ 2.460e-10 2.280e-10 -9.609 -9.642 -0.033 (0)

PbSO4 3.126e-11 3.126e-11 -10.505 -10.505 0.000 (0)

Pb(CO3)2-2 1.627e-11 1.200e-11 -10.789 -10.921 -0.132 (0)

PbNO3+ 2.268e-12 2.102e-12 -11.644 -11.677 -0.033 (0)

Pb(OH)2 1.782e-12 1.782e-12 -11.749 -11.749 0.000 (0)

PbCl+ 1.192e-12 1.104e-12 -11.924 -11.957 -0.033 (0)

Pb(SO4)2-2 4.439e-14 3.274e-14 -13.353 -13.485 -0.132 (0)

Pb(NO3)2 1.733e-15 1.733e-15 -14.761 -14.761 0.000 (0)

Pb(OH)3- 4.752e-16 4.404e-16 -15.323 -15.356 -0.033 (0)

PbCl2 4.692e-16 4.692e-16 -15.329 -15.329 0.000 (0)

Pb2OH+3 2.632e-18 1.327e-18 -17.580 -17.877 -0.297 (0)

Pb(OH)4-2 3.614e-20 2.666e-20 -19.442 -19.574 -0.132 (0)

PbCl3- 2.441e-20 2.262e-20 -19.612 -19.646 -0.033 (0)

Pb3(OH)4+2 2.688e-24 1.982e-24 -23.571 -23.703 -0.132 (0)

PbCl4-2 1.137e-24 8.383e-25 -23.944 -24.077 -0.132 (0)

Pb4(OH)4+4 3.480e-29 1.030e-29 -28.458 -28.987 -0.529 (0)

S(6) 2.585e-04

SO4-2 2.298e-04 1.738e-04 -3.639 -3.760 -0.121 (0)

CaSO4 2.489e-05 2.489e-05 -4.604 -4.604 0.000 (0)

MgSO4 3.471e-06 3.471e-06 -5.460 -5.460 0.000 (0)

NaSO4- 2.699e-07 2.521e-07 -6.569 -6.598 -0.030 (0)

KSO4- 5.222e-08 4.878e-08 -7.282 -7.312 -0.030 (0)

CdSO4 5.596e-09 5.596e-09 -8.252 -8.252 0.000 (0)

ZnSO4 2.500e-09 2.500e-09 -8.602 -8.602 0.000 (0)

CrOHSO4 7.462e-10 7.462e-10 -9.127 -9.127 0.000 (0)

HSO4- 4.634e-10 4.324e-10 -9.334 -9.364 -0.030 (0)

CuSO4 1.242e-10 1.242e-10 -9.906 -9.906 0.000 (0)

PbSO4 3.126e-11 3.126e-11 -10.505 -10.505 0.000 (0)

Cd(SO4)2-2 2.142e-11 1.580e-11 -10.669 -10.801 -0.132 (0)

CrSO4+ 1.206e-11 1.117e-11 -10.919 -10.952 -0.033 (0)

Zn(SO4)2-2 5.859e-12 4.321e-12 -11.232 -11.364 -0.132 (0)

Pb(SO4)2-2 4.439e-14 3.274e-14 -13.353 -13.485 -0.132 (0)

Cr2(OH)2SO4+2 1.654e-15 1.220e-15 -14.781 -14.914 -0.132 (0)

Cr2(OH)2(SO4)2 1.260e-17 1.260e-17 -16.900 -16.900 0.000 (0)

CrO3SO4-2 2.136e-28 1.576e-28 -27.670 -27.803 -0.132 (0)

Zn 1.153e-07

Zn+2 9.928e-08 7.508e-08 -7.003 -7.124 -0.121 (0)

ZnCO3 8.386e-09 8.386e-09 -8.076 -8.076 0.000 (0)

ZnHCO3+ 4.321e-09 4.005e-09 -8.364 -8.397 -0.033 (0)

ZnSO4 2.500e-09 2.500e-09 -8.602 -8.602 0.000 (0)

ZnOH+ 6.075e-10 5.630e-10 -9.216 -9.249 -0.033 (0)

ZnNO3+ 9.069e-11 8.404e-11 -10.042 -10.076 -0.033 (0)

Zn(OH)2 7.268e-11 7.268e-11 -10.139 -10.139 0.000 (0)

ZnCl+ 1.839e-11 1.715e-11 -10.735 -10.766 -0.030 (0)

ZnOHCl 6.228e-12 6.228e-12 -11.206 -11.206 0.000 (0)

Zn(SO4)2-2 5.859e-12 4.321e-12 -11.232 -11.364 -0.132 (0)

Zn(OH)3- 9.715e-14 9.003e-14 -13.013 -13.046 -0.033 (0)

Zn(NO3)2 6.139e-15 6.139e-15 -14.212 -14.212 0.000 (0)

ZnCl2 1.412e-15 1.412e-15 -14.850 -14.850 0.000 (0)

Zn(OH)4-2 1.201e-18 8.858e-19 -17.920 -18.053 -0.132 (0)

ZnCl3- 1.152e-19 1.074e-19 -18.939 -18.969 -0.030 (0)

ZnCl4-2 6.365e-24 4.836e-24 -23.196 -23.316 -0.119 (0)

------------------------------Saturation indices-------------------------------

Phase SI** log IAP log K(283 K, 1 atm)

Anglesite -5.29 -13.20 -7.90 PbSO4

Anhydrite -2.60 -6.90 -4.29 CaSO4

Antlerite -8.26 0.52 8.79 Cu3(OH)4SO4

Aragonite -0.66 -8.85 -8.19 CaCO3

Arsenolite -60.67 -63.98 -3.32 As4O6

Artinite -9.46 1.26 10.72 MgCO3:Mg(OH)2:3H2O

As2O5 -33.47 -26.56 6.92 As2O5

Atacamite -6.93 1.33 8.26 Cu2(OH)3Cl

Azurite -5.90 -21.92 -16.02 Cu3(OH)2(CO3)2

Bianchite -9.13 -10.88 -1.76 ZnSO4:6H2O

Brochantite -10.23 6.88 17.10 Cu4(OH)6SO4

Brucite -7.03 10.87 17.90 Mg(OH)2

Ca3(AsO4)2:4H2O -13.93 8.37 22.30 Ca3(AsO4)2:4H2O

CaCrO4 -19.38 -21.39 -2.02 CaCrO4

Calcite -0.44 -8.85 -8.41 CaCO3

Cd(OH)2 -6.52 8.00 14.52 Cd(OH)2

Cd(OH)2(am) -6.54 8.00 14.54 Cd(OH)2

Cd3(OH)2(SO4)2 -19.79 -13.08 6.71 Cd3(OH)2(SO4)2

Cd3(OH)4SO4 -17.10 5.46 22.56 Cd3(OH)4SO4

Cd4(OH)6SO4 -14.95 13.45 28.40 Cd4(OH)6SO4

CdCl2 -14.28 -14.76 -0.49 CdCl2

CdCl2:1H2O -13.14 -14.76 -1.62 CdCl2:1H2O

CdCl2:2.5H2O -12.78 -14.76 -1.98 CdCl2:2.5H2O

Cdmetal(alpha) -29.00 -14.78 14.21 Cd

Cdmetal(gamma) -29.10 -14.78 14.32 Cd

CdOHCl -7.21 -3.38 3.82 CdOHCl

CdSO4 -10.85 -10.54 0.31 CdSO4

CdSO4:1H2O -9.11 -10.54 -1.43 CdSO4:1H2O

CdSO4:2.67H2O -8.84 -10.54 -1.71 CdSO4:2.67H2O

Cerussite -1.79 -15.15 -13.36 PbCO3

CH4(g) -68.18 -111.61 -43.43 CH4

Chalcanthite -9.49 -12.19 -2.70 CuSO4:5H2O

Claudetite -60.40 -63.98 -3.58 As4O6

CO2(g) -2.31 -20.49 -18.18 CO2

Cotunnite -12.39 -17.42 -5.02 PbCl2

Cr(OH)2 -18.53 -7.38 11.15 Cr(OH)2

Cr(OH)3 -0.49 1.13 1.61 Cr(OH)3

Cr(OH)3(am) 1.88 1.13 -0.75 Cr(OH)3

Cr2O3 4.14 2.25 -1.89 Cr2O3

CrCl2 -45.26 -30.14 15.12 CrCl2

CrCl3 -49.26 -33.02 16.24 CrCl3

Crmetal -62.24 -30.16 32.08 Cr

CrO3 -29.87 -33.04 -3.16 CrO3

Cu(OH)2 -2.84 6.35 9.20 Cu(OH)2

Cu2(OH)3NO3 -8.00 1.93 9.92 Cu2(OH)3NO3

Cu2SO4 -21.59 -23.36 -1.77 Cu2SO4

Cu3(AsO4)2:2H2O -13.59 -7.49 6.10 Cu3(AsO4)2:2H2O

CuCO3 -2.64 -14.14 -11.50 CuCO3

CuCrO4 -21.24 -26.68 -5.44 CuCrO4

Cumetal -4.38 -13.80 -9.42 Cu

CuOCuSO4 -17.41 -5.83 11.58 CuOCuSO4

Cuprite -4.56 -4.82 -0.26 Cu2O

CuSO4 -15.80 -12.19 3.62 CuSO4

Dolomite(disordered) -2.36 -18.47 -16.11 CaMg(CO3)2

Dolomite(ordered) -1.74 -18.47 -16.72 CaMg(CO3)2

Epsomite -5.43 -7.67 -2.23 MgSO4:7H2O

Goslarite -8.74 -10.88 -2.14 ZnSO4:7H2O

Gypsum -2.28 -6.90 -4.62 CaSO4:2H2O

Halite -9.12 -7.55 1.57 NaCl

Huntite -8.74 -37.70 -28.97 CaMg3(CO3)4

Hydrocerussite -6.18 -24.95 -18.77 Pb3(OH)2(CO3)2

Hydromagnesite -20.86 -27.60 -6.74 Mg5(CO3)4(OH)2:4H2O

K2Cr2O7 -42.03 -60.02 -17.99 K2Cr2O7

K2CrO4 -26.30 -26.98 -0.68 K2CrO4

Langite -12.15 6.88 19.03 Cu4(OH)6SO4:H2O

Larnakite -7.62 -7.85 -0.23 PbO:PbSO4

Laurionite -6.66 -6.04 0.62 PbOHCl

Lime -22.86 11.64 34.50 CaO

Litharge -7.96 5.34 13.30 PbO

Magnesite -1.97 -9.62 -7.65 MgCO3

Malachite -1.77 -7.78 -6.01 Cu2(OH)2CO3

Massicot -8.17 5.34 13.51 PbO

Melanothallite -23.25 -16.41 6.85 CuCl2

Mg(OH)2(active) -7.92 10.87 18.79 Mg(OH)2

MgCr2O4 -4.74 13.13 17.87 MgCr2O4

MgCrO4 -28.37 -22.16 6.21 MgCrO4

Minium -38.62 38.81 77.44 Pb3O4

Mirabilite -9.03 -10.88 -1.85 Na2SO4:10H2O

Monteponite -8.06 8.00 16.06 CdO

Na2Cr2O7 -48.31 -58.41 -10.10 Na2Cr2O7

Na2CrO4 -28.49 -25.37 3.11 Na2CrO4

Nantokite -6.66 -13.79 -7.13 CuCl

Natron -10.91 -12.83 -1.92 Na2CO3:10H2O

Nesquehonite -5.17 -9.62 -4.45 MgCO3:3H2O

O2(g) -42.83 45.56 88.39 O2

Otavite -0.50 -12.49 -11.99 CdCO3

Pb(OH)2 -3.35 5.34 8.69 Pb(OH)2

Pb10(OH)6O(CO3)6 -60.74 -69.50 -8.76 Pb10(OH)6O(CO3)6

Pb2(OH)3Cl -9.48 -0.69 8.79 Pb2(OH)3Cl

Pb2O(OH)2 -15.50 10.69 26.19 Pb2O(OH)2

Pb2O3 -27.57 33.47 61.04 Pb2O3

Pb2OCO3 -9.62 -9.80 -0.18 Pb2OCO3

Pb3(AsO4)2 -16.32 -10.52 5.80 Pb3(AsO4)2

Pb3O2CO3 -16.50 -4.46 12.05 Pb3O2CO3

Pb3O2SO4 -13.93 -2.51 11.42 Pb3O2SO4

Pb4(OH)6SO4 -18.26 2.84 21.10 Pb4(OH)6SO4

Pb4O3SO4 -20.30 2.84 23.14 Pb4O3SO4

PbCrO4 -14.68 -27.69 -13.01 PbCrO4

Pbmetal -21.67 -17.44 4.24 Pb

PbO:0.3H2O -7.64 5.34 12.98 PbO:0.33H2O

Periclase -12.11 10.87 22.99 MgO

Phosgenite -12.75 -32.56 -19.81 PbCl2:PbCO3

Plattnerite -24.22 28.12 52.35 PbO2

Portlandite -12.36 11.64 24.00 Ca(OH)2

Smithsonite -2.98 -12.84 -9.85 ZnCO3

Tenorite -1.89 6.35 8.25 CuO

Thenardite -11.28 -10.88 0.41 Na2SO4

Thermonatrite -13.56 -12.83 0.73 Na2CO3:H2O

Zincite -4.51 7.66 12.17 ZnO

Zincosite -15.58 -10.88 4.70 ZnSO4

Zn(NO3)2:6H2O -17.00 -13.91 3.09 Zn(NO3)2:6H2O

Zn(OH)2 -4.54 7.66 12.20 Zn(OH)2

Zn(OH)2(am) -5.57 7.66 13.22 Zn(OH)2

Zn(OH)2(beta) -4.87 7.66 12.53 Zn(OH)2

Zn(OH)2(epsilon) -4.64 7.66 12.29 Zn(OH)2

Zn(OH)2(gamma) -4.08 7.66 11.73 Zn(OH)2

Zn2(OH)2SO4 -10.73 -3.23 7.50 Zn2(OH)2SO4

Zn2(OH)3Cl -11.26 3.93 15.19 Zn2(OH)3Cl

Zn3(AsO4)2:2.5H2O -17.24 -3.59 13.65 Zn3(AsO4)2:2.5H2O

Zn3O(SO4)2 -35.42 -14.11 21.31 Zn3O(SO4)2

Zn4(OH)6SO4 -16.32 12.08 28.40 Zn4(OH)6SO4

Zn5(OH)8Cl2 -22.98 15.52 38.50 Zn5(OH)8Cl2

ZnCl2 -22.83 -15.11 7.72 ZnCl2

ZnCO3:1H2O -2.58 -12.84 -10.26 ZnCO3:1H2O

Znmetal -42.34 -15.12 27.21 Zn

ZnO(active) -4.36 7.66 12.01 ZnO

ZnSO4:1H2O -10.66 -10.88 -0.23 ZnSO4:1H2O

**For a gas, SI = log10(fugacity). Fugacity = pressure * phi / 1 atm.

For ideal gases, phi = 1.

Initial solution 9.

----------------------------Distribution of species----------------------------

Log Log Log mole V

Species Molality Activity Molality Activity Gamma cm?mol

OH- 2.080e-06 1.973e-06 -5.682 -5.705 -0.023 (0)

H+ 1.633e-09 1.549e-09 -8.787 -8.810 -0.023 0.00

H2O 5.551e+01 1.000e+00 1.744 -0.000 0.000 18.02

As(3) 9.524e-23

H3AsO3 7.970e-23 7.970e-23 -22.099 -22.099 0.000 (0)

H2AsO3- 1.554e-23 1.469e-23 -22.809 -22.833 -0.024 (0)

HAsO3-2 5.464e-27 4.366e-27 -26.262 -26.360 -0.097 (0)

AsO3-3 1.048e-31 6.326e-32 -30.980 -31.199 -0.219 (0)

H4AsO3+ 6.469e-32 6.116e-32 -31.189 -31.214 -0.024 (0)

As(5) 1.046e-08

HAsO4-2 1.031e-08 8.238e-09 -7.987 -8.084 -0.097 (0)

H2AsO4- 1.312e-10 1.241e-10 -9.882 -9.906 -0.024 (0)

AsO4-3 1.938e-11 1.170e-11 -10.713 -10.932 -0.219 (0)

H3AsO4 2.868e-17 2.869e-17 -16.542 -16.542 0.000 (0)

C(4) 1.733e-03

HCO3- 1.657e-03 1.574e-03 -2.781 -2.803 -0.022 (0)

CO3-2 4.304e-05 3.487e-05 -4.366 -4.458 -0.091 (0)

CaCO3 1.364e-05 1.364e-05 -4.865 -4.865 0.000 (0)

CaHCO3+ 6.990e-06 6.641e-06 -5.156 -5.178 -0.022 (0)

H2CO3 6.667e-06 6.667e-06 -5.176 -5.176 0.000 (0)

MgCO3 3.121e-06 3.121e-06 -5.506 -5.506 0.000 (0)

MgHCO3+ 2.167e-06 2.056e-06 -5.664 -5.687 -0.023 (0)

ZnCO3 3.655e-08 3.655e-08 -7.437 -7.437 0.000 (0)

NaHCO3 2.781e-08 2.781e-08 -7.556 -7.556 0.000 (0)

NaCO3- 2.475e-08 2.350e-08 -7.606 -7.629 -0.022 (0)

CuCO3 6.668e-09 6.668e-09 -8.176 -8.176 0.000 (0)

PbCO3 1.843e-09 1.843e-09 -8.735 -8.735 0.000 (0)

Cu(CO3)2-2 7.831e-10 6.257e-10 -9.106 -9.204 -0.097 (0)

ZnHCO3+ 7.018e-10 6.635e-10 -9.154 -9.178 -0.024 (0)

CdCO3 5.237e-10 5.237e-10 -9.281 -9.281 0.000 (0)

Pb(CO3)2-2 2.319e-10 1.853e-10 -9.635 -9.732 -0.097 (0)

Cd(CO3)2-2 1.694e-11 1.354e-11 -10.771 -10.869 -0.097 (0)

PbHCO3+ 1.592e-11 1.505e-11 -10.798 -10.823 -0.024 (0)

CuHCO3+ 2.497e-12 2.360e-12 -11.603 -11.627 -0.024 (0)

CdHCO3+ 1.828e-12 1.728e-12 -11.738 -11.762 -0.024 (0)

Ca 4.537e-04

Ca+2 4.290e-04 3.475e-04 -3.368 -3.459 -0.091 (0)

CaCO3 1.364e-05 1.364e-05 -4.865 -4.865 0.000 (0)

CaHCO3+ 6.990e-06 6.641e-06 -5.156 -5.178 -0.022 (0)

CaSO4 3.821e-06 3.821e-06 -5.418 -5.418 0.000 (0)

CaNO3+ 2.537e-07 2.399e-07 -6.596 -6.620 -0.024 (0)

CaOH+ 1.206e-08 1.145e-08 -7.919 -7.941 -0.022 (0)

Cd 1.377e-09

Cd+2 8.135e-10 6.590e-10 -9.090 -9.181 -0.091 (0)

CdCO3 5.237e-10 5.237e-10 -9.281 -9.281 0.000 (0)

Cd(CO3)2-2 1.694e-11 1.354e-11 -10.771 -10.869 -0.097 (0)

CdOH+ 1.116e-11 1.055e-11 -10.952 -10.977 -0.024 (0)

CdSO4 7.166e-12 7.166e-12 -11.145 -11.145 0.000 (0)

CdHCO3+ 1.828e-12 1.728e-12 -11.738 -11.762 -0.024 (0)

Cd(OH)2 1.396e-12 1.396e-12 -11.855 -11.855 0.000 (0)

CdCl+ 8.409e-13 7.951e-13 -12.075 -12.100 -0.024 (0)

CdNO3+ 6.716e-13 6.349e-13 -12.173 -12.197 -0.024 (0)

CdOHCl 1.467e-13 1.467e-13 -12.833 -12.833 0.000 (0)

Cd(SO4)2-2 8.140e-15 6.504e-15 -14.089 -14.187 -0.097 (0)

Cd(OH)3- 5.864e-16 5.544e-16 -15.232 -15.256 -0.024 (0)

CdCl2 4.099e-17 4.099e-17 -16.387 -16.387 0.000 (0)

Cd(NO3)2 3.952e-17 3.952e-17 -16.403 -16.403 0.000 (0)

Cd2OH+3 6.995e-20 4.223e-20 -19.155 -19.374 -0.219 (0)

Cd(OH)4-2 7.383e-22 5.899e-22 -21.132 -21.229 -0.097 (0)

CdCl3- 3.040e-22 2.874e-22 -21.517 -21.542 -0.024 (0)

Cl 1.360e-05

Cl- 1.360e-05 1.291e-05 -4.866 -4.889 -0.023 (0)

ZnOHCl 5.026e-12 5.026e-12 -11.299 -11.299 0.000 (0)

CdCl+ 8.409e-13 7.951e-13 -12.075 -12.100 -0.024 (0)

ZnCl+ 5.547e-13 5.262e-13 -12.256 -12.279 -0.023 (0)

CdOHCl 1.467e-13 1.467e-13 -12.833 -12.833 0.000 (0)

CuCl 2.230e-14 2.230e-14 -13.652 -13.652 0.000 (0)

PbCl+ 7.071e-15 6.685e-15 -14.151 -14.175 -0.024 (0)

CuCl+ 5.865e-16 5.564e-16 -15.232 -15.255 -0.023 (0)

CuCl2- 6.582e-17 6.244e-17 -16.182 -16.205 -0.023 (0)

CdCl2 4.099e-17 4.099e-17 -16.387 -16.387 0.000 (0)

ZnCl2 5.478e-18 5.478e-18 -17.261 -17.261 0.000 (0)

PbCl2 3.592e-19 3.592e-19 -18.445 -18.445 0.000 (0)

CrCl+2 1.975e-19 1.578e-19 -18.704 -18.802 -0.097 (0)

CuCl2 1.156e-21 1.156e-21 -20.937 -20.937 0.000 (0)

CdCl3- 3.040e-22 2.874e-22 -21.517 -21.542 -0.024 (0)

CuCl3-2 1.996e-22 1.621e-22 -21.700 -21.790 -0.090 (0)

ZnCl3- 5.553e-23 5.268e-23 -22.255 -22.278 -0.023 (0)

CrOHCl2 2.749e-23 2.749e-23 -22.561 -22.561 0.000 (0)

PbCl3- 2.316e-24 2.190e-24 -23.635 -23.660 -0.024 (0)

CrCl2+ 5.162e-26 4.881e-26 -25.287 -25.312 -0.024 (0)

CrO3Cl- 2.610e-26 2.468e-26 -25.583 -25.608 -0.024 (0)

ZnCl4-2 3.692e-28 3.000e-28 -27.433 -27.523 -0.090 (0)

CuCl3- 1.110e-28 1.053e-28 -27.955 -27.978 -0.023 (0)

PbCl4-2 1.284e-29 1.026e-29 -28.891 -28.989 -0.097 (0)

CuCl4-2 1.422e-35 1.155e-35 -34.847 -34.937 -0.090 (0)

Cr(2) 2.693e-26

Cr+2 2.693e-26 2.152e-26 -25.570 -25.667 -0.097 (0)

Cr(3) 5.769e-07

Cr(OH)3 2.836e-07 2.836e-07 -6.547 -6.547 0.000 (0)

Cr(OH)2+ 1.228e-07 1.161e-07 -6.911 -6.935 -0.024 (0)

CrO2- 9.196e-08 8.695e-08 -7.036 -7.061 -0.024 (0)

Cr(OH)4- 7.762e-08 7.339e-08 -7.110 -7.134 -0.024 (0)

Cr(OH)+2 9.709e-10 7.758e-10 -9.013 -9.110 -0.097 (0)

CrOHSO4 1.946e-12 1.946e-12 -11.711 -11.711 0.000 (0)

Cr+3 2.728e-14 1.647e-14 -13.564 -13.783 -0.219 (0)

CrSO4+ 1.172e-15 1.108e-15 -14.931 -14.956 -0.024 (0)

CrCl+2 1.975e-19 1.578e-19 -18.704 -18.802 -0.097 (0)

CrNO3+2 4.446e-20 3.553e-20 -19.352 -19.449 -0.097 (0)

Cr2(OH)2SO4+2 3.231e-20 2.582e-20 -19.491 -19.588 -0.097 (0)

Cr2(OH)2(SO4)2 8.568e-23 8.568e-23 -22.067 -22.067 0.000 (0)

CrOHCl2 2.749e-23 2.749e-23 -22.561 -22.561 0.000 (0)

CrCl2+ 5.162e-26 4.881e-26 -25.287 -25.312 -0.024 (0)

Cr(6) 4.855e-11

CrO4-2 4.835e-11 3.917e-11 -10.316 -10.407 -0.091 (0)

HCrO4- 1.989e-13 1.881e-13 -12.701 -12.726 -0.024 (0)

NaCrO4- 4.824e-15 4.561e-15 -14.317 -14.341 -0.024 (0)

KCrO4- 3.535e-15 3.343e-15 -14.452 -14.476 -0.024 (0)

H2CrO4 1.071e-22 1.071e-22 -21.970 -21.970 0.000 (0)

CrO3SO4-2 6.475e-24 5.173e-24 -23.189 -23.286 -0.097 (0)

Cr2O7-2 2.304e-24 1.841e-24 -23.638 -23.735 -0.097 (0)

CrO3Cl- 2.610e-26 2.468e-26 -25.583 -25.608 -0.024 (0)

Cu(1) 1.474e-12

Cu+ 1.452e-12 1.373e-12 -11.838 -11.862 -0.024 (0)

CuCl 2.230e-14 2.230e-14 -13.652 -13.652 0.000 (0)

CuCl2- 6.582e-17 6.244e-17 -16.182 -16.205 -0.023 (0)

CuCl3-2 1.996e-22 1.621e-22 -21.700 -21.790 -0.090 (0)

Cu(2) 8.700e-09

CuCO3 6.668e-09 6.668e-09 -8.176 -8.176 0.000 (0)

Cu(OH)2 8.660e-10 8.660e-10 -9.062 -9.062 0.000 (0)

Cu(CO3)2-2 7.831e-10 6.257e-10 -9.106 -9.204 -0.097 (0)

CuOH+ 3.274e-10 3.106e-10 -9.485 -9.508 -0.023 (0)

Cu+2 4.009e-11 3.248e-11 -10.397 -10.488 -0.091 (0)

Cu(OH)3- 1.221e-11 1.155e-11 -10.913 -10.937 -0.024 (0)

CuHCO3+ 2.497e-12 2.360e-12 -11.603 -11.627 -0.024 (0)

CuSO4 3.451e-13 3.451e-13 -12.462 -12.462 0.000 (0)

CuNO3+ 2.306e-14 2.181e-14 -13.637 -13.661 -0.024 (0)

Cu2(OH)2+2 2.726e-15 2.178e-15 -14.565 -14.662 -0.097 (0)

Cu(OH)4-2 7.395e-16 5.909e-16 -15.131 -15.228 -0.097 (0)

CuCl+ 5.865e-16 5.564e-16 -15.232 -15.255 -0.023 (0)

Cu(NO3)2 4.892e-19 4.892e-19 -18.310 -18.310 0.000 (0)

CuCl2 1.156e-21 1.156e-21 -20.937 -20.937 0.000 (0)

CuCl3- 1.110e-28 1.053e-28 -27.955 -27.978 -0.023 (0)

CuCl4-2 1.422e-35 1.155e-35 -34.847 -34.937 -0.090 (0)

H(0) 3.973e-29

H2 1.986e-29 1.987e-29 -28.702 -28.702 0.000 (0)

K 2.422e-05

K+ 2.421e-05 2.297e-05 -4.616 -4.639 -0.023 (0)

KSO4- 8.764e-09 8.323e-09 -8.057 -8.080 -0.022 (0)

KCrO4- 3.535e-15 3.343e-15 -14.452 -14.476 -0.024 (0)

Mg 1.783e-04

Mg+2 1.717e-04 1.391e-04 -3.765 -3.857 -0.091 (0)

MgCO3 3.121e-06 3.121e-06 -5.506 -5.506 0.000 (0)

MgHCO3+ 2.167e-06 2.056e-06 -5.664 -5.687 -0.023 (0)

MgSO4 1.249e-06 1.249e-06 -5.903 -5.903 0.000 (0)

MgOH+ 8.892e-08 8.451e-08 -7.051 -7.073 -0.022 (0)

N(5) 2.053e-04

NO3- 2.050e-04 1.945e-04 -3.688 -3.711 -0.023 (0)

CaNO3+ 2.537e-07 2.399e-07 -6.596 -6.620 -0.024 (0)

ZnNO3+ 1.039e-11 9.820e-12 -10.984 -11.008 -0.024 (0)

CdNO3+ 6.716e-13 6.349e-13 -12.173 -12.197 -0.024 (0)

PbNO3+ 5.127e-14 4.847e-14 -13.290 -13.315 -0.024 (0)

CuNO3+ 2.306e-14 2.181e-14 -13.637 -13.661 -0.024 (0)

Zn(NO3)2 3.454e-16 3.454e-16 -15.462 -15.462 0.000 (0)

Cd(NO3)2 3.952e-17 3.952e-17 -16.403 -16.403 0.000 (0)

Pb(NO3)2 1.924e-17 1.924e-17 -16.716 -16.716 0.000 (0)

Cu(NO3)2 4.892e-19 4.892e-19 -18.310 -18.310 0.000 (0)

CrNO3+2 4.446e-20 3.553e-20 -19.352 -19.449 -0.097 (0)

Na 2.476e-05

Na+ 2.470e-05 2.343e-05 -4.607 -4.630 -0.023 (0)

NaHCO3 2.781e-08 2.781e-08 -7.556 -7.556 0.000 (0)

NaCO3- 2.475e-08 2.350e-08 -7.606 -7.629 -0.022 (0)

NaSO4- 7.246e-09 6.882e-09 -8.140 -8.162 -0.022 (0)

NaCrO4- 4.824e-15 4.561e-15 -14.317 -14.341 -0.024 (0)

O(0) 1.207e-39

O2 6.035e-40 6.038e-40 -39.219 -39.219 0.000 (0)

Pb 2.476e-09

PbCO3 1.843e-09 1.843e-09 -8.735 -8.735 0.000 (0)

PbOH+ 3.037e-10 2.871e-10 -9.518 -9.542 -0.024 (0)

Pb(CO3)2-2 2.319e-10 1.853e-10 -9.635 -9.732 -0.097 (0)

Pb(OH)2 5.902e-11 5.902e-11 -10.229 -10.229 0.000 (0)

Pb+2 2.170e-11 1.758e-11 -10.663 -10.755 -0.091 (0)

PbHCO3+ 1.592e-11 1.505e-11 -10.798 -10.823 -0.024 (0)

PbSO4 4.811e-13 4.811e-13 -12.318 -12.318 0.000 (0)

Pb(OH)3- 4.059e-13 3.837e-13 -12.392 -12.416 -0.024 (0)

PbNO3+ 5.127e-14 4.847e-14 -13.290 -13.315 -0.024 (0)

PbCl+ 7.071e-15 6.685e-15 -14.151 -14.175 -0.024 (0)

Pb(OH)4-2 7.646e-16 6.109e-16 -15.117 -15.214 -0.097 (0)

Pb(SO4)2-2 2.027e-16 1.619e-16 -15.693 -15.791 -0.097 (0)

Pb(NO3)2 1.924e-17 1.924e-17 -16.716 -16.716 0.000 (0)

PbCl2 3.592e-19 3.592e-19 -18.445 -18.445 0.000 (0)

Pb2OH+3 1.325e-19 8.001e-20 -18.878 -19.097 -0.219 (0)

Pb3(OH)4+2 1.303e-22 1.041e-22 -21.885 -21.982 -0.097 (0)

PbCl3- 2.316e-24 2.190e-24 -23.635 -23.660 -0.024 (0)

Pb4(OH)4+4 6.354e-29 2.590e-29 -28.197 -28.587 -0.390 (0)

PbCl4-2 1.284e-29 1.026e-29 -28.891 -28.989 -0.097 (0)

S(6) 7.405e-05

SO4-2 6.896e-05 5.587e-05 -4.161 -4.253 -0.091 (0)

CaSO4 3.821e-06 3.821e-06 -5.418 -5.418 0.000 (0)

MgSO4 1.249e-06 1.249e-06 -5.903 -5.903 0.000 (0)

KSO4- 8.764e-09 8.323e-09 -8.057 -8.080 -0.022 (0)

NaSO4- 7.246e-09 6.882e-09 -8.140 -8.162 -0.022 (0)

ZnSO4 1.950e-10 1.950e-10 -9.710 -9.710 0.000 (0)

CdSO4 7.166e-12 7.166e-12 -11.145 -11.145 0.000 (0)

HSO4- 5.568e-12 5.284e-12 -11.254 -11.277 -0.023 (0)

CrOHSO4 1.946e-12 1.946e-12 -11.711 -11.711 0.000 (0)

PbSO4 4.811e-13 4.811e-13 -12.318 -12.318 0.000 (0)

CuSO4 3.451e-13 3.451e-13 -12.462 -12.462 0.000 (0)

Zn(SO4)2-2 1.356e-13 1.083e-13 -12.868 -12.965 -0.097 (0)

Cd(SO4)2-2 8.140e-15 6.504e-15 -14.089 -14.187 -0.097 (0)

CrSO4+ 1.172e-15 1.108e-15 -14.931 -14.956 -0.024 (0)

Pb(SO4)2-2 2.027e-16 1.619e-16 -15.693 -15.791 -0.097 (0)

Cr2(OH)2SO4+2 3.231e-20 2.582e-20 -19.491 -19.588 -0.097 (0)

Cr2(OH)2(SO4)2 8.568e-23 8.568e-23 -22.067 -22.067 0.000 (0)

CrO3SO4-2 6.475e-24 5.173e-24 -23.189 -23.286 -0.097 (0)

Zn 7.637e-08

ZnCO3 3.655e-08 3.655e-08 -7.437 -7.437 0.000 (0)

Zn+2 2.249e-08 1.822e-08 -7.648 -7.740 -0.091 (0)

Zn(OH)2 1.220e-08 1.220e-08 -7.914 -7.914 0.000 (0)

ZnOH+ 3.800e-09 3.593e-09 -8.420 -8.445 -0.024 (0)

ZnHCO3+ 7.018e-10 6.635e-10 -9.154 -9.178 -0.024 (0)

Zn(OH)3- 4.205e-10 3.975e-10 -9.376 -9.401 -0.024 (0)

ZnSO4 1.950e-10 1.950e-10 -9.710 -9.710 0.000 (0)

ZnNO3+ 1.039e-11 9.820e-12 -10.984 -11.008 -0.024 (0)

ZnOHCl 5.026e-12 5.026e-12 -11.299 -11.299 0.000 (0)

ZnCl+ 5.547e-13 5.262e-13 -12.256 -12.279 -0.023 (0)

Zn(SO4)2-2 1.356e-13 1.083e-13 -12.868 -12.965 -0.097 (0)

Zn(OH)4-2 1.288e-13 1.029e-13 -12.890 -12.988 -0.097 (0)

Zn(NO3)2 3.454e-16 3.454e-16 -15.462 -15.462 0.000 (0)

ZnCl2 5.478e-18 5.478e-18 -17.261 -17.261 0.000 (0)

ZnCl3- 5.553e-23 5.268e-23 -22.255 -22.278 -0.023 (0)

ZnCl4-2 3.692e-28 3.000e-28 -27.433 -27.523 -0.090 (0)

------------------------------Saturation indices-------------------------------

Phase SI** log IAP log K(283 K, 1 atm)

Anglesite -7.11 -15.01 -7.90 PbSO4

Anhydrite -3.42 -7.71 -4.29 CaSO4

Antlerite -9.27 -0.48 8.79 Cu3(OH)4SO4

Aragonite 0.27 -7.92 -8.19 CaCO3

Arsenolite -85.08 -88.39 -3.32 As4O6

Artinite -5.27 5.45 10.72 MgCO3:Mg(OH)2:3H2O

As2O5 -40.00 -33.08 6.92 As2O5

Atacamite -7.69 0.56 8.26 Cu2(OH)3Cl

Azurite -6.74 -22.76 -16.02 Cu3(OH)2(CO3)2

Bianchite -10.23 -11.99 -1.76 ZnSO4:6H2O

Brochantite -10.45 6.65 17.10 Cu4(OH)6SO4

Brucite -4.14 13.76 17.90 Mg(OH)2

Ca3(AsO4)2:4H2O -12.90 9.40 22.30 Ca3(AsO4)2:4H2O

CaCrO4 -11.85 -13.87 -2.02 CaCrO4

Calcite 0.49 -7.92 -8.41 CaCO3

Cd(OH)2 -6.08 8.44 14.52 Cd(OH)2

Cd(OH)2(am) -6.10 8.44 14.54 Cd(OH)2

Cd3(OH)2(SO4)2 -25.14 -18.43 6.71 Cd3(OH)2(SO4)2

Cd3(OH)4SO4 -19.12 3.44 22.56 Cd3(OH)4SO4

Cd4(OH)6SO4 -16.52 11.88 28.40 Cd4(OH)6SO4

CdCl2 -18.47 -18.96 -0.49 CdCl2

CdCl2:1H2O -17.34 -18.96 -1.62 CdCl2:1H2O

CdCl2:2.5H2O -16.98 -18.96 -1.98 CdCl2:2.5H2O

Cdmetal(alpha) -31.39 -17.18 14.21 Cd

Cdmetal(gamma) -31.50 -17.18 14.32 Cd

CdOHCl -9.08 -5.26 3.82 CdOHCl

CdSO4 -13.74 -13.43 0.31 CdSO4

CdSO4:1H2O -12.00 -13.43 -1.43 CdSO4:1H2O

CdSO4:2.67H2O -11.73 -13.43 -1.71 CdSO4:2.67H2O

Cerussite -1.85 -15.21 -13.36 PbCO3

CH4(g) -81.13 -124.56 -43.43 CH4

Chalcanthite -12.05 -14.74 -2.70 CuSO4:5H2O

Claudetite -84.81 -88.39 -3.58 As4O6

CO2(g) -3.89 -22.08 -18.18 CO2

Cotunnite -15.51 -20.53 -5.02 PbCl2

Cr(OH)2 -19.20 -8.05 11.15 Cr(OH)2

Cr(OH)3 0.26 1.87 1.61 Cr(OH)3

Cr(OH)3(am) 2.62 1.87 -0.75 Cr(OH)3

Cr2O3 5.64 3.75 -1.89 Cr2O3

CrCl2 -50.57 -35.45 15.12 CrCl2

CrCl3 -55.46 -39.22 16.24 CrCl3

Crmetal -65.75 -33.67 32.08 Cr

CrO3 -24.86 -28.03 -3.16 CrO3

Cu(OH)2 -2.07 7.13 9.20 Cu(OH)2

Cu2(OH)3NO3 -8.18 1.74 9.92 Cu2(OH)3NO3

Cu2SO4 -26.20 -27.98 -1.77 Cu2SO4

Cu3(AsO4)2:2H2O -17.79 -11.69 6.10 Cu3(AsO4)2:2H2O

CuCO3 -3.45 -14.95 -11.50 CuCO3

CuCrO4 -15.46 -20.90 -5.44 CuCrO4

Cumetal -6.44 -15.86 -9.42 Cu

CuOCuSO4 -19.19 -7.61 11.58 CuOCuSO4

Cuprite -5.85 -6.10 -0.26 Cu2O

CuSO4 -18.36 -14.74 3.62 CuSO4

Dolomite(disordered) -0.12 -16.23 -16.11 CaMg(CO3)2

Dolomite(ordered) 0.49 -16.23 -16.72 CaMg(CO3)2

Epsomite -5.88 -8.11 -2.23 MgSO4:7H2O

Goslarite -9.85 -11.99 -2.14 ZnSO4:7H2O

Gypsum -3.09 -7.71 -4.62 CaSO4:2H2O

Halite -11.09 -9.52 1.57 NaCl

Huntite -3.89 -32.86 -28.97 CaMg3(CO3)4

Hydrocerussite -4.79 -23.56 -18.77 Pb3(OH)2(CO3)2

Hydromagnesite -12.76 -19.49 -6.74 Mg5(CO3)4(OH)2:4H2O

K2Cr2O7 -29.72 -47.71 -17.99 K2Cr2O7

K2CrO4 -19.00 -19.68 -0.68 K2CrO4

Langite -12.37 6.65 19.03 Cu4(OH)6SO4:H2O

Larnakite -7.91 -8.14 -0.23 PbO:PbSO4

Laurionite -7.46 -6.83 0.62 PbOHCl

Lime -20.34 14.16 34.50 CaO

Litharge -6.44 6.87 13.30 PbO

Magnesite -0.67 -8.31 -7.65 MgCO3

Malachite -1.80 -7.81 -6.01 Cu2(OH)2CO3

Massicot -6.65 6.87 13.51 PbO

Melanothallite -27.11 -20.27 6.85 CuCl2

Mg(OH)2(active) -5.03 13.76 18.79 Mg(OH)2

MgCr2O4 -0.35 17.51 17.87 MgCr2O4

MgCrO4 -20.47 -14.26 6.21 MgCrO4

Minium -31.22 46.22 77.44 Pb3O4

Mirabilite -11.66 -13.51 -1.85 Na2SO4:10H2O

Monteponite -7.62 8.44 16.06 CdO

Na2Cr2O7 -37.59 -47.69 -10.10 Na2Cr2O7

Na2CrO4 -22.78 -19.67 3.11 Na2CrO4

Nantokite -9.63 -16.75 -7.13 CuCl

Natron -11.80 -13.72 -1.92 Na2CO3:10H2O

Nesquehonite -3.87 -8.31 -4.45 MgCO3:3H2O

O2(g) -37.15 51.24 88.39 O2

Otavite -1.64 -13.64 -11.99 CdCO3

Pb(OH)2 -1.83 6.87 8.69 Pb(OH)2

Pb10(OH)6O(CO3)6 -55.05 -63.81 -8.76 Pb10(OH)6O(CO3)6

Pb2(OH)3Cl -8.76 0.03 8.79 Pb2(OH)3Cl

Pb2O(OH)2 -12.46 13.73 26.19 Pb2O(OH)2

Pb2O3 -21.69 39.35 61.04 Pb2O3

Pb2OCO3 -8.17 -8.35 -0.18 Pb2OCO3

Pb3(AsO4)2 -18.29 -12.49 5.80 Pb3(AsO4)2

Pb3O2CO3 -13.53 -1.48 12.05 Pb3O2CO3

Pb3O2SO4 -12.70 -1.28 11.42 Pb3O2SO4

Pb4(OH)6SO4 -15.51 5.59 21.10 Pb4(OH)6SO4

Pb4O3SO4 -17.56 5.59 23.14 Pb4O3SO4

PbCrO4 -8.15 -21.16 -13.01 PbCrO4

Pbmetal -22.99 -18.75 4.24 Pb

PbO:0.3H2O -6.11 6.87 12.98 PbO:0.33H2O

Periclase -9.22 13.76 22.99 MgO

Phosgenite -15.94 -35.75 -19.81 PbCl2:PbCO3

Plattnerite -19.86 32.49 52.35 PbO2

Portlandite -9.84 14.16 24.00 Ca(OH)2

Smithsonite -2.34 -12.20 -9.85 ZnCO3

Tenorite -1.11 7.13 8.25 CuO

Thenardite -13.92 -13.51 0.41 Na2SO4

Thermonatrite -14.45 -13.72 0.73 Na2CO3:H2O

Zincite -2.29 9.88 12.17 ZnO

Zincosite -16.69 -11.99 4.70 ZnSO4

Zn(NO3)2:6H2O -18.25 -15.16 3.09 Zn(NO3)2:6H2O

Zn(OH)2 -2.32 9.88 12.20 Zn(OH)2

Zn(OH)2(am) -3.34 9.88 13.22 Zn(OH)2

Zn(OH)2(beta) -2.65 9.88 12.53 Zn(OH)2

Zn(OH)2(epsilon) -2.41 9.88 12.29 Zn(OH)2

Zn(OH)2(gamma) -1.85 9.88 11.73 Zn(OH)2

Zn2(OH)2SO4 -9.61 -2.11 7.50 Zn2(OH)2SO4

Zn2(OH)3Cl -9.13 6.06 15.19 Zn2(OH)3Cl

Zn3(AsO4)2:2.5H2O -17.09 -3.44 13.65 Zn3(AsO4)2:2.5H2O

Zn3O(SO4)2 -35.41 -14.10 21.31 Zn3O(SO4)2

Zn4(OH)6SO4 -10.75 17.65 28.40 Zn4(OH)6SO4

Zn5(OH)8Cl2 -16.50 22.00 38.50 Zn5(OH)8Cl2

ZnCl2 -25.24 -17.52 7.72 ZnCl2

ZnCO3:1H2O -1.94 -12.20 -10.26 ZnCO3:1H2O

Znmetal -42.95 -15.74 27.21 Zn

ZnO(active) -2.13 9.88 12.01 ZnO

ZnSO4:1H2O -11.76 -11.99 -0.23 ZnSO4:1H2O

**For a gas, SI = log10(fugacity). Fugacity = pressure * phi / 1 atm.

For ideal gases, phi = 1.

Initial solution 10.

----------------------------Distribution of species----------------------------

Log Log Log mole V

Species Molality Activity Molality Activity Gamma cm?mol

OH- 2.188e-06 2.065e-06 -5.660 -5.685 -0.025 (0)

H+ 1.566e-09 1.479e-09 -8.805 -8.830 -0.025 0.00

H2O 5.551e+01 9.999e-01 1.744 -0.000 0.000 18.02

As(3) 9.168e-23

H3AsO3 7.607e-23 7.607e-23 -22.119 -22.119 0.000 (0)

H2AsO3- 1.561e-23 1.468e-23 -22.807 -22.833 -0.026 (0)

HAsO3-2 5.830e-27 4.569e-27 -26.234 -26.340 -0.106 (0)

AsO3-3 1.199e-31 6.932e-32 -30.921 -31.159 -0.238 (0)

H4AsO3+ 5.925e-32 5.574e-32 -31.227 -31.254 -0.026 (0)

As(5) 1.223e-08

HAsO4-2 1.206e-08 9.453e-09 -7.919 -8.024 -0.106 (0)

H2AsO4- 1.445e-10 1.360e-10 -9.840 -9.867 -0.026 (0)

AsO4-3 2.432e-11 1.405e-11 -10.614 -10.852 -0.238 (0)

H3AsO4 3.001e-17 3.003e-17 -16.523 -16.522 0.000 (0)

C(4) 2.087e-03

HCO3- 1.987e-03 1.879e-03 -2.702 -2.726 -0.024 (0)

CO3-2 5.474e-05 4.360e-05 -4.262 -4.360 -0.099 (0)

CaCO3 1.992e-05 1.992e-05 -4.701 -4.701 0.000 (0)

CaHCO3+ 9.788e-06 9.262e-06 -5.009 -5.033 -0.024 (0)

H2CO3 7.604e-06 7.604e-06 -5.119 -5.119 0.000 (0)

MgCO3 4.433e-06 4.433e-06 -5.353 -5.353 0.000 (0)

MgHCO3+ 2.953e-06 2.789e-06 -5.530 -5.555 -0.025 (0)

CuCO3 1.148e-07 1.148e-07 -6.940 -6.940 0.000 (0)

NaHCO3 4.876e-08 4.876e-08 -7.312 -7.312 0.000 (0)

NaCO3- 4.562e-08 4.315e-08 -7.341 -7.365 -0.024 (0)

ZnCO3 4.154e-08 4.154e-08 -7.382 -7.382 0.000 (0)

Cu(CO3)2-2 1.719e-08 1.347e-08 -7.765 -7.871 -0.106 (0)

CdCO3 1.699e-08 1.699e-08 -7.770 -7.770 0.000 (0)

PbCO3 2.859e-09 2.859e-09 -8.544 -8.544 0.000 (0)

ZnHCO3+ 7.654e-10 7.202e-10 -9.116 -9.143 -0.026 (0)

Cd(CO3)2-2 7.009e-10 5.493e-10 -9.154 -9.260 -0.106 (0)

Pb(CO3)2-2 4.587e-10 3.595e-10 -9.338 -9.444 -0.106 (0)

CdHCO3+ 5.692e-11 5.356e-11 -10.245 -10.271 -0.026 (0)

CuHCO3+ 4.124e-11 3.880e-11 -10.385 -10.411 -0.026 (0)

PbHCO3+ 2.369e-11 2.229e-11 -10.625 -10.652 -0.026 (0)

Ca 5.443e-04

Ca+2 5.095e-04 4.058e-04 -3.293 -3.392 -0.099 (0)

CaCO3 1.992e-05 1.992e-05 -4.701 -4.701 0.000 (0)

CaHCO3+ 9.788e-06 9.262e-06 -5.009 -5.033 -0.024 (0)

CaSO4 4.825e-06 4.825e-06 -5.316 -5.316 0.000 (0)

CaNO3+ 2.983e-07 2.807e-07 -6.525 -6.552 -0.026 (0)

CaOH+ 1.480e-08 1.401e-08 -7.830 -7.854 -0.024 (0)

Cd 3.982e-08

Cd+2 2.147e-08 1.710e-08 -7.668 -7.767 -0.099 (0)

CdCO3 1.699e-08 1.699e-08 -7.770 -7.770 0.000 (0)

Cd(CO3)2-2 7.009e-10 5.493e-10 -9.154 -9.260 -0.106 (0)

CdOH+ 3.046e-10 2.866e-10 -9.516 -9.543 -0.026 (0)

CdSO4 2.011e-10 2.011e-10 -9.697 -9.697 0.000 (0)

CdHCO3+ 5.692e-11 5.356e-11 -10.245 -10.271 -0.026 (0)

Cd(OH)2 3.971e-11 3.971e-11 -10.401 -10.401 0.000 (0)

CdCl+ 3.158e-11 2.971e-11 -10.501 -10.527 -0.026 (0)

CdNO3+ 1.754e-11 1.651e-11 -10.756 -10.782 -0.026 (0)

CdOHCl 5.742e-12 5.742e-12 -11.241 -11.241 0.000 (0)

Cd(SO4)2-2 2.518e-13 1.973e-13 -12.599 -12.705 -0.106 (0)

Cd(OH)3- 1.755e-14 1.652e-14 -13.756 -13.782 -0.026 (0)

CdCl2 2.206e-15 2.206e-15 -14.656 -14.656 0.000 (0)

Cd(NO3)2 1.029e-15 1.029e-15 -14.987 -14.987 0.000 (0)

Cd2OH+3 5.151e-17 2.977e-17 -16.288 -16.526 -0.238 (0)

CdCl3- 2.367e-20 2.227e-20 -19.626 -19.652 -0.026 (0)

Cd(OH)4-2 2.348e-20 1.840e-20 -19.629 -19.735 -0.106 (0)

Cl 1.967e-05

Cl- 1.967e-05 1.859e-05 -4.706 -4.731 -0.025 (0)

CdCl+ 3.158e-11 2.971e-11 -10.501 -10.527 -0.026 (0)

ZnOHCl 6.888e-12 6.888e-12 -11.162 -11.162 0.000 (0)

CdOHCl 5.742e-12 5.742e-12 -11.241 -11.241 0.000 (0)

ZnCl+ 7.290e-13 6.887e-13 -12.137 -12.162 -0.025 (0)

CuCl 4.421e-13 4.421e-13 -12.355 -12.355 0.000 (0)

PbCl+ 1.269e-14 1.194e-14 -13.896 -13.923 -0.026 (0)

CuCl+ 1.167e-14 1.103e-14 -13.933 -13.958 -0.025 (0)

CdCl2 2.206e-15 2.206e-15 -14.656 -14.656 0.000 (0)

CuCl2- 1.887e-15 1.782e-15 -14.724 -14.749 -0.025 (0)

ZnCl2 1.033e-17 1.033e-17 -16.986 -16.986 0.000 (0)

PbCl2 9.240e-19 9.240e-19 -18.034 -18.034 0.000 (0)

CrCl+2 3.345e-19 2.622e-19 -18.476 -18.581 -0.106 (0)

CuCl2 3.301e-20 3.301e-20 -19.481 -19.481 0.000 (0)

CdCl3- 2.367e-20 2.227e-20 -19.626 -19.652 -0.026 (0)

CuCl3-2 8.340e-21 6.665e-21 -20.079 -20.176 -0.097 (0)

ZnCl3- 1.514e-22 1.430e-22 -21.820 -21.845 -0.025 (0)

CrOHCl2 6.887e-23 6.887e-23 -22.162 -22.162 0.000 (0)

PbCl3- 8.621e-24 8.111e-24 -23.064 -23.091 -0.026 (0)

CrCl2+ 1.241e-25 1.168e-25 -24.906 -24.933 -0.026 (0)

CrO3Cl- 5.744e-26 5.405e-26 -25.241 -25.267 -0.026 (0)

CuCl3- 4.582e-27 4.328e-27 -26.339 -26.364 -0.025 (0)

ZnCl4-2 1.467e-27 1.173e-27 -26.833 -26.931 -0.097 (0)

PbCl4-2 6.985e-29 5.474e-29 -28.156 -28.262 -0.106 (0)

CuCl4-2 8.558e-34 6.839e-34 -33.068 -33.165 -0.097 (0)

Cr(2) 3.168e-26

Cr+2 3.168e-26 2.483e-26 -25.499 -25.605 -0.106 (0)

Cr(3) 7.692e-07

Cr(OH)3 3.756e-07 3.756e-07 -6.425 -6.425 0.000 (0)

Cr(OH)2+ 1.561e-07 1.469e-07 -6.807 -6.833 -0.026 (0)

CrO2- 1.282e-07 1.206e-07 -6.892 -6.919 -0.026 (0)

Cr(OH)4- 1.082e-07 1.018e-07 -6.966 -6.992 -0.026 (0)

Cr(OH)+2 1.196e-09 9.371e-10 -8.922 -9.028 -0.106 (0)

CrOHSO4 2.542e-12 2.542e-12 -11.595 -11.595 0.000 (0)

Cr+3 3.287e-14 1.900e-14 -13.483 -13.721 -0.238 (0)

CrSO4+ 1.469e-15 1.382e-15 -14.833 -14.860 -0.026 (0)

CrCl+2 3.345e-19 2.622e-19 -18.476 -18.581 -0.106 (0)

CrNO3+2 5.240e-20 4.107e-20 -19.281 -19.387 -0.106 (0)

Cr2(OH)2SO4+2 5.198e-20 4.074e-20 -19.284 -19.390 -0.106 (0)

Cr2(OH)2(SO4)2 1.462e-22 1.462e-22 -21.835 -21.835 0.000 (0)

CrOHCl2 6.887e-23 6.887e-23 -22.162 -22.162 0.000 (0)

CrCl2+ 1.241e-25 1.168e-25 -24.906 -24.933 -0.026 (0)

Cr(6) 8.233e-11

CrO4-2 8.199e-11 6.531e-11 -10.086 -10.185 -0.099 (0)

HCrO4- 3.183e-13 2.995e-13 -12.497 -12.524 -0.026 (0)

NaCrO4- 1.187e-14 1.117e-14 -13.926 -13.952 -0.026 (0)

KCrO4- 6.872e-15 6.465e-15 -14.163 -14.189 -0.026 (0)

H2CrO4 1.629e-22 1.629e-22 -21.788 -21.788 0.000 (0)

CrO3SO4-2 1.085e-23 8.507e-24 -22.964 -23.070 -0.106 (0)

Cr2O7-2 5.957e-24 4.668e-24 -23.225 -23.331 -0.106 (0)

CrO3Cl- 5.744e-26 5.405e-26 -25.241 -25.267 -0.026 (0)

Cu(1) 2.052e-11

Cu+ 2.008e-11 1.889e-11 -10.697 -10.724 -0.026 (0)

CuCl 4.421e-13 4.421e-13 -12.355 -12.355 0.000 (0)

CuCl2- 1.887e-15 1.782e-15 -14.724 -14.749 -0.025 (0)

CuCl3-2 8.340e-21 6.665e-21 -20.079 -20.176 -0.097 (0)

Cu(2) 1.506e-07

CuCO3 1.148e-07 1.148e-07 -6.940 -6.940 0.000 (0)

Cu(CO3)2-2 1.719e-08 1.347e-08 -7.765 -7.871 -0.106 (0)

Cu(OH)2 1.307e-08 1.307e-08 -7.884 -7.884 0.000 (0)

CuOH+ 4.739e-09 4.477e-09 -8.324 -8.349 -0.025 (0)

Cu+2 5.612e-10 4.470e-10 -9.251 -9.350 -0.099 (0)

Cu(OH)3- 1.940e-10 1.825e-10 -9.712 -9.739 -0.026 (0)

CuHCO3+ 4.124e-11 3.880e-11 -10.385 -10.411 -0.026 (0)

CuSO4 5.137e-12 5.137e-12 -11.289 -11.289 0.000 (0)

Cu2(OH)2+2 5.772e-13 4.524e-13 -12.239 -12.344 -0.106 (0)

CuNO3+ 3.196e-13 3.007e-13 -12.495 -12.522 -0.026 (0)

Cu(OH)4-2 1.248e-14 9.778e-15 -13.904 -14.010 -0.106 (0)

CuCl+ 1.167e-14 1.103e-14 -13.933 -13.958 -0.025 (0)

Cu(NO3)2 6.760e-18 6.760e-18 -17.170 -17.170 0.000 (0)

CuCl2 3.301e-20 3.301e-20 -19.481 -19.481 0.000 (0)

CuCl3- 4.582e-27 4.328e-27 -26.339 -26.364 -0.025 (0)

CuCl4-2 8.558e-34 6.839e-34 -33.068 -33.165 -0.097 (0)

H(0) 3.623e-29

H2 1.811e-29 1.813e-29 -28.742 -28.742 0.000 (0)

K 2.822e-05

K+ 2.821e-05 2.665e-05 -4.550 -4.574 -0.025 (0)

KSO4- 1.104e-08 1.044e-08 -7.957 -7.981 -0.024 (0)

KCrO4- 6.872e-15 6.465e-15 -14.163 -14.189 -0.026 (0)

Mg 2.073e-04

Mg+2 1.983e-04 1.580e-04 -3.703 -3.801 -0.099 (0)

MgCO3 4.433e-06 4.433e-06 -5.353 -5.353 0.000 (0)

MgHCO3+ 2.953e-06 2.789e-06 -5.530 -5.555 -0.025 (0)

MgSO4 1.534e-06 1.534e-06 -5.814 -5.814 0.000 (0)

MgOH+ 1.062e-07 1.005e-07 -6.974 -6.998 -0.024 (0)

N(5) 2.066e-04

NO3- 2.063e-04 1.949e-04 -3.685 -3.710 -0.025 (0)

CaNO3+ 2.983e-07 2.807e-07 -6.525 -6.552 -0.026 (0)

CdNO3+ 1.754e-11 1.651e-11 -10.756 -10.782 -0.026 (0)

ZnNO3+ 9.504e-12 8.942e-12 -11.022 -11.049 -0.026 (0)

CuNO3+ 3.196e-13 3.007e-13 -12.495 -12.522 -0.026 (0)

PbNO3+ 6.402e-14 6.024e-14 -13.194 -13.220 -0.026 (0)

Cd(NO3)2 1.029e-15 1.029e-15 -14.987 -14.987 0.000 (0)

Zn(NO3)2 3.152e-16 3.152e-16 -15.501 -15.501 0.000 (0)

Pb(NO3)2 2.396e-17 2.396e-17 -16.620 -16.620 0.000 (0)

Cu(NO3)2 6.760e-18 6.760e-18 -17.170 -17.170 0.000 (0)

CrNO3+2 5.240e-20 4.107e-20 -19.281 -19.387 -0.106 (0)

Na 3.652e-05

Na+ 3.642e-05 3.441e-05 -4.439 -4.463 -0.025 (0)

NaHCO3 4.876e-08 4.876e-08 -7.312 -7.312 0.000 (0)

NaCO3- 4.562e-08 4.315e-08 -7.341 -7.365 -0.024 (0)

NaSO4- 1.155e-08 1.093e-08 -7.937 -7.962 -0.024 (0)

NaCrO4- 1.187e-14 1.117e-14 -13.926 -13.952 -0.026 (0)

O(0) 1.451e-39

O2 7.255e-40 7.259e-40 -39.139 -39.139 0.000 (0)

Pb 3.846e-09

PbCO3 2.859e-09 2.859e-09 -8.544 -8.544 0.000 (0)

Pb(CO3)2-2 4.587e-10 3.595e-10 -9.338 -9.444 -0.106 (0)

PbOH+ 3.963e-10 3.729e-10 -9.402 -9.428 -0.026 (0)

Pb(OH)2 8.028e-11 8.028e-11 -10.095 -10.095 0.000 (0)

Pb+2 2.738e-11 2.181e-11 -10.563 -10.661 -0.099 (0)

PbHCO3+ 2.369e-11 2.229e-11 -10.625 -10.652 -0.026 (0)

PbSO4 6.453e-13 6.453e-13 -12.190 -12.190 0.000 (0)

Pb(OH)3- 5.808e-13 5.465e-13 -12.236 -12.262 -0.026 (0)

PbNO3+ 6.402e-14 6.024e-14 -13.194 -13.220 -0.026 (0)

PbCl+ 1.269e-14 1.194e-14 -13.896 -13.923 -0.026 (0)

Pb(OH)4-2 1.162e-15 9.111e-16 -14.935 -15.040 -0.106 (0)

Pb(SO4)2-2 2.997e-16 2.349e-16 -15.523 -15.629 -0.106 (0)

Pb(NO3)2 2.396e-17 2.396e-17 -16.620 -16.620 0.000 (0)

PbCl2 9.240e-19 9.240e-19 -18.034 -18.034 0.000 (0)

Pb2OH+3 2.230e-19 1.289e-19 -18.652 -18.890 -0.238 (0)

Pb3(OH)4+2 3.049e-22 2.390e-22 -21.516 -21.622 -0.106 (0)

PbCl3- 8.621e-24 8.111e-24 -23.064 -23.091 -0.026 (0)

Pb4(OH)4+4 1.954e-28 7.371e-29 -27.709 -28.132 -0.423 (0)

PbCl4-2 6.985e-29 5.474e-29 -28.156 -28.262 -0.106 (0)

S(6) 8.222e-05

SO4-2 7.584e-05 6.041e-05 -4.120 -4.219 -0.099 (0)

CaSO4 4.825e-06 4.825e-06 -5.316 -5.316 0.000 (0)

MgSO4 1.534e-06 1.534e-06 -5.814 -5.814 0.000 (0)

NaSO4- 1.155e-08 1.093e-08 -7.937 -7.962 -0.024 (0)

KSO4- 1.104e-08 1.044e-08 -7.957 -7.981 -0.024 (0)

CdSO4 2.011e-10 2.011e-10 -9.697 -9.697 0.000 (0)

ZnSO4 1.916e-10 1.916e-10 -9.718 -9.718 0.000 (0)

HSO4- 5.774e-12 5.457e-12 -11.239 -11.263 -0.025 (0)

CuSO4 5.137e-12 5.137e-12 -11.289 -11.289 0.000 (0)

CrOHSO4 2.542e-12 2.542e-12 -11.595 -11.595 0.000 (0)

PbSO4 6.453e-13 6.453e-13 -12.190 -12.190 0.000 (0)

Cd(SO4)2-2 2.518e-13 1.973e-13 -12.599 -12.705 -0.106 (0)

Zn(SO4)2-2 1.469e-13 1.151e-13 -12.833 -12.939 -0.106 (0)

CrSO4+ 1.469e-15 1.382e-15 -14.833 -14.860 -0.026 (0)

Pb(SO4)2-2 2.997e-16 2.349e-16 -15.523 -15.629 -0.106 (0)

Cr2(OH)2SO4+2 5.198e-20 4.074e-20 -19.284 -19.390 -0.106 (0)

Cr2(OH)2(SO4)2 1.462e-22 1.462e-22 -21.835 -21.835 0.000 (0)

CrO3SO4-2 1.085e-23 8.507e-24 -22.964 -23.070 -0.106 (0)

Zn 7.953e-08

ZnCO3 4.154e-08 4.154e-08 -7.382 -7.382 0.000 (0)

Zn+2 2.078e-08 1.656e-08 -7.682 -7.781 -0.099 (0)

Zn(OH)2 1.216e-08 1.216e-08 -7.915 -7.915 0.000 (0)

ZnOH+ 3.634e-09 3.419e-09 -8.440 -8.466 -0.026 (0)

ZnHCO3+ 7.654e-10 7.202e-10 -9.116 -9.143 -0.026 (0)

Zn(OH)3- 4.409e-10 4.148e-10 -9.356 -9.382 -0.026 (0)

ZnSO4 1.916e-10 1.916e-10 -9.718 -9.718 0.000 (0)

ZnNO3+ 9.504e-12 8.942e-12 -11.022 -11.049 -0.026 (0)

ZnOHCl 6.888e-12 6.888e-12 -11.162 -11.162 0.000 (0)

ZnCl+ 7.290e-13 6.887e-13 -12.137 -12.162 -0.025 (0)

Zn(SO4)2-2 1.469e-13 1.151e-13 -12.833 -12.939 -0.106 (0)

Zn(OH)4-2 1.434e-13 1.124e-13 -12.843 -12.949 -0.106 (0)

Zn(NO3)2 3.152e-16 3.152e-16 -15.501 -15.501 0.000 (0)

ZnCl2 1.033e-17 1.033e-17 -16.986 -16.986 0.000 (0)

ZnCl3- 1.514e-22 1.430e-22 -21.820 -21.845 -0.025 (0)

ZnCl4-2 1.467e-27 1.173e-27 -26.833 -26.931 -0.097 (0)

------------------------------Saturation indices-------------------------------

Phase SI** log IAP log K(283 K, 1 atm)

Anglesite -6.98 -14.88 -7.90 PbSO4

Anhydrite -3.32 -7.61 -4.29 CaSO4

Antlerite -5.74 3.05 8.79 Cu3(OH)4SO4

Aragonite 0.44 -7.75 -8.19 CaCO3

Arsenolite -85.16 -88.48 -3.32 As4O6

Artinite -5.02 5.70 10.72 MgCO3:Mg(OH)2:3H2O

As2O5 -39.96 -33.04 6.92 As2O5

Atacamite -5.20 3.06 8.26 Cu2(OH)3Cl

Azurite -3.09 -19.11 -16.02 Cu3(OH)2(CO3)2

Bianchite -10.24 -12.00 -1.76 ZnSO4:6H2O

Brochantite -5.74 11.36 17.10 Cu4(OH)6SO4

Brucite -4.04 13.86 17.90 Mg(OH)2

Ca3(AsO4)2:4H2O -12.54 9.76 22.30 Ca3(AsO4)2:4H2O

CaCrO4 -11.56 -13.58 -2.02 CaCrO4

Calcite 0.65 -7.75 -8.41 CaCO3

Cd(OH)2 -4.63 9.89 14.52 Cd(OH)2

Cd(OH)2(am) -4.64 9.89 14.54 Cd(OH)2

Cd3(OH)2(SO4)2 -20.79 -14.08 6.71 Cd3(OH)2(SO4)2

Cd3(OH)4SO4 -14.76 7.80 22.56 Cd3(OH)4SO4

Cd4(OH)6SO4 -10.71 17.69 28.40 Cd4(OH)6SO4

CdCl2 -16.74 -17.23 -0.49 CdCl2

CdCl2:1H2O -15.60 -17.23 -1.62 CdCl2:1H2O

CdCl2:2.5H2O -15.25 -17.23 -1.98 CdCl2:2.5H2O

Cdmetal(alpha) -29.98 -15.77 14.21 Cd

Cdmetal(gamma) -30.09 -15.77 14.32 Cd

CdOHCl -7.49 -3.67 3.82 CdOHCl

CdSO4 -12.30 -11.99 0.31 CdSO4

CdSO4:1H2O -10.55 -11.99 -1.43 CdSO4:1H2O

CdSO4:2.67H2O -10.28 -11.99 -1.71 CdSO4:2.67H2O

Cerussite -1.66 -15.02 -13.36 PbCO3

CH4(g) -81.23 -124.66 -43.43 CH4

Chalcanthite -10.87 -13.57 -2.70 CuSO4:5H2O

Claudetite -84.89 -88.48 -3.58 As4O6

CO2(g) -3.84 -22.02 -18.18 CO2

Cotunnite -15.10 -20.12 -5.02 PbCl2

Cr(OH)2 -19.09 -7.95 11.15 Cr(OH)2

Cr(OH)3 0.39 2.00 1.61 Cr(OH)3

Cr(OH)3(am) 2.75 2.00 -0.75 Cr(OH)3

Cr2O3 5.88 3.99 -1.89 Cr2O3

CrCl2 -50.19 -35.07 15.12 CrCl2

CrCl3 -54.92 -38.69 16.24 CrCl3

Crmetal -65.68 -33.61 32.08 Cr

CrO3 -24.68 -27.85 -3.16 CrO3

Cu(OH)2 -0.89 8.31 9.20 Cu(OH)2

Cu2(OH)3NO3 -5.84 4.08 9.92 Cu2(OH)3NO3

Cu2SO4 -23.89 -25.67 -1.77 Cu2SO4

Cu3(AsO4)2:2H2O -14.21 -8.11 6.10 Cu3(AsO4)2:2H2O

CuCO3 -2.21 -13.71 -11.50 CuCO3

CuCrO4 -14.09 -19.53 -5.44 CuCrO4

Cumetal -5.30 -14.72 -9.42 Cu

CuOCuSO4 -16.84 -5.26 11.58 CuOCuSO4

Cuprite -3.53 -3.79 -0.26 Cu2O

CuSO4 -17.19 -13.57 3.62 CuSO4

Dolomite(disordered) 0.20 -15.91 -16.11 CaMg(CO3)2

Dolomite(ordered) 0.81 -15.91 -16.72 CaMg(CO3)2

Epsomite -5.79 -8.02 -2.23 MgSO4:7H2O

Goslarite -9.86 -12.00 -2.14 ZnSO4:7H2O

Gypsum -2.99 -7.61 -4.62 CaSO4:2H2O

Halite -10.76 -9.19 1.57 NaCl

Huntite -3.27 -32.24 -28.97 CaMg3(CO3)4

Hydrocerussite -4.27 -23.05 -18.77 Pb3(OH)2(CO3)2

Hydromagnesite -12.05 -18.79 -6.74 Mg5(CO3)4(OH)2:4H2O

K2Cr2O7 -29.19 -47.18 -17.99 K2Cr2O7

K2CrO4 -18.65 -19.33 -0.68 K2CrO4

Langite -7.66 11.36 19.03 Cu4(OH)6SO4:H2O

Larnakite -7.65 -7.88 -0.23 PbO:PbSO4

Laurionite -7.19 -6.56 0.62 PbOHCl

Lime -20.23 14.27 34.50 CaO

Litharge -6.30 7.00 13.30 PbO

Magnesite -0.52 -8.16 -7.65 MgCO3

Malachite 0.61 -5.40 -6.01 Cu2(OH)2CO3

Massicot -6.52 7.00 13.51 PbO

Melanothallite -25.66 -18.81 6.85 CuCl2

Mg(OH)2(active) -4.94 13.86 18.79 Mg(OH)2

MgCr2O4 -0.01 17.85 17.87 MgCr2O4

MgCrO4 -20.19 -13.99 6.21 MgCrO4

Minium -30.78 46.66 77.44 Pb3O4

Mirabilite -11.29 -13.15 -1.85 Na2SO4:10H2O

Monteponite -6.17 9.89 16.06 CdO

Na2Cr2O7 -36.86 -46.96 -10.10 Na2Cr2O7

Na2CrO4 -22.22 -19.11 3.11 Na2CrO4

Nantokite -8.33 -15.45 -7.13 CuCl

Natron -11.37 -13.29 -1.92 Na2CO3:10H2O

Nesquehonite -3.72 -8.16 -4.45 MgCO3:3H2O

O2(g) -37.07 51.32 88.39 O2

Otavite -0.13 -12.13 -11.99 CdCO3

Pb(OH)2 -1.69 7.00 8.69 Pb(OH)2

Pb10(OH)6O(CO3)6 -53.38 -62.14 -8.76 Pb10(OH)6O(CO3)6

Pb2(OH)3Cl -8.36 0.44 8.79 Pb2(OH)3Cl

Pb2O(OH)2 -12.19 14.00 26.19 Pb2O(OH)2

Pb2O3 -21.38 39.66 61.04 Pb2O3

Pb2OCO3 -7.84 -8.02 -0.18 Pb2OCO3

Pb3(AsO4)2 -17.85 -12.05 5.80 Pb3(AsO4)2

Pb3O2CO3 -13.07 -1.02 12.05 Pb3O2CO3

Pb3O2SO4 -12.30 -0.88 11.42 Pb3O2SO4

Pb4(OH)6SO4 -14.98 6.12 21.10 Pb4(OH)6SO4

Pb4O3SO4 -17.03 6.12 23.14 Pb4O3SO4

PbCrO4 -7.84 -20.85 -13.01 PbCrO4

Pbmetal -22.90 -18.66 4.24 Pb

PbO:0.3H2O -5.98 7.00 12.98 PbO:0.33H2O

Periclase -9.13 13.86 22.99 MgO

Phosgenite -15.33 -35.14 -19.81 PbCl2:PbCO3

Plattnerite -19.69 32.66 52.35 PbO2

Portlandite -9.73 14.27 24.00 Ca(OH)2

Smithsonite -2.29 -12.14 -9.85 ZnCO3

Tenorite 0.06 8.31 8.25 CuO

Thenardite -13.55 -13.15 0.41 Na2SO4

Thermonatrite -14.02 -13.29 0.73 Na2CO3:H2O

Zincite -2.29 9.88 12.17 ZnO

Zincosite -16.70 -12.00 4.70 ZnSO4

Zn(NO3)2:6H2O -18.29 -15.20 3.09 Zn(NO3)2:6H2O

Zn(OH)2 -2.32 9.88 12.20 Zn(OH)2

Zn(OH)2(am) -3.34 9.88 13.22 Zn(OH)2

Zn(OH)2(beta) -2.65 9.88 12.53 Zn(OH)2

Zn(OH)2(epsilon) -2.41 9.88 12.29 Zn(OH)2

Zn(OH)2(gamma) -1.86 9.88 11.73 Zn(OH)2

Zn2(OH)2SO4 -9.62 -2.12 7.50 Zn2(OH)2SO4

Zn2(OH)3Cl -8.99 6.20 15.19 Zn2(OH)3Cl

Zn3(AsO4)2:2.5H2O -17.06 -3.41 13.65 Zn3(AsO4)2:2.5H2O

Zn3O(SO4)2 -35.43 -14.12 21.31 Zn3O(SO4)2

Zn4(OH)6SO4 -10.76 17.64 28.40 Zn4(OH)6SO4

Zn5(OH)8Cl2 -16.23 22.27 38.50 Zn5(OH)8Cl2

ZnCl2 -24.97 -17.24 7.72 ZnCl2

ZnCO3:1H2O -1.88 -12.14 -10.26 ZnCO3:1H2O

Znmetal -42.99 -15.78 27.21 Zn

ZnO(active) -2.13 9.88 12.01 ZnO

ZnSO4:1H2O -11.77 -12.00 -0.23 ZnSO4:1H2O

**For a gas, SI = log10(fugacity). Fugacity = pressure * phi / 1 atm.

For ideal gases, phi = 1.

Initial solution 11.

----------------------------Distribution of species----------------------------

Log Log Log mole V

Species Molality Activity Molality Activity Gamma cm?mol

OH- 1.866e-06 1.758e-06 -5.729 -5.755 -0.026 (0)

H+ 1.843e-09 1.738e-09 -8.734 -8.760 -0.026 0.00

H2O 5.551e+01 9.999e-01 1.744 -0.000 0.000 18.02

As(3) 5.279e-22

H3AsO3 4.493e-22 4.493e-22 -21.347 -21.347 0.000 (0)

H2AsO3- 7.863e-23 7.382e-23 -22.104 -22.132 -0.027 (0)

HAsO3-2 2.517e-26 1.955e-26 -25.599 -25.709 -0.110 (0)

AsO3-3 4.458e-31 2.525e-31 -30.351 -30.598 -0.247 (0)

H4AsO3+ 4.121e-31 3.868e-31 -30.385 -30.412 -0.027 (0)

As(5) 3.832e-08

HAsO4-2 3.773e-08 2.930e-08 -7.423 -7.533 -0.110 (0)

H2AsO4- 5.275e-10 4.952e-10 -9.278 -9.305 -0.027 (0)

AsO4-3 6.547e-11 3.708e-11 -10.184 -10.431 -0.247 (0)

H3AsO4 1.284e-16 1.285e-16 -15.891 -15.891 0.000 (0)

C(4) 1.992e-03

HCO3- 1.906e-03 1.799e-03 -2.720 -2.745 -0.025 (0)

CO3-2 4.495e-05 3.553e-05 -4.347 -4.449 -0.102 (0)

CaCO3 1.715e-05 1.715e-05 -4.766 -4.766 0.000 (0)

CaHCO3+ 9.916e-06 9.367e-06 -5.004 -5.028 -0.025 (0)

H2CO3 8.552e-06 8.552e-06 -5.068 -5.068 0.000 (0)

MgCO3 3.106e-06 3.106e-06 -5.508 -5.508 0.000 (0)

MgHCO3+ 2.435e-06 2.296e-06 -5.614 -5.639 -0.026 (0)

NaHCO3 1.087e-07 1.087e-07 -6.964 -6.964 0.000 (0)

NaCO3- 8.673e-08 8.188e-08 -7.062 -7.087 -0.025 (0)

ZnCO3 4.470e-08 4.470e-08 -7.350 -7.350 0.000 (0)

CuCO3 2.834e-08 2.834e-08 -7.548 -7.548 0.000 (0)

CdCO3 7.676e-09 7.676e-09 -8.115 -8.115 0.000 (0)

Cu(CO3)2-2 3.489e-09 2.710e-09 -8.457 -8.567 -0.110 (0)

PbCO3 1.694e-09 1.694e-09 -8.771 -8.771 0.000 (0)

ZnHCO3+ 9.698e-10 9.105e-10 -9.013 -9.041 -0.027 (0)

Cd(CO3)2-2 2.602e-10 2.021e-10 -9.585 -9.694 -0.110 (0)

Pb(CO3)2-2 2.234e-10 1.735e-10 -9.651 -9.761 -0.110 (0)

CdHCO3+ 3.027e-11 2.842e-11 -10.519 -10.546 -0.027 (0)

PbHCO3+ 1.653e-11 1.552e-11 -10.782 -10.809 -0.027 (0)

CuHCO3+ 1.199e-11 1.126e-11 -10.921 -10.949 -0.027 (0)

Ca 5.829e-04

Ca+2 5.424e-04 4.287e-04 -3.266 -3.368 -0.102 (0)

CaCO3 1.715e-05 1.715e-05 -4.766 -4.766 0.000 (0)

CaSO4 1.310e-05 1.310e-05 -4.883 -4.883 0.000 (0)

CaHCO3+ 9.916e-06 9.367e-06 -5.004 -5.028 -0.025 (0)

CaNO3+ 2.800e-07 2.628e-07 -6.553 -6.580 -0.027 (0)

CaOH+ 1.333e-08 1.259e-08 -7.875 -7.900 -0.025 (0)

Cd 2.045e-08

Cd+2 1.199e-08 9.479e-09 -7.921 -8.023 -0.102 (0)

CdCO3 7.676e-09 7.676e-09 -8.115 -8.115 0.000 (0)

CdSO4 2.865e-10 2.865e-10 -9.543 -9.543 0.000 (0)

Cd(CO3)2-2 2.602e-10 2.021e-10 -9.585 -9.694 -0.110 (0)

CdOH+ 1.440e-10 1.352e-10 -9.841 -9.869 -0.027 (0)

CdHCO3+ 3.027e-11 2.842e-11 -10.519 -10.546 -0.027 (0)

CdCl+ 2.789e-11 2.618e-11 -10.555 -10.582 -0.027 (0)

Cd(OH)2 1.595e-11 1.595e-11 -10.797 -10.797 0.000 (0)

CdNO3+ 8.640e-12 8.111e-12 -11.063 -11.091 -0.027 (0)

CdOHCl 4.306e-12 4.306e-12 -11.366 -11.366 0.000 (0)

Cd(SO4)2-2 9.304e-13 7.226e-13 -12.031 -12.141 -0.110 (0)

Cd(OH)3- 6.014e-15 5.645e-15 -14.221 -14.248 -0.027 (0)

CdCl2 3.089e-15 3.089e-15 -14.510 -14.510 0.000 (0)

Cd(NO3)2 4.483e-16 4.483e-16 -15.348 -15.348 0.000 (0)

Cd2OH+3 1.375e-17 7.787e-18 -16.862 -17.109 -0.247 (0)

CdCl3- 5.282e-20 4.959e-20 -19.277 -19.305 -0.027 (0)

Cd(OH)4-2 6.893e-21 5.354e-21 -20.162 -20.271 -0.110 (0)

Cl 3.133e-05

Cl- 3.133e-05 2.954e-05 -4.504 -4.530 -0.026 (0)

CdCl+ 2.789e-11 2.618e-11 -10.555 -10.582 -0.027 (0)

ZnOHCl 1.231e-11 1.231e-11 -10.910 -10.910 0.000 (0)

CdOHCl 4.306e-12 4.306e-12 -11.366 -11.366 0.000 (0)

ZnCl+ 1.533e-12 1.446e-12 -11.814 -11.840 -0.026 (0)

CuCl 2.130e-13 2.130e-13 -12.672 -12.672 0.000 (0)

PbCl+ 1.470e-14 1.380e-14 -13.833 -13.860 -0.027 (0)

CuCl+ 5.636e-15 5.313e-15 -14.249 -14.275 -0.026 (0)

CdCl2 3.089e-15 3.089e-15 -14.510 -14.510 0.000 (0)

CuCl2- 1.448e-15 1.365e-15 -14.839 -14.865 -0.026 (0)

ZnCl2 3.446e-17 3.446e-17 -16.463 -16.463 0.000 (0)

PbCl2 1.698e-18 1.698e-18 -17.770 -17.770 0.000 (0)

CrCl+2 6.583e-19 5.113e-19 -18.182 -18.291 -0.110 (0)

CdCl3- 5.282e-20 4.959e-20 -19.277 -19.305 -0.027 (0)

CuCl2 2.528e-20 2.528e-20 -19.597 -19.597 0.000 (0)

CuCl3-2 1.023e-20 8.113e-21 -19.990 -20.091 -0.101 (0)

ZnCl3- 8.045e-22 7.585e-22 -21.094 -21.120 -0.026 (0)

CrOHCl2 1.817e-22 1.817e-22 -21.741 -21.741 0.000 (0)

PbCl3- 2.524e-23 2.369e-23 -22.598 -22.625 -0.027 (0)

CrCl2+ 3.856e-25 3.620e-25 -24.414 -24.441 -0.027 (0)

CrO3Cl- 4.269e-26 4.007e-26 -25.370 -25.397 -0.027 (0)

ZnCl4-2 1.247e-26 9.886e-27 -25.904 -26.005 -0.101 (0)

CuCl3- 5.589e-27 5.269e-27 -26.253 -26.278 -0.026 (0)

PbCl4-2 3.272e-28 2.542e-28 -27.485 -27.595 -0.110 (0)

CuCl4-2 1.669e-33 1.323e-33 -32.778 -32.878 -0.101 (0)

Cr(2) 3.922e-26

Cr+2 3.922e-26 3.046e-26 -25.407 -25.516 -0.110 (0)

Cr(3) 5.769e-07

Cr(OH)3 2.841e-07 2.841e-07 -6.547 -6.547 0.000 (0)

Cr(OH)2+ 1.390e-07 1.305e-07 -6.857 -6.884 -0.027 (0)

CrO2- 8.271e-08 7.765e-08 -7.082 -7.110 -0.027 (0)

Cr(OH)4- 6.981e-08 6.553e-08 -7.156 -7.184 -0.027 (0)

Cr(OH)+2 1.260e-09 9.786e-10 -8.900 -9.009 -0.110 (0)

CrOHSO4 6.822e-12 6.822e-12 -11.166 -11.166 0.000 (0)

Cr+3 4.116e-14 2.331e-14 -13.386 -13.632 -0.247 (0)

CrSO4+ 4.641e-15 4.357e-15 -14.333 -14.361 -0.027 (0)

CrCl+2 6.583e-19 5.113e-19 -18.182 -18.291 -0.110 (0)

Cr2(OH)2SO4+2 1.470e-19 1.142e-19 -18.833 -18.942 -0.110 (0)

CrNO3+2 5.749e-20 4.466e-20 -19.240 -19.350 -0.110 (0)

Cr2(OH)2(SO4)2 1.053e-21 1.053e-21 -20.978 -20.978 0.000 (0)

CrOHCl2 1.817e-22 1.817e-22 -21.741 -21.741 0.000 (0)

CrCl2+ 3.856e-25 3.620e-25 -24.414 -24.441 -0.027 (0)

Cr(6) 2.806e-11

CrO4-2 2.792e-11 2.207e-11 -10.554 -10.656 -0.102 (0)

HCrO4- 1.267e-13 1.189e-13 -12.897 -12.925 -0.027 (0)

NaCrO4- 9.360e-15 8.787e-15 -14.029 -14.056 -0.027 (0)

KCrO4- 2.430e-15 2.281e-15 -14.614 -14.642 -0.027 (0)

H2CrO4 7.597e-23 7.597e-23 -22.119 -22.119 0.000 (0)

CrO3SO4-2 1.313e-23 1.020e-23 -22.882 -22.991 -0.110 (0)

Cr2O7-2 9.473e-25 7.358e-25 -24.024 -24.133 -0.110 (0)

CrO3Cl- 4.269e-26 4.007e-26 -25.370 -25.397 -0.027 (0)

Cu(1) 6.314e-12

Cu+ 6.100e-12 5.726e-12 -11.215 -11.242 -0.027 (0)

CuCl 2.130e-13 2.130e-13 -12.672 -12.672 0.000 (0)

CuCl2- 1.448e-15 1.365e-15 -14.839 -14.865 -0.026 (0)

CuCl3-2 1.023e-20 8.113e-21 -19.990 -20.091 -0.101 (0)

Cu(2) 3.615e-08

CuCO3 2.834e-08 2.834e-08 -7.548 -7.548 0.000 (0)

Cu(CO3)2-2 3.489e-09 2.710e-09 -8.457 -8.567 -0.110 (0)

Cu(OH)2 2.870e-09 2.870e-09 -8.542 -8.542 0.000 (0)

CuOH+ 1.225e-09 1.155e-09 -8.912 -8.937 -0.026 (0)

Cu+2 1.714e-10 1.355e-10 -9.766 -9.868 -0.102 (0)

Cu(OH)3- 3.633e-11 3.411e-11 -10.440 -10.467 -0.027 (0)

CuHCO3+ 1.199e-11 1.126e-11 -10.921 -10.949 -0.027 (0)

CuSO4 4.002e-12 4.002e-12 -11.398 -11.398 0.000 (0)

CuNO3+ 8.606e-14 8.080e-14 -13.065 -13.093 -0.027 (0)

Cu2(OH)2+2 3.876e-14 3.011e-14 -13.412 -13.521 -0.110 (0)

CuCl+ 5.636e-15 5.313e-15 -14.249 -14.275 -0.026 (0)

Cu(OH)4-2 2.002e-15 1.555e-15 -14.698 -14.808 -0.110 (0)

Cu(NO3)2 1.610e-18 1.610e-18 -17.793 -17.793 0.000 (0)

CuCl2 2.528e-20 2.528e-20 -19.597 -19.597 0.000 (0)

CuCl3- 5.589e-27 5.269e-27 -26.253 -26.278 -0.026 (0)

CuCl4-2 1.669e-33 1.323e-33 -32.778 -32.878 -0.101 (0)

H(0) 5.001e-29

H2 2.500e-29 2.502e-29 -28.602 -28.602 0.000 (0)

K 2.954e-05

K+ 2.951e-05 2.782e-05 -4.530 -4.556 -0.026 (0)

KSO4- 2.968e-08 2.802e-08 -7.528 -7.553 -0.025 (0)

KCrO4- 2.430e-15 2.281e-15 -14.614 -14.642 -0.027 (0)

Mg 1.809e-04

Mg+2 1.719e-04 1.358e-04 -3.765 -3.867 -0.102 (0)

MgSO4 3.390e-06 3.390e-06 -5.470 -5.470 0.000 (0)

MgCO3 3.106e-06 3.106e-06 -5.508 -5.508 0.000 (0)

MgHCO3+ 2.435e-06 2.296e-06 -5.614 -5.639 -0.026 (0)

MgOH+ 7.784e-08 7.356e-08 -7.109 -7.133 -0.025 (0)

N(5) 1.835e-04

NO3- 1.832e-04 1.728e-04 -3.737 -3.763 -0.026 (0)

CaNO3+ 2.800e-07 2.628e-07 -6.553 -6.580 -0.027 (0)

ZnNO3+ 1.115e-11 1.047e-11 -10.953 -10.980 -0.027 (0)

CdNO3+ 8.640e-12 8.111e-12 -11.063 -11.091 -0.027 (0)

CuNO3+ 8.606e-14 8.080e-14 -13.065 -13.093 -0.027 (0)

PbNO3+ 4.136e-14 3.883e-14 -13.383 -13.411 -0.027 (0)

Cd(NO3)2 4.483e-16 4.483e-16 -15.348 -15.348 0.000 (0)

Zn(NO3)2 3.270e-16 3.270e-16 -15.485 -15.485 0.000 (0)

Pb(NO3)2 1.369e-17 1.369e-17 -16.864 -16.864 0.000 (0)

Cu(NO3)2 1.610e-18 1.610e-18 -17.793 -17.793 0.000 (0)

CrNO3+2 5.749e-20 4.466e-20 -19.240 -19.350 -0.110 (0)

Na 8.525e-05

Na+ 8.498e-05 8.013e-05 -4.071 -4.096 -0.026 (0)

NaHCO3 1.087e-07 1.087e-07 -6.964 -6.964 0.000 (0)

NaCO3- 8.673e-08 8.188e-08 -7.062 -7.087 -0.025 (0)

NaSO4- 6.928e-08 6.540e-08 -7.159 -7.184 -0.025 (0)

NaCrO4- 9.360e-15 8.787e-15 -14.029 -14.056 -0.027 (0)

O(0) 7.614e-40

O2 3.807e-40 3.810e-40 -39.419 -39.419 0.000 (0)

Pb 2.243e-09

PbCO3 1.694e-09 1.694e-09 -8.771 -8.771 0.000 (0)

PbOH+ 2.459e-10 2.308e-10 -9.609 -9.637 -0.027 (0)

Pb(CO3)2-2 2.234e-10 1.735e-10 -9.651 -9.761 -0.110 (0)

Pb(OH)2 4.229e-11 4.229e-11 -10.374 -10.374 0.000 (0)

Pb+2 2.007e-11 1.586e-11 -10.698 -10.800 -0.102 (0)

PbHCO3+ 1.653e-11 1.552e-11 -10.782 -10.809 -0.027 (0)

PbSO4 1.206e-12 1.206e-12 -11.919 -11.919 0.000 (0)

Pb(OH)3- 2.610e-13 2.450e-13 -12.583 -12.611 -0.027 (0)

PbNO3+ 4.136e-14 3.883e-14 -13.383 -13.411 -0.027 (0)

PbCl+ 1.470e-14 1.380e-14 -13.833 -13.860 -0.027 (0)

Pb(SO4)2-2 1.453e-15 1.128e-15 -14.838 -14.948 -0.110 (0)

Pb(OH)4-2 4.476e-16 3.477e-16 -15.349 -15.459 -0.110 (0)

Pb(NO3)2 1.369e-17 1.369e-17 -16.864 -16.864 0.000 (0)

PbCl2 1.698e-18 1.698e-18 -17.770 -17.770 0.000 (0)

Pb2OH+3 1.024e-19 5.802e-20 -18.990 -19.236 -0.247 (0)

Pb3(OH)4+2 6.209e-23 4.822e-23 -22.207 -22.317 -0.110 (0)

PbCl3- 2.524e-23 2.369e-23 -22.598 -22.625 -0.027 (0)

PbCl4-2 3.272e-28 2.542e-28 -27.485 -27.595 -0.110 (0)

Pb4(OH)4+4 2.972e-29 1.082e-29 -28.527 -28.966 -0.439 (0)

S(6) 2.131e-04

SO4-2 1.965e-04 1.553e-04 -3.707 -3.809 -0.102 (0)

CaSO4 1.310e-05 1.310e-05 -4.883 -4.883 0.000 (0)

MgSO4 3.390e-06 3.390e-06 -5.470 -5.470 0.000 (0)

NaSO4- 6.928e-08 6.540e-08 -7.159 -7.184 -0.025 (0)

KSO4- 2.968e-08 2.802e-08 -7.528 -7.553 -0.025 (0)

ZnSO4 6.505e-10 6.505e-10 -9.187 -9.187 0.000 (0)

CdSO4 2.865e-10 2.865e-10 -9.543 -9.543 0.000 (0)

HSO4- 1.747e-11 1.648e-11 -10.758 -10.783 -0.025 (0)

CrOHSO4 6.822e-12 6.822e-12 -11.166 -11.166 0.000 (0)

CuSO4 4.002e-12 4.002e-12 -11.398 -11.398 0.000 (0)

Zn(SO4)2-2 1.293e-12 1.004e-12 -11.888 -11.998 -0.110 (0)

PbSO4 1.206e-12 1.206e-12 -11.919 -11.919 0.000 (0)

Cd(SO4)2-2 9.304e-13 7.226e-13 -12.031 -12.141 -0.110 (0)

CrSO4+ 4.641e-15 4.357e-15 -14.333 -14.361 -0.027 (0)

Pb(SO4)2-2 1.453e-15 1.128e-15 -14.838 -14.948 -0.110 (0)

Cr2(OH)2SO4+2 1.470e-19 1.142e-19 -18.833 -18.942 -0.110 (0)

Cr2(OH)2(SO4)2 1.053e-21 1.053e-21 -20.978 -20.978 0.000 (0)

CrO3SO4-2 1.313e-23 1.020e-23 -22.882 -22.991 -0.110 (0)

Zn 9.009e-08

ZnCO3 4.470e-08 4.470e-08 -7.350 -7.350 0.000 (0)

Zn+2 2.766e-08 2.186e-08 -7.558 -7.660 -0.102 (0)

Zn(OH)2 1.163e-08 1.163e-08 -7.934 -7.934 0.000 (0)

ZnOH+ 4.094e-09 3.844e-09 -8.388 -8.415 -0.027 (0)

ZnHCO3+ 9.698e-10 9.105e-10 -9.013 -9.041 -0.027 (0)

ZnSO4 6.505e-10 6.505e-10 -9.187 -9.187 0.000 (0)

Zn(OH)3- 3.598e-10 3.378e-10 -9.444 -9.471 -0.027 (0)

ZnOHCl 1.231e-11 1.231e-11 -10.910 -10.910 0.000 (0)

ZnNO3+ 1.115e-11 1.047e-11 -10.953 -10.980 -0.027 (0)

ZnCl+ 1.533e-12 1.446e-12 -11.814 -11.840 -0.026 (0)

Zn(SO4)2-2 1.293e-12 1.004e-12 -11.888 -11.998 -0.110 (0)

Zn(OH)4-2 1.003e-13 7.792e-14 -12.999 -13.108 -0.110 (0)

Zn(NO3)2 3.270e-16 3.270e-16 -15.485 -15.485 0.000 (0)

ZnCl2 3.446e-17 3.446e-17 -16.463 -16.463 0.000 (0)

ZnCl3- 8.045e-22 7.585e-22 -21.094 -21.120 -0.026 (0)

ZnCl4-2 1.247e-26 9.886e-27 -25.904 -26.005 -0.101 (0)

------------------------------Saturation indices-------------------------------

Phase SI** log IAP log K(283 K, 1 atm)

Anglesite -6.71 -14.61 -7.90 PbSO4

Anhydrite -2.88 -7.18 -4.29 CaSO4

Antlerite -7.16 1.63 8.79 Cu3(OH)4SO4

Aragonite 0.37 -7.82 -8.19 CaCO3

Arsenolite -82.07 -85.39 -3.32 As4O6

Artinite -5.38 5.34 10.72 MgCO3:Mg(OH)2:3H2O

As2O5 -38.70 -31.78 6.92 As2O5

Atacamite -6.24 2.01 8.26 Cu2(OH)3Cl

Azurite -4.96 -20.98 -16.02 Cu3(OH)2(CO3)2

Bianchite -9.71 -11.47 -1.76 ZnSO4:6H2O

Brochantite -7.83 9.28 17.10 Cu4(OH)6SO4

Brucite -4.25 13.65 17.90 Mg(OH)2

Ca3(AsO4)2:4H2O -11.63 10.67 22.30 Ca3(AsO4)2:4H2O

CaCrO4 -12.01 -14.02 -2.02 CaCrO4

Calcite 0.59 -7.82 -8.41 CaCO3

Cd(OH)2 -5.03 9.50 14.52 Cd(OH)2

Cd(OH)2(am) -5.04 9.50 14.54 Cd(OH)2

Cd3(OH)2(SO4)2 -20.88 -14.17 6.71 Cd3(OH)2(SO4)2

Cd3(OH)4SO4 -15.40 7.16 22.56 Cd3(OH)4SO4

Cd4(OH)6SO4 -11.74 16.66 28.40 Cd4(OH)6SO4

CdCl2 -16.60 -17.08 -0.49 CdCl2

CdCl2:1H2O -15.46 -17.08 -1.62 CdCl2:1H2O

CdCl2:2.5H2O -15.10 -17.08 -1.98 CdCl2:2.5H2O

Cdmetal(alpha) -30.24 -16.02 14.21 Cd

Cdmetal(gamma) -30.35 -16.02 14.32 Cd

CdOHCl -7.62 -3.79 3.82 CdOHCl

CdSO4 -12.14 -11.83 0.31 CdSO4

CdSO4:1H2O -10.40 -11.83 -1.43 CdSO4:1H2O

CdSO4:2.67H2O -10.13 -11.83 -1.71 CdSO4:2.67H2O

Cerussite -1.89 -15.25 -13.36 PbCO3

CH4(g) -80.62 -124.05 -43.43 CH4

Chalcanthite -10.98 -13.68 -2.70 CuSO4:5H2O

Claudetite -81.81 -85.39 -3.58 As4O6

CO2(g) -3.78 -21.97 -18.18 CO2

Cotunnite -14.84 -19.86 -5.02 PbCl2

Cr(OH)2 -19.15 -8.00 11.15 Cr(OH)2

Cr(OH)3 0.26 1.88 1.61 Cr(OH)3

Cr(OH)3(am) 2.63 1.88 -0.75 Cr(OH)3

Cr2O3 5.64 3.75 -1.89 Cr2O3

CrCl2 -49.69 -34.58 15.12 CrCl2

CrCl3 -54.23 -37.99 16.24 CrCl3

Crmetal -65.60 -33.52 32.08 Cr

CrO3 -25.01 -28.18 -3.16 CrO3

Cu(OH)2 -1.55 7.65 9.20 Cu(OH)2

Cu2(OH)3NO3 -7.14 2.78 9.92 Cu2(OH)3NO3

Cu2SO4 -24.52 -26.29 -1.77 Cu2SO4

Cu3(AsO4)2:2H2O -14.93 -8.83 6.10 Cu3(AsO4)2:2H2O

CuCO3 -2.82 -14.32 -11.50 CuCO3

CuCrO4 -15.08 -20.52 -5.44 CuCrO4

Cumetal -5.82 -15.24 -9.42 Cu

CuOCuSO4 -17.61 -6.03 11.58 CuOCuSO4

Cuprite -4.71 -4.96 -0.26 Cu2O

CuSO4 -17.29 -13.68 3.62 CuSO4

Dolomite(disordered) -0.02 -16.13 -16.11 CaMg(CO3)2

Dolomite(ordered) 0.59 -16.13 -16.72 CaMg(CO3)2

Epsomite -5.44 -7.68 -2.23 MgSO4:7H2O

Goslarite -9.33 -11.47 -2.14 ZnSO4:7H2O

Gypsum -2.56 -7.18 -4.62 CaSO4:2H2O

Halite -10.19 -8.63 1.57 NaCl

Huntite -3.80 -32.77 -28.97 CaMg3(CO3)4

Hydrocerussite -5.01 -23.78 -18.77 Pb3(OH)2(CO3)2

Hydromagnesite -12.87 -19.61 -6.74 Mg5(CO3)4(OH)2:4H2O

K2Cr2O7 -29.95 -47.94 -17.99 K2Cr2O7

K2CrO4 -19.08 -19.77 -0.68 K2CrO4

Langite -9.75 9.28 19.03 Cu4(OH)6SO4:H2O

Larnakite -7.66 -7.89 -0.23 PbO:PbSO4

Laurionite -7.19 -6.57 0.62 PbOHCl

Lime -20.35 14.15 34.50 CaO

Litharge -6.58 6.72 13.30 PbO

Magnesite -0.67 -8.32 -7.65 MgCO3

Malachite -0.65 -6.67 -6.01 Cu2(OH)2CO3

Massicot -6.79 6.72 13.51 PbO

Melanothallite -25.77 -18.93 6.85 CuCl2

Mg(OH)2(active) -5.14 13.65 18.79 Mg(OH)2

MgCr2O4 -0.46 17.40 17.87 MgCr2O4

MgCrO4 -20.73 -14.52 6.21 MgCrO4

Minium -31.76 45.68 77.44 Pb3O4

Mirabilite -10.15 -12.00 -1.85 Na2SO4:10H2O

Monteponite -6.57 9.50 16.06 CdO

Na2Cr2O7 -36.92 -47.02 -10.10 Na2Cr2O7

Na2CrO4 -21.96 -18.85 3.11 Na2CrO4

Nantokite -8.65 -15.77 -7.13 CuCl

Natron -10.72 -12.64 -1.92 Na2CO3:10H2O

Nesquehonite -3.87 -8.32 -4.45 MgCO3:3H2O

O2(g) -37.35 51.04 88.39 O2

Otavite -0.48 -12.47 -11.99 CdCO3

Pb(OH)2 -1.97 6.72 8.69 Pb(OH)2

Pb10(OH)6O(CO3)6 -55.85 -64.61 -8.76 Pb10(OH)6O(CO3)6

Pb2(OH)3Cl -8.64 0.15 8.79 Pb2(OH)3Cl

Pb2O(OH)2 -12.75 13.44 26.19 Pb2O(OH)2

Pb2O3 -22.08 38.96 61.04 Pb2O3

Pb2OCO3 -8.35 -8.53 -0.18 Pb2OCO3

Pb3(AsO4)2 -17.42 -11.62 5.80 Pb3(AsO4)2

Pb3O2CO3 -13.85 -1.81 12.05 Pb3O2CO3

Pb3O2SO4 -12.59 -1.17 11.42 Pb3O2SO4

Pb4(OH)6SO4 -15.55 5.55 21.10 Pb4(OH)6SO4

Pb4O3SO4 -17.59 5.55 23.14 Pb4O3SO4

PbCrO4 -8.45 -21.46 -13.01 PbCrO4

Pbmetal -23.04 -18.80 4.24 Pb

PbO:0.3H2O -6.26 6.72 12.98 PbO:0.33H2O

Periclase -9.33 13.65 22.99 MgO

Phosgenite -15.30 -35.11 -19.81 PbCl2:PbCO3

Plattnerite -20.11 32.24 52.35 PbO2

Portlandite -9.85 14.15 24.00 Ca(OH)2

Smithsonite -2.26 -12.11 -9.85 ZnCO3

Tenorite -0.59 7.65 8.25 CuO

Thenardite -12.41 -12.00 0.41 Na2SO4

Thermonatrite -13.38 -12.64 0.73 Na2CO3:H2O

Zincite -2.31 9.86 12.17 ZnO

Zincosite -16.17 -11.47 4.70 ZnSO4

Zn(NO3)2:6H2O -18.27 -15.19 3.09 Zn(NO3)2:6H2O

Zn(OH)2 -2.34 9.86 12.20 Zn(OH)2

Zn(OH)2(am) -3.36 9.86 13.22 Zn(OH)2

Zn(OH)2(beta) -2.67 9.86 12.53 Zn(OH)2

Zn(OH)2(epsilon) -2.43 9.86 12.29 Zn(OH)2

Zn(OH)2(gamma) -1.87 9.86 11.73 Zn(OH)2

Zn2(OH)2SO4 -9.11 -1.61 7.50 Zn2(OH)2SO4

Zn2(OH)3Cl -8.76 6.43 15.19 Zn2(OH)3Cl

Zn3(AsO4)2:2.5H2O -15.85 -2.20 13.65 Zn3(AsO4)2:2.5H2O

Zn3O(SO4)2 -34.39 -13.08 21.31 Zn3O(SO4)2

Zn4(OH)6SO4 -10.29 18.11 28.40 Zn4(OH)6SO4

Zn5(OH)8Cl2 -15.78 22.72 38.50 Zn5(OH)8Cl2

ZnCl2 -24.44 -16.72 7.72 ZnCl2

ZnCO3:1H2O -1.85 -12.11 -10.26 ZnCO3:1H2O

Znmetal -42.87 -15.66 27.21 Zn

ZnO(active) -2.15 9.86 12.01 ZnO

ZnSO4:1H2O -11.24 -11.47 -0.23 ZnSO4:1H2O

**For a gas, SI = log10(fugacity). Fugacity = pressure * phi / 1 atm.

For ideal gases, phi = 1.

Initial solution 12.

----------------------------Distribution of species----------------------------

Log Log Log mole V

Species Molality Activity Molality Activity Gamma cm?mol

OH- 2.097e-06 1.973e-06 -5.678 -5.705 -0.026 (0)

H+ 1.645e-09 1.549e-09 -8.784 -8.810 -0.026 0.00

H2O 5.551e+01 9.999e-01 1.744 -0.000 0.000 18.02

As(3) 3.795e-22

H3AsO3 3.171e-22 3.171e-22 -21.499 -21.499 0.000 (0)

H2AsO3- 6.238e-23 5.846e-23 -22.205 -22.233 -0.028 (0)

HAsO3-2 2.253e-26 1.737e-26 -25.647 -25.760 -0.113 (0)

AsO3-3 4.516e-31 2.517e-31 -30.345 -30.599 -0.254 (0)

H4AsO3+ 2.597e-31 2.434e-31 -30.586 -30.614 -0.028 (0)

As(5) 4.312e-08

HAsO4-2 4.251e-08 3.278e-08 -7.372 -7.484 -0.113 (0)

H2AsO4- 5.268e-10 4.937e-10 -9.278 -9.307 -0.028 (0)

AsO4-3 8.350e-11 4.654e-11 -10.078 -10.332 -0.254 (0)

H3AsO4 1.141e-16 1.142e-16 -15.943 -15.942 0.000 (0)

C(4) 2.154e-03

HCO3- 2.052e-03 1.934e-03 -2.688 -2.714 -0.026 (0)

CO3-2 5.455e-05 4.285e-05 -4.263 -4.368 -0.105 (0)

CaCO3 2.170e-05 2.170e-05 -4.664 -4.664 0.000 (0)

CaHCO3+ 1.120e-05 1.056e-05 -4.951 -4.976 -0.025 (0)

H2CO3 8.193e-06 8.193e-06 -5.087 -5.087 0.000 (0)

MgCO3 3.870e-06 3.870e-06 -5.412 -5.412 0.000 (0)

MgHCO3+ 2.709e-06 2.550e-06 -5.567 -5.594 -0.026 (0)

NaHCO3 1.572e-07 1.572e-07 -6.804 -6.804 0.000 (0)

NaCO3- 1.409e-07 1.329e-07 -6.851 -6.877 -0.026 (0)

CuCO3 9.770e-08 9.770e-08 -7.010 -7.010 0.000 (0)

ZnCO3 6.032e-08 6.032e-08 -7.220 -7.220 0.000 (0)

Cu(CO3)2-2 1.461e-08 1.127e-08 -7.835 -7.948 -0.113 (0)

CdCO3 8.513e-09 8.513e-09 -8.070 -8.070 0.000 (0)

PbCO3 3.960e-09 3.960e-09 -8.402 -8.402 0.000 (0)

ZnHCO3+ 1.169e-09 1.095e-09 -8.932 -8.961 -0.028 (0)

Pb(CO3)2-2 6.345e-10 4.893e-10 -9.198 -9.310 -0.113 (0)

Cd(CO3)2-2 3.506e-10 2.704e-10 -9.455 -9.568 -0.113 (0)

CuHCO3+ 3.691e-11 3.459e-11 -10.433 -10.461 -0.028 (0)

PbHCO3+ 3.450e-11 3.233e-11 -10.462 -10.490 -0.028 (0)

CdHCO3+ 2.998e-11 2.809e-11 -10.523 -10.551 -0.028 (0)

Ca 6.185e-04

Ca+2 5.726e-04 4.497e-04 -3.242 -3.347 -0.105 (0)

CaCO3 2.170e-05 2.170e-05 -4.664 -4.664 0.000 (0)

CaSO4 1.269e-05 1.269e-05 -4.897 -4.897 0.000 (0)

CaHCO3+ 1.120e-05 1.056e-05 -4.951 -4.976 -0.025 (0)

CaNO3+ 3.432e-07 3.216e-07 -6.464 -6.493 -0.028 (0)

CaOH+ 1.572e-08 1.482e-08 -7.804 -7.829 -0.025 (0)

Cd 2.045e-08

Cd+2 1.110e-08 8.717e-09 -7.955 -8.060 -0.105 (0)

CdCO3 8.513e-09 8.513e-09 -8.070 -8.070 0.000 (0)

Cd(CO3)2-2 3.506e-10 2.704e-10 -9.455 -9.568 -0.113 (0)

CdSO4 2.432e-10 2.432e-10 -9.614 -9.614 0.000 (0)

CdOH+ 1.489e-10 1.395e-10 -9.827 -9.855 -0.028 (0)

CdCl+ 3.086e-11 2.892e-11 -10.511 -10.539 -0.028 (0)

CdHCO3+ 2.998e-11 2.809e-11 -10.523 -10.551 -0.028 (0)

Cd(OH)2 1.846e-11 1.846e-11 -10.734 -10.734 0.000 (0)

CdNO3+ 9.283e-12 8.699e-12 -11.032 -11.061 -0.028 (0)

CdOHCl 5.338e-12 5.338e-12 -11.273 -11.273 0.000 (0)

Cd(SO4)2-2 7.341e-13 5.662e-13 -12.134 -12.247 -0.113 (0)

Cd(OH)3- 7.825e-15 7.333e-15 -14.107 -14.135 -0.028 (0)

CdCl2 4.100e-15 4.100e-15 -14.387 -14.387 0.000 (0)

Cd(NO3)2 5.608e-16 5.608e-16 -15.251 -15.251 0.000 (0)

Cd2OH+3 1.326e-17 7.388e-18 -16.878 -17.131 -0.254 (0)

CdCl3- 8.437e-20 7.906e-20 -19.074 -19.102 -0.028 (0)

Cd(OH)4-2 1.012e-20 7.803e-21 -19.995 -20.108 -0.113 (0)

Cl 3.770e-05

Cl- 3.770e-05 3.549e-05 -4.424 -4.450 -0.026 (0)

CdCl+ 3.086e-11 2.892e-11 -10.511 -10.539 -0.028 (0)

ZnOHCl 1.856e-11 1.856e-11 -10.731 -10.731 0.000 (0)

CdOHCl 5.338e-12 5.338e-12 -11.273 -11.273 0.000 (0)

ZnCl+ 2.065e-12 1.943e-12 -11.685 -11.711 -0.026 (0)

CuCl 7.312e-13 7.312e-13 -12.136 -12.136 0.000 (0)

PbCl+ 3.430e-14 3.215e-14 -13.465 -13.493 -0.028 (0)

CuCl+ 1.938e-14 1.824e-14 -13.713 -13.739 -0.026 (0)

CuCl2- 5.981e-15 5.630e-15 -14.223 -14.249 -0.026 (0)

CdCl2 4.100e-15 4.100e-15 -14.387 -14.387 0.000 (0)

ZnCl2 5.564e-17 5.564e-17 -16.255 -16.255 0.000 (0)

PbCl2 4.749e-18 4.749e-18 -17.323 -17.323 0.000 (0)

CrCl+2 5.602e-19 4.320e-19 -18.252 -18.364 -0.113 (0)

CuCl2 1.043e-19 1.043e-19 -18.982 -18.982 0.000 (0)

CdCl3- 8.437e-20 7.906e-20 -19.074 -19.102 -0.028 (0)

CuCl3-2 5.099e-20 4.020e-20 -19.292 -19.396 -0.103 (0)

ZnCl3- 1.563e-21 1.471e-21 -20.806 -20.832 -0.026 (0)

CrOHCl2 2.070e-22 2.070e-22 -21.684 -21.684 0.000 (0)

PbCl3- 8.496e-23 7.962e-23 -22.071 -22.099 -0.028 (0)

CrCl2+ 3.921e-25 3.674e-25 -24.407 -24.435 -0.028 (0)

CrO3Cl- 7.209e-26 6.756e-26 -25.142 -25.170 -0.028 (0)

ZnCl4-2 2.923e-26 2.304e-26 -25.534 -25.638 -0.103 (0)

CuCl3- 2.774e-26 2.611e-26 -25.557 -25.583 -0.026 (0)

PbCl4-2 1.331e-27 1.026e-27 -26.876 -26.989 -0.113 (0)

CuCl4-2 9.993e-33 7.878e-33 -32.000 -32.104 -0.103 (0)

Cr(2) 2.778e-26

Cr+2 2.778e-26 2.142e-26 -25.556 -25.669 -0.113 (0)

Cr(3) 5.769e-07

Cr(OH)3 2.823e-07 2.823e-07 -6.549 -6.549 0.000 (0)

Cr(OH)2+ 1.233e-07 1.156e-07 -6.909 -6.937 -0.028 (0)

CrO2- 9.236e-08 8.655e-08 -7.035 -7.063 -0.028 (0)

Cr(OH)4- 7.795e-08 7.305e-08 -7.108 -7.136 -0.028 (0)

Cr(OH)+2 1.001e-09 7.723e-10 -8.999 -9.112 -0.113 (0)

CrOHSO4 4.969e-12 4.969e-12 -11.304 -11.304 0.000 (0)

Cr+3 2.941e-14 1.639e-14 -13.531 -13.785 -0.254 (0)

CrSO4+ 3.018e-15 2.829e-15 -14.520 -14.548 -0.028 (0)

CrCl+2 5.602e-19 4.320e-19 -18.252 -18.364 -0.113 (0)

Cr2(OH)2SO4+2 8.510e-20 6.563e-20 -19.070 -19.183 -0.113 (0)

CrNO3+2 4.750e-20 3.663e-20 -19.323 -19.436 -0.113 (0)

Cr2(OH)2(SO4)2 5.587e-22 5.587e-22 -21.253 -21.253 0.000 (0)

CrOHCl2 2.070e-22 2.070e-22 -21.684 -21.684 0.000 (0)

CrCl2+ 3.921e-25 3.674e-25 -24.407 -24.435 -0.028 (0)

Cr(6) 4.986e-11

CrO4-2 4.963e-11 3.899e-11 -10.304 -10.409 -0.105 (0)

HCrO4- 1.998e-13 1.872e-13 -12.699 -12.728 -0.028 (0)

NaCrO4- 2.228e-14 2.088e-14 -13.652 -13.680 -0.028 (0)

KCrO4- 4.588e-15 4.300e-15 -14.338 -14.367 -0.028 (0)

H2CrO4 1.066e-22 1.066e-22 -21.972 -21.972 0.000 (0)

CrO3SO4-2 1.713e-23 1.321e-23 -22.766 -22.879 -0.113 (0)

Cr2O7-2 2.365e-24 1.824e-24 -23.626 -23.739 -0.113 (0)

CrO3Cl- 7.209e-26 6.756e-26 -25.142 -25.170 -0.028 (0)

Cu(1) 1.820e-11

Cu+ 1.746e-11 1.637e-11 -10.758 -10.786 -0.028 (0)

CuCl 7.312e-13 7.312e-13 -12.136 -12.136 0.000 (0)

CuCl2- 5.981e-15 5.630e-15 -14.223 -14.249 -0.026 (0)

CuCl3-2 5.099e-20 4.020e-20 -19.292 -19.396 -0.103 (0)

Cu(2) 1.273e-07

CuCO3 9.770e-08 9.770e-08 -7.010 -7.010 0.000 (0)

Cu(CO3)2-2 1.461e-08 1.127e-08 -7.835 -7.948 -0.113 (0)

Cu(OH)2 1.033e-08 1.033e-08 -7.986 -7.986 0.000 (0)

CuOH+ 3.934e-09 3.703e-09 -8.405 -8.431 -0.026 (0)

Cu+2 4.930e-10 3.872e-10 -9.307 -9.412 -0.105 (0)

Cu(OH)3- 1.469e-10 1.377e-10 -9.833 -9.861 -0.028 (0)

CuHCO3+ 3.691e-11 3.459e-11 -10.433 -10.461 -0.028 (0)

CuSO4 1.056e-11 1.056e-11 -10.976 -10.976 0.000 (0)

Cu2(OH)2+2 4.014e-13 3.096e-13 -12.396 -12.509 -0.113 (0)

CuNO3+ 2.874e-13 2.693e-13 -12.542 -12.570 -0.028 (0)

CuCl+ 1.938e-14 1.824e-14 -13.713 -13.739 -0.026 (0)

Cu(OH)4-2 9.135e-15 7.045e-15 -14.039 -14.152 -0.113 (0)

Cu(NO3)2 6.258e-18 6.258e-18 -17.204 -17.204 0.000 (0)

CuCl2 1.043e-19 1.043e-19 -18.982 -18.982 0.000 (0)

CuCl3- 2.774e-26 2.611e-26 -25.557 -25.583 -0.026 (0)

CuCl4-2 9.993e-33 7.878e-33 -32.000 -32.104 -0.103 (0)

H(0) 3.972e-29

H2 1.986e-29 1.987e-29 -28.702 -28.702 0.000 (0)

K 3.156e-05

K+ 3.153e-05 2.969e-05 -4.501 -4.527 -0.026 (0)

KSO4- 2.927e-08 2.759e-08 -7.534 -7.559 -0.026 (0)

KCrO4- 4.588e-15 4.300e-15 -14.338 -14.367 -0.028 (0)

Mg 1.886e-04

Mg+2 1.787e-04 1.403e-04 -3.748 -3.853 -0.105 (0)

MgCO3 3.870e-06 3.870e-06 -5.412 -5.412 0.000 (0)

MgSO4 3.233e-06 3.233e-06 -5.490 -5.490 0.000 (0)

MgHCO3+ 2.709e-06 2.550e-06 -5.567 -5.594 -0.026 (0)

MgOH+ 9.037e-08 8.527e-08 -7.044 -7.069 -0.025 (0)

N(5) 2.144e-04

NO3- 2.140e-04 2.015e-04 -3.670 -3.696 -0.026 (0)

CaNO3+ 3.432e-07 3.216e-07 -6.464 -6.493 -0.028 (0)

ZnNO3+ 1.458e-11 1.366e-11 -10.836 -10.865 -0.028 (0)

CdNO3+ 9.283e-12 8.699e-12 -11.032 -11.061 -0.028 (0)

CuNO3+ 2.874e-13 2.693e-13 -12.542 -12.570 -0.028 (0)

PbNO3+ 9.367e-14 8.778e-14 -13.028 -13.057 -0.028 (0)

Cd(NO3)2 5.608e-16 5.608e-16 -15.251 -15.251 0.000 (0)

Zn(NO3)2 4.977e-16 4.977e-16 -15.303 -15.303 0.000 (0)

Pb(NO3)2 3.609e-17 3.609e-17 -16.443 -16.443 0.000 (0)

Cu(NO3)2 6.258e-18 6.258e-18 -17.204 -17.204 0.000 (0)

CrNO3+2 4.750e-20 3.663e-20 -19.323 -19.436 -0.113 (0)

Na 1.149e-04

Na+ 1.145e-04 1.078e-04 -3.941 -3.967 -0.026 (0)

NaHCO3 1.572e-07 1.572e-07 -6.804 -6.804 0.000 (0)

NaCO3- 1.409e-07 1.329e-07 -6.851 -6.877 -0.026 (0)

NaSO4- 8.614e-08 8.120e-08 -7.065 -7.090 -0.026 (0)

NaCrO4- 2.228e-14 2.088e-14 -13.652 -13.680 -0.028 (0)

O(0) 1.207e-39

O2 6.034e-40 6.038e-40 -39.219 -39.219 0.000 (0)

Pb 5.310e-09

PbCO3 3.960e-09 3.960e-09 -8.402 -8.402 0.000 (0)

Pb(CO3)2-2 6.345e-10 4.893e-10 -9.198 -9.310 -0.113 (0)

PbOH+ 5.357e-10 5.020e-10 -9.271 -9.299 -0.028 (0)

Pb(OH)2 1.032e-10 1.032e-10 -9.986 -9.986 0.000 (0)

Pb+2 3.914e-11 3.074e-11 -10.407 -10.512 -0.105 (0)

PbHCO3+ 3.450e-11 3.233e-11 -10.462 -10.490 -0.028 (0)

PbSO4 2.158e-12 2.158e-12 -11.666 -11.666 0.000 (0)

Pb(OH)3- 7.159e-13 6.709e-13 -12.145 -12.173 -0.028 (0)

PbNO3+ 9.367e-14 8.778e-14 -13.028 -13.057 -0.028 (0)

PbCl+ 3.430e-14 3.215e-14 -13.465 -13.493 -0.028 (0)

Pb(SO4)2-2 2.416e-15 1.863e-15 -14.617 -14.730 -0.113 (0)

Pb(OH)4-2 1.385e-15 1.068e-15 -14.859 -14.971 -0.113 (0)

Pb(NO3)2 3.609e-17 3.609e-17 -16.443 -16.443 0.000 (0)

PbCl2 4.749e-18 4.749e-18 -17.323 -17.323 0.000 (0)

Pb2OH+3 4.388e-19 2.446e-19 -18.358 -18.612 -0.254 (0)

Pb3(OH)4+2 7.218e-22 5.567e-22 -21.142 -21.254 -0.113 (0)

PbCl3- 8.496e-23 7.962e-23 -22.071 -22.099 -0.028 (0)

PbCl4-2 1.331e-27 1.026e-27 -26.876 -26.989 -0.113 (0)

Pb4(OH)4+4 6.842e-28 2.420e-28 -27.165 -27.616 -0.451 (0)

S(6) 1.985e-04

SO4-2 1.825e-04 1.433e-04 -3.739 -3.844 -0.105 (0)

CaSO4 1.269e-05 1.269e-05 -4.897 -4.897 0.000 (0)

MgSO4 3.233e-06 3.233e-06 -5.490 -5.490 0.000 (0)

NaSO4- 8.614e-08 8.120e-08 -7.065 -7.090 -0.026 (0)

KSO4- 2.927e-08 2.759e-08 -7.534 -7.559 -0.026 (0)

ZnSO4 6.719e-10 6.719e-10 -9.173 -9.173 0.000 (0)

CdSO4 2.432e-10 2.432e-10 -9.614 -9.614 0.000 (0)

HSO4- 1.439e-11 1.356e-11 -10.842 -10.868 -0.026 (0)

CuSO4 1.056e-11 1.056e-11 -10.976 -10.976 0.000 (0)

CrOHSO4 4.969e-12 4.969e-12 -11.304 -11.304 0.000 (0)

PbSO4 2.158e-12 2.158e-12 -11.666 -11.666 0.000 (0)

Zn(SO4)2-2 1.242e-12 9.575e-13 -11.906 -12.019 -0.113 (0)

Cd(SO4)2-2 7.341e-13 5.662e-13 -12.134 -12.247 -0.113 (0)

CrSO4+ 3.018e-15 2.829e-15 -14.520 -14.548 -0.028 (0)

Pb(SO4)2-2 2.416e-15 1.863e-15 -14.617 -14.730 -0.113 (0)

Cr2(OH)2SO4+2 8.510e-20 6.563e-20 -19.070 -19.183 -0.113 (0)

Cr2(OH)2(SO4)2 5.587e-22 5.587e-22 -21.253 -21.253 0.000 (0)

CrO3SO4-2 1.713e-23 1.321e-23 -22.766 -22.879 -0.113 (0)

Zn 1.154e-07

ZnCO3 6.032e-08 6.032e-08 -7.220 -7.220 0.000 (0)

Zn+2 3.115e-08 2.446e-08 -7.507 -7.611 -0.105 (0)

Zn(OH)2 1.639e-08 1.639e-08 -7.786 -7.786 0.000 (0)

ZnOH+ 5.149e-09 4.826e-09 -8.288 -8.316 -0.028 (0)

ZnHCO3+ 1.169e-09 1.095e-09 -8.932 -8.961 -0.028 (0)

ZnSO4 6.719e-10 6.719e-10 -9.173 -9.173 0.000 (0)

Zn(OH)3- 5.697e-10 5.339e-10 -9.244 -9.273 -0.028 (0)

ZnOHCl 1.856e-11 1.856e-11 -10.731 -10.731 0.000 (0)

ZnNO3+ 1.458e-11 1.366e-11 -10.836 -10.865 -0.028 (0)

ZnCl+ 2.065e-12 1.943e-12 -11.685 -11.711 -0.026 (0)

Zn(SO4)2-2 1.242e-12 9.575e-13 -11.906 -12.019 -0.113 (0)

Zn(OH)4-2 1.792e-13 1.382e-13 -12.747 -12.860 -0.113 (0)

Zn(NO3)2 4.977e-16 4.977e-16 -15.303 -15.303 0.000 (0)

ZnCl2 5.564e-17 5.564e-17 -16.255 -16.255 0.000 (0)

ZnCl3- 1.563e-21 1.471e-21 -20.806 -20.832 -0.026 (0)

ZnCl4-2 2.923e-26 2.304e-26 -25.534 -25.638 -0.103 (0)

------------------------------Saturation indices-------------------------------

Phase SI** log IAP log K(283 K, 1 atm)

Anglesite -6.45 -14.36 -7.90 PbSO4

Anhydrite -2.90 -7.19 -4.29 CaSO4

Antlerite -5.63 3.16 8.79 Cu3(OH)4SO4

Aragonite 0.47 -7.72 -8.19 CaCO3

Arsenolite -82.68 -85.99 -3.32 As4O6

Artinite -5.17 5.55 10.72 MgCO3:Mg(OH)2:3H2O

As2O5 -38.80 -31.88 6.92 As2O5

Atacamite -5.10 3.16 8.26 Cu2(OH)3Cl

Azurite -3.33 -19.35 -16.02 Cu3(OH)2(CO3)2

Bianchite -9.70 -11.46 -1.76 ZnSO4:6H2O

Brochantite -5.74 11.37 17.10 Cu4(OH)6SO4

Brucite -4.13 13.77 17.90 Mg(OH)2

Ca3(AsO4)2:4H2O -11.37 10.93 22.30 Ca3(AsO4)2:4H2O

CaCrO4 -11.74 -13.76 -2.02 CaCrO4

Calcite 0.69 -7.72 -8.41 CaCO3

Cd(OH)2 -4.96 9.56 14.52 Cd(OH)2

Cd(OH)2(am) -4.98 9.56 14.54 Cd(OH)2

Cd3(OH)2(SO4)2 -20.96 -14.25 6.71 Cd3(OH)2(SO4)2

Cd3(OH)4SO4 -15.34 7.22 22.56 Cd3(OH)4SO4

Cd4(OH)6SO4 -11.62 16.78 28.40 Cd4(OH)6SO4

CdCl2 -16.47 -16.96 -0.49 CdCl2

CdCl2:1H2O -15.34 -16.96 -1.62 CdCl2:1H2O

CdCl2:2.5H2O -14.98 -16.96 -1.98 CdCl2:2.5H2O

Cdmetal(alpha) -30.27 -16.06 14.21 Cd

Cdmetal(gamma) -30.38 -16.06 14.32 Cd

CdOHCl -7.52 -3.70 3.82 CdOHCl

CdSO4 -12.21 -11.90 0.31 CdSO4

CdSO4:1H2O -10.47 -11.90 -1.43 CdSO4:1H2O

CdSO4:2.67H2O -10.20 -11.90 -1.71 CdSO4:2.67H2O

Cerussite -1.52 -14.88 -13.36 PbCO3

CH4(g) -81.04 -124.47 -43.43 CH4

Chalcanthite -10.56 -13.26 -2.70 CuSO4:5H2O

Claudetite -82.41 -85.99 -3.58 As4O6

CO2(g) -3.80 -21.99 -18.18 CO2

Cotunnite -14.39 -19.41 -5.02 PbCl2

Cr(OH)2 -19.20 -8.05 11.15 Cr(OH)2

Cr(OH)3 0.26 1.87 1.61 Cr(OH)3

Cr(OH)3(am) 2.62 1.87 -0.75 Cr(OH)3

Cr2O3 5.63 3.75 -1.89 Cr2O3

CrCl2 -49.69 -34.57 15.12 CrCl2

CrCl3 -54.14 -37.91 16.24 CrCl3

Crmetal -65.75 -33.67 32.08 Cr

CrO3 -24.87 -28.03 -3.16 CrO3

Cu(OH)2 -0.99 8.21 9.20 Cu(OH)2

Cu2(OH)3NO3 -6.01 3.91 9.92 Cu2(OH)3NO3

Cu2SO4 -23.64 -25.42 -1.77 Cu2SO4

Cu3(AsO4)2:2H2O -13.36 -7.26 6.10 Cu3(AsO4)2:2H2O

CuCO3 -2.28 -13.78 -11.50 CuCO3

CuCrO4 -14.38 -19.82 -5.44 CuCrO4

Cumetal -5.36 -14.79 -9.42 Cu

CuOCuSO4 -16.63 -5.05 11.58 CuOCuSO4

Cuprite -3.70 -3.95 -0.26 Cu2O

CuSO4 -16.87 -13.26 3.62 CuSO4

Dolomite(disordered) 0.17 -15.94 -16.11 CaMg(CO3)2

Dolomite(ordered) 0.79 -15.94 -16.72 CaMg(CO3)2

Epsomite -5.46 -7.70 -2.23 MgSO4:7H2O

Goslarite -9.31 -11.46 -2.14 ZnSO4:7H2O

Gypsum -2.57 -7.19 -4.62 CaSO4:2H2O

Halite -9.99 -8.42 1.57 NaCl

Huntite -3.41 -32.38 -28.97 CaMg3(CO3)4

Hydrocerussite -3.88 -22.65 -18.77 Pb3(OH)2(CO3)2

Hydromagnesite -12.38 -19.12 -6.74 Mg5(CO3)4(OH)2:4H2O

K2Cr2O7 -29.50 -47.49 -17.99 K2Cr2O7

K2CrO4 -18.78 -19.46 -0.68 K2CrO4

Langite -7.66 11.37 19.03 Cu4(OH)6SO4:H2O

Larnakite -7.02 -7.25 -0.23 PbO:PbSO4

Laurionite -6.78 -6.15 0.62 PbOHCl

Lime -20.23 14.27 34.50 CaO

Litharge -6.19 7.11 13.30 PbO

Magnesite -0.58 -8.22 -7.65 MgCO3

Malachite 0.44 -5.57 -6.01 Cu2(OH)2CO3

Massicot -6.41 7.11 13.51 PbO

Melanothallite -25.16 -18.31 6.85 CuCl2

Mg(OH)2(active) -5.03 13.77 18.79 Mg(OH)2

MgCr2O4 -0.35 17.51 17.87 MgCr2O4

MgCrO4 -20.47 -14.26 6.21 MgCrO4

Minium -30.49 46.94 77.44 Pb3O4

Mirabilite -9.93 -11.78 -1.85 Na2SO4:10H2O

Monteponite -6.50 9.56 16.06 CdO

Na2Cr2O7 -36.27 -46.37 -10.10 Na2Cr2O7

Na2CrO4 -21.46 -18.34 3.11 Na2CrO4

Nantokite -8.11 -15.24 -7.13 CuCl

Natron -10.38 -12.30 -1.92 Na2CO3:10H2O

Nesquehonite -3.78 -8.22 -4.45 MgCO3:3H2O

O2(g) -37.15 51.24 88.39 O2

Otavite -0.43 -12.43 -11.99 CdCO3

Pb(OH)2 -1.59 7.11 8.69 Pb(OH)2

Pb10(OH)6O(CO3)6 -52.09 -60.85 -8.76 Pb10(OH)6O(CO3)6

Pb2(OH)3Cl -7.84 0.96 8.79 Pb2(OH)3Cl

Pb2O(OH)2 -11.97 14.22 26.19 Pb2O(OH)2

Pb2O3 -21.20 39.84 61.04 Pb2O3

Pb2OCO3 -7.59 -7.77 -0.18 Pb2OCO3

Pb3(AsO4)2 -16.36 -10.56 5.80 Pb3(AsO4)2

Pb3O2CO3 -12.71 -0.66 12.05 Pb3O2CO3

Pb3O2SO4 -11.56 -0.14 11.42 Pb3O2SO4

Pb4(OH)6SO4 -14.13 6.97 21.10 Pb4(OH)6SO4

Pb4O3SO4 -16.18 6.97 23.14 Pb4O3SO4

PbCrO4 -7.91 -20.92 -13.01 PbCrO4

Pbmetal -22.75 -18.51 4.24 Pb

PbO:0.3H2O -5.87 7.11 12.98 PbO:0.33H2O

Periclase -9.22 13.77 22.99 MgO

Phosgenite -14.48 -34.29 -19.81 PbCl2:PbCO3

Plattnerite -19.62 32.73 52.35 PbO2

Portlandite -9.72 14.27 24.00 Ca(OH)2

Smithsonite -2.13 -11.98 -9.85 ZnCO3

Tenorite -0.04 8.21 8.25 CuO

Thenardite -12.18 -11.78 0.41 Na2SO4

Thermonatrite -13.04 -12.30 0.73 Na2CO3:H2O

Zincite -2.16 10.01 12.17 ZnO

Zincosite -16.15 -11.46 4.70 ZnSO4

Zn(NO3)2:6H2O -18.09 -15.00 3.09 Zn(NO3)2:6H2O

Zn(OH)2 -2.19 10.01 12.20 Zn(OH)2

Zn(OH)2(am) -3.21 10.01 13.22 Zn(OH)2

Zn(OH)2(beta) -2.52 10.01 12.53 Zn(OH)2

Zn(OH)2(epsilon) -2.28 10.01 12.29 Zn(OH)2

Zn(OH)2(gamma) -1.73 10.01 11.73 Zn(OH)2

Zn2(OH)2SO4 -8.95 -1.45 7.50 Zn2(OH)2SO4

Zn2(OH)3Cl -8.43 6.76 15.19 Zn2(OH)3Cl

Zn3(AsO4)2:2.5H2O -15.51 -1.86 13.65 Zn3(AsO4)2:2.5H2O

Zn3O(SO4)2 -34.21 -12.90 21.31 Zn3O(SO4)2

Zn4(OH)6SO4 -9.83 18.57 28.40 Zn4(OH)6SO4

Zn5(OH)8Cl2 -14.98 23.52 38.50 Zn5(OH)8Cl2

ZnCl2 -24.23 -16.51 7.72 ZnCl2

ZnCO3:1H2O -1.72 -11.98 -10.26 ZnCO3:1H2O

Znmetal -42.82 -15.61 27.21 Zn

ZnO(active) -2.00 10.01 12.01 ZnO

ZnSO4:1H2O -11.23 -11.46 -0.23 ZnSO4:1H2O

**For a gas, SI = log10(fugacity). Fugacity = pressure * phi / 1 atm.

For ideal gases, phi = 1.

Initial solution 13.

----------------------------Distribution of species----------------------------

Log Log Log mole V

Species Molality Activity Molality Activity Gamma cm?mol

OH- 9.528e-07 9.016e-07 -6.021 -6.045 -0.024 (0)

H+ 3.579e-09 3.388e-09 -8.446 -8.470 -0.024 0.00

H2O 5.551e+01 1.000e+00 1.744 -0.000 0.000 18.02

As(3) 4.445e-21

H3AsO3 4.080e-21 4.080e-21 -20.389 -20.389 0.000 (0)

H2AsO3- 3.645e-22 3.438e-22 -21.438 -21.464 -0.025 (0)

HAsO3-2 5.900e-26 4.670e-26 -25.229 -25.331 -0.102 (0)

H4AsO3+ 7.262e-30 6.850e-30 -29.139 -29.164 -0.025 (0)

AsO3-3 5.234e-31 3.093e-31 -30.281 -30.510 -0.228 (0)

As(5) 2.392e-08

HAsO4-2 2.326e-08 1.841e-08 -7.633 -7.735 -0.102 (0)

H2AsO4- 6.431e-10 6.066e-10 -9.192 -9.217 -0.025 (0)

AsO4-3 2.022e-11 1.195e-11 -10.694 -10.923 -0.228 (0)

H3AsO4 3.067e-16 3.069e-16 -15.513 -15.513 0.000 (0)

C(4) 1.311e-03

HCO3- 1.272e-03 1.206e-03 -2.895 -2.919 -0.023 (0)

CO3-2 1.520e-05 1.221e-05 -4.818 -4.913 -0.095 (0)

H2CO3 1.118e-05 1.118e-05 -4.952 -4.952 0.000 (0)

CaHCO3+ 5.926e-06 5.619e-06 -5.227 -5.250 -0.023 (0)

CaCO3 5.276e-06 5.276e-06 -5.278 -5.278 0.000 (0)

MgHCO3+ 7.160e-07 6.778e-07 -6.145 -6.169 -0.024 (0)

MgCO3 4.703e-07 4.703e-07 -6.328 -6.328 0.000 (0)

NaHCO3 1.377e-07 1.377e-07 -6.861 -6.861 0.000 (0)

NaCO3- 5.614e-08 5.321e-08 -7.251 -7.274 -0.023 (0)

ZnCO3 1.055e-08 1.055e-08 -7.977 -7.977 0.000 (0)

CuCO3 4.914e-09 4.914e-09 -8.309 -8.309 0.000 (0)

CdCO3 1.220e-09 1.220e-09 -8.914 -8.914 0.000 (0)

PbCO3 6.326e-10 6.326e-10 -9.199 -9.199 0.000 (0)

ZnHCO3+ 4.444e-10 4.191e-10 -9.352 -9.378 -0.025 (0)

Cu(CO3)2-2 2.041e-10 1.615e-10 -9.690 -9.792 -0.102 (0)

Pb(CO3)2-2 2.815e-11 2.228e-11 -10.550 -10.652 -0.102 (0)

Cd(CO3)2-2 1.395e-11 1.104e-11 -10.855 -10.957 -0.102 (0)

PbHCO3+ 1.198e-11 1.130e-11 -10.921 -10.947 -0.025 (0)

CdHCO3+ 9.336e-12 8.806e-12 -11.030 -11.055 -0.025 (0)

CuHCO3+ 4.034e-12 3.805e-12 -11.394 -11.420 -0.025 (0)

Ca 5.066e-04

Ca+2 4.775e-04 3.836e-04 -3.321 -3.416 -0.095 (0)

CaSO4 1.753e-05 1.753e-05 -4.756 -4.756 0.000 (0)

CaHCO3+ 5.926e-06 5.619e-06 -5.227 -5.250 -0.023 (0)

CaCO3 5.276e-06 5.276e-06 -5.278 -5.278 0.000 (0)

CaNO3+ 3.538e-07 3.338e-07 -6.451 -6.477 -0.025 (0)

CaOH+ 6.095e-09 5.780e-09 -8.215 -8.238 -0.023 (0)

Cd 6.962e-09

Cd+2 5.454e-09 4.382e-09 -8.263 -8.358 -0.095 (0)

CdCO3 1.220e-09 1.220e-09 -8.914 -8.914 0.000 (0)

CdSO4 1.979e-10 1.979e-10 -9.703 -9.703 0.000 (0)

CdOH+ 3.399e-11 3.206e-11 -10.469 -10.494 -0.025 (0)

CdCl+ 2.315e-11 2.183e-11 -10.636 -10.661 -0.025 (0)

Cd(CO3)2-2 1.395e-11 1.104e-11 -10.855 -10.957 -0.102 (0)

CdHCO3+ 9.336e-12 8.806e-12 -11.030 -11.055 -0.025 (0)

CdNO3+ 5.640e-12 5.320e-12 -11.249 -11.274 -0.025 (0)

Cd(OH)2 1.939e-12 1.939e-12 -11.712 -11.712 0.000 (0)

CdOHCl 1.842e-12 1.842e-12 -11.735 -11.735 0.000 (0)

Cd(SO4)2-2 9.429e-13 7.463e-13 -12.026 -12.127 -0.102 (0)

CdCl2 4.648e-15 4.648e-15 -14.333 -14.333 0.000 (0)

Cd(NO3)2 4.173e-16 4.173e-16 -15.380 -15.380 0.000 (0)

Cd(OH)3- 3.732e-16 3.520e-16 -15.428 -15.453 -0.025 (0)

Cd2OH+3 1.444e-18 8.533e-19 -17.840 -18.069 -0.228 (0)

CdCl3- 1.427e-19 1.346e-19 -18.846 -18.871 -0.025 (0)

Cd(OH)4-2 2.163e-22 1.712e-22 -21.665 -21.766 -0.102 (0)

Cl 5.630e-05

Cl- 5.630e-05 5.330e-05 -4.250 -4.273 -0.024 (0)

CdCl+ 2.315e-11 2.183e-11 -10.636 -10.661 -0.025 (0)

ZnOHCl 7.820e-12 7.820e-12 -11.107 -11.107 0.000 (0)

ZnCl+ 1.892e-12 1.791e-12 -11.723 -11.747 -0.024 (0)

CdOHCl 1.842e-12 1.842e-12 -11.735 -11.735 0.000 (0)

CuCl 1.937e-13 1.937e-13 -12.713 -12.713 0.000 (0)

PbCl+ 2.868e-14 2.706e-14 -13.542 -13.568 -0.025 (0)

CuCl+ 5.106e-15 4.833e-15 -14.292 -14.316 -0.024 (0)

CdCl2 4.648e-15 4.648e-15 -14.333 -14.333 0.000 (0)

CuCl2- 2.366e-15 2.240e-15 -14.626 -14.650 -0.024 (0)

ZnCl2 7.701e-17 7.701e-17 -16.113 -16.113 0.000 (0)

PbCl2 6.003e-18 6.003e-18 -17.222 -17.222 0.000 (0)

CrCl+2 5.220e-18 4.132e-18 -17.282 -17.384 -0.102 (0)

CdCl3- 1.427e-19 1.346e-19 -18.846 -18.871 -0.025 (0)

CuCl2 4.149e-20 4.149e-20 -19.382 -19.382 0.000 (0)

CuCl3-2 2.981e-20 2.402e-20 -19.526 -19.619 -0.094 (0)

ZnCl3- 3.230e-21 3.058e-21 -20.491 -20.515 -0.024 (0)

CrOHCl2 1.359e-21 1.359e-21 -20.867 -20.867 0.000 (0)

PbCl3- 1.602e-22 1.511e-22 -21.795 -21.821 -0.025 (0)

CrCl2+ 5.595e-24 5.277e-24 -23.252 -23.278 -0.025 (0)

ZnCl4-2 8.924e-26 7.191e-26 -25.049 -25.143 -0.094 (0)

CuCl3- 1.648e-26 1.560e-26 -25.783 -25.807 -0.024 (0)

CrO3Cl- 6.248e-27 5.893e-27 -26.204 -26.230 -0.025 (0)

PbCl4-2 3.695e-27 2.925e-27 -26.432 -26.534 -0.102 (0)

CuCl4-2 8.772e-33 7.069e-33 -32.057 -32.151 -0.094 (0)

Cr(2) 1.724e-25

Cr+2 1.724e-25 1.364e-25 -24.763 -24.865 -0.102 (0)

Cr(3) 3.846e-07

Cr(OH)3 1.717e-07 1.717e-07 -6.765 -6.765 0.000 (0)

Cr(OH)2+ 1.631e-07 1.538e-07 -6.788 -6.813 -0.025 (0)

CrO2- 2.551e-08 2.406e-08 -7.593 -7.619 -0.025 (0)

Cr(OH)4- 2.153e-08 2.031e-08 -7.667 -7.692 -0.025 (0)

Cr(OH)+2 2.840e-09 2.248e-09 -8.547 -8.648 -0.102 (0)

CrOHSO4 2.343e-11 2.343e-11 -10.630 -10.630 0.000 (0)

Cr+3 1.767e-13 1.044e-13 -12.753 -12.981 -0.228 (0)

CrSO4+ 3.093e-14 2.917e-14 -13.510 -13.535 -0.025 (0)

CrCl+2 5.220e-18 4.132e-18 -17.282 -17.384 -0.102 (0)

Cr2(OH)2SO4+2 1.138e-18 9.007e-19 -17.944 -18.045 -0.102 (0)

CrNO3+2 3.586e-19 2.838e-19 -18.445 -18.547 -0.102 (0)

Cr2(OH)2(SO4)2 1.242e-20 1.242e-20 -19.906 -19.906 0.000 (0)

CrOHCl2 1.359e-21 1.359e-21 -20.867 -20.867 0.000 (0)

CrCl2+ 5.595e-24 5.277e-24 -23.252 -23.278 -0.025 (0)

Cr(6) 5.946e-13

CrO4-2 5.889e-13 4.732e-13 -12.230 -12.325 -0.095 (0)

HCrO4- 5.270e-15 4.971e-15 -14.278 -14.304 -0.025 (0)

NaCrO4- 3.775e-16 3.561e-16 -15.423 -15.448 -0.025 (0)

KCrO4- 3.384e-17 3.192e-17 -16.471 -16.496 -0.025 (0)

H2CrO4 6.192e-24 6.192e-24 -23.208 -23.208 0.000 (0)

CrO3SO4-2 1.570e-24 1.243e-24 -23.804 -23.906 -0.102 (0)

CrO3Cl- 6.248e-27 5.893e-27 -26.204 -26.230 -0.025 (0)

Cr2O7-2 1.625e-27 1.286e-27 -26.789 -26.891 -0.102 (0)

Cu(1) 3.257e-12

Cu+ 3.061e-12 2.887e-12 -11.514 -11.539 -0.025 (0)

CuCl 1.937e-13 1.937e-13 -12.713 -12.713 0.000 (0)

CuCl2- 2.366e-15 2.240e-15 -14.626 -14.650 -0.024 (0)

CuCl3-2 2.981e-20 2.402e-20 -19.526 -19.619 -0.094 (0)

Cu(2) 5.908e-09

CuCO3 4.914e-09 4.914e-09 -8.309 -8.309 0.000 (0)

Cu(OH)2 3.806e-10 3.806e-10 -9.419 -9.419 0.000 (0)

CuOH+ 3.155e-10 2.987e-10 -9.501 -9.525 -0.024 (0)

Cu(CO3)2-2 2.041e-10 1.615e-10 -9.690 -9.792 -0.102 (0)

Cu+2 8.504e-11 6.832e-11 -10.070 -10.165 -0.095 (0)

CuHCO3+ 4.034e-12 3.805e-12 -11.394 -11.420 -0.025 (0)

CuSO4 3.016e-12 3.016e-12 -11.521 -11.521 0.000 (0)

Cu(OH)3- 2.460e-12 2.320e-12 -11.609 -11.635 -0.025 (0)

CuNO3+ 6.129e-14 5.781e-14 -13.213 -13.238 -0.025 (0)

CuCl+ 5.106e-15 4.833e-15 -14.292 -14.316 -0.024 (0)

Cu2(OH)2+2 2.544e-15 2.014e-15 -14.594 -14.696 -0.102 (0)

Cu(OH)4-2 6.855e-17 5.426e-17 -16.164 -16.266 -0.102 (0)

Cu(NO3)2 1.634e-18 1.634e-18 -17.787 -17.787 0.000 (0)

CuCl2 4.149e-20 4.149e-20 -19.382 -19.382 0.000 (0)

CuCl3- 1.648e-26 1.560e-26 -25.783 -25.807 -0.024 (0)

CuCl4-2 8.772e-33 7.069e-33 -32.057 -32.151 -0.094 (0)

H(0) 1.901e-28

H2 9.507e-29 9.513e-29 -28.022 -28.022 0.000 (0)

K 1.921e-05

K+ 1.918e-05 1.816e-05 -4.717 -4.741 -0.024 (0)

KSO4- 2.884e-08 2.733e-08 -7.540 -7.563 -0.023 (0)

KCrO4- 3.384e-17 3.192e-17 -16.471 -16.496 -0.025 (0)

Mg 7.790e-05

Mg+2 7.447e-05 5.983e-05 -4.128 -4.223 -0.095 (0)

MgSO4 2.232e-06 2.232e-06 -5.651 -5.651 0.000 (0)

MgHCO3+ 7.160e-07 6.778e-07 -6.145 -6.169 -0.024 (0)

MgCO3 4.703e-07 4.703e-07 -6.328 -6.328 0.000 (0)

MgOH+ 1.752e-08 1.662e-08 -7.757 -7.779 -0.023 (0)

N(5) 2.593e-04

NO3- 2.589e-04 2.451e-04 -3.587 -3.611 -0.024 (0)

CaNO3+ 3.538e-07 3.338e-07 -6.451 -6.477 -0.025 (0)

ZnNO3+ 1.081e-11 1.020e-11 -10.966 -10.991 -0.025 (0)

CdNO3+ 5.640e-12 5.320e-12 -11.249 -11.274 -0.025 (0)

PbNO3+ 6.346e-14 5.986e-14 -13.198 -13.223 -0.025 (0)

CuNO3+ 6.129e-14 5.781e-14 -13.213 -13.238 -0.025 (0)

Zn(NO3)2 4.521e-16 4.521e-16 -15.345 -15.345 0.000 (0)

Cd(NO3)2 4.173e-16 4.173e-16 -15.380 -15.380 0.000 (0)

Pb(NO3)2 2.994e-17 2.994e-17 -16.524 -16.524 0.000 (0)

Cu(NO3)2 1.634e-18 1.634e-18 -17.787 -17.787 0.000 (0)

CrNO3+2 3.586e-19 2.838e-19 -18.445 -18.547 -0.102 (0)

Na 1.604e-04

Na+ 1.600e-04 1.515e-04 -3.796 -3.820 -0.024 (0)

NaSO4- 1.950e-07 1.848e-07 -6.710 -6.733 -0.023 (0)

NaHCO3 1.377e-07 1.377e-07 -6.861 -6.861 0.000 (0)

NaCO3- 5.614e-08 5.321e-08 -7.251 -7.274 -0.023 (0)

NaCrO4- 3.775e-16 3.561e-16 -15.423 -15.448 -0.025 (0)

O(0) 0.000e+00

O2 0.000e+00 0.000e+00 -40.579 -40.579 0.000 (0)

Pb 8.447e-10

PbCO3 6.326e-10 6.326e-10 -9.199 -9.199 0.000 (0)

PbOH+ 1.363e-10 1.286e-10 -9.865 -9.891 -0.025 (0)

Pb(CO3)2-2 2.815e-11 2.228e-11 -10.550 -10.652 -0.102 (0)

Pb+2 2.145e-11 1.723e-11 -10.669 -10.764 -0.095 (0)

Pb(OH)2 1.208e-11 1.208e-11 -10.918 -10.918 0.000 (0)

PbHCO3+ 1.198e-11 1.130e-11 -10.921 -10.947 -0.025 (0)

PbSO4 1.958e-12 1.958e-12 -11.708 -11.708 0.000 (0)

PbNO3+ 6.346e-14 5.986e-14 -13.198 -13.223 -0.025 (0)

Pb(OH)3- 3.807e-14 3.591e-14 -13.419 -13.445 -0.025 (0)

PbCl+ 2.868e-14 2.706e-14 -13.542 -13.568 -0.025 (0)

Pb(SO4)2-2 3.460e-15 2.739e-15 -14.461 -14.562 -0.102 (0)

Pb(OH)4-2 3.302e-17 2.613e-17 -16.481 -16.583 -0.102 (0)

Pb(NO3)2 2.994e-17 2.994e-17 -16.524 -16.524 0.000 (0)

PbCl2 6.003e-18 6.003e-18 -17.222 -17.222 0.000 (0)

Pb2OH+3 5.943e-20 3.512e-20 -19.226 -19.454 -0.228 (0)

PbCl3- 1.602e-22 1.511e-22 -21.795 -21.821 -0.025 (0)

Pb3(OH)4+2 5.405e-24 4.278e-24 -23.267 -23.369 -0.102 (0)

PbCl4-2 3.695e-27 2.925e-27 -26.432 -26.534 -0.102 (0)

Pb4(OH)4+4 2.656e-30 1.043e-30 -29.576 -29.982 -0.406 (0)

S(6) 3.089e-04

SO4-2 2.889e-04 2.321e-04 -3.539 -3.634 -0.095 (0)

CaSO4 1.753e-05 1.753e-05 -4.756 -4.756 0.000 (0)

MgSO4 2.232e-06 2.232e-06 -5.651 -5.651 0.000 (0)

NaSO4- 1.950e-07 1.848e-07 -6.710 -6.733 -0.023 (0)

KSO4- 2.884e-08 2.733e-08 -7.540 -7.563 -0.023 (0)

ZnSO4 6.677e-10 6.677e-10 -9.175 -9.175 0.000 (0)

CdSO4 1.979e-10 1.979e-10 -9.703 -9.703 0.000 (0)

HSO4- 5.071e-11 4.802e-11 -10.295 -10.319 -0.024 (0)

CrOHSO4 2.343e-11 2.343e-11 -10.630 -10.630 0.000 (0)

CuSO4 3.016e-12 3.016e-12 -11.521 -11.521 0.000 (0)

PbSO4 1.958e-12 1.958e-12 -11.708 -11.708 0.000 (0)

Zn(SO4)2-2 1.947e-12 1.541e-12 -11.711 -11.812 -0.102 (0)

Cd(SO4)2-2 9.429e-13 7.463e-13 -12.026 -12.127 -0.102 (0)

CrSO4+ 3.093e-14 2.917e-14 -13.510 -13.535 -0.025 (0)

Pb(SO4)2-2 3.460e-15 2.739e-15 -14.461 -14.562 -0.102 (0)

Cr2(OH)2SO4+2 1.138e-18 9.007e-19 -17.944 -18.045 -0.102 (0)

Cr2(OH)2(SO4)2 1.242e-20 1.242e-20 -19.906 -19.906 0.000 (0)

CrO3SO4-2 1.570e-24 1.243e-24 -23.804 -23.906 -0.102 (0)

Zn 3.394e-08

Zn+2 1.869e-08 1.501e-08 -7.728 -7.824 -0.095 (0)

ZnCO3 1.055e-08 1.055e-08 -7.977 -7.977 0.000 (0)

Zn(OH)2 2.101e-09 2.101e-09 -8.678 -8.678 0.000 (0)

ZnOH+ 1.435e-09 1.354e-09 -8.843 -8.868 -0.025 (0)

ZnSO4 6.677e-10 6.677e-10 -9.175 -9.175 0.000 (0)

ZnHCO3+ 4.444e-10 4.191e-10 -9.352 -9.378 -0.025 (0)

Zn(OH)3- 3.318e-11 3.129e-11 -10.479 -10.505 -0.025 (0)

ZnNO3+ 1.081e-11 1.020e-11 -10.966 -10.991 -0.025 (0)

ZnOHCl 7.820e-12 7.820e-12 -11.107 -11.107 0.000 (0)

Zn(SO4)2-2 1.947e-12 1.541e-12 -11.711 -11.812 -0.102 (0)

ZnCl+ 1.892e-12 1.791e-12 -11.723 -11.747 -0.024 (0)

Zn(OH)4-2 4.677e-15 3.702e-15 -14.330 -14.432 -0.102 (0)

Zn(NO3)2 4.521e-16 4.521e-16 -15.345 -15.345 0.000 (0)

ZnCl2 7.701e-17 7.701e-17 -16.113 -16.113 0.000 (0)

ZnCl3- 3.230e-21 3.058e-21 -20.491 -20.515 -0.024 (0)

ZnCl4-2 8.924e-26 7.191e-26 -25.049 -25.143 -0.094 (0)

------------------------------Saturation indices-------------------------------

Phase SI** log IAP log K(283 K, 1 atm)

Anglesite -6.50 -14.40 -7.90 PbSO4

Anhydrite -2.76 -7.05 -4.29 CaSO4

Antlerite -9.04 -0.25 8.79 Cu3(OH)4SO4

Aragonite -0.14 -8.33 -8.19 CaCO3

Arsenolite -78.24 -81.56 -3.32 As4O6

Artinite -7.14 3.58 10.72 MgCO3:Mg(OH)2:3H2O

As2O5 -37.94 -31.03 6.92 As2O5

Atacamite -7.45 0.81 8.26 Cu2(OH)3Cl

Azurite -7.36 -23.38 -16.02 Cu3(OH)2(CO3)2

Bianchite -9.70 -11.46 -1.76 ZnSO4:6H2O

Brochantite -10.58 6.52 17.10 Cu4(OH)6SO4

Brucite -5.19 12.72 17.90 Mg(OH)2

Ca3(AsO4)2:4H2O -12.75 9.55 22.30 Ca3(AsO4)2:4H2O

CaCrO4 -13.73 -15.74 -2.02 CaCrO4

Calcite 0.08 -8.33 -8.41 CaCO3

Cd(OH)2 -5.94 8.58 14.52 Cd(OH)2

Cd(OH)2(am) -5.95 8.58 14.54 Cd(OH)2

Cd3(OH)2(SO4)2 -22.11 -15.40 6.71 Cd3(OH)2(SO4)2

Cd3(OH)4SO4 -17.39 5.17 22.56 Cd3(OH)4SO4

Cd4(OH)6SO4 -14.65 13.75 28.40 Cd4(OH)6SO4

CdCl2 -16.42 -16.90 -0.49 CdCl2

CdCl2:1H2O -15.28 -16.90 -1.62 CdCl2:1H2O

CdCl2:2.5H2O -14.92 -16.90 -1.98 CdCl2:2.5H2O

Cdmetal(alpha) -30.57 -16.36 14.21 Cd

Cdmetal(gamma) -30.68 -16.36 14.32 Cd

CdOHCl -7.99 -4.16 3.82 CdOHCl

CdSO4 -12.30 -11.99 0.31 CdSO4

CdSO4:1H2O -10.56 -11.99 -1.43 CdSO4:1H2O

CdSO4:2.67H2O -10.29 -11.99 -1.71 CdSO4:2.67H2O

Cerussite -2.32 -15.68 -13.36 PbCO3

CH4(g) -78.18 -121.61 -43.43 CH4

Chalcanthite -11.10 -13.80 -2.70 CuSO4:5H2O

Claudetite -77.98 -81.56 -3.58 As4O6

CO2(g) -3.67 -21.85 -18.18 CO2

Cotunnite -14.29 -19.31 -5.02 PbCl2

Cr(OH)2 -19.07 -7.93 11.15 Cr(OH)2

Cr(OH)3 0.05 1.66 1.61 Cr(OH)3

Cr(OH)3(am) 2.41 1.66 -0.75 Cr(OH)3

Cr2O3 5.20 3.31 -1.89 Cr2O3

CrCl2 -48.53 -33.41 15.12 CrCl2

CrCl3 -52.81 -36.57 16.24 CrCl3

Crmetal -64.94 -32.87 32.08 Cr

CrO3 -26.10 -29.26 -3.16 CrO3

Cu(OH)2 -2.42 6.77 9.20 Cu(OH)2

Cu2(OH)3NO3 -8.46 1.47 9.92 Cu2(OH)3NO3

Cu2SO4 -24.94 -26.71 -1.77 Cu2SO4

Cu3(AsO4)2:2H2O -16.80 -10.70 6.10 Cu3(AsO4)2:2H2O

CuCO3 -3.58 -15.08 -11.50 CuCO3

CuCrO4 -17.05 -22.49 -5.44 CuCrO4

Cumetal -6.12 -15.54 -9.42 Cu

CuOCuSO4 -18.61 -7.03 11.58 CuOCuSO4

Cuprite -5.88 -6.14 -0.26 Cu2O

CuSO4 -17.42 -13.80 3.62 CuSO4

Dolomite(disordered) -1.36 -17.47 -16.11 CaMg(CO3)2

Dolomite(ordered) -0.74 -17.47 -16.72 CaMg(CO3)2

Epsomite -5.62 -7.86 -2.23 MgSO4:7H2O

Goslarite -9.31 -11.46 -2.14 ZnSO4:7H2O

Gypsum -2.43 -7.05 -4.62 CaSO4:2H2O

Halite -9.66 -8.09 1.57 NaCl

Huntite -6.77 -35.74 -28.97 CaMg3(CO3)4

Hydrocerussite -6.41 -25.18 -18.77 Pb3(OH)2(CO3)2

Hydromagnesite -17.09 -23.83 -6.74 Mg5(CO3)4(OH)2:4H2O

K2Cr2O7 -33.08 -51.07 -17.99 K2Cr2O7

K2CrO4 -21.12 -21.81 -0.68 K2CrO4

Langite -12.50 6.52 19.03 Cu4(OH)6SO4:H2O

Larnakite -7.99 -8.22 -0.23 PbO:PbSO4

Laurionite -7.19 -6.57 0.62 PbOHCl

Lime -20.98 13.52 34.50 CaO

Litharge -7.13 6.18 13.30 PbO

Magnesite -1.49 -9.14 -7.65 MgCO3

Malachite -2.29 -8.30 -6.01 Cu2(OH)2CO3

Massicot -7.34 6.18 13.51 PbO

Melanothallite -25.56 -18.71 6.85 CuCl2

Mg(OH)2(active) -6.08 12.72 18.79 Mg(OH)2

MgCr2O4 -1.83 16.03 17.87 MgCr2O4

MgCrO4 -22.75 -16.55 6.21 MgCrO4

Minium -33.97 43.47 77.44 Pb3O4

Mirabilite -9.42 -11.27 -1.85 Na2SO4:10H2O

Monteponite -7.48 8.58 16.06 CdO

Na2Cr2O7 -39.13 -49.23 -10.10 Na2Cr2O7

Na2CrO4 -23.08 -19.96 3.11 Na2CrO4

Nantokite -8.69 -15.81 -7.13 CuCl

Natron -10.63 -12.55 -1.92 Na2CO3:10H2O

Nesquehonite -4.69 -9.14 -4.45 MgCO3:3H2O

O2(g) -38.51 49.88 88.39 O2

Otavite -1.28 -13.27 -11.99 CdCO3

Pb(OH)2 -2.52 6.18 8.69 Pb(OH)2

Pb10(OH)6O(CO3)6 -60.60 -69.36 -8.76 Pb10(OH)6O(CO3)6

Pb2(OH)3Cl -9.18 -0.39 8.79 Pb2(OH)3Cl

Pb2O(OH)2 -13.84 12.35 26.19 Pb2O(OH)2

Pb2O3 -23.75 37.29 61.04 Pb2O3

Pb2OCO3 -9.32 -9.50 -0.18 Pb2OCO3

Pb3(AsO4)2 -18.30 -12.50 5.80 Pb3(AsO4)2

Pb3O2CO3 -15.37 -3.32 12.05 Pb3O2CO3

Pb3O2SO4 -13.47 -2.05 11.42 Pb3O2SO4

Pb4(OH)6SO4 -16.97 4.13 21.10 Pb4(OH)6SO4

Pb4O3SO4 -19.01 4.13 23.14 Pb4O3SO4

PbCrO4 -10.08 -23.09 -13.01 PbCrO4

Pbmetal -23.00 -18.76 4.24 Pb

PbO:0.3H2O -6.80 6.18 12.98 PbO:0.33H2O

Periclase -10.27 12.72 22.99 MgO

Phosgenite -15.18 -34.99 -19.81 PbCl2:PbCO3

Plattnerite -21.23 31.12 52.35 PbO2

Portlandite -10.47 13.52 24.00 Ca(OH)2

Smithsonite -2.88 -12.74 -9.85 ZnCO3

Tenorite -1.47 6.77 8.25 CuO

Thenardite -11.68 -11.27 0.41 Na2SO4

Thermonatrite -13.29 -12.55 0.73 Na2CO3:H2O

Zincite -3.05 9.12 12.17 ZnO

Zincosite -16.15 -11.46 4.70 ZnSO4

Zn(NO3)2:6H2O -18.13 -15.04 3.09 Zn(NO3)2:6H2O

Zn(OH)2 -3.08 9.12 12.20 Zn(OH)2

Zn(OH)2(am) -4.11 9.12 13.22 Zn(OH)2

Zn(OH)2(beta) -3.41 9.12 12.53 Zn(OH)2

Zn(OH)2(epsilon) -3.18 9.12 12.29 Zn(OH)2

Zn(OH)2(gamma) -2.62 9.12 11.73 Zn(OH)2

Zn2(OH)2SO4 -9.84 -2.34 7.50 Zn2(OH)2SO4

Zn2(OH)3Cl -9.70 5.49 15.19 Zn2(OH)3Cl

Zn3(AsO4)2:2.5H2O -17.33 -3.68 13.65 Zn3(AsO4)2:2.5H2O

Zn3O(SO4)2 -35.11 -13.80 21.31 Zn3O(SO4)2

Zn4(OH)6SO4 -12.51 15.89 28.40 Zn4(OH)6SO4

Zn5(OH)8Cl2 -18.40 20.10 38.50 Zn5(OH)8Cl2

ZnCl2 -24.09 -16.37 7.72 ZnCl2

ZnCO3:1H2O -2.48 -12.74 -10.26 ZnCO3:1H2O

Znmetal -43.04 -15.82 27.21 Zn

ZnO(active) -2.90 9.12 12.01 ZnO

ZnSO4:1H2O -11.23 -11.46 -0.23 ZnSO4:1H2O

**For a gas, SI = log10(fugacity). Fugacity = pressure * phi / 1 atm.

For ideal gases, phi = 1.

Initial solution 14.

----------------------------Distribution of species----------------------------

Log Log Log mole V

Species Molality Activity Molality Activity Gamma cm?mol

OH- 1.204e-06 1.135e-06 -5.919 -5.945 -0.026 (0)

H+ 2.854e-09 2.692e-09 -8.545 -8.570 -0.025 0.00

H2O 5.551e+01 9.999e-01 1.744 -0.000 0.000 18.02

As(3) 3.407e-21

H3AsO3 3.062e-21 3.062e-21 -20.514 -20.514 0.000 (0)

H2AsO3- 3.458e-22 3.248e-22 -21.461 -21.488 -0.027 (0)

HAsO3-2 7.137e-26 5.554e-26 -25.146 -25.255 -0.109 (0)

H4AsO3+ 4.347e-30 4.083e-30 -29.362 -29.389 -0.027 (0)

AsO3-3 8.143e-31 4.631e-31 -30.089 -30.334 -0.245 (0)

As(5) 4.561e-08

HAsO4-2 4.459e-08 3.470e-08 -7.351 -7.460 -0.109 (0)

H2AsO4- 9.669e-10 9.082e-10 -9.015 -9.042 -0.027 (0)

AsO4-3 4.985e-11 2.835e-11 -10.302 -10.547 -0.245 (0)

H3AsO4 3.647e-16 3.650e-16 -15.438 -15.438 0.000 (0)

C(4) 1.745e-03

HCO3- 1.685e-03 1.592e-03 -2.773 -2.798 -0.025 (0)

CO3-2 2.564e-05 2.029e-05 -4.591 -4.693 -0.102 (0)

H2CO3 1.172e-05 1.172e-05 -4.931 -4.931 0.000 (0)

CaCO3 1.015e-05 1.015e-05 -4.994 -4.994 0.000 (0)

CaHCO3+ 9.086e-06 8.586e-06 -5.042 -5.066 -0.025 (0)

MgHCO3+ 1.329e-06 1.254e-06 -5.876 -5.902 -0.025 (0)

MgCO3 1.095e-06 1.095e-06 -5.961 -5.961 0.000 (0)

NaHCO3 2.086e-07 2.086e-07 -6.681 -6.681 0.000 (0)

NaCO3- 1.074e-07 1.014e-07 -6.969 -6.994 -0.025 (0)

ZnCO3 3.315e-08 3.315e-08 -7.479 -7.479 0.000 (0)

CuCO3 1.907e-08 1.907e-08 -7.720 -7.720 0.000 (0)

PbCO3 5.387e-09 5.387e-09 -8.269 -8.269 0.000 (0)

CdCO3 3.392e-09 3.392e-09 -8.470 -8.470 0.000 (0)

Cu(CO3)2-2 1.339e-09 1.042e-09 -8.873 -8.982 -0.109 (0)

ZnHCO3+ 1.114e-09 1.046e-09 -8.953 -8.980 -0.027 (0)

Pb(CO3)2-2 4.051e-10 3.153e-10 -9.392 -9.501 -0.109 (0)

PbHCO3+ 8.139e-11 7.644e-11 -10.089 -10.117 -0.027 (0)

Cd(CO3)2-2 6.557e-11 5.102e-11 -10.183 -10.292 -0.109 (0)

CdHCO3+ 2.071e-11 1.945e-11 -10.684 -10.711 -0.027 (0)

CuHCO3+ 1.249e-11 1.173e-11 -10.903 -10.931 -0.027 (0)

Ca 5.968e-04

Ca+2 5.612e-04 4.442e-04 -3.251 -3.352 -0.102 (0)

CaSO4 1.597e-05 1.597e-05 -4.797 -4.797 0.000 (0)

CaCO3 1.015e-05 1.015e-05 -4.994 -4.994 0.000 (0)

CaHCO3+ 9.086e-06 8.586e-06 -5.042 -5.066 -0.025 (0)

CaNO3+ 4.760e-07 4.470e-07 -6.322 -6.350 -0.027 (0)

CaOH+ 8.916e-09 8.425e-09 -8.050 -8.074 -0.025 (0)

Cd 1.316e-08

Cd+2 9.264e-09 7.333e-09 -8.033 -8.135 -0.102 (0)

CdCO3 3.392e-09 3.392e-09 -8.470 -8.470 0.000 (0)

CdSO4 2.607e-10 2.607e-10 -9.584 -9.584 0.000 (0)

CdOH+ 7.192e-11 6.755e-11 -10.143 -10.170 -0.027 (0)

Cd(CO3)2-2 6.557e-11 5.102e-11 -10.183 -10.292 -0.109 (0)

CdCl+ 5.894e-11 5.536e-11 -10.230 -10.257 -0.027 (0)

CdHCO3+ 2.071e-11 1.945e-11 -10.684 -10.711 -0.027 (0)

CdNO3+ 1.097e-11 1.030e-11 -10.960 -10.987 -0.027 (0)

CdOHCl 5.880e-12 5.880e-12 -11.231 -11.231 0.000 (0)

Cd(OH)2 5.143e-12 5.143e-12 -11.289 -11.289 0.000 (0)

Cd(SO4)2-2 9.938e-13 7.733e-13 -12.003 -12.112 -0.109 (0)

CdCl2 1.786e-14 1.786e-14 -13.748 -13.748 0.000 (0)

Cd(OH)3- 1.252e-15 1.176e-15 -14.903 -14.930 -0.027 (0)

Cd(NO3)2 9.346e-16 9.346e-16 -15.029 -15.029 0.000 (0)

Cd2OH+3 5.292e-18 3.009e-18 -17.276 -17.522 -0.245 (0)

CdCl3- 8.342e-19 7.834e-19 -18.079 -18.106 -0.027 (0)

Cd(OH)4-2 9.250e-22 7.198e-22 -21.034 -21.143 -0.109 (0)

Cl 8.561e-05

Cl- 8.561e-05 8.076e-05 -4.067 -4.093 -0.025 (0)

CdCl+ 5.894e-11 5.536e-11 -10.230 -10.257 -0.027 (0)

ZnOHCl 2.821e-11 2.821e-11 -10.550 -10.550 0.000 (0)

CdOHCl 5.880e-12 5.880e-12 -11.231 -11.231 0.000 (0)

ZnCl+ 5.441e-12 5.132e-12 -11.264 -11.290 -0.025 (0)

CuCl 6.858e-13 6.858e-13 -12.164 -12.164 0.000 (0)

PbCl+ 2.237e-13 2.101e-13 -12.650 -12.678 -0.027 (0)

CuCl+ 1.814e-14 1.711e-14 -13.741 -13.767 -0.025 (0)

CdCl2 1.786e-14 1.786e-14 -13.748 -13.748 0.000 (0)

CuCl2- 1.274e-14 1.201e-14 -13.895 -13.920 -0.025 (0)

ZnCl2 3.343e-16 3.343e-16 -15.476 -15.476 0.000 (0)

PbCl2 7.063e-17 7.063e-17 -16.151 -16.151 0.000 (0)

CrCl+2 6.408e-18 4.986e-18 -17.193 -17.302 -0.109 (0)

CdCl3- 8.342e-19 7.834e-19 -18.079 -18.106 -0.027 (0)

CuCl3-2 2.457e-19 1.952e-19 -18.610 -18.710 -0.100 (0)

CuCl2 2.225e-19 2.225e-19 -18.653 -18.653 0.000 (0)

ZnCl3- 2.133e-20 2.011e-20 -19.671 -19.696 -0.025 (0)

CrOHCl2 3.127e-21 3.127e-21 -20.505 -20.505 0.000 (0)

PbCl3- 2.868e-21 2.694e-21 -20.542 -20.570 -0.027 (0)

CrCl2+ 1.027e-23 9.649e-24 -22.988 -23.016 -0.027 (0)

ZnCl4-2 9.023e-25 7.167e-25 -24.045 -24.145 -0.100 (0)

CuCl3- 1.344e-25 1.268e-25 -24.872 -24.897 -0.025 (0)

PbCl4-2 1.015e-25 7.900e-26 -24.993 -25.102 -0.109 (0)

CrO3Cl- 3.014e-26 2.831e-26 -25.521 -25.548 -0.027 (0)

CuCl4-2 1.096e-31 8.703e-32 -30.960 -31.060 -0.100 (0)

Cr(2) 1.397e-25

Cr+2 1.397e-25 1.087e-25 -24.855 -24.964 -0.109 (0)

Cr(3) 5.770e-07

Cr(OH)3 2.728e-07 2.728e-07 -6.564 -6.564 0.000 (0)

Cr(OH)2+ 2.067e-07 1.941e-07 -6.685 -6.712 -0.027 (0)

CrO2- 5.126e-08 4.814e-08 -7.290 -7.317 -0.027 (0)

Cr(OH)4- 4.326e-08 4.063e-08 -7.364 -7.391 -0.027 (0)

Cr(OH)+2 2.897e-09 2.254e-09 -8.538 -8.647 -0.109 (0)

CrOHSO4 1.848e-11 1.848e-11 -10.733 -10.733 0.000 (0)

Cr+3 1.462e-13 8.316e-14 -12.835 -13.080 -0.245 (0)

CrSO4+ 1.947e-14 1.828e-14 -13.711 -13.738 -0.027 (0)

CrCl+2 6.408e-18 4.986e-18 -17.193 -17.302 -0.109 (0)

Cr2(OH)2SO4+2 9.157e-19 7.125e-19 -18.038 -18.147 -0.109 (0)

CrNO3+2 3.361e-19 2.615e-19 -18.474 -18.582 -0.109 (0)

Cr2(OH)2(SO4)2 7.729e-21 7.729e-21 -20.112 -20.112 0.000 (0)

CrOHCl2 3.127e-21 3.127e-21 -20.505 -20.505 0.000 (0)

CrCl2+ 1.027e-23 9.649e-24 -22.988 -23.016 -0.027 (0)

Cr(6) 3.027e-12

CrO4-2 3.004e-12 2.378e-12 -11.522 -11.624 -0.102 (0)

HCrO4- 2.113e-14 1.984e-14 -13.675 -13.702 -0.027 (0)

NaCrO4- 2.186e-15 2.053e-15 -14.660 -14.688 -0.027 (0)

KCrO4- 2.105e-16 1.977e-16 -15.677 -15.704 -0.027 (0)

H2CrO4 1.963e-23 1.963e-23 -22.707 -22.707 0.000 (0)

CrO3SO4-2 3.984e-24 3.100e-24 -23.400 -23.509 -0.109 (0)

CrO3Cl- 3.014e-26 2.831e-26 -25.521 -25.548 -0.027 (0)

Cr2O7-2 2.633e-26 2.049e-26 -25.579 -25.688 -0.109 (0)

Cu(1) 7.881e-12

Cu+ 7.182e-12 6.746e-12 -11.144 -11.171 -0.027 (0)

CuCl 6.858e-13 6.858e-13 -12.164 -12.164 0.000 (0)

CuCl2- 1.274e-14 1.201e-14 -13.895 -13.920 -0.025 (0)

CuCl3-2 2.457e-19 1.952e-19 -18.610 -18.710 -0.100 (0)

Cu(2) 2.298e-08

CuCO3 1.907e-08 1.907e-08 -7.720 -7.720 0.000 (0)

Cu(OH)2 1.409e-09 1.409e-09 -8.851 -8.851 0.000 (0)

Cu(CO3)2-2 1.339e-09 1.042e-09 -8.873 -8.982 -0.109 (0)

CuOH+ 9.314e-10 8.784e-10 -9.031 -9.056 -0.025 (0)

Cu+2 2.016e-10 1.596e-10 -9.695 -9.797 -0.102 (0)

CuHCO3+ 1.249e-11 1.173e-11 -10.903 -10.931 -0.027 (0)

Cu(OH)3- 1.151e-11 1.081e-11 -10.939 -10.966 -0.027 (0)

CuSO4 5.544e-12 5.544e-12 -11.256 -11.256 0.000 (0)

CuNO3+ 1.663e-13 1.562e-13 -12.779 -12.806 -0.027 (0)

Cu2(OH)2+2 2.238e-14 1.742e-14 -13.650 -13.759 -0.109 (0)

CuCl+ 1.814e-14 1.711e-14 -13.741 -13.767 -0.025 (0)

Cu(OH)4-2 4.092e-16 3.184e-16 -15.388 -15.497 -0.109 (0)

Cu(NO3)2 5.110e-18 5.110e-18 -17.292 -17.292 0.000 (0)

CuCl2 2.225e-19 2.225e-19 -18.653 -18.653 0.000 (0)

CuCl3- 1.344e-25 1.268e-25 -24.872 -24.897 -0.025 (0)

CuCl4-2 1.096e-31 8.703e-32 -30.960 -31.060 -0.100 (0)

H(0) 1.200e-28

H2 5.998e-29 6.002e-29 -28.222 -28.222 0.000 (0)

K 2.376e-05

K+ 2.373e-05 2.238e-05 -4.625 -4.650 -0.025 (0)

KSO4- 2.807e-08 2.651e-08 -7.552 -7.577 -0.025 (0)

KCrO4- 2.105e-16 1.977e-16 -15.677 -15.704 -0.027 (0)

Mg 1.108e-04

Mg+2 1.059e-04 8.385e-05 -3.975 -4.077 -0.102 (0)

MgSO4 2.461e-06 2.461e-06 -5.609 -5.609 0.000 (0)

MgHCO3+ 1.329e-06 1.254e-06 -5.876 -5.902 -0.025 (0)

MgCO3 1.095e-06 1.095e-06 -5.961 -5.961 0.000 (0)

MgOH+ 3.101e-08 2.932e-08 -7.508 -7.533 -0.024 (0)

N(5) 3.011e-04

NO3- 3.006e-04 2.836e-04 -3.522 -3.547 -0.025 (0)

CaNO3+ 4.760e-07 4.470e-07 -6.322 -6.350 -0.027 (0)

ZnNO3+ 2.376e-11 2.231e-11 -10.624 -10.651 -0.027 (0)

CdNO3+ 1.097e-11 1.030e-11 -10.960 -10.987 -0.027 (0)

PbNO3+ 3.779e-13 3.549e-13 -12.423 -12.450 -0.027 (0)

CuNO3+ 1.663e-13 1.562e-13 -12.779 -12.806 -0.027 (0)

Zn(NO3)2 1.144e-15 1.144e-15 -14.941 -14.941 0.000 (0)

Cd(NO3)2 9.346e-16 9.346e-16 -15.029 -15.029 0.000 (0)

Pb(NO3)2 2.054e-16 2.054e-16 -15.687 -15.687 0.000 (0)

Cu(NO3)2 5.110e-18 5.110e-18 -17.292 -17.292 0.000 (0)

CrNO3+2 3.361e-19 2.615e-19 -18.474 -18.582 -0.109 (0)

Na 1.847e-04

Na+ 1.842e-04 1.738e-04 -3.735 -3.760 -0.025 (0)

NaHCO3 2.086e-07 2.086e-07 -6.681 -6.681 0.000 (0)

NaSO4- 1.766e-07 1.668e-07 -6.753 -6.778 -0.025 (0)

NaCO3- 1.074e-07 1.014e-07 -6.969 -6.994 -0.025 (0)

NaCrO4- 2.186e-15 2.053e-15 -14.660 -14.688 -0.027 (0)

O(0) 0.000e+00

O2 0.000e+00 0.000e+00 -40.179 -40.179 0.000 (0)

Pb 6.976e-09

PbCO3 5.387e-09 5.387e-09 -8.269 -8.269 0.000 (0)

PbOH+ 8.835e-10 8.298e-10 -9.054 -9.081 -0.027 (0)

Pb(CO3)2-2 4.051e-10 3.153e-10 -9.392 -9.501 -0.109 (0)

Pb+2 1.116e-10 8.831e-11 -9.952 -10.054 -0.102 (0)

Pb(OH)2 9.817e-11 9.817e-11 -10.008 -10.008 0.000 (0)

PbHCO3+ 8.139e-11 7.644e-11 -10.089 -10.117 -0.027 (0)

PbSO4 7.898e-12 7.898e-12 -11.102 -11.102 0.000 (0)

Pb(OH)3- 3.910e-13 3.672e-13 -12.408 -12.435 -0.027 (0)

PbNO3+ 3.779e-13 3.549e-13 -12.423 -12.450 -0.027 (0)

PbCl+ 2.237e-13 2.101e-13 -12.650 -12.678 -0.027 (0)

Pb(SO4)2-2 1.117e-14 8.691e-15 -13.952 -14.061 -0.109 (0)

Pb(OH)4-2 4.324e-16 3.364e-16 -15.364 -15.473 -0.109 (0)

Pb(NO3)2 2.054e-16 2.054e-16 -15.687 -15.687 0.000 (0)

PbCl2 7.063e-17 7.063e-17 -16.151 -16.151 0.000 (0)

Pb2OH+3 2.042e-18 1.161e-18 -17.690 -17.935 -0.245 (0)

PbCl3- 2.868e-21 2.694e-21 -20.542 -20.570 -0.027 (0)

Pb3(OH)4+2 1.859e-21 1.447e-21 -20.731 -20.840 -0.109 (0)

PbCl4-2 1.015e-25 7.900e-26 -24.993 -25.102 -0.109 (0)

Pb4(OH)4+4 4.930e-27 1.807e-27 -26.307 -26.743 -0.436 (0)

S(6) 2.493e-04

SO4-2 2.307e-04 1.826e-04 -3.637 -3.738 -0.102 (0)

CaSO4 1.597e-05 1.597e-05 -4.797 -4.797 0.000 (0)

MgSO4 2.461e-06 2.461e-06 -5.609 -5.609 0.000 (0)

NaSO4- 1.766e-07 1.668e-07 -6.753 -6.778 -0.025 (0)

KSO4- 2.807e-08 2.651e-08 -7.552 -7.577 -0.025 (0)

ZnSO4 9.936e-10 9.936e-10 -9.003 -9.003 0.000 (0)

CdSO4 2.607e-10 2.607e-10 -9.584 -9.584 0.000 (0)

HSO4- 3.181e-11 3.002e-11 -10.497 -10.523 -0.025 (0)

CrOHSO4 1.848e-11 1.848e-11 -10.733 -10.733 0.000 (0)

PbSO4 7.898e-12 7.898e-12 -11.102 -11.102 0.000 (0)

CuSO4 5.544e-12 5.544e-12 -11.256 -11.256 0.000 (0)

Zn(SO4)2-2 2.319e-12 1.804e-12 -11.635 -11.744 -0.109 (0)

Cd(SO4)2-2 9.938e-13 7.733e-13 -12.003 -12.112 -0.109 (0)

CrSO4+ 1.947e-14 1.828e-14 -13.711 -13.738 -0.027 (0)

Pb(SO4)2-2 1.117e-14 8.691e-15 -13.952 -14.061 -0.109 (0)

Cr2(OH)2SO4+2 9.157e-19 7.125e-19 -18.038 -18.147 -0.109 (0)

Cr2(OH)2(SO4)2 7.729e-21 7.729e-21 -20.112 -20.112 0.000 (0)

CrO3SO4-2 3.984e-24 3.100e-24 -23.400 -23.509 -0.109 (0)

Zn 8.105e-08

Zn+2 3.587e-08 2.839e-08 -7.445 -7.547 -0.102 (0)

ZnCO3 3.315e-08 3.315e-08 -7.479 -7.479 0.000 (0)

Zn(OH)2 6.297e-09 6.297e-09 -8.201 -8.201 0.000 (0)

ZnOH+ 3.431e-09 3.223e-09 -8.465 -8.492 -0.027 (0)

ZnHCO3+ 1.114e-09 1.046e-09 -8.953 -8.980 -0.027 (0)

ZnSO4 9.936e-10 9.936e-10 -9.003 -9.003 0.000 (0)

Zn(OH)3- 1.257e-10 1.181e-10 -9.901 -9.928 -0.027 (0)

ZnOHCl 2.821e-11 2.821e-11 -10.550 -10.550 0.000 (0)

ZnNO3+ 2.376e-11 2.231e-11 -10.624 -10.651 -0.027 (0)

ZnCl+ 5.441e-12 5.132e-12 -11.264 -11.290 -0.025 (0)

Zn(SO4)2-2 2.319e-12 1.804e-12 -11.635 -11.744 -0.109 (0)

Zn(OH)4-2 2.260e-14 1.758e-14 -13.646 -13.755 -0.109 (0)

Zn(NO3)2 1.144e-15 1.144e-15 -14.941 -14.941 0.000 (0)

ZnCl2 3.343e-16 3.343e-16 -15.476 -15.476 0.000 (0)

ZnCl3- 2.133e-20 2.011e-20 -19.671 -19.696 -0.025 (0)

ZnCl4-2 9.023e-25 7.167e-25 -24.045 -24.145 -0.100 (0)

------------------------------Saturation indices-------------------------------

Phase SI** log IAP log K(283 K, 1 atm)

Anglesite -5.89 -13.79 -7.90 PbSO4

Anhydrite -2.80 -7.09 -4.29 CaSO4

Antlerite -7.64 1.15 8.79 Cu3(OH)4SO4

Aragonite 0.14 -8.05 -8.19 CaCO3

Arsenolite -78.74 -82.06 -3.32 As4O6

Artinite -6.42 4.29 10.72 MgCO3:Mg(OH)2:3H2O

As2O5 -37.79 -30.88 6.92 As2O5

Atacamite -6.23 2.02 8.26 Cu2(OH)3Cl

Azurite -5.61 -21.64 -16.02 Cu3(OH)2(CO3)2

Bianchite -9.53 -11.29 -1.76 ZnSO4:6H2O

Brochantite -8.61 8.49 17.10 Cu4(OH)6SO4

Brucite -4.84 13.06 17.90 Mg(OH)2

Ca3(AsO4)2:4H2O -11.81 10.49 22.30 Ca3(AsO4)2:4H2O

CaCrO4 -12.96 -14.98 -2.02 CaCrO4

Calcite 0.36 -8.05 -8.41 CaCO3

Cd(OH)2 -5.52 9.01 14.52 Cd(OH)2

Cd(OH)2(am) -5.53 9.01 14.54 Cd(OH)2

Cd3(OH)2(SO4)2 -21.45 -14.74 6.71 Cd3(OH)2(SO4)2

Cd3(OH)4SO4 -16.42 6.14 22.56 Cd3(OH)4SO4

Cd4(OH)6SO4 -13.26 15.14 28.40 Cd4(OH)6SO4

CdCl2 -15.83 -16.32 -0.49 CdCl2

CdCl2:1H2O -14.70 -16.32 -1.62 CdCl2:1H2O

CdCl2:2.5H2O -14.34 -16.32 -1.98 CdCl2:2.5H2O

Cdmetal(alpha) -30.35 -16.13 14.21 Cd

Cdmetal(gamma) -30.46 -16.13 14.32 Cd

CdOHCl -7.48 -3.66 3.82 CdOHCl

CdSO4 -12.18 -11.87 0.31 CdSO4

CdSO4:1H2O -10.44 -11.87 -1.43 CdSO4:1H2O

CdSO4:2.67H2O -10.17 -11.87 -1.71 CdSO4:2.67H2O

Cerussite -1.39 -14.75 -13.36 PbCO3

CH4(g) -78.96 -122.39 -43.43 CH4

Chalcanthite -10.84 -13.54 -2.70 CuSO4:5H2O

Claudetite -78.48 -82.06 -3.58 As4O6

CO2(g) -3.65 -21.83 -18.18 CO2

Cotunnite -13.22 -18.24 -5.02 PbCl2

Cr(OH)2 -18.97 -7.82 11.15 Cr(OH)2

Cr(OH)3 0.25 1.86 1.61 Cr(OH)3

Cr(OH)3(am) 2.61 1.86 -0.75 Cr(OH)3

Cr2O3 5.60 3.72 -1.89 Cr2O3

CrCl2 -48.27 -33.15 15.12 CrCl2

CrCl3 -52.37 -36.13 16.24 CrCl3

Crmetal -65.04 -32.96 32.08 Cr

CrO3 -25.60 -28.76 -3.16 CrO3

Cu(OH)2 -1.85 7.34 9.20 Cu(OH)2

Cu2(OH)3NO3 -7.36 2.57 9.92 Cu2(OH)3NO3

Cu2SO4 -24.31 -26.08 -1.77 Cu2SO4

Cu3(AsO4)2:2H2O -14.95 -8.85 6.10 Cu3(AsO4)2:2H2O

CuCO3 -2.99 -14.49 -11.50 CuCO3

CuCrO4 -15.98 -21.42 -5.44 CuCrO4

Cumetal -5.75 -15.17 -9.42 Cu

CuOCuSO4 -17.77 -6.19 11.58 CuOCuSO4

Cuprite -4.95 -5.20 -0.26 Cu2O

CuSO4 -17.15 -13.54 3.62 CuSO4

Dolomite(disordered) -0.70 -16.81 -16.11 CaMg(CO3)2

Dolomite(ordered) -0.09 -16.81 -16.72 CaMg(CO3)2

Epsomite -5.58 -7.82 -2.23 MgSO4:7H2O

Goslarite -9.14 -11.29 -2.14 ZnSO4:7H2O

Gypsum -2.47 -7.09 -4.62 CaSO4:2H2O

Halite -9.42 -7.85 1.57 NaCl

Huntite -5.38 -34.35 -28.97 CaMg3(CO3)4

Hydrocerussite -3.64 -22.41 -18.77 Pb3(OH)2(CO3)2

Hydromagnesite -15.27 -22.01 -6.74 Mg5(CO3)4(OH)2:4H2O

K2Cr2O7 -31.70 -49.69 -17.99 K2Cr2O7

K2CrO4 -20.24 -20.92 -0.68 K2CrO4

Langite -10.53 8.49 19.03 Cu4(OH)6SO4:H2O

Larnakite -6.47 -6.71 -0.23 PbO:PbSO4

Laurionite -6.20 -5.58 0.62 PbOHCl

Lime -20.71 13.79 34.50 CaO

Litharge -6.22 7.09 13.30 PbO

Magnesite -1.12 -8.77 -7.65 MgCO3

Malachite -1.13 -7.15 -6.01 Cu2(OH)2CO3

Massicot -6.43 7.09 13.51 PbO

Melanothallite -24.83 -17.98 6.85 CuCl2

Mg(OH)2(active) -5.73 13.06 18.79 Mg(OH)2

MgCr2O4 -1.09 16.78 17.87 MgCr2O4

MgCrO4 -21.91 -15.70 6.21 MgCrO4

Minium -31.04 46.40 77.44 Pb3O4

Mirabilite -9.41 -11.26 -1.85 Na2SO4:10H2O

Monteponite -7.06 9.01 16.06 CdO

Na2Cr2O7 -37.81 -47.91 -10.10 Na2Cr2O7

Na2CrO4 -22.26 -19.14 3.11 Na2CrO4

Nantokite -8.14 -15.26 -7.13 CuCl

Natron -10.29 -12.21 -1.92 Na2CO3:10H2O

Nesquehonite -4.32 -8.77 -4.45 MgCO3:3H2O

O2(g) -38.11 50.28 88.39 O2

Otavite -0.83 -12.83 -11.99 CdCO3

Pb(OH)2 -1.61 7.09 8.69 Pb(OH)2

Pb10(OH)6O(CO3)6 -51.38 -60.14 -8.76 Pb10(OH)6O(CO3)6

Pb2(OH)3Cl -7.28 1.51 8.79 Pb2(OH)3Cl

Pb2O(OH)2 -12.02 14.17 26.19 Pb2O(OH)2

Pb2O3 -21.73 39.31 61.04 Pb2O3

Pb2OCO3 -7.48 -7.66 -0.18 Pb2OCO3

Pb3(AsO4)2 -15.42 -9.62 5.80 Pb3(AsO4)2

Pb3O2CO3 -12.62 -0.57 12.05 Pb3O2CO3

Pb3O2SO4 -11.04 0.38 11.42 Pb3O2SO4

Pb4(OH)6SO4 -13.63 7.47 21.10 Pb4(OH)6SO4

Pb4O3SO4 -15.68 7.47 23.14 Pb4O3SO4

PbCrO4 -8.67 -21.68 -13.01 PbCrO4

Pbmetal -22.29 -18.05 4.24 Pb

PbO:0.3H2O -5.89 7.09 12.98 PbO:0.33H2O

Periclase -9.92 13.06 22.99 MgO

Phosgenite -13.18 -32.99 -19.81 PbCl2:PbCO3

Plattnerite -20.12 32.23 52.35 PbO2

Portlandite -10.21 13.79 24.00 Ca(OH)2

Smithsonite -2.39 -12.24 -9.85 ZnCO3

Tenorite -0.90 7.34 8.25 CuO

Thenardite -11.66 -11.26 0.41 Na2SO4

Thermonatrite -12.95 -12.21 0.73 Na2CO3:H2O

Zincite -2.57 9.59 12.17 ZnO

Zincosite -15.98 -11.29 4.70 ZnSO4

Zn(NO3)2:6H2O -17.73 -14.64 3.09 Zn(NO3)2:6H2O

Zn(OH)2 -2.61 9.59 12.20 Zn(OH)2

Zn(OH)2(am) -3.63 9.59 13.22 Zn(OH)2

Zn(OH)2(beta) -2.93 9.59 12.53 Zn(OH)2

Zn(OH)2(epsilon) -2.70 9.59 12.29 Zn(OH)2

Zn(OH)2(gamma) -2.14 9.59 11.73 Zn(OH)2

Zn2(OH)2SO4 -9.19 -1.69 7.50 Zn2(OH)2SO4

Zn2(OH)3Cl -8.67 6.52 15.19 Zn2(OH)3Cl

Zn3(AsO4)2:2.5H2O -15.75 -2.10 13.65 Zn3(AsO4)2:2.5H2O

Zn3O(SO4)2 -34.29 -12.98 21.31 Zn3O(SO4)2

Zn4(OH)6SO4 -10.91 17.49 28.40 Zn4(OH)6SO4

Zn5(OH)8Cl2 -15.86 22.64 38.50 Zn5(OH)8Cl2

ZnCl2 -23.46 -15.73 7.72 ZnCl2

ZnCO3:1H2O -1.98 -12.24 -10.26 ZnCO3:1H2O

Znmetal -42.76 -15.55 27.21 Zn

ZnO(active) -2.42 9.59 12.01 ZnO

ZnSO4:1H2O -11.06 -11.29 -0.23 ZnSO4:1H2O

**For a gas, SI = log10(fugacity). Fugacity = pressure * phi / 1 atm.

For ideal gases, phi = 1.

Initial solution 15.

----------------------------Distribution of species----------------------------

Log Log Log mole V

Species Molality Activity Molality Activity Gamma cm?mol

OH- 5.628e-07 5.314e-07 -6.250 -6.275 -0.025 (0)

H+ 6.085e-09 5.749e-09 -8.216 -8.240 -0.025 0.00

H2O 5.551e+01 9.999e-01 1.744 -0.000 0.000 18.02

As(3) 2.292e-20

H3AsO3 2.177e-20 2.177e-20 -19.662 -19.662 0.000 (0)

H2AsO3- 1.149e-21 1.081e-21 -20.940 -20.966 -0.026 (0)

HAsO3-2 1.104e-25 8.657e-26 -24.957 -25.063 -0.106 (0)

H4AsO3+ 6.590e-29 6.201e-29 -28.181 -28.208 -0.026 (0)

AsO3-3 5.843e-31 3.380e-31 -30.233 -30.471 -0.238 (0)

As(5) 1.584e-08

HAsO4-2 1.512e-08 1.186e-08 -7.820 -7.926 -0.106 (0)

H2AsO4- 7.044e-10 6.628e-10 -9.152 -9.179 -0.026 (0)

AsO4-3 7.842e-12 4.536e-12 -11.106 -11.343 -0.238 (0)

H3AsO4 5.686e-16 5.689e-16 -15.245 -15.245 0.000 (0)

C(4) 1.890e-03

HCO3- 1.833e-03 1.734e-03 -2.737 -2.761 -0.024 (0)

H2CO3 2.726e-05 2.726e-05 -4.564 -4.564 0.000 (0)

CO3-2 1.299e-05 1.035e-05 -4.886 -4.985 -0.099 (0)

CaHCO3+ 9.756e-06 9.233e-06 -5.011 -5.035 -0.024 (0)

CaCO3 5.110e-06 5.110e-06 -5.292 -5.292 0.000 (0)

MgHCO3+ 1.289e-06 1.218e-06 -5.890 -5.914 -0.025 (0)

MgCO3 4.982e-07 4.982e-07 -6.303 -6.303 0.000 (0)

NaHCO3 6.679e-08 6.679e-08 -7.175 -7.175 0.000 (0)

NaCO3- 1.608e-08 1.521e-08 -7.794 -7.818 -0.024 (0)

ZnCO3 1.215e-08 1.215e-08 -7.915 -7.915 0.000 (0)

CuCO3 9.635e-09 9.635e-09 -8.016 -8.016 0.000 (0)

ZnHCO3+ 8.701e-10 8.187e-10 -9.060 -9.087 -0.026 (0)

PbCO3 7.602e-10 7.602e-10 -9.119 -9.119 0.000 (0)

Cu(CO3)2-2 3.423e-10 2.684e-10 -9.466 -9.571 -0.106 (0)

CdCO3 4.748e-11 4.748e-11 -10.323 -10.323 0.000 (0)

Pb(CO3)2-2 2.894e-11 2.269e-11 -10.539 -10.644 -0.106 (0)

PbHCO3+ 2.449e-11 2.304e-11 -10.611 -10.638 -0.026 (0)

CuHCO3+ 1.345e-11 1.266e-11 -10.871 -10.898 -0.026 (0)

CdHCO3+ 6.180e-13 5.816e-13 -12.209 -12.235 -0.026 (0)

Cd(CO3)2-2 4.646e-13 3.642e-13 -12.333 -12.439 -0.106 (0)

Ca 5.806e-04

Ca+2 5.504e-04 4.386e-04 -3.259 -3.358 -0.099 (0)

CaSO4 1.495e-05 1.495e-05 -4.825 -4.825 0.000 (0)

CaHCO3+ 9.756e-06 9.233e-06 -5.011 -5.035 -0.024 (0)

CaCO3 5.110e-06 5.110e-06 -5.292 -5.292 0.000 (0)

CaNO3+ 3.158e-07 2.971e-07 -6.501 -6.527 -0.026 (0)

CaOH+ 4.115e-09 3.895e-09 -8.386 -8.410 -0.024 (0)

Cd 3.095e-10

Cd+2 2.527e-10 2.013e-10 -9.597 -9.696 -0.099 (0)

CdCO3 4.748e-11 4.748e-11 -10.323 -10.323 0.000 (0)

CdSO4 6.787e-12 6.787e-12 -11.168 -11.168 0.000 (0)

CdOH+ 9.226e-13 8.682e-13 -12.035 -12.061 -0.026 (0)

CdHCO3+ 6.180e-13 5.816e-13 -12.209 -12.235 -0.026 (0)

Cd(CO3)2-2 4.646e-13 3.642e-13 -12.333 -12.439 -0.106 (0)

CdCl+ 2.850e-13 2.682e-13 -12.545 -12.572 -0.026 (0)

CdNO3+ 2.023e-13 1.903e-13 -12.694 -12.720 -0.026 (0)

Cd(OH)2 3.095e-14 3.095e-14 -13.509 -13.509 0.000 (0)

Cd(SO4)2-2 2.436e-14 1.910e-14 -13.613 -13.719 -0.106 (0)

CdOHCl 1.334e-14 1.334e-14 -13.875 -13.875 0.000 (0)

CdCl2 1.527e-17 1.527e-17 -16.816 -16.816 0.000 (0)

Cd(NO3)2 1.163e-17 1.163e-17 -16.935 -16.935 0.000 (0)

Cd(OH)3- 3.520e-18 3.312e-18 -17.453 -17.480 -0.026 (0)

Cd2OH+3 1.835e-21 1.062e-21 -20.736 -20.974 -0.238 (0)

CdCl3- 1.256e-22 1.182e-22 -21.901 -21.927 -0.026 (0)

Cd(OH)4-2 1.211e-24 9.495e-25 -23.917 -24.022 -0.106 (0)

Cl 1.508e-05

Cl- 1.508e-05 1.425e-05 -4.821 -4.846 -0.025 (0)

ZnOHCl 1.675e-12 1.675e-12 -11.776 -11.776 0.000 (0)

ZnCl+ 6.889e-13 6.508e-13 -12.162 -12.187 -0.025 (0)

CdCl+ 2.850e-13 2.682e-13 -12.545 -12.572 -0.026 (0)

CuCl 1.199e-13 1.199e-13 -12.921 -12.921 0.000 (0)

CdOHCl 1.334e-14 1.334e-14 -13.875 -13.875 0.000 (0)

PbCl+ 1.090e-14 1.026e-14 -13.962 -13.989 -0.026 (0)

CuCl+ 3.166e-15 2.991e-15 -14.499 -14.524 -0.025 (0)

CuCl2- 3.924e-16 3.707e-16 -15.406 -15.431 -0.025 (0)

CdCl2 1.527e-17 1.527e-17 -16.816 -16.816 0.000 (0)

CrCl+2 1.091e-17 8.557e-18 -16.962 -17.068 -0.106 (0)

ZnCl2 7.483e-18 7.483e-18 -17.126 -17.126 0.000 (0)

PbCl2 6.087e-19 6.087e-19 -18.216 -18.216 0.000 (0)

CuCl2 6.865e-21 6.865e-21 -20.163 -20.163 0.000 (0)

CuCl3-2 1.330e-21 1.063e-21 -20.876 -20.974 -0.097 (0)

CrOHCl2 4.434e-22 4.434e-22 -21.353 -21.353 0.000 (0)

CdCl3- 1.256e-22 1.182e-22 -21.901 -21.927 -0.026 (0)

ZnCl3- 8.410e-23 7.945e-23 -22.075 -22.100 -0.025 (0)

PbCl3- 4.355e-24 4.098e-24 -23.361 -23.387 -0.026 (0)

CrCl2+ 3.105e-24 2.922e-24 -23.508 -23.534 -0.026 (0)

CuCl3- 7.306e-28 6.902e-28 -27.136 -27.161 -0.025 (0)

ZnCl4-2 6.250e-28 4.996e-28 -27.204 -27.301 -0.097 (0)

CrO3Cl- 5.439e-28 5.118e-28 -27.265 -27.291 -0.026 (0)

PbCl4-2 2.705e-29 2.121e-29 -28.568 -28.674 -0.106 (0)

CuCl4-2 1.046e-34 8.363e-35 -33.980 -34.078 -0.097 (0)

Cr(2) 1.348e-24

Cr+2 1.348e-24 1.057e-24 -23.870 -23.976 -0.106 (0)

Cr(3) 7.693e-07

Cr(OH)2+ 4.398e-07 4.138e-07 -6.357 -6.383 -0.026 (0)

Cr(OH)3 2.723e-07 2.723e-07 -6.565 -6.565 0.000 (0)

CrO2- 2.390e-08 2.249e-08 -7.622 -7.648 -0.026 (0)

Cr(OH)4- 2.018e-08 1.899e-08 -7.695 -7.722 -0.026 (0)

Cr(OH)+2 1.309e-08 1.026e-08 -7.883 -7.989 -0.106 (0)

CrOHSO4 7.981e-11 7.981e-11 -10.098 -10.098 0.000 (0)

Cr+3 1.398e-12 8.086e-13 -11.854 -12.092 -0.238 (0)

CrSO4+ 1.792e-13 1.686e-13 -12.747 -12.773 -0.026 (0)

Cr2(OH)2SO4+2 1.787e-17 1.401e-17 -16.748 -16.854 -0.106 (0)

CrCl+2 1.091e-17 8.557e-18 -16.962 -17.068 -0.106 (0)

CrNO3+2 2.183e-18 1.712e-18 -17.661 -17.767 -0.106 (0)

Cr2(OH)2(SO4)2 1.441e-19 1.441e-19 -18.841 -18.841 0.000 (0)

CrOHCl2 4.434e-22 4.434e-22 -21.353 -21.353 0.000 (0)

CrCl2+ 3.105e-24 2.922e-24 -23.508 -23.534 -0.026 (0)

Cr(6) 6.804e-14

CrO4-2 6.701e-14 5.339e-14 -13.174 -13.273 -0.099 (0)

HCrO4- 1.011e-15 9.516e-16 -14.995 -15.022 -0.026 (0)

NaCrO4- 1.441e-17 1.356e-17 -16.841 -16.868 -0.026 (0)

KCrO4- 3.278e-18 3.085e-18 -17.484 -17.511 -0.026 (0)

H2CrO4 2.011e-24 2.011e-24 -23.697 -23.697 0.000 (0)

CrO3SO4-2 3.842e-25 3.012e-25 -24.415 -24.521 -0.106 (0)

CrO3Cl- 5.439e-28 5.118e-28 -27.265 -27.291 -0.026 (0)

Cr2O7-2 6.011e-29 4.713e-29 -28.221 -28.327 -0.106 (0)

Cu(1) 7.222e-12

Cu+ 7.101e-12 6.682e-12 -11.149 -11.175 -0.026 (0)

CuCl 1.199e-13 1.199e-13 -12.921 -12.921 0.000 (0)

CuCl2- 3.924e-16 3.707e-16 -15.406 -15.431 -0.025 (0)

CuCl3-2 1.330e-21 1.063e-21 -20.876 -20.974 -0.097 (0)

Cu(2) 1.093e-08

CuCO3 9.635e-09 9.635e-09 -8.016 -8.016 0.000 (0)

CuOH+ 4.313e-10 4.074e-10 -9.365 -9.390 -0.025 (0)

Cu(CO3)2-2 3.423e-10 2.684e-10 -9.466 -9.571 -0.106 (0)

Cu(OH)2 3.060e-10 3.060e-10 -9.514 -9.514 0.000 (0)

Cu+2 1.984e-10 1.581e-10 -9.702 -9.801 -0.099 (0)

CuHCO3+ 1.345e-11 1.266e-11 -10.871 -10.898 -0.026 (0)

CuSO4 5.209e-12 5.209e-12 -11.283 -11.283 0.000 (0)

Cu(OH)3- 1.168e-12 1.100e-12 -11.932 -11.959 -0.026 (0)

CuNO3+ 1.107e-13 1.042e-13 -12.956 -12.982 -0.026 (0)

Cu2(OH)2+2 4.779e-15 3.747e-15 -14.321 -14.426 -0.106 (0)

CuCl+ 3.166e-15 2.991e-15 -14.499 -14.524 -0.025 (0)

Cu(OH)4-2 1.933e-17 1.516e-17 -16.714 -16.819 -0.106 (0)

Cu(NO3)2 2.294e-18 2.294e-18 -17.639 -17.639 0.000 (0)

CuCl2 6.865e-21 6.865e-21 -20.163 -20.163 0.000 (0)

CuCl3- 7.306e-28 6.902e-28 -27.136 -27.161 -0.025 (0)

CuCl4-2 1.046e-34 8.363e-35 -33.980 -34.078 -0.097 (0)

H(0) 5.472e-28

H2 2.736e-28 2.738e-28 -27.563 -27.563 0.000 (0)

K 1.648e-05

K+ 1.646e-05 1.555e-05 -4.784 -4.808 -0.025 (0)

KSO4- 1.847e-08 1.747e-08 -7.734 -7.758 -0.024 (0)

KCrO4- 3.278e-18 3.085e-18 -17.484 -17.511 -0.026 (0)

Mg 9.776e-05

Mg+2 9.387e-05 7.480e-05 -4.027 -4.126 -0.099 (0)

MgSO4 2.083e-06 2.083e-06 -5.681 -5.681 0.000 (0)

MgHCO3+ 1.289e-06 1.218e-06 -5.890 -5.914 -0.025 (0)

MgCO3 4.982e-07 4.982e-07 -6.303 -6.303 0.000 (0)

MgOH+ 1.293e-08 1.225e-08 -7.888 -7.912 -0.024 (0)

N(5) 2.024e-04

NO3- 2.021e-04 1.909e-04 -3.695 -3.719 -0.025 (0)

CaNO3+ 3.158e-07 2.971e-07 -6.501 -6.527 -0.026 (0)

ZnNO3+ 1.147e-11 1.079e-11 -10.940 -10.967 -0.026 (0)

CdNO3+ 2.023e-13 1.903e-13 -12.694 -12.720 -0.026 (0)

CuNO3+ 1.107e-13 1.042e-13 -12.956 -12.982 -0.026 (0)

PbNO3+ 7.026e-14 6.611e-14 -13.153 -13.180 -0.026 (0)

Zn(NO3)2 3.726e-16 3.726e-16 -15.429 -15.429 0.000 (0)

Pb(NO3)2 2.576e-17 2.576e-17 -16.589 -16.589 0.000 (0)

Cd(NO3)2 1.163e-17 1.163e-17 -16.935 -16.935 0.000 (0)

Cu(NO3)2 2.294e-18 2.294e-18 -17.639 -17.639 0.000 (0)

CrNO3+2 2.183e-18 1.712e-18 -17.661 -17.767 -0.106 (0)

Na 5.421e-05

Na+ 5.408e-05 5.109e-05 -4.267 -4.292 -0.025 (0)

NaHCO3 6.679e-08 6.679e-08 -7.175 -7.175 0.000 (0)

NaSO4- 4.918e-08 4.652e-08 -7.308 -7.332 -0.024 (0)

NaCO3- 1.608e-08 1.521e-08 -7.794 -7.818 -0.024 (0)

NaCrO4- 1.441e-17 1.356e-17 -16.841 -16.868 -0.026 (0)

O(0) 0.000e+00

O2 0.000e+00 0.000e+00 -41.498 -41.497 0.000 (0)

Pb 9.667e-10

PbCO3 7.602e-10 7.602e-10 -9.119 -9.119 0.000 (0)

PbOH+ 1.143e-10 1.075e-10 -9.942 -9.969 -0.026 (0)

Pb+2 3.067e-11 2.444e-11 -10.513 -10.612 -0.099 (0)

Pb(CO3)2-2 2.894e-11 2.269e-11 -10.539 -10.644 -0.106 (0)

PbHCO3+ 2.449e-11 2.304e-11 -10.611 -10.638 -0.026 (0)

Pb(OH)2 5.955e-12 5.955e-12 -11.225 -11.225 0.000 (0)

PbSO4 2.073e-12 2.073e-12 -11.683 -11.683 0.000 (0)

PbNO3+ 7.026e-14 6.611e-14 -13.153 -13.180 -0.026 (0)

Pb(OH)3- 1.108e-14 1.043e-14 -13.955 -13.982 -0.026 (0)

PbCl+ 1.090e-14 1.026e-14 -13.962 -13.989 -0.026 (0)

Pb(SO4)2-2 2.760e-15 2.164e-15 -14.559 -14.665 -0.106 (0)

Pb(NO3)2 2.576e-17 2.576e-17 -16.589 -16.589 0.000 (0)

Pb(OH)4-2 5.707e-18 4.474e-18 -17.244 -17.349 -0.106 (0)

PbCl2 6.087e-19 6.087e-19 -18.216 -18.216 0.000 (0)

Pb2OH+3 7.199e-20 4.164e-20 -19.143 -19.380 -0.238 (0)

PbCl3- 4.355e-24 4.098e-24 -23.361 -23.387 -0.026 (0)

Pb3(OH)4+2 1.879e-24 1.473e-24 -23.726 -23.832 -0.106 (0)

PbCl4-2 2.705e-29 2.121e-29 -28.568 -28.674 -0.106 (0)

Pb4(OH)4+4 1.348e-30 5.093e-31 -29.870 -30.293 -0.423 (0)

S(6) 2.345e-04

SO4-2 2.174e-04 1.732e-04 -3.663 -3.761 -0.099 (0)

CaSO4 1.495e-05 1.495e-05 -4.825 -4.825 0.000 (0)

MgSO4 2.083e-06 2.083e-06 -5.681 -5.681 0.000 (0)

NaSO4- 4.918e-08 4.652e-08 -7.308 -7.332 -0.024 (0)

KSO4- 1.847e-08 1.747e-08 -7.734 -7.758 -0.024 (0)

ZnSO4 6.772e-10 6.772e-10 -9.169 -9.169 0.000 (0)

CrOHSO4 7.981e-11 7.981e-11 -10.098 -10.098 0.000 (0)

HSO4- 6.434e-11 6.081e-11 -10.192 -10.216 -0.025 (0)

CdSO4 6.787e-12 6.787e-12 -11.168 -11.168 0.000 (0)

CuSO4 5.209e-12 5.209e-12 -11.283 -11.283 0.000 (0)

PbSO4 2.073e-12 2.073e-12 -11.683 -11.683 0.000 (0)

Zn(SO4)2-2 1.488e-12 1.166e-12 -11.827 -11.933 -0.106 (0)

CrSO4+ 1.792e-13 1.686e-13 -12.747 -12.773 -0.026 (0)

Cd(SO4)2-2 2.436e-14 1.910e-14 -13.613 -13.719 -0.106 (0)

Pb(SO4)2-2 2.760e-15 2.164e-15 -14.559 -14.665 -0.106 (0)

Cr2(OH)2SO4+2 1.787e-17 1.401e-17 -16.748 -16.854 -0.106 (0)

Cr2(OH)2(SO4)2 1.441e-19 1.441e-19 -18.841 -18.841 0.000 (0)

CrO3SO4-2 3.842e-25 3.012e-25 -24.415 -24.521 -0.106 (0)

Zn 4.147e-08

Zn+2 2.561e-08 2.040e-08 -7.592 -7.690 -0.099 (0)

ZnCO3 1.215e-08 1.215e-08 -7.915 -7.915 0.000 (0)

ZnOH+ 1.152e-09 1.084e-09 -8.938 -8.965 -0.026 (0)

Zn(OH)2 9.920e-10 9.920e-10 -9.003 -9.003 0.000 (0)

ZnHCO3+ 8.701e-10 8.187e-10 -9.060 -9.087 -0.026 (0)

ZnSO4 6.772e-10 6.772e-10 -9.169 -9.169 0.000 (0)

ZnNO3+ 1.147e-11 1.079e-11 -10.940 -10.967 -0.026 (0)

Zn(OH)3- 9.254e-12 8.708e-12 -11.034 -11.060 -0.026 (0)

ZnOHCl 1.675e-12 1.675e-12 -11.776 -11.776 0.000 (0)

Zn(SO4)2-2 1.488e-12 1.166e-12 -11.827 -11.933 -0.106 (0)

ZnCl+ 6.889e-13 6.508e-13 -12.162 -12.187 -0.025 (0)

Zn(OH)4-2 7.745e-16 6.072e-16 -15.111 -15.217 -0.106 (0)

Zn(NO3)2 3.726e-16 3.726e-16 -15.429 -15.429 0.000 (0)

ZnCl2 7.483e-18 7.483e-18 -17.126 -17.126 0.000 (0)

ZnCl3- 8.410e-23 7.945e-23 -22.075 -22.100 -0.025 (0)

ZnCl4-2 6.250e-28 4.996e-28 -27.204 -27.301 -0.097 (0)

------------------------------Saturation indices-------------------------------

Phase SI** log IAP log K(283 K, 1 atm)

Anglesite -6.47 -14.37 -7.90 PbSO4

Anhydrite -2.83 -7.12 -4.29 CaSO4

Antlerite -8.99 -0.20 8.79 Cu3(OH)4SO4

Aragonite -0.15 -8.34 -8.19 CaCO3

Arsenolite -75.33 -78.65 -3.32 As4O6

Artinite -7.47 3.24 10.72 MgCO3:Mg(OH)2:3H2O

As2O5 -37.41 -30.49 6.92 As2O5

Atacamite -7.99 0.27 8.26 Cu2(OH)3Cl

Azurite -6.87 -22.89 -16.02 Cu3(OH)2(CO3)2

Bianchite -9.69 -11.45 -1.76 ZnSO4:6H2O

Brochantite -10.63 6.48 17.10 Cu4(OH)6SO4

Brucite -5.55 12.35 17.90 Mg(OH)2

Ca3(AsO4)2:4H2O -13.42 8.88 22.30 Ca3(AsO4)2:4H2O

CaCrO4 -14.61 -16.63 -2.02 CaCrO4

Calcite 0.06 -8.34 -8.41 CaCO3

Cd(OH)2 -7.74 6.78 14.52 Cd(OH)2

Cd(OH)2(am) -7.75 6.78 14.54 Cd(OH)2

Cd3(OH)2(SO4)2 -26.84 -20.13 6.71 Cd3(OH)2(SO4)2

Cd3(OH)4SO4 -22.45 0.11 22.56 Cd3(OH)4SO4

Cd4(OH)6SO4 -21.50 6.90 28.40 Cd4(OH)6SO4

CdCl2 -18.90 -19.39 -0.49 CdCl2

CdCl2:1H2O -17.76 -19.39 -1.62 CdCl2:1H2O

CdCl2:2.5H2O -17.41 -19.39 -1.98 CdCl2:2.5H2O

Cdmetal(alpha) -31.91 -17.70 14.21 Cd

Cdmetal(gamma) -32.02 -17.70 14.32 Cd

CdOHCl -10.13 -6.30 3.82 CdOHCl

CdSO4 -13.77 -13.46 0.31 CdSO4

CdSO4:1H2O -12.02 -13.46 -1.43 CdSO4:1H2O

CdSO4:2.67H2O -11.75 -13.46 -1.71 CdSO4:2.67H2O

Cerussite -2.24 -15.60 -13.36 PbCO3

CH4(g) -75.96 -119.39 -43.43 CH4

Chalcanthite -10.87 -13.56 -2.70 CuSO4:5H2O

Claudetite -75.07 -78.65 -3.58 As4O6

CO2(g) -3.28 -21.47 -18.18 CO2

Cotunnite -15.28 -20.30 -5.02 PbCl2

Cr(OH)2 -18.64 -7.50 11.15 Cr(OH)2

Cr(OH)3 0.25 1.86 1.61 Cr(OH)3

Cr(OH)3(am) 2.61 1.86 -0.75 Cr(OH)3

Cr2O3 5.60 3.71 -1.89 Cr2O3

CrCl2 -48.79 -33.67 15.12 CrCl2

CrCl3 -53.64 -37.40 16.24 CrCl3

Crmetal -64.06 -31.98 32.08 Cr

CrO3 -26.59 -29.75 -3.16 CrO3

Cu(OH)2 -2.52 6.68 9.20 Cu(OH)2

Cu2(OH)3NO3 -8.52 1.40 9.92 Cu2(OH)3NO3

Cu2SO4 -24.34 -26.11 -1.77 Cu2SO4

Cu3(AsO4)2:2H2O -16.55 -10.45 6.10 Cu3(AsO4)2:2H2O

CuCO3 -3.29 -14.79 -11.50 CuCO3

CuCrO4 -17.63 -23.07 -5.44 CuCrO4

Cumetal -5.75 -15.18 -9.42 Cu

CuOCuSO4 -18.46 -6.88 11.58 CuOCuSO4

Cuprite -5.61 -5.87 -0.26 Cu2O

CuSO4 -17.18 -13.56 3.62 CuSO4

Dolomite(disordered) -1.34 -17.45 -16.11 CaMg(CO3)2

Dolomite(ordered) -0.73 -17.45 -16.72 CaMg(CO3)2

Epsomite -5.65 -7.89 -2.23 MgSO4:7H2O

Goslarite -9.31 -11.45 -2.14 ZnSO4:7H2O

Gypsum -2.50 -7.12 -4.62 CaSO4:2H2O

Halite -10.71 -9.14 1.57 NaCl

Huntite -6.71 -35.68 -28.97 CaMg3(CO3)4

Hydrocerussite -6.55 -25.33 -18.77 Pb3(OH)2(CO3)2

Hydromagnesite -17.35 -24.09 -6.74 Mg5(CO3)4(OH)2:4H2O

K2Cr2O7 -34.65 -52.64 -17.99 K2Cr2O7

K2CrO4 -22.21 -22.89 -0.68 K2CrO4

Langite -12.55 6.48 19.03 Cu4(OH)6SO4:H2O

Larnakite -8.27 -8.50 -0.23 PbO:PbSO4

Laurionite -7.84 -7.22 0.62 PbOHCl

Lime -21.38 13.12 34.50 CaO

Litharge -7.43 5.87 13.30 PbO

Magnesite -1.47 -9.11 -7.65 MgCO3

Malachite -2.09 -8.11 -6.01 Cu2(OH)2CO3

Massicot -7.65 5.87 13.51 PbO

Melanothallite -26.34 -19.49 6.85 CuCl2

Mg(OH)2(active) -6.44 12.35 18.79 Mg(OH)2

MgCr2O4 -1.80 16.07 17.87 MgCr2O4

MgCrO4 -23.60 -17.40 6.21 MgCrO4

Minium -35.35 42.09 77.44 Pb3O4

Mirabilite -10.49 -12.34 -1.85 Na2SO4:10H2O

Monteponite -9.28 6.78 16.06 CdO

Na2Cr2O7 -41.51 -51.61 -10.10 Na2Cr2O7

Na2CrO4 -24.97 -21.86 3.11 Na2CrO4

Nantokite -8.90 -16.02 -7.13 CuCl

Natron -11.65 -13.57 -1.92 Na2CO3:10H2O

Nesquehonite -4.67 -9.11 -4.45 MgCO3:3H2O

O2(g) -39.43 48.96 88.39 O2

Otavite -2.69 -14.68 -11.99 CdCO3

Pb(OH)2 -2.82 5.87 8.69 Pb(OH)2

Pb10(OH)6O(CO3)6 -61.35 -70.11 -8.76 Pb10(OH)6O(CO3)6

Pb2(OH)3Cl -10.14 -1.35 8.79 Pb2(OH)3Cl

Pb2O(OH)2 -14.45 11.74 26.19 Pb2O(OH)2

Pb2O3 -24.82 36.22 61.04 Pb2O3

Pb2OCO3 -9.55 -9.73 -0.18 Pb2OCO3

Pb3(AsO4)2 -18.68 -12.88 5.80 Pb3(AsO4)2

Pb3O2CO3 -15.91 -3.86 12.05 Pb3O2CO3

Pb3O2SO4 -14.06 -2.64 11.42 Pb3O2SO4

Pb4(OH)6SO4 -17.87 3.23 21.10 Pb4(OH)6SO4

Pb4O3SO4 -19.91 3.23 23.14 Pb4O3SO4

PbCrO4 -10.87 -23.88 -13.01 PbCrO4

Pbmetal -22.85 -18.61 4.24 Pb

PbO:0.3H2O -7.11 5.87 12.98 PbO:0.33H2O

Periclase -10.63 12.35 22.99 MgO

Phosgenite -16.09 -35.90 -19.81 PbCl2:PbCO3

Plattnerite -22.00 30.35 52.35 PbO2

Portlandite -10.87 13.12 24.00 Ca(OH)2

Smithsonite -2.82 -12.68 -9.85 ZnCO3

Tenorite -1.57 6.68 8.25 CuO

Thenardite -12.75 -12.34 0.41 Na2SO4

Thermonatrite -14.30 -13.57 0.73 Na2CO3:H2O

Zincite -3.38 8.79 12.17 ZnO

Zincosite -16.15 -11.45 4.70 ZnSO4

Zn(NO3)2:6H2O -18.22 -15.13 3.09 Zn(NO3)2:6H2O

Zn(OH)2 -3.41 8.79 12.20 Zn(OH)2

Zn(OH)2(am) -4.43 8.79 13.22 Zn(OH)2

Zn(OH)2(beta) -3.74 8.79 12.53 Zn(OH)2

Zn(OH)2(epsilon) -3.50 8.79 12.29 Zn(OH)2

Zn(OH)2(gamma) -2.94 8.79 11.73 Zn(OH)2

Zn2(OH)2SO4 -10.16 -2.66 7.50 Zn2(OH)2SO4

Zn2(OH)3Cl -10.70 4.49 15.19 Zn2(OH)3Cl

Zn3(AsO4)2:2.5H2O -17.77 -4.12 13.65 Zn3(AsO4)2:2.5H2O

Zn3O(SO4)2 -35.42 -14.11 21.31 Zn3O(SO4)2

Zn4(OH)6SO4 -13.48 14.92 28.40 Zn4(OH)6SO4

Zn5(OH)8Cl2 -20.72 17.78 38.50 Zn5(OH)8Cl2

ZnCl2 -25.11 -17.38 7.72 ZnCl2

ZnCO3:1H2O -2.42 -12.68 -10.26 ZnCO3:1H2O

Znmetal -42.90 -15.69 27.21 Zn

ZnO(active) -3.22 8.79 12.01 ZnO

ZnSO4:1H2O -11.22 -11.45 -0.23 ZnSO4:1H2O

**For a gas, SI = log10(fugacity). Fugacity = pressure * phi / 1 atm.

For ideal gases, phi = 1.

Initial solution 16.

----------------------------Distribution of species----------------------------

Log Log Log mole V

Species Molality Activity Molality Activity Gamma cm?mol

OH- 1.486e-06 1.396e-06 -5.828 -5.855 -0.027 (0)

H+ 2.327e-09 2.188e-09 -8.633 -8.660 -0.027 0.00

H2O 5.551e+01 9.999e-01 1.744 -0.000 0.000 18.02

As(3) 8.043e-22

H3AsO3 7.058e-22 7.058e-22 -21.151 -21.151 0.000 (0)

H2AsO3- 9.843e-23 9.211e-23 -22.007 -22.036 -0.029 (0)

HAsO3-2 2.527e-26 1.938e-26 -25.597 -25.713 -0.115 (0)

H4AsO3+ 8.176e-31 7.651e-31 -30.087 -30.116 -0.029 (0)

AsO3-3 3.613e-31 1.988e-31 -30.442 -30.702 -0.259 (0)

As(5) 2.435e-08

HAsO4-2 2.390e-08 1.833e-08 -7.622 -7.737 -0.115 (0)

H2AsO4- 4.166e-10 3.899e-10 -9.380 -9.409 -0.029 (0)

AsO4-3 3.348e-11 1.842e-11 -10.475 -10.735 -0.259 (0)

H3AsO4 1.273e-16 1.274e-16 -15.895 -15.895 0.000 (0)

C(4) 2.006e-03

HCO3- 1.927e-03 1.814e-03 -2.715 -2.741 -0.026 (0)

CO3-2 3.640e-05 2.845e-05 -4.439 -4.546 -0.107 (0)

CaCO3 1.594e-05 1.594e-05 -4.797 -4.797 0.000 (0)

CaHCO3+ 1.164e-05 1.096e-05 -4.934 -4.960 -0.026 (0)

H2CO3 1.085e-05 1.085e-05 -4.964 -4.964 0.000 (0)

MgCO3 2.015e-06 2.015e-06 -5.696 -5.696 0.000 (0)

MgHCO3+ 1.995e-06 1.875e-06 -5.700 -5.727 -0.027 (0)

NaHCO3 1.610e-07 1.610e-07 -6.793 -6.793 0.000 (0)

NaCO3- 1.023e-07 9.634e-08 -6.990 -7.016 -0.026 (0)

ZnCO3 3.566e-08 3.566e-08 -7.448 -7.448 0.000 (0)

CuCO3 2.935e-08 2.935e-08 -7.532 -7.532 0.000 (0)

PbCO3 3.451e-09 3.451e-09 -8.462 -8.462 0.000 (0)

Cu(CO3)2-2 2.931e-09 2.247e-09 -8.533 -8.648 -0.115 (0)

CdCO3 1.620e-09 1.620e-09 -8.790 -8.790 0.000 (0)

ZnHCO3+ 9.771e-10 9.144e-10 -9.010 -9.039 -0.029 (0)

Pb(CO3)2-2 3.693e-10 2.832e-10 -9.433 -9.548 -0.115 (0)

Cd(CO3)2-2 4.457e-11 3.418e-11 -10.351 -10.466 -0.115 (0)

PbHCO3+ 4.254e-11 3.980e-11 -10.371 -10.400 -0.029 (0)

CuHCO3+ 1.568e-11 1.467e-11 -10.805 -10.833 -0.029 (0)

CdHCO3+ 8.071e-12 7.553e-12 -11.093 -11.122 -0.029 (0)

Ca 6.826e-04

Ca+2 6.367e-04 4.977e-04 -3.196 -3.303 -0.107 (0)

CaSO4 1.763e-05 1.763e-05 -4.754 -4.754 0.000 (0)

CaCO3 1.594e-05 1.594e-05 -4.797 -4.797 0.000 (0)

CaHCO3+ 1.164e-05 1.096e-05 -4.934 -4.960 -0.026 (0)

CaNO3+ 6.786e-07 6.350e-07 -6.168 -6.197 -0.029 (0)

CaOH+ 1.233e-08 1.161e-08 -7.909 -7.935 -0.026 (0)

Cd 5.009e-09

Cd+2 3.197e-09 2.499e-09 -8.495 -8.602 -0.107 (0)

CdCO3 1.620e-09 1.620e-09 -8.790 -8.790 0.000 (0)

CdSO4 8.756e-11 8.756e-11 -10.058 -10.058 0.000 (0)

Cd(CO3)2-2 4.457e-11 3.418e-11 -10.351 -10.466 -0.115 (0)

CdOH+ 3.026e-11 2.831e-11 -10.519 -10.548 -0.029 (0)

CdCl+ 1.196e-11 1.119e-11 -10.922 -10.951 -0.029 (0)

CdHCO3+ 8.071e-12 7.553e-12 -11.093 -11.122 -0.029 (0)

CdNO3+ 4.755e-12 4.450e-12 -11.323 -11.352 -0.029 (0)

Cd(OH)2 2.653e-12 2.653e-12 -11.576 -11.576 0.000 (0)

CdOHCl 1.462e-12 1.462e-12 -11.835 -11.835 0.000 (0)

Cd(SO4)2-2 3.340e-13 2.561e-13 -12.476 -12.592 -0.115 (0)

CdCl2 2.141e-15 2.141e-15 -14.669 -14.669 0.000 (0)

Cd(OH)3- 7.970e-16 7.458e-16 -15.099 -15.127 -0.029 (0)

Cd(NO3)2 5.119e-16 5.119e-16 -15.291 -15.291 0.000 (0)

Cd2OH+3 7.811e-19 4.298e-19 -18.107 -18.367 -0.259 (0)

CdCl3- 5.955e-20 5.573e-20 -19.225 -19.254 -0.029 (0)

Cd(OH)4-2 7.327e-22 5.618e-22 -21.135 -21.250 -0.115 (0)

Cl 5.095e-05

Cl- 5.095e-05 4.791e-05 -4.293 -4.320 -0.027 (0)

ZnOHCl 1.579e-11 1.579e-11 -10.802 -10.802 0.000 (0)

CdCl+ 1.196e-11 1.119e-11 -10.922 -10.951 -0.029 (0)

ZnCl+ 2.484e-12 2.335e-12 -11.605 -11.632 -0.027 (0)

CdOHCl 1.462e-12 1.462e-12 -11.835 -11.835 0.000 (0)

CuCl 4.465e-13 4.465e-13 -12.350 -12.350 0.000 (0)

PbCl+ 6.085e-14 5.694e-14 -13.216 -13.245 -0.029 (0)

CuCl+ 1.185e-14 1.114e-14 -13.926 -13.953 -0.027 (0)

CuCl2- 4.935e-15 4.640e-15 -14.307 -14.334 -0.027 (0)

CdCl2 2.141e-15 2.141e-15 -14.669 -14.669 0.000 (0)

ZnCl2 9.024e-17 9.024e-17 -16.045 -16.045 0.000 (0)

PbCl2 1.136e-17 1.136e-17 -16.945 -16.945 0.000 (0)

CrCl+2 2.134e-18 1.636e-18 -17.671 -17.786 -0.115 (0)

CuCl2 8.594e-20 8.594e-20 -19.066 -19.066 0.000 (0)

CdCl3- 5.955e-20 5.573e-20 -19.225 -19.254 -0.029 (0)

CuCl3-2 5.700e-20 4.472e-20 -19.244 -19.350 -0.105 (0)

ZnCl3- 3.426e-21 3.221e-21 -20.465 -20.492 -0.027 (0)

CrOHCl2 7.490e-22 7.490e-22 -21.126 -21.126 0.000 (0)

PbCl3- 2.746e-22 2.569e-22 -21.561 -21.590 -0.029 (0)

CrCl2+ 2.007e-24 1.878e-24 -23.697 -23.726 -0.029 (0)

ZnCl4-2 8.676e-26 6.807e-26 -25.062 -25.167 -0.105 (0)

CrO3Cl- 3.442e-26 3.221e-26 -25.463 -25.492 -0.029 (0)

CuCl3- 3.089e-26 2.904e-26 -25.510 -25.537 -0.027 (0)

PbCl4-2 5.829e-27 4.470e-27 -26.234 -26.350 -0.115 (0)

CuCl4-2 1.508e-32 1.183e-32 -31.822 -31.927 -0.105 (0)

Cr(2) 7.840e-26

Cr+2 7.840e-26 6.012e-26 -25.106 -25.221 -0.115 (0)

Cr(3) 5.770e-07

Cr(OH)3 2.810e-07 2.810e-07 -6.551 -6.551 0.000 (0)

Cr(OH)2+ 1.737e-07 1.625e-07 -6.760 -6.789 -0.029 (0)

CrO2- 6.519e-08 6.101e-08 -7.186 -7.215 -0.029 (0)

Cr(OH)4- 5.502e-08 5.149e-08 -7.259 -7.288 -0.029 (0)

Cr(OH)+2 2.001e-09 1.534e-09 -8.699 -8.814 -0.115 (0)

CrOHSO4 1.240e-11 1.240e-11 -10.907 -10.907 0.000 (0)

Cr+3 8.361e-14 4.600e-14 -13.078 -13.337 -0.259 (0)

CrSO4+ 1.065e-14 9.970e-15 -13.972 -14.001 -0.029 (0)

CrCl+2 2.134e-18 1.636e-18 -17.671 -17.786 -0.115 (0)

Cr2(OH)2SO4+2 4.243e-19 3.253e-19 -18.372 -18.488 -0.115 (0)

CrNO3+2 2.392e-19 1.834e-19 -18.621 -18.737 -0.115 (0)

Cr2(OH)2(SO4)2 3.479e-21 3.479e-21 -20.459 -20.459 0.000 (0)

CrOHCl2 7.490e-22 7.490e-22 -21.126 -21.126 0.000 (0)

CrCl2+ 2.007e-24 1.878e-24 -23.697 -23.726 -0.029 (0)

Cr(6) 8.886e-12

CrO4-2 8.831e-12 6.903e-12 -11.054 -11.161 -0.107 (0)

HCrO4- 5.004e-14 4.682e-14 -13.301 -13.330 -0.029 (0)

NaCrO4- 4.315e-15 4.038e-15 -14.365 -14.394 -0.029 (0)

KCrO4- 7.226e-16 6.762e-16 -15.141 -15.170 -0.029 (0)

H2CrO4 3.766e-23 3.766e-23 -22.424 -22.424 0.000 (0)

CrO3SO4-2 7.645e-24 5.862e-24 -23.117 -23.232 -0.115 (0)

Cr2O7-2 1.488e-25 1.141e-25 -24.827 -24.943 -0.115 (0)

CrO3Cl- 3.442e-26 3.221e-26 -25.463 -25.492 -0.029 (0)

Cu(1) 8.363e-12

Cu+ 7.911e-12 7.403e-12 -11.102 -11.131 -0.029 (0)

CuCl 4.465e-13 4.465e-13 -12.350 -12.350 0.000 (0)

CuCl2- 4.935e-15 4.640e-15 -14.307 -14.334 -0.027 (0)

CuCl3-2 5.700e-20 4.472e-20 -19.244 -19.350 -0.105 (0)

Cu(2) 3.615e-08

CuCO3 2.935e-08 2.935e-08 -7.532 -7.532 0.000 (0)

Cu(CO3)2-2 2.931e-09 2.247e-09 -8.533 -8.648 -0.115 (0)

Cu(OH)2 2.341e-09 2.341e-09 -8.631 -8.631 0.000 (0)

CuOH+ 1.261e-09 1.186e-09 -8.899 -8.926 -0.027 (0)

Cu+2 2.241e-10 1.752e-10 -9.650 -9.757 -0.107 (0)

Cu(OH)3- 2.362e-11 2.210e-11 -10.627 -10.656 -0.029 (0)

CuHCO3+ 1.568e-11 1.467e-11 -10.805 -10.833 -0.029 (0)

CuSO4 5.998e-12 5.998e-12 -11.222 -11.222 0.000 (0)

CuNO3+ 2.323e-13 2.174e-13 -12.634 -12.663 -0.029 (0)

Cu2(OH)2+2 4.141e-14 3.175e-14 -13.383 -13.498 -0.115 (0)

CuCl+ 1.185e-14 1.114e-14 -13.926 -13.953 -0.027 (0)

Cu(OH)4-2 1.044e-15 8.005e-16 -14.981 -15.097 -0.115 (0)

Cu(NO3)2 9.013e-18 9.013e-18 -17.045 -17.045 0.000 (0)

CuCl2 8.594e-20 8.594e-20 -19.066 -19.066 0.000 (0)

CuCl3- 3.089e-26 2.904e-26 -25.510 -25.537 -0.027 (0)

CuCl4-2 1.508e-32 1.183e-32 -31.822 -31.927 -0.105 (0)

H(0) 7.925e-29

H2 3.962e-29 3.966e-29 -28.402 -28.402 0.000 (0)

K 2.807e-05

K+ 2.804e-05 2.637e-05 -4.552 -4.579 -0.027 (0)

KSO4- 3.269e-08 3.078e-08 -7.486 -7.512 -0.026 (0)

KCrO4- 7.226e-16 6.762e-16 -15.141 -15.170 -0.029 (0)

Mg 1.480e-04

Mg+2 1.408e-04 1.101e-04 -3.851 -3.958 -0.107 (0)

MgSO4 3.185e-06 3.185e-06 -5.497 -5.497 0.000 (0)

MgCO3 2.015e-06 2.015e-06 -5.696 -5.696 0.000 (0)

MgHCO3+ 1.995e-06 1.875e-06 -5.700 -5.727 -0.027 (0)

MgOH+ 5.023e-08 4.734e-08 -7.299 -7.325 -0.026 (0)

N(5) 3.830e-04

NO3- 3.824e-04 3.595e-04 -3.418 -3.444 -0.027 (0)

CaNO3+ 6.786e-07 6.350e-07 -6.168 -6.197 -0.029 (0)

ZnNO3+ 2.319e-11 2.170e-11 -10.635 -10.664 -0.029 (0)

CdNO3+ 4.755e-12 4.450e-12 -11.323 -11.352 -0.029 (0)

CuNO3+ 2.323e-13 2.174e-13 -12.634 -12.663 -0.029 (0)

PbNO3+ 2.197e-13 2.056e-13 -12.658 -12.687 -0.029 (0)

Zn(NO3)2 1.411e-15 1.411e-15 -14.851 -14.851 0.000 (0)

Cd(NO3)2 5.119e-16 5.119e-16 -15.291 -15.291 0.000 (0)

Pb(NO3)2 1.508e-16 1.508e-16 -15.821 -15.821 0.000 (0)

Cu(NO3)2 9.013e-18 9.013e-18 -17.045 -17.045 0.000 (0)

CrNO3+2 2.392e-19 1.834e-19 -18.621 -18.737 -0.115 (0)

Na 1.256e-04

Na+ 1.252e-04 1.177e-04 -3.902 -3.929 -0.027 (0)

NaHCO3 1.610e-07 1.610e-07 -6.793 -6.793 0.000 (0)

NaSO4- 1.183e-07 1.114e-07 -6.927 -6.953 -0.026 (0)

NaCO3- 1.023e-07 9.634e-08 -6.990 -7.016 -0.026 (0)

NaCrO4- 4.315e-15 4.038e-15 -14.365 -14.394 -0.029 (0)

O(0) 3.031e-40

O2 1.516e-40 1.517e-40 -39.819 -39.819 0.000 (0)

Pb 4.485e-09

PbCO3 3.451e-09 3.451e-09 -8.462 -8.462 0.000 (0)

PbOH+ 4.984e-10 4.664e-10 -9.302 -9.331 -0.029 (0)

Pb(CO3)2-2 3.693e-10 2.832e-10 -9.433 -9.548 -0.115 (0)

Pb(OH)2 6.788e-11 6.788e-11 -10.168 -10.168 0.000 (0)

Pb+2 5.162e-11 4.035e-11 -10.287 -10.394 -0.107 (0)

PbHCO3+ 4.254e-11 3.980e-11 -10.371 -10.400 -0.029 (0)

PbSO4 3.557e-12 3.557e-12 -11.449 -11.449 0.000 (0)

Pb(OH)3- 3.339e-13 3.124e-13 -12.476 -12.505 -0.029 (0)

PbNO3+ 2.197e-13 2.056e-13 -12.658 -12.687 -0.029 (0)

PbCl+ 6.085e-14 5.694e-14 -13.216 -13.245 -0.029 (0)

Pb(SO4)2-2 5.032e-15 3.859e-15 -14.298 -14.414 -0.115 (0)

Pb(OH)4-2 4.592e-16 3.521e-16 -15.338 -15.453 -0.115 (0)

Pb(NO3)2 1.508e-16 1.508e-16 -15.821 -15.821 0.000 (0)

PbCl2 1.136e-17 1.136e-17 -16.945 -16.945 0.000 (0)

Pb2OH+3 5.421e-19 2.983e-19 -18.266 -18.525 -0.259 (0)

Pb3(OH)4+2 4.122e-22 3.161e-22 -21.385 -21.500 -0.115 (0)

PbCl3- 2.746e-22 2.569e-22 -21.561 -21.590 -0.029 (0)

PbCl4-2 5.829e-27 4.470e-27 -26.234 -26.350 -0.115 (0)

Pb4(OH)4+4 5.218e-28 1.804e-28 -27.283 -27.744 -0.461 (0)

S(6) 2.513e-04

SO4-2 2.303e-04 1.800e-04 -3.638 -3.745 -0.107 (0)

CaSO4 1.763e-05 1.763e-05 -4.754 -4.754 0.000 (0)

MgSO4 3.185e-06 3.185e-06 -5.497 -5.497 0.000 (0)

NaSO4- 1.183e-07 1.114e-07 -6.927 -6.953 -0.026 (0)

KSO4- 3.269e-08 3.078e-08 -7.486 -7.512 -0.026 (0)

ZnSO4 7.513e-10 7.513e-10 -9.124 -9.124 0.000 (0)

CdSO4 8.756e-11 8.756e-11 -10.058 -10.058 0.000 (0)

HSO4- 2.557e-11 2.405e-11 -10.592 -10.619 -0.027 (0)

CrOHSO4 1.240e-11 1.240e-11 -10.907 -10.907 0.000 (0)

CuSO4 5.998e-12 5.998e-12 -11.222 -11.222 0.000 (0)

PbSO4 3.557e-12 3.557e-12 -11.449 -11.449 0.000 (0)

Zn(SO4)2-2 1.754e-12 1.345e-12 -11.756 -11.871 -0.115 (0)

Cd(SO4)2-2 3.340e-13 2.561e-13 -12.476 -12.592 -0.115 (0)

CrSO4+ 1.065e-14 9.970e-15 -13.972 -14.001 -0.029 (0)

Pb(SO4)2-2 5.032e-15 3.859e-15 -14.298 -14.414 -0.115 (0)

Cr2(OH)2SO4+2 4.243e-19 3.253e-19 -18.372 -18.488 -0.115 (0)

Cr2(OH)2(SO4)2 3.479e-21 3.479e-21 -20.459 -20.459 0.000 (0)

CrO3SO4-2 7.645e-24 5.862e-24 -23.117 -23.232 -0.115 (0)

Zn 7.603e-08

ZnCO3 3.566e-08 3.566e-08 -7.448 -7.448 0.000 (0)

Zn+2 2.786e-08 2.178e-08 -7.555 -7.662 -0.107 (0)

Zn(OH)2 7.311e-09 7.311e-09 -8.136 -8.136 0.000 (0)

ZnOH+ 3.250e-09 3.041e-09 -8.488 -8.517 -0.029 (0)

ZnHCO3+ 9.771e-10 9.144e-10 -9.010 -9.039 -0.029 (0)

ZnSO4 7.513e-10 7.513e-10 -9.124 -9.124 0.000 (0)

Zn(OH)3- 1.802e-10 1.686e-10 -9.744 -9.773 -0.029 (0)

ZnNO3+ 2.319e-11 2.170e-11 -10.635 -10.664 -0.029 (0)

ZnOHCl 1.579e-11 1.579e-11 -10.802 -10.802 0.000 (0)

ZnCl+ 2.484e-12 2.335e-12 -11.605 -11.632 -0.027 (0)

Zn(SO4)2-2 1.754e-12 1.345e-12 -11.756 -11.871 -0.115 (0)

Zn(OH)4-2 4.029e-14 3.090e-14 -13.395 -13.510 -0.115 (0)

Zn(NO3)2 1.411e-15 1.411e-15 -14.851 -14.851 0.000 (0)

ZnCl2 9.024e-17 9.024e-17 -16.045 -16.045 0.000 (0)

ZnCl3- 3.426e-21 3.221e-21 -20.465 -20.492 -0.027 (0)

ZnCl4-2 8.676e-26 6.807e-26 -25.062 -25.167 -0.105 (0)

------------------------------Saturation indices-------------------------------

Phase SI** log IAP log K(283 K, 1 atm)

Anglesite -6.24 -14.14 -7.90 PbSO4

Anhydrite -2.75 -7.05 -4.29 CaSO4

Antlerite -7.16 1.63 8.79 Cu3(OH)4SO4

Aragonite 0.34 -7.85 -8.19 CaCO3

Arsenolite -81.29 -84.61 -3.32 As4O6

Artinite -5.86 4.86 10.72 MgCO3:Mg(OH)2:3H2O

As2O5 -38.71 -31.79 6.92 As2O5

Atacamite -6.11 2.15 8.26 Cu2(OH)3Cl

Azurite -5.02 -21.04 -16.02 Cu3(OH)2(CO3)2

Bianchite -9.65 -11.41 -1.76 ZnSO4:6H2O

Brochantite -7.92 9.19 17.10 Cu4(OH)6SO4

Brucite -4.54 13.36 17.90 Mg(OH)2

Ca3(AsO4)2:4H2O -12.04 10.26 22.30 Ca3(AsO4)2:4H2O

CaCrO4 -12.45 -14.46 -2.02 CaCrO4

Calcite 0.56 -7.85 -8.41 CaCO3

Cd(OH)2 -5.80 8.72 14.52 Cd(OH)2

Cd(OH)2(am) -5.82 8.72 14.54 Cd(OH)2

Cd3(OH)2(SO4)2 -22.69 -15.98 6.71 Cd3(OH)2(SO4)2

Cd3(OH)4SO4 -17.47 5.09 22.56 Cd3(OH)4SO4

Cd4(OH)6SO4 -14.59 13.81 28.40 Cd4(OH)6SO4

CdCl2 -16.76 -17.24 -0.49 CdCl2

CdCl2:1H2O -15.62 -17.24 -1.62 CdCl2:1H2O

CdCl2:2.5H2O -15.26 -17.24 -1.98 CdCl2:2.5H2O

Cdmetal(alpha) -30.82 -16.60 14.21 Cd

Cdmetal(gamma) -30.92 -16.60 14.32 Cd

CdOHCl -8.09 -4.26 3.82 CdOHCl

CdSO4 -12.66 -12.35 0.31 CdSO4

CdSO4:1H2O -10.91 -12.35 -1.43 CdSO4:1H2O

CdSO4:2.67H2O -10.64 -12.35 -1.71 CdSO4:2.67H2O

Cerussite -1.58 -14.94 -13.36 PbCO3

CH4(g) -79.71 -123.15 -43.43 CH4

Chalcanthite -10.81 -13.50 -2.70 CuSO4:5H2O

Claudetite -81.02 -84.61 -3.58 As4O6

CO2(g) -3.68 -21.87 -18.18 CO2

Cotunnite -14.01 -19.03 -5.02 PbCl2

Cr(OH)2 -19.05 -7.90 11.15 Cr(OH)2

Cr(OH)3 0.26 1.87 1.61 Cr(OH)3

Cr(OH)3(am) 2.62 1.87 -0.75 Cr(OH)3

Cr2O3 5.63 3.74 -1.89 Cr2O3

CrCl2 -48.98 -33.86 15.12 CrCl2

CrCl3 -53.31 -37.07 16.24 CrCl3

Crmetal -65.30 -33.22 32.08 Cr

CrO3 -25.32 -28.48 -3.16 CrO3

Cu(OH)2 -1.63 7.56 9.20 Cu(OH)2

Cu2(OH)3NO3 -6.90 3.02 9.92 Cu2(OH)3NO3

Cu2SO4 -24.23 -26.01 -1.77 Cu2SO4

Cu3(AsO4)2:2H2O -15.20 -9.10 6.10 Cu3(AsO4)2:2H2O

CuCO3 -2.80 -14.30 -11.50 CuCO3

CuCrO4 -15.48 -20.92 -5.44 CuCrO4

Cumetal -5.71 -15.13 -9.42 Cu

CuOCuSO4 -17.52 -5.94 11.58 CuOCuSO4

Cuprite -4.69 -4.94 -0.26 Cu2O

CuSO4 -17.12 -13.50 3.62 CuSO4

Dolomite(disordered) -0.24 -16.35 -16.11 CaMg(CO3)2

Dolomite(ordered) 0.37 -16.35 -16.72 CaMg(CO3)2

Epsomite -5.47 -7.70 -2.23 MgSO4:7H2O

Goslarite -9.26 -11.41 -2.14 ZnSO4:7H2O

Gypsum -2.43 -7.05 -4.62 CaSO4:2H2O

Halite -9.82 -8.25 1.57 NaCl

Huntite -4.39 -33.36 -28.97 CaMg3(CO3)4

Hydrocerussite -4.18 -22.95 -18.77 Pb3(OH)2(CO3)2

Hydromagnesite -13.92 -20.66 -6.74 Mg5(CO3)4(OH)2:4H2O

K2Cr2O7 -30.81 -48.80 -17.99 K2Cr2O7

K2CrO4 -19.64 -20.32 -0.68 K2CrO4

Langite -9.84 9.19 19.03 Cu4(OH)6SO4:H2O

Larnakite -6.98 -7.21 -0.23 PbO:PbSO4

Laurionite -6.68 -6.05 0.62 PbOHCl

Lime -20.48 14.02 34.50 CaO

Litharge -6.38 6.93 13.30 PbO

Magnesite -0.86 -8.50 -7.65 MgCO3

Malachite -0.72 -6.74 -6.01 Cu2(OH)2CO3

Massicot -6.59 6.93 13.51 PbO

Melanothallite -25.24 -18.40 6.85 CuCl2

Mg(OH)2(active) -5.43 13.36 18.79 Mg(OH)2

MgCr2O4 -0.76 17.10 17.87 MgCr2O4

MgCrO4 -21.33 -15.12 6.21 MgCrO4

Minium -31.34 46.10 77.44 Pb3O4

Mirabilite -9.75 -11.60 -1.85 Na2SO4:10H2O

Monteponite -7.35 8.72 16.06 CdO

Na2Cr2O7 -37.40 -47.50 -10.10 Na2Cr2O7

Na2CrO4 -22.13 -19.02 3.11 Na2CrO4

Nantokite -8.32 -15.45 -7.13 CuCl

Natron -10.48 -12.40 -1.92 Na2CO3:10H2O

Nesquehonite -4.06 -8.50 -4.45 MgCO3:3H2O

O2(g) -37.75 50.64 88.39 O2

Otavite -1.15 -13.15 -11.99 CdCO3

Pb(OH)2 -1.77 6.93 8.69 Pb(OH)2

Pb10(OH)6O(CO3)6 -53.18 -61.94 -8.76 Pb10(OH)6O(CO3)6

Pb2(OH)3Cl -7.92 0.87 8.79 Pb2(OH)3Cl

Pb2O(OH)2 -12.34 13.85 26.19 Pb2O(OH)2

Pb2O3 -21.87 39.17 61.04 Pb2O3

Pb2OCO3 -7.84 -8.01 -0.18 Pb2OCO3

Pb3(AsO4)2 -16.81 -11.01 5.80 Pb3(AsO4)2

Pb3O2CO3 -13.13 -1.09 12.05 Pb3O2CO3

Pb3O2SO4 -11.71 -0.29 11.42 Pb3O2SO4

Pb4(OH)6SO4 -14.46 6.64 21.10 Pb4(OH)6SO4

Pb4O3SO4 -16.51 6.64 23.14 Pb4O3SO4

PbCrO4 -8.55 -21.56 -13.01 PbCrO4

Pbmetal -22.63 -18.39 4.24 Pb

PbO:0.3H2O -6.05 6.93 12.98 PbO:0.33H2O

Periclase -9.63 13.36 22.99 MgO

Phosgenite -14.16 -33.97 -19.81 PbCl2:PbCO3

Plattnerite -20.10 32.25 52.35 PbO2

Portlandite -9.98 14.02 24.00 Ca(OH)2

Smithsonite -2.35 -12.21 -9.85 ZnCO3

Tenorite -0.68 7.56 8.25 CuO

Thenardite -12.01 -11.60 0.41 Na2SO4

Thermonatrite -13.14 -12.40 0.73 Na2CO3:H2O

Zincite -2.51 9.66 12.17 ZnO

Zincosite -16.10 -11.41 4.70 ZnSO4

Zn(NO3)2:6H2O -17.64 -14.55 3.09 Zn(NO3)2:6H2O

Zn(OH)2 -2.54 9.66 12.20 Zn(OH)2

Zn(OH)2(am) -3.56 9.66 13.22 Zn(OH)2

Zn(OH)2(beta) -2.87 9.66 12.53 Zn(OH)2

Zn(OH)2(epsilon) -2.64 9.66 12.29 Zn(OH)2

Zn(OH)2(gamma) -2.08 9.66 11.73 Zn(OH)2

Zn2(OH)2SO4 -9.25 -1.75 7.50 Zn2(OH)2SO4

Zn2(OH)3Cl -8.85 6.34 15.19 Zn2(OH)3Cl

Zn3(AsO4)2:2.5H2O -16.47 -2.82 13.65 Zn3(AsO4)2:2.5H2O

Zn3O(SO4)2 -34.46 -13.16 21.31 Zn3O(SO4)2

Zn4(OH)6SO4 -10.83 17.57 28.40 Zn4(OH)6SO4

Zn5(OH)8Cl2 -16.17 22.33 38.50 Zn5(OH)8Cl2

ZnCl2 -24.02 -16.30 7.72 ZnCl2

ZnCO3:1H2O -1.95 -12.21 -10.26 ZnCO3:1H2O

Znmetal -42.87 -15.66 27.21 Zn

ZnO(active) -2.35 9.66 12.01 ZnO

ZnSO4:1H2O -11.18 -11.41 -0.23 ZnSO4:1H2O

**For a gas, SI = log10(fugacity). Fugacity = pressure * phi / 1 atm.

For ideal gases, phi = 1.

Initial solution 17.

----------------------------Distribution of species----------------------------

Log Log Log mole V

Species Molality Activity Molality Activity Gamma cm?mol

OH- 5.697e-07 5.314e-07 -6.244 -6.275 -0.030 (0)

H+ 6.157e-09 5.749e-09 -8.211 -8.240 -0.030 0.00

H2O 5.551e+01 9.999e-01 1.744 -0.000 0.000 18.02

As(3) 5.920e-21

H3AsO3 5.619e-21 5.619e-21 -20.250 -20.250 0.000 (0)

H2AsO3- 3.007e-22 2.791e-22 -21.522 -21.554 -0.032 (0)

HAsO3-2 3.012e-26 2.235e-26 -25.521 -25.651 -0.130 (0)

H4AsO3+ 1.724e-29 1.600e-29 -28.763 -28.796 -0.032 (0)

AsO3-3 1.707e-31 8.723e-32 -30.768 -31.059 -0.292 (0)

As(5) 4.311e-09

HAsO4-2 4.125e-09 3.060e-09 -8.385 -8.514 -0.130 (0)

H2AsO4- 1.843e-10 1.711e-10 -9.734 -9.767 -0.032 (0)

AsO4-3 2.291e-12 1.171e-12 -11.640 -11.932 -0.292 (0)

H3AsO4 1.467e-16 1.468e-16 -15.834 -15.833 0.000 (0)

C(4) 2.925e-03

HCO3- 2.826e-03 2.643e-03 -2.549 -2.578 -0.029 (0)

H2CO3 4.156e-05 4.156e-05 -4.381 -4.381 0.000 (0)

CO3-2 2.076e-05 1.578e-05 -4.683 -4.802 -0.119 (0)

CaHCO3+ 1.371e-05 1.283e-05 -4.863 -4.892 -0.029 (0)

MgHCO3+ 1.081e-05 1.010e-05 -4.966 -4.996 -0.030 (0)

CaCO3 7.102e-06 7.102e-06 -5.149 -5.149 0.000 (0)

MgCO3 4.129e-06 4.129e-06 -5.384 -5.384 0.000 (0)

NaHCO3 1.824e-07 1.824e-07 -6.739 -6.739 0.000 (0)

ZnCO3 5.373e-08 5.373e-08 -7.270 -7.270 0.000 (0)

CuCO3 4.683e-08 4.683e-08 -7.330 -7.330 0.000 (0)

NaCO3- 4.441e-08 4.154e-08 -7.353 -7.382 -0.029 (0)

PbCO3 1.010e-08 1.010e-08 -7.996 -7.996 0.000 (0)

ZnHCO3+ 3.901e-09 3.621e-09 -8.409 -8.441 -0.032 (0)

Cu(CO3)2-2 2.680e-09 1.989e-09 -8.572 -8.701 -0.130 (0)

Pb(CO3)2-2 6.194e-10 4.596e-10 -9.208 -9.338 -0.130 (0)

PbHCO3+ 3.298e-10 3.061e-10 -9.482 -9.514 -0.032 (0)

CdCO3 2.626e-10 2.626e-10 -9.581 -9.581 0.000 (0)

CuHCO3+ 6.629e-11 6.152e-11 -10.179 -10.211 -0.032 (0)

Cd(CO3)2-2 4.139e-12 3.071e-12 -11.383 -11.513 -0.130 (0)

CdHCO3+ 3.465e-12 3.216e-12 -11.460 -11.493 -0.032 (0)

Ca 5.601e-04

Ca+2 5.260e-04 3.998e-04 -3.279 -3.398 -0.119 (0)

CaHCO3+ 1.371e-05 1.283e-05 -4.863 -4.892 -0.029 (0)

CaSO4 1.291e-05 1.291e-05 -4.889 -4.889 0.000 (0)

CaCO3 7.102e-06 7.102e-06 -5.149 -5.149 0.000 (0)

CaNO3+ 3.622e-07 3.361e-07 -6.441 -6.473 -0.032 (0)

CaOH+ 3.793e-09 3.550e-09 -8.421 -8.450 -0.029 (0)

Cd 1.262e-09

Cd+2 9.605e-10 7.301e-10 -9.017 -9.137 -0.119 (0)

CdCO3 2.626e-10 2.626e-10 -9.581 -9.581 0.000 (0)

CdSO4 2.331e-11 2.331e-11 -10.632 -10.632 0.000 (0)

Cd(CO3)2-2 4.139e-12 3.071e-12 -11.383 -11.513 -0.130 (0)

CdHCO3+ 3.465e-12 3.216e-12 -11.460 -11.493 -0.032 (0)

CdOH+ 3.392e-12 3.148e-12 -11.470 -11.502 -0.032 (0)

CdCl+ 3.313e-12 3.075e-12 -11.480 -11.512 -0.032 (0)

CdNO3+ 9.231e-13 8.567e-13 -12.035 -12.067 -0.032 (0)

CdOHCl 1.529e-13 1.529e-13 -12.816 -12.816 0.000 (0)

Cd(OH)2 1.122e-13 1.122e-13 -12.950 -12.950 0.000 (0)

Cd(SO4)2-2 8.371e-14 6.211e-14 -13.077 -13.207 -0.130 (0)

CdCl2 5.534e-16 5.534e-16 -15.257 -15.257 0.000 (0)

Cd(NO3)2 6.495e-17 6.495e-17 -16.187 -16.187 0.000 (0)

Cd(OH)3- 1.294e-17 1.201e-17 -16.888 -16.920 -0.032 (0)

Cd2OH+3 2.733e-20 1.396e-20 -19.563 -19.855 -0.292 (0)

CdCl3- 1.460e-20 1.355e-20 -19.836 -19.868 -0.032 (0)

Cd(OH)4-2 4.640e-24 3.443e-24 -23.333 -23.463 -0.130 (0)

Cl 4.826e-05

Cl- 4.826e-05 4.506e-05 -4.316 -4.346 -0.030 (0)

ZnOHCl 1.536e-11 1.536e-11 -10.814 -10.814 0.000 (0)

ZnCl+ 6.393e-12 5.968e-12 -11.194 -11.224 -0.030 (0)

CdCl+ 3.313e-12 3.075e-12 -11.480 -11.512 -0.032 (0)

CuCl 1.208e-12 1.208e-12 -11.918 -11.918 0.000 (0)

PbCl+ 3.046e-13 2.827e-13 -12.516 -12.549 -0.032 (0)

CdOHCl 1.529e-13 1.529e-13 -12.816 -12.816 0.000 (0)

CuCl+ 3.229e-14 3.014e-14 -13.491 -13.521 -0.030 (0)

CuCl2- 1.265e-14 1.181e-14 -13.898 -13.928 -0.030 (0)

CdCl2 5.534e-16 5.534e-16 -15.257 -15.257 0.000 (0)

ZnCl2 2.169e-16 2.169e-16 -15.664 -15.664 0.000 (0)

PbCl2 5.301e-17 5.301e-17 -16.276 -16.276 0.000 (0)

CrCl+2 2.708e-17 2.010e-17 -16.567 -16.697 -0.130 (0)

CuCl2 2.187e-19 2.187e-19 -18.660 -18.660 0.000 (0)

CuCl3-2 1.402e-19 1.070e-19 -18.853 -18.970 -0.117 (0)

CdCl3- 1.460e-20 1.355e-20 -19.836 -19.868 -0.032 (0)

ZnCl3- 7.800e-21 7.282e-21 -20.108 -20.138 -0.030 (0)

CrOHCl2 3.292e-21 3.292e-21 -20.483 -20.483 0.000 (0)

PbCl3- 1.216e-21 1.128e-21 -20.915 -20.948 -0.032 (0)

CrCl2+ 2.338e-23 2.170e-23 -22.631 -22.664 -0.032 (0)

ZnCl4-2 1.896e-25 1.448e-25 -24.722 -24.839 -0.117 (0)

CuCl3- 7.447e-26 6.952e-26 -25.128 -25.158 -0.030 (0)

PbCl4-2 2.488e-26 1.846e-26 -25.604 -25.734 -0.130 (0)

CrO3Cl- 1.295e-27 1.202e-27 -26.888 -26.920 -0.032 (0)

CuCl4-2 3.487e-32 2.663e-32 -31.457 -31.575 -0.117 (0)

Cr(2) 1.058e-24

Cr+2 1.058e-24 7.850e-25 -23.976 -24.105 -0.130 (0)

Cr(3) 5.770e-07

Cr(OH)2+ 3.312e-07 3.074e-07 -6.480 -6.512 -0.032 (0)

Cr(OH)3 2.022e-07 2.022e-07 -6.694 -6.694 0.000 (0)

CrO2- 1.800e-08 1.671e-08 -7.745 -7.777 -0.032 (0)

Cr(OH)4- 1.519e-08 1.410e-08 -7.818 -7.851 -0.032 (0)

Cr(OH)+2 1.027e-08 7.623e-09 -7.988 -8.118 -0.130 (0)

CrOHSO4 5.614e-11 5.614e-11 -10.251 -10.251 0.000 (0)

Cr+3 1.176e-12 6.007e-13 -11.930 -12.221 -0.292 (0)

CrSO4+ 1.278e-13 1.186e-13 -12.893 -12.926 -0.032 (0)

CrCl+2 2.708e-17 2.010e-17 -16.567 -16.697 -0.130 (0)

Cr2(OH)2SO4+2 9.865e-18 7.319e-18 -17.006 -17.136 -0.130 (0)

CrNO3+2 2.127e-18 1.578e-18 -17.672 -17.802 -0.130 (0)

Cr2(OH)2(SO4)2 7.132e-20 7.132e-20 -19.147 -19.147 0.000 (0)

CrOHCl2 3.292e-21 3.292e-21 -20.483 -20.483 0.000 (0)

CrCl2+ 2.338e-23 2.170e-23 -22.631 -22.664 -0.032 (0)

Cr(6) 5.296e-14

CrO4-2 5.218e-14 3.966e-14 -13.283 -13.402 -0.119 (0)

HCrO4- 7.616e-16 7.068e-16 -15.118 -15.151 -0.032 (0)

NaCrO4- 1.943e-17 1.803e-17 -16.712 -16.744 -0.032 (0)

KCrO4- 4.218e-18 3.915e-18 -17.375 -17.407 -0.032 (0)

H2CrO4 1.494e-24 1.494e-24 -23.826 -23.826 0.000 (0)

CrO3SO4-2 2.856e-25 2.119e-25 -24.544 -24.674 -0.130 (0)

CrO3Cl- 1.295e-27 1.202e-27 -26.888 -26.920 -0.032 (0)

Cr2O7-2 3.504e-29 2.600e-29 -28.455 -28.585 -0.130 (0)

Cu(1) 2.417e-11

Cu+ 2.295e-11 2.130e-11 -10.639 -10.672 -0.032 (0)

CuCl 1.208e-12 1.208e-12 -11.918 -11.918 0.000 (0)

CuCl2- 1.265e-14 1.181e-14 -13.898 -13.928 -0.030 (0)

CuCl3-2 1.402e-19 1.070e-19 -18.853 -18.970 -0.117 (0)

Cu(2) 5.262e-08

CuCO3 4.683e-08 4.683e-08 -7.330 -7.330 0.000 (0)

Cu(CO3)2-2 2.680e-09 1.989e-09 -8.572 -8.701 -0.130 (0)

CuOH+ 1.391e-09 1.299e-09 -8.857 -8.887 -0.030 (0)

Cu(OH)2 9.755e-10 9.755e-10 -9.011 -9.011 0.000 (0)

Cu+2 6.631e-10 5.040e-10 -9.178 -9.298 -0.119 (0)

CuHCO3+ 6.629e-11 6.152e-11 -10.179 -10.211 -0.032 (0)

CuSO4 1.572e-11 1.572e-11 -10.803 -10.803 0.000 (0)

Cu(OH)3- 3.776e-12 3.504e-12 -11.423 -11.455 -0.032 (0)

CuNO3+ 4.441e-13 4.121e-13 -12.353 -12.385 -0.032 (0)

Cu2(OH)2+2 5.130e-14 3.807e-14 -13.290 -13.419 -0.130 (0)

CuCl+ 3.229e-14 3.014e-14 -13.491 -13.521 -0.030 (0)

Cu(OH)4-2 6.511e-17 4.831e-17 -16.186 -16.316 -0.130 (0)

Cu(NO3)2 1.126e-17 1.126e-17 -16.948 -16.948 0.000 (0)

CuCl2 2.187e-19 2.187e-19 -18.660 -18.660 0.000 (0)

CuCl3- 7.447e-26 6.952e-26 -25.128 -25.158 -0.030 (0)

CuCl4-2 3.487e-32 2.663e-32 -31.457 -31.575 -0.117 (0)

H(0) 5.471e-28

H2 2.735e-28 2.738e-28 -27.563 -27.563 0.000 (0)

K 2.849e-05

K+ 2.846e-05 2.657e-05 -4.546 -4.576 -0.030 (0)

KSO4- 3.023e-08 2.827e-08 -7.520 -7.549 -0.029 (0)

KCrO4- 4.218e-18 3.915e-18 -17.375 -17.407 -0.032 (0)

Mg 5.607e-04

Mg+2 5.349e-04 4.066e-04 -3.272 -3.391 -0.119 (0)

MgHCO3+ 1.081e-05 1.010e-05 -4.966 -4.996 -0.030 (0)

MgSO4 1.072e-05 1.072e-05 -4.970 -4.970 0.000 (0)

MgCO3 4.129e-06 4.129e-06 -5.384 -5.384 0.000 (0)

MgOH+ 7.107e-08 6.656e-08 -7.148 -7.177 -0.028 (0)

N(5) 2.541e-04

NO3- 2.537e-04 2.369e-04 -3.596 -3.625 -0.030 (0)

CaNO3+ 3.622e-07 3.361e-07 -6.441 -6.473 -0.032 (0)

ZnNO3+ 4.187e-11 3.886e-11 -10.378 -10.411 -0.032 (0)

CdNO3+ 9.231e-13 8.567e-13 -12.035 -12.067 -0.032 (0)

PbNO3+ 7.703e-13 7.150e-13 -12.113 -12.146 -0.032 (0)

CuNO3+ 4.441e-13 4.121e-13 -12.353 -12.385 -0.032 (0)

Zn(NO3)2 1.665e-15 1.665e-15 -14.779 -14.779 0.000 (0)

Pb(NO3)2 3.457e-16 3.457e-16 -15.461 -15.461 0.000 (0)

Cd(NO3)2 6.495e-17 6.495e-17 -16.187 -16.187 0.000 (0)

Cu(NO3)2 1.126e-17 1.126e-17 -16.948 -16.948 0.000 (0)

CrNO3+2 2.127e-18 1.578e-18 -17.672 -17.802 -0.130 (0)

Na 9.832e-05

Na+ 9.801e-05 9.151e-05 -4.009 -4.039 -0.030 (0)

NaHCO3 1.824e-07 1.824e-07 -6.739 -6.739 0.000 (0)

NaSO4- 8.437e-08 7.891e-08 -7.074 -7.103 -0.029 (0)

NaCO3- 4.441e-08 4.154e-08 -7.353 -7.382 -0.029 (0)

NaCrO4- 1.943e-17 1.803e-17 -16.712 -16.744 -0.032 (0)

O(0) 0.000e+00

O2 0.000e+00 0.000e+00 -41.498 -41.497 0.000 (0)

Pb 1.241e-08

PbCO3 1.010e-08 1.010e-08 -7.996 -7.996 0.000 (0)

PbOH+ 1.009e-09 9.368e-10 -8.996 -9.028 -0.032 (0)

Pb(CO3)2-2 6.194e-10 4.596e-10 -9.208 -9.338 -0.130 (0)

PbHCO3+ 3.298e-10 3.061e-10 -9.482 -9.514 -0.032 (0)

Pb+2 2.802e-10 2.129e-10 -9.553 -9.672 -0.119 (0)

Pb(OH)2 5.189e-11 5.189e-11 -10.285 -10.285 0.000 (0)

PbSO4 1.711e-11 1.711e-11 -10.767 -10.767 0.000 (0)

PbNO3+ 7.703e-13 7.150e-13 -12.113 -12.146 -0.032 (0)

PbCl+ 3.046e-13 2.827e-13 -12.516 -12.549 -0.032 (0)

Pb(OH)3- 9.792e-14 9.088e-14 -13.009 -13.042 -0.032 (0)

Pb(SO4)2-2 2.279e-14 1.691e-14 -13.642 -13.772 -0.130 (0)

Pb(NO3)2 3.457e-16 3.457e-16 -15.461 -15.461 0.000 (0)

PbCl2 5.301e-17 5.301e-17 -16.276 -16.276 0.000 (0)

Pb(OH)4-2 5.254e-17 3.898e-17 -16.280 -16.409 -0.130 (0)

Pb2OH+3 6.188e-18 3.162e-18 -17.208 -17.500 -0.292 (0)

Pb3(OH)4+2 1.314e-21 9.747e-22 -20.882 -21.011 -0.130 (0)

PbCl3- 1.216e-21 1.128e-21 -20.915 -20.948 -0.032 (0)

PbCl4-2 2.488e-26 1.846e-26 -25.604 -25.734 -0.130 (0)

Pb4(OH)4+4 9.685e-27 2.936e-27 -26.014 -26.532 -0.518 (0)

S(6) 2.396e-04

SO4-2 2.158e-04 1.640e-04 -3.666 -3.785 -0.119 (0)

CaSO4 1.291e-05 1.291e-05 -4.889 -4.889 0.000 (0)

MgSO4 1.072e-05 1.072e-05 -4.970 -4.970 0.000 (0)

NaSO4- 8.437e-08 7.891e-08 -7.074 -7.103 -0.029 (0)

KSO4- 3.023e-08 2.827e-08 -7.520 -7.549 -0.029 (0)

ZnSO4 1.860e-09 1.860e-09 -8.730 -8.730 0.000 (0)

HSO4- 6.164e-11 5.758e-11 -10.210 -10.240 -0.030 (0)

CrOHSO4 5.614e-11 5.614e-11 -10.251 -10.251 0.000 (0)

CdSO4 2.331e-11 2.331e-11 -10.632 -10.632 0.000 (0)

PbSO4 1.711e-11 1.711e-11 -10.767 -10.767 0.000 (0)

CuSO4 1.572e-11 1.572e-11 -10.803 -10.803 0.000 (0)

Zn(SO4)2-2 4.089e-12 3.034e-12 -11.388 -11.518 -0.130 (0)

CrSO4+ 1.278e-13 1.186e-13 -12.893 -12.926 -0.032 (0)

Cd(SO4)2-2 8.371e-14 6.211e-14 -13.077 -13.207 -0.130 (0)

Pb(SO4)2-2 2.279e-14 1.691e-14 -13.642 -13.772 -0.130 (0)

Cr2(OH)2SO4+2 9.865e-18 7.319e-18 -17.006 -17.136 -0.130 (0)

Cr2(OH)2(SO4)2 7.132e-20 7.132e-20 -19.147 -19.147 0.000 (0)

CrO3SO4-2 2.856e-25 2.119e-25 -24.544 -24.674 -0.130 (0)

Zn 1.437e-07

Zn+2 7.786e-08 5.918e-08 -7.109 -7.228 -0.119 (0)

ZnCO3 5.373e-08 5.373e-08 -7.270 -7.270 0.000 (0)

ZnHCO3+ 3.901e-09 3.621e-09 -8.409 -8.441 -0.032 (0)

ZnOH+ 3.389e-09 3.145e-09 -8.470 -8.502 -0.032 (0)

Zn(OH)2 2.877e-09 2.877e-09 -8.541 -8.541 0.000 (0)

ZnSO4 1.860e-09 1.860e-09 -8.730 -8.730 0.000 (0)

ZnNO3+ 4.187e-11 3.886e-11 -10.378 -10.411 -0.032 (0)

Zn(OH)3- 2.721e-11 2.526e-11 -10.565 -10.598 -0.032 (0)

ZnOHCl 1.536e-11 1.536e-11 -10.814 -10.814 0.000 (0)

ZnCl+ 6.393e-12 5.968e-12 -11.194 -11.224 -0.030 (0)

Zn(SO4)2-2 4.089e-12 3.034e-12 -11.388 -11.518 -0.130 (0)

Zn(OH)4-2 2.373e-15 1.761e-15 -14.625 -14.754 -0.130 (0)

Zn(NO3)2 1.665e-15 1.665e-15 -14.779 -14.779 0.000 (0)

ZnCl2 2.169e-16 2.169e-16 -15.664 -15.664 0.000 (0)

ZnCl3- 7.800e-21 7.282e-21 -20.108 -20.138 -0.030 (0)

ZnCl4-2 1.896e-25 1.448e-25 -24.722 -24.839 -0.117 (0)

------------------------------Saturation indices-------------------------------

Phase SI** log IAP log K(283 K, 1 atm)

Anglesite -5.56 -13.46 -7.90 PbSO4

Anhydrite -2.89 -7.18 -4.29 CaSO4

Antlerite -7.50 1.28 8.79 Cu3(OH)4SO4

Aragonite -0.01 -8.20 -8.19 CaCO3

Arsenolite -77.68 -81.00 -3.32 As4O6

Artinite -5.82 4.90 10.72 MgCO3:Mg(OH)2:3H2O

As2O5 -38.58 -31.67 6.92 As2O5

Atacamite -6.48 1.78 8.26 Cu2(OH)3Cl

Azurite -4.99 -21.02 -16.02 Cu3(OH)2(CO3)2

Bianchite -9.25 -11.01 -1.76 ZnSO4:6H2O

Brochantite -8.64 8.47 17.10 Cu4(OH)6SO4

Brucite -4.81 13.09 17.90 Mg(OH)2

Ca3(AsO4)2:4H2O -14.72 7.58 22.30 Ca3(AsO4)2:4H2O

CaCrO4 -14.78 -16.80 -2.02 CaCrO4

Calcite 0.21 -8.20 -8.41 CaCO3

Cd(OH)2 -7.18 7.34 14.52 Cd(OH)2

Cd(OH)2(am) -7.19 7.34 14.54 Cd(OH)2

Cd3(OH)2(SO4)2 -25.21 -18.50 6.71 Cd3(OH)2(SO4)2

Cd3(OH)4SO4 -20.79 1.77 22.56 Cd3(OH)4SO4

Cd4(OH)6SO4 -19.29 9.11 28.40 Cd4(OH)6SO4

CdCl2 -17.34 -17.83 -0.49 CdCl2

CdCl2:1H2O -16.21 -17.83 -1.62 CdCl2:1H2O

CdCl2:2.5H2O -15.85 -17.83 -1.98 CdCl2:2.5H2O

Cdmetal(alpha) -31.35 -17.14 14.21 Cd

Cdmetal(gamma) -31.46 -17.14 14.32 Cd

CdOHCl -9.07 -5.24 3.82 CdOHCl

CdSO4 -13.23 -12.92 0.31 CdSO4

CdSO4:1H2O -11.49 -12.92 -1.43 CdSO4:1H2O

CdSO4:2.67H2O -11.22 -12.92 -1.71 CdSO4:2.67H2O

Cerussite -1.11 -14.47 -13.36 PbCO3

CH4(g) -75.77 -119.21 -43.43 CH4

Chalcanthite -10.39 -13.08 -2.70 CuSO4:5H2O

Claudetite -77.42 -81.00 -3.58 As4O6

CO2(g) -3.10 -21.28 -18.18 CO2

Cotunnite -13.34 -18.36 -5.02 PbCl2

Cr(OH)2 -18.77 -7.62 11.15 Cr(OH)2

Cr(OH)3 0.12 1.73 1.61 Cr(OH)3

Cr(OH)3(am) 2.48 1.73 -0.75 Cr(OH)3

Cr2O3 5.34 3.46 -1.89 Cr2O3

CrCl2 -47.92 -32.80 15.12 CrCl2

CrCl3 -52.27 -36.03 16.24 CrCl3

Crmetal -64.18 -32.11 32.08 Cr

CrO3 -26.72 -29.88 -3.16 CrO3

Cu(OH)2 -2.01 7.18 9.20 Cu(OH)2

Cu2(OH)3NO3 -7.42 2.50 9.92 Cu2(OH)3NO3

Cu2SO4 -23.36 -25.13 -1.77 Cu2SO4

Cu3(AsO4)2:2H2O -16.22 -10.12 6.10 Cu3(AsO4)2:2H2O

CuCO3 -2.60 -14.10 -11.50 CuCO3

CuCrO4 -17.26 -22.70 -5.44 CuCrO4

Cumetal -5.25 -14.67 -9.42 Cu

CuOCuSO4 -17.48 -5.90 11.58 CuOCuSO4

Cuprite -4.61 -4.86 -0.26 Cu2O

CuSO4 -16.70 -13.08 3.62 CuSO4

Dolomite(disordered) -0.28 -16.39 -16.11 CaMg(CO3)2

Dolomite(ordered) 0.33 -16.39 -16.72 CaMg(CO3)2

Epsomite -4.94 -7.18 -2.23 MgSO4:7H2O

Goslarite -8.87 -11.01 -2.14 ZnSO4:7H2O

Gypsum -2.56 -7.18 -4.62 CaSO4:2H2O

Halite -9.95 -8.38 1.57 NaCl

Huntite -3.81 -32.78 -28.97 CaMg3(CO3)4

Hydrocerussite -3.37 -22.14 -18.77 Pb3(OH)2(CO3)2

Hydromagnesite -12.94 -19.68 -6.74 Mg5(CO3)4(OH)2:4H2O

K2Cr2O7 -34.44 -52.44 -17.99 K2Cr2O7

K2CrO4 -21.87 -22.55 -0.68 K2CrO4

Langite -10.56 8.47 19.03 Cu4(OH)6SO4:H2O

Larnakite -6.42 -6.65 -0.23 PbO:PbSO4

Laurionite -6.40 -5.78 0.62 PbOHCl

Lime -21.42 13.08 34.50 CaO

Litharge -6.49 6.81 13.30 PbO

Magnesite -0.55 -8.19 -7.65 MgCO3

Malachite -0.90 -6.92 -6.01 Cu2(OH)2CO3

Massicot -6.71 6.81 13.51 PbO

Melanothallite -24.84 -17.99 6.85 CuCl2

Mg(OH)2(active) -5.70 13.09 18.79 Mg(OH)2

MgCr2O4 -1.32 16.55 17.87 MgCr2O4

MgCrO4 -23.00 -16.79 6.21 MgCrO4

Minium -32.53 44.91 77.44 Pb3O4

Mirabilite -10.01 -11.86 -1.85 Na2SO4:10H2O

Monteponite -8.72 7.34 16.06 CdO

Na2Cr2O7 -41.26 -51.36 -10.10 Na2Cr2O7

Na2CrO4 -24.59 -21.48 3.11 Na2CrO4

Nantokite -7.89 -15.02 -7.13 CuCl

Natron -10.96 -12.88 -1.92 Na2CO3:10H2O

Nesquehonite -3.75 -8.19 -4.45 MgCO3:3H2O

O2(g) -39.43 48.96 88.39 O2

Otavite -1.94 -13.94 -11.99 CdCO3

Pb(OH)2 -1.88 6.81 8.69 Pb(OH)2

Pb10(OH)6O(CO3)6 -50.85 -59.61 -8.76 Pb10(OH)6O(CO3)6

Pb2(OH)3Cl -7.76 1.03 8.79 Pb2(OH)3Cl

Pb2O(OH)2 -12.57 13.62 26.19 Pb2O(OH)2

Pb2O3 -22.94 38.10 61.04 Pb2O3

Pb2OCO3 -7.49 -7.66 -0.18 Pb2OCO3

Pb3(AsO4)2 -17.04 -11.24 5.80 Pb3(AsO4)2

Pb3O2CO3 -12.90 -0.86 12.05 Pb3O2CO3

Pb3O2SO4 -11.26 0.16 11.42 Pb3O2SO4

Pb4(OH)6SO4 -14.13 6.97 21.10 Pb4(OH)6SO4

Pb4O3SO4 -16.17 6.97 23.14 Pb4O3SO4

PbCrO4 -10.06 -23.07 -13.01 PbCrO4

Pbmetal -21.91 -17.67 4.24 Pb

PbO:0.3H2O -6.17 6.81 12.98 PbO:0.33H2O

Periclase -9.90 13.09 22.99 MgO

Phosgenite -13.03 -32.84 -19.81 PbCl2:PbCO3

Plattnerite -21.06 31.29 52.35 PbO2

Portlandite -10.92 13.08 24.00 Ca(OH)2

Smithsonite -2.18 -12.03 -9.85 ZnCO3

Tenorite -1.06 7.18 8.25 CuO

Thenardite -12.27 -11.86 0.41 Na2SO4

Thermonatrite -13.61 -12.88 0.73 Na2CO3:H2O

Zincite -2.91 9.25 12.17 ZnO

Zincosite -15.71 -11.01 4.70 ZnSO4

Zn(NO3)2:6H2O -17.57 -14.48 3.09 Zn(NO3)2:6H2O

Zn(OH)2 -2.95 9.25 12.20 Zn(OH)2

Zn(OH)2(am) -3.97 9.25 13.22 Zn(OH)2

Zn(OH)2(beta) -3.27 9.25 12.53 Zn(OH)2

Zn(OH)2(epsilon) -3.04 9.25 12.29 Zn(OH)2

Zn(OH)2(gamma) -2.48 9.25 11.73 Zn(OH)2

Zn2(OH)2SO4 -9.26 -1.76 7.50 Zn2(OH)2SO4

Zn2(OH)3Cl -9.27 5.92 15.19 Zn2(OH)3Cl

Zn3(AsO4)2:2.5H2O -17.56 -3.91 13.65 Zn3(AsO4)2:2.5H2O

Zn3O(SO4)2 -34.08 -12.77 21.31 Zn3O(SO4)2

Zn4(OH)6SO4 -11.65 16.75 28.40 Zn4(OH)6SO4

Zn5(OH)8Cl2 -17.41 21.09 38.50 Zn5(OH)8Cl2

ZnCl2 -23.64 -15.92 7.72 ZnCl2

ZnCO3:1H2O -1.77 -12.03 -10.26 ZnCO3:1H2O

Znmetal -42.44 -15.23 27.21 Zn

ZnO(active) -2.76 9.25 12.01 ZnO

ZnSO4:1H2O -10.78 -11.01 -0.23 ZnSO4:1H2O

**For a gas, SI = log10(fugacity). Fugacity = pressure * phi / 1 atm.

For ideal gases, phi = 1.

Initial solution 18.

----------------------------Distribution of species----------------------------

Log Log Log mole V

Species Molality Activity Molality Activity Gamma cm?mol

OH- 1.918e-06 1.758e-06 -5.717 -5.755 -0.038 (0)

H+ 1.894e-09 1.738e-09 -8.723 -8.760 -0.037 0.00

H2O 5.551e+01 9.999e-01 1.744 -0.000 0.000 18.02

As(3) 5.444e-23

H3AsO3 4.610e-23 4.610e-23 -22.336 -22.336 0.000 (0)

H2AsO3- 8.335e-24 7.574e-24 -23.079 -23.121 -0.042 (0)

HAsO3-2 2.942e-27 2.006e-27 -26.531 -26.698 -0.166 (0)

AsO3-3 6.131e-32 2.591e-32 -31.212 -31.587 -0.374 (0)

H4AsO3+ 4.368e-32 3.969e-32 -31.360 -31.401 -0.042 (0)

As(5) 4.474e-09

HAsO4-2 4.409e-09 3.006e-09 -8.356 -8.522 -0.166 (0)

H2AsO4- 5.591e-11 5.080e-11 -10.253 -10.294 -0.042 (0)

AsO4-3 9.004e-12 3.804e-12 -11.046 -11.420 -0.374 (0)

H3AsO4 1.316e-17 1.318e-17 -16.881 -16.880 0.001 (0)

C(4) 4.653e-03

HCO3- 4.381e-03 4.030e-03 -2.358 -2.395 -0.036 (0)

CO3-2 1.122e-04 7.958e-05 -3.950 -4.099 -0.149 (0)

CaCO3 5.208e-05 5.208e-05 -4.283 -4.283 0.000 (0)

CaHCO3+ 3.089e-05 2.845e-05 -4.510 -4.546 -0.036 (0)

MgCO3 3.057e-05 3.057e-05 -4.515 -4.515 0.000 (0)

MgHCO3+ 2.463e-05 2.260e-05 -4.608 -4.646 -0.037 (0)

H2CO3 1.916e-05 1.916e-05 -4.718 -4.718 0.000 (0)

NaHCO3 1.104e-06 1.104e-06 -5.957 -5.957 0.000 (0)

NaCO3- 9.041e-07 8.318e-07 -6.044 -6.080 -0.036 (0)

ZnCO3 3.232e-08 3.232e-08 -7.490 -7.490 0.000 (0)

CuCO3 9.895e-09 9.895e-09 -8.005 -8.005 0.000 (0)

Cu(CO3)2-2 3.108e-09 2.120e-09 -8.507 -8.674 -0.166 (0)

PbCO3 2.395e-09 2.395e-09 -8.621 -8.621 0.000 (0)

CdCO3 8.823e-10 8.823e-10 -9.054 -9.054 0.000 (0)

Pb(CO3)2-2 8.062e-10 5.497e-10 -9.094 -9.260 -0.166 (0)

ZnHCO3+ 7.246e-10 6.585e-10 -9.140 -9.181 -0.042 (0)

Cd(CO3)2-2 7.633e-11 5.205e-11 -10.117 -10.284 -0.166 (0)

PbHCO3+ 2.415e-11 2.194e-11 -10.617 -10.659 -0.042 (0)

CuHCO3+ 4.325e-12 3.930e-12 -11.364 -11.406 -0.042 (0)

CdHCO3+ 3.595e-12 3.267e-12 -11.444 -11.486 -0.042 (0)

Ca 9.333e-04

Ca+2 8.197e-04 5.812e-04 -3.086 -3.236 -0.149 (0)

CaCO3 5.208e-05 5.208e-05 -4.283 -4.283 0.000 (0)

CaHCO3+ 3.089e-05 2.845e-05 -4.510 -4.546 -0.036 (0)

CaSO4 2.984e-05 2.984e-05 -4.525 -4.525 0.000 (0)

CaNO3+ 7.737e-07 7.031e-07 -6.111 -6.153 -0.042 (0)

CaOH+ 1.854e-08 1.707e-08 -7.732 -7.768 -0.036 (0)

Cd 1.688e-09

CdCO3 8.823e-10 8.823e-10 -9.054 -9.054 0.000 (0)

Cd+2 6.860e-10 4.864e-10 -9.164 -9.313 -0.149 (0)

Cd(CO3)2-2 7.633e-11 5.205e-11 -10.117 -10.284 -0.166 (0)

CdSO4 2.469e-11 2.469e-11 -10.607 -10.607 0.000 (0)

CdOH+ 7.635e-12 6.938e-12 -11.117 -11.159 -0.042 (0)

CdCl+ 5.167e-12 4.695e-12 -11.287 -11.328 -0.042 (0)

CdHCO3+ 3.595e-12 3.267e-12 -11.444 -11.486 -0.042 (0)

CdNO3+ 9.037e-13 8.212e-13 -12.044 -12.086 -0.042 (0)

Cd(OH)2 8.182e-13 8.182e-13 -12.087 -12.087 0.000 (0)

CdOHCl 7.723e-13 7.723e-13 -12.112 -12.112 0.000 (0)

Cd(SO4)2-2 1.534e-13 1.046e-13 -12.814 -12.980 -0.166 (0)

CdCl2 1.937e-15 1.937e-15 -14.713 -14.713 0.000 (0)

Cd(OH)3- 3.187e-16 2.896e-16 -15.497 -15.538 -0.042 (0)

Cd(NO3)2 8.955e-17 8.955e-17 -16.048 -16.048 0.000 (0)

CdCl3- 1.196e-19 1.087e-19 -18.922 -18.964 -0.042 (0)

Cd2OH+3 4.852e-20 2.050e-20 -19.314 -19.688 -0.374 (0)

Cd(OH)4-2 4.028e-22 2.746e-22 -21.395 -21.561 -0.166 (0)

Cl 1.125e-04

Cl- 1.125e-04 1.033e-04 -3.949 -3.986 -0.037 (0)

ZnOHCl 1.389e-11 1.389e-11 -10.857 -10.857 0.000 (0)

CdCl+ 5.167e-12 4.695e-12 -11.287 -11.328 -0.042 (0)

ZnCl+ 1.778e-12 1.631e-12 -11.750 -11.787 -0.037 (0)

CdOHCl 7.723e-13 7.723e-13 -12.112 -12.112 0.000 (0)

CuCl 1.160e-13 1.160e-13 -12.935 -12.935 0.000 (0)

PbCl+ 3.352e-14 3.046e-14 -13.475 -13.516 -0.042 (0)

CuCl+ 3.155e-15 2.894e-15 -14.501 -14.538 -0.037 (0)

CuCl2- 2.833e-15 2.599e-15 -14.548 -14.585 -0.037 (0)

CdCl2 1.937e-15 1.937e-15 -14.713 -14.713 0.000 (0)

ZnCl2 1.359e-16 1.359e-16 -15.867 -15.867 0.000 (0)

PbCl2 1.309e-17 1.309e-17 -16.883 -16.883 0.000 (0)

CrCl+2 2.578e-18 1.758e-18 -17.589 -17.755 -0.166 (0)

CdCl3- 1.196e-19 1.087e-19 -18.922 -18.964 -0.042 (0)

CuCl3-2 7.563e-20 5.400e-20 -19.121 -19.268 -0.146 (0)

CuCl2 4.814e-20 4.814e-20 -19.318 -19.318 0.000 (0)

ZnCl3- 1.140e-20 1.046e-20 -19.943 -19.981 -0.037 (0)

CrOHCl2 2.183e-21 2.183e-21 -20.661 -20.661 0.000 (0)

PbCl3- 7.028e-22 6.386e-22 -21.153 -21.195 -0.042 (0)

CrCl2+ 4.786e-24 4.349e-24 -23.320 -23.362 -0.042 (0)

ZnCl4-2 6.673e-25 4.764e-25 -24.176 -24.322 -0.146 (0)

CrO3Cl- 1.516e-25 1.377e-25 -24.819 -24.861 -0.042 (0)

CuCl3- 3.823e-26 3.507e-26 -25.418 -25.455 -0.037 (0)

PbCl4-2 3.512e-26 2.395e-26 -25.454 -25.621 -0.166 (0)

CuCl4-2 4.312e-32 3.079e-32 -31.365 -31.512 -0.146 (0)

Cr(2) 4.393e-26

Cr+2 4.393e-26 2.996e-26 -25.357 -25.524 -0.166 (0)

Cr(3) 5.769e-07

Cr(OH)3 2.793e-07 2.793e-07 -6.554 -6.554 0.000 (0)

Cr(OH)2+ 1.412e-07 1.284e-07 -6.850 -6.892 -0.042 (0)

CrO2- 8.402e-08 7.635e-08 -7.076 -7.117 -0.042 (0)

Cr(OH)4- 7.090e-08 6.443e-08 -7.149 -7.191 -0.042 (0)

Cr(OH)+2 1.411e-09 9.623e-10 -8.850 -9.017 -0.166 (0)

CrOHSO4 1.127e-11 1.127e-11 -10.948 -10.948 0.000 (0)

Cr+3 5.425e-14 2.292e-14 -13.266 -13.640 -0.374 (0)

CrSO4+ 7.920e-15 7.197e-15 -14.101 -14.143 -0.042 (0)

CrCl+2 2.578e-18 1.758e-18 -17.589 -17.755 -0.166 (0)

Cr2(OH)2SO4+2 2.720e-19 1.854e-19 -18.565 -18.732 -0.166 (0)

CrNO3+2 1.271e-19 8.665e-20 -18.896 -19.062 -0.166 (0)

Cr2(OH)2(SO4)2 2.873e-21 2.873e-21 -20.542 -20.542 0.000 (0)

CrOHCl2 2.183e-21 2.183e-21 -20.661 -20.661 0.000 (0)

CrCl2+ 4.786e-24 4.349e-24 -23.320 -23.362 -0.042 (0)

Cr(6) 3.077e-11

CrO4-2 3.060e-11 2.170e-11 -10.514 -10.664 -0.149 (0)

HCrO4- 1.286e-13 1.169e-13 -12.891 -12.932 -0.042 (0)

NaCrO4- 4.311e-14 3.917e-14 -13.365 -13.407 -0.042 (0)

KCrO4- 4.653e-15 4.228e-15 -14.332 -14.374 -0.042 (0)

H2CrO4 7.469e-23 7.469e-23 -22.127 -22.127 0.000 (0)

CrO3SO4-2 2.470e-23 1.684e-23 -22.607 -22.774 -0.166 (0)

Cr2O7-2 1.043e-24 7.113e-25 -23.982 -24.148 -0.166 (0)

CrO3Cl- 1.516e-25 1.377e-25 -24.819 -24.861 -0.042 (0)

Cu(1) 1.101e-12

Cu+ 9.821e-13 8.924e-13 -12.008 -12.049 -0.042 (0)

CuCl 1.160e-13 1.160e-13 -12.935 -12.935 0.000 (0)

CuCl2- 2.833e-15 2.599e-15 -14.548 -14.585 -0.037 (0)

CuCl3-2 7.563e-20 5.400e-20 -19.121 -19.268 -0.146 (0)

Cu(2) 1.369e-08

CuCO3 9.895e-09 9.895e-09 -8.005 -8.005 0.000 (0)

Cu(CO3)2-2 3.108e-09 2.120e-09 -8.507 -8.674 -0.166 (0)

Cu(OH)2 4.472e-10 4.472e-10 -9.350 -9.350 0.000 (0)

CuOH+ 1.962e-10 1.800e-10 -9.707 -9.745 -0.037 (0)

Cu+2 2.978e-11 2.112e-11 -10.526 -10.675 -0.149 (0)

Cu(OH)3- 5.848e-12 5.314e-12 -11.233 -11.275 -0.042 (0)

CuHCO3+ 4.325e-12 3.930e-12 -11.364 -11.406 -0.042 (0)

CuSO4 1.048e-12 1.048e-12 -11.980 -11.980 0.000 (0)

CuNO3+ 2.734e-14 2.484e-14 -13.563 -13.605 -0.042 (0)

CuCl+ 3.155e-15 2.894e-15 -14.501 -14.538 -0.037 (0)

Cu2(OH)2+2 1.072e-15 7.312e-16 -14.970 -15.136 -0.166 (0)

Cu(OH)4-2 3.554e-16 2.423e-16 -15.449 -15.616 -0.166 (0)

Cu(NO3)2 9.766e-19 9.766e-19 -18.010 -18.010 0.000 (0)

CuCl2 4.814e-20 4.814e-20 -19.318 -19.318 0.000 (0)

CuCl3- 3.823e-26 3.507e-26 -25.418 -25.455 -0.037 (0)

CuCl4-2 4.312e-32 3.079e-32 -31.365 -31.512 -0.146 (0)

H(0) 4.996e-29

H2 2.498e-29 2.502e-29 -28.602 -28.602 0.001 (0)

K 5.726e-05

K+ 5.716e-05 5.246e-05 -4.243 -4.280 -0.037 (0)

KSO4- 9.645e-08 8.873e-08 -7.016 -7.052 -0.036 (0)

KCrO4- 4.653e-15 4.228e-15 -14.332 -14.374 -0.042 (0)

Mg 9.222e-04

Mg+2 8.417e-04 5.968e-04 -3.075 -3.224 -0.149 (0)

MgCO3 3.057e-05 3.057e-05 -4.515 -4.515 0.000 (0)

MgSO4 2.502e-05 2.502e-05 -4.602 -4.602 0.000 (0)

MgHCO3+ 2.463e-05 2.260e-05 -4.608 -4.646 -0.037 (0)

MgOH+ 3.506e-07 3.232e-07 -6.455 -6.491 -0.035 (0)

N(5) 3.722e-04

NO3- 3.714e-04 3.408e-04 -3.430 -3.467 -0.037 (0)

CaNO3+ 7.737e-07 7.031e-07 -6.111 -6.153 -0.042 (0)

ZnNO3+ 7.337e-12 6.667e-12 -11.134 -11.176 -0.042 (0)

CdNO3+ 9.037e-13 8.212e-13 -12.044 -12.086 -0.042 (0)

PbNO3+ 5.322e-14 4.836e-14 -13.274 -13.316 -0.042 (0)

CuNO3+ 2.734e-14 2.484e-14 -13.563 -13.605 -0.042 (0)

Zn(NO3)2 4.110e-16 4.110e-16 -15.386 -15.386 0.000 (0)

Cd(NO3)2 8.955e-17 8.955e-17 -16.048 -16.048 0.000 (0)

Pb(NO3)2 3.364e-17 3.364e-17 -16.473 -16.473 0.000 (0)

Cu(NO3)2 9.766e-19 9.766e-19 -18.010 -18.010 0.000 (0)

CrNO3+2 1.271e-19 8.665e-20 -18.896 -19.062 -0.166 (0)

Na 3.985e-04

Na+ 3.959e-04 3.633e-04 -3.402 -3.440 -0.037 (0)

NaHCO3 1.104e-06 1.104e-06 -5.957 -5.957 0.000 (0)

NaCO3- 9.041e-07 8.318e-07 -6.044 -6.080 -0.036 (0)

NaSO4- 5.415e-07 4.981e-07 -6.266 -6.303 -0.036 (0)

NaCrO4- 4.311e-14 3.917e-14 -13.365 -13.407 -0.042 (0)

O(0) 7.606e-40

O2 3.803e-40 3.809e-40 -39.420 -39.419 0.001 (0)

Pb 3.428e-09

PbCO3 2.395e-09 2.395e-09 -8.621 -8.621 0.000 (0)

Pb(CO3)2-2 8.062e-10 5.497e-10 -9.094 -9.260 -0.166 (0)

PbOH+ 1.603e-10 1.457e-10 -9.795 -9.837 -0.042 (0)

Pb(OH)2 2.669e-11 2.669e-11 -10.574 -10.574 0.000 (0)

PbHCO3+ 2.415e-11 2.194e-11 -10.617 -10.659 -0.042 (0)

Pb+2 1.412e-11 1.001e-11 -10.850 -11.000 -0.149 (0)

PbSO4 1.279e-12 1.279e-12 -11.893 -11.893 0.000 (0)

Pb(OH)3- 1.702e-13 1.546e-13 -12.769 -12.811 -0.042 (0)

PbNO3+ 5.322e-14 4.836e-14 -13.274 -13.316 -0.042 (0)

PbCl+ 3.352e-14 3.046e-14 -13.475 -13.516 -0.042 (0)

Pb(SO4)2-2 2.947e-15 2.009e-15 -14.531 -14.697 -0.166 (0)

Pb(OH)4-2 3.218e-16 2.194e-16 -15.492 -15.659 -0.166 (0)

Pb(NO3)2 3.364e-17 3.364e-17 -16.473 -16.473 0.000 (0)

PbCl2 1.309e-17 1.309e-17 -16.883 -16.883 0.000 (0)

Pb2OH+3 5.471e-20 2.312e-20 -19.262 -19.636 -0.374 (0)

PbCl3- 7.028e-22 6.386e-22 -21.153 -21.195 -0.042 (0)

Pb3(OH)4+2 1.778e-23 1.213e-23 -22.750 -22.916 -0.166 (0)

PbCl4-2 3.512e-26 2.395e-26 -25.454 -25.621 -0.166 (0)

Pb4(OH)4+4 7.942e-30 1.717e-30 -29.100 -29.765 -0.665 (0)

S(6) 4.233e-04

SO4-2 3.678e-04 2.608e-04 -3.434 -3.584 -0.149 (0)

CaSO4 2.984e-05 2.984e-05 -4.525 -4.525 0.000 (0)

MgSO4 2.502e-05 2.502e-05 -4.602 -4.602 0.000 (0)

NaSO4- 5.415e-07 4.981e-07 -6.266 -6.303 -0.036 (0)

KSO4- 9.645e-08 8.873e-08 -7.016 -7.052 -0.036 (0)

ZnSO4 3.528e-10 3.528e-10 -9.453 -9.453 0.000 (0)

HSO4- 3.014e-11 2.768e-11 -10.521 -10.558 -0.037 (0)

CdSO4 2.469e-11 2.469e-11 -10.607 -10.607 0.000 (0)

CrOHSO4 1.127e-11 1.127e-11 -10.948 -10.948 0.000 (0)

Zn(SO4)2-2 1.342e-12 9.148e-13 -11.872 -12.039 -0.166 (0)

PbSO4 1.279e-12 1.279e-12 -11.893 -11.893 0.000 (0)

CuSO4 1.048e-12 1.048e-12 -11.980 -11.980 0.000 (0)

Cd(SO4)2-2 1.534e-13 1.046e-13 -12.814 -12.980 -0.166 (0)

CrSO4+ 7.920e-15 7.197e-15 -14.101 -14.143 -0.042 (0)

Pb(SO4)2-2 2.947e-15 2.009e-15 -14.531 -14.697 -0.166 (0)

Cr2(OH)2SO4+2 2.720e-19 1.854e-19 -18.565 -18.732 -0.166 (0)

Cr2(OH)2(SO4)2 2.873e-21 2.873e-21 -20.542 -20.542 0.000 (0)

CrO3SO4-2 2.470e-23 1.684e-23 -22.607 -22.774 -0.166 (0)

Zn 4.862e-08

ZnCO3 3.232e-08 3.232e-08 -7.490 -7.490 0.000 (0)

Zn+2 9.954e-09 7.058e-09 -8.002 -8.151 -0.149 (0)

Zn(OH)2 3.755e-09 3.755e-09 -8.425 -8.425 0.000 (0)

ZnOH+ 1.365e-09 1.241e-09 -8.865 -8.906 -0.042 (0)

ZnHCO3+ 7.246e-10 6.585e-10 -9.140 -9.181 -0.042 (0)

ZnSO4 3.528e-10 3.528e-10 -9.453 -9.453 0.000 (0)

Zn(OH)3- 1.200e-10 1.090e-10 -9.921 -9.962 -0.042 (0)

ZnOHCl 1.389e-11 1.389e-11 -10.857 -10.857 0.000 (0)

ZnNO3+ 7.337e-12 6.667e-12 -11.134 -11.176 -0.042 (0)

ZnCl+ 1.778e-12 1.631e-12 -11.750 -11.787 -0.037 (0)

Zn(SO4)2-2 1.342e-12 9.148e-13 -11.872 -12.039 -0.166 (0)

Zn(OH)4-2 3.688e-14 2.515e-14 -13.433 -13.600 -0.166 (0)

Zn(NO3)2 4.110e-16 4.110e-16 -15.386 -15.386 0.000 (0)

ZnCl2 1.359e-16 1.359e-16 -15.867 -15.867 0.000 (0)

ZnCl3- 1.140e-20 1.046e-20 -19.943 -19.981 -0.037 (0)

ZnCl4-2 6.673e-25 4.764e-25 -24.176 -24.322 -0.146 (0)

------------------------------Saturation indices-------------------------------

Phase SI** log IAP log K(283 K, 1 atm)

Anglesite -6.68 -14.58 -7.90 PbSO4

Anhydrite -2.53 -6.82 -4.29 CaSO4

Antlerite -9.36 -0.57 8.79 Cu3(OH)4SO4

Aragonite 0.85 -7.33 -8.19 CaCO3

Arsenolite -86.03 -89.34 -3.32 As4O6

Artinite -3.74 6.97 10.72 MgCO3:Mg(OH)2:3H2O

As2O5 -40.68 -33.76 6.92 As2O5

Atacamite -7.32 0.94 8.26 Cu2(OH)3Cl

Azurite -6.68 -22.70 -16.02 Cu3(OH)2(CO3)2

Bianchite -9.98 -11.74 -1.76 ZnSO4:6H2O

Brochantite -10.83 6.27 17.10 Cu4(OH)6SO4

Brucite -3.61 14.30 17.90 Mg(OH)2

Ca3(AsO4)2:4H2O -13.21 9.09 22.30 Ca3(AsO4)2:4H2O

CaCrO4 -11.88 -13.90 -2.02 CaCrO4

Calcite 1.07 -7.33 -8.41 CaCO3

Cd(OH)2 -6.32 8.21 14.52 Cd(OH)2

Cd(OH)2(am) -6.33 8.21 14.54 Cd(OH)2

Cd3(OH)2(SO4)2 -24.30 -17.59 6.71 Cd3(OH)2(SO4)2

Cd3(OH)4SO4 -19.04 3.52 22.56 Cd3(OH)4SO4

Cd4(OH)6SO4 -16.68 11.72 28.40 Cd4(OH)6SO4

CdCl2 -16.80 -17.29 -0.49 CdCl2

CdCl2:1H2O -15.66 -17.29 -1.62 CdCl2:1H2O

CdCl2:2.5H2O -15.30 -17.29 -1.98 CdCl2:2.5H2O

Cdmetal(alpha) -31.53 -17.31 14.21 Cd

Cdmetal(gamma) -31.64 -17.31 14.32 Cd

CdOHCl -8.36 -4.54 3.82 CdOHCl

CdSO4 -13.21 -12.90 0.31 CdSO4

CdSO4:1H2O -11.46 -12.90 -1.43 CdSO4:1H2O

CdSO4:2.67H2O -11.19 -12.90 -1.71 CdSO4:2.67H2O

Cerussite -1.74 -15.10 -13.36 PbCO3

CH4(g) -80.27 -123.70 -43.43 CH4

Chalcanthite -11.56 -14.26 -2.70 CuSO4:5H2O

Claudetite -85.76 -89.34 -3.58 As4O6

CO2(g) -3.43 -21.62 -18.18 CO2

Cotunnite -13.95 -18.97 -5.02 PbCl2

Cr(OH)2 -19.15 -8.00 11.15 Cr(OH)2

Cr(OH)3 0.26 1.87 1.61 Cr(OH)3

Cr(OH)3(am) 2.62 1.87 -0.75 Cr(OH)3

Cr2O3 5.62 3.74 -1.89 Cr2O3

CrCl2 -48.62 -33.50 15.12 CrCl2

CrCl3 -52.61 -36.37 16.24 CrCl3

Crmetal -65.60 -33.52 32.08 Cr

CrO3 -25.02 -28.18 -3.16 CrO3

Cu(OH)2 -2.35 6.84 9.20 Cu(OH)2

Cu2(OH)3NO3 -8.46 1.46 9.92 Cu2(OH)3NO3

Cu2SO4 -25.91 -27.68 -1.77 Cu2SO4

Cu3(AsO4)2:2H2O -19.33 -13.23 6.10 Cu3(AsO4)2:2H2O

CuCO3 -3.27 -14.77 -11.50 CuCO3

CuCrO4 -15.90 -21.34 -5.44 CuCrO4

Cumetal -6.63 -16.05 -9.42 Cu

CuOCuSO4 -19.00 -7.41 11.58 CuOCuSO4

Cuprite -6.32 -6.58 -0.26 Cu2O

CuSO4 -17.88 -14.26 3.62 CuSO4

Dolomite(disordered) 1.45 -14.66 -16.11 CaMg(CO3)2

Dolomite(ordered) 2.07 -14.66 -16.72 CaMg(CO3)2

Epsomite -4.57 -6.81 -2.23 MgSO4:7H2O

Goslarite -9.59 -11.74 -2.14 ZnSO4:7H2O

Gypsum -2.20 -6.82 -4.62 CaSO4:2H2O

Halite -8.99 -7.43 1.57 NaCl

Huntite -0.34 -29.30 -28.97 CaMg3(CO3)4

Hydrocerussite -4.91 -23.68 -18.77 Pb3(OH)2(CO3)2

Hydromagnesite -8.26 -15.00 -6.74 Mg5(CO3)4(OH)2:4H2O

K2Cr2O7 -29.42 -47.41 -17.99 K2Cr2O7

K2CrO4 -18.54 -19.22 -0.68 K2CrO4

Langite -12.75 6.27 19.03 Cu4(OH)6SO4:H2O

Larnakite -7.83 -8.06 -0.23 PbO:PbSO4

Laurionite -6.85 -6.23 0.62 PbOHCl

Lime -20.21 14.28 34.50 CaO

Litharge -6.78 6.52 13.30 PbO

Magnesite 0.32 -7.32 -7.65 MgCO3

Malachite -1.92 -7.93 -6.01 Cu2(OH)2CO3

Massicot -6.99 6.52 13.51 PbO

Melanothallite -25.49 -18.65 6.85 CuCl2

Mg(OH)2(active) -4.50 14.30 18.79 Mg(OH)2

MgCr2O4 0.17 18.03 17.87 MgCr2O4

MgCrO4 -20.09 -13.89 6.21 MgCrO4

Minium -32.36 45.08 77.44 Pb3O4

Mirabilite -8.61 -10.46 -1.85 Na2SO4:10H2O

Monteponite -7.86 8.21 16.06 CdO

Na2Cr2O7 -35.63 -45.73 -10.10 Na2Cr2O7

Na2CrO4 -20.66 -17.54 3.11 Na2CrO4

Nantokite -8.91 -16.04 -7.13 CuCl

Natron -9.06 -10.98 -1.92 Na2CO3:10H2O

Nesquehonite -2.88 -7.32 -4.45 MgCO3:3H2O

O2(g) -37.35 51.04 88.39 O2

Otavite -1.42 -13.41 -11.99 CdCO3

Pb(OH)2 -2.17 6.52 8.69 Pb(OH)2

Pb10(OH)6O(CO3)6 -55.75 -64.51 -8.76 Pb10(OH)6O(CO3)6

Pb2(OH)3Cl -8.50 0.29 8.79 Pb2(OH)3Cl

Pb2O(OH)2 -13.15 13.04 26.19 Pb2O(OH)2

Pb2O3 -22.48 38.56 61.04 Pb2O3

Pb2OCO3 -8.40 -8.58 -0.18 Pb2OCO3

Pb3(AsO4)2 -20.00 -14.20 5.80 Pb3(AsO4)2

Pb3O2CO3 -14.10 -2.06 12.05 Pb3O2CO3

Pb3O2SO4 -12.96 -1.54 11.42 Pb3O2SO4

Pb4(OH)6SO4 -16.12 4.98 21.10 Pb4(OH)6SO4

Pb4O3SO4 -18.17 4.98 23.14 Pb4O3SO4

PbCrO4 -8.65 -21.66 -13.01 PbCrO4

Pbmetal -23.24 -19.00 4.24 Pb

PbO:0.3H2O -6.46 6.52 12.98 PbO:0.33H2O

Periclase -8.69 14.30 22.99 MgO

Phosgenite -14.26 -34.07 -19.81 PbCl2:PbCO3

Plattnerite -20.31 32.04 52.35 PbO2

Portlandite -9.71 14.28 24.00 Ca(OH)2

Smithsonite -2.40 -12.25 -9.85 ZnCO3

Tenorite -1.40 6.84 8.25 CuO

Thenardite -10.87 -10.46 0.41 Na2SO4

Thermonatrite -11.71 -10.98 0.73 Na2CO3:H2O

Zincite -2.80 9.37 12.17 ZnO

Zincosite -16.43 -11.73 4.70 ZnSO4

Zn(NO3)2:6H2O -18.17 -15.09 3.09 Zn(NO3)2:6H2O

Zn(OH)2 -2.83 9.37 12.20 Zn(OH)2

Zn(OH)2(am) -3.85 9.37 13.22 Zn(OH)2

Zn(OH)2(beta) -3.16 9.37 12.53 Zn(OH)2

Zn(OH)2(epsilon) -2.92 9.37 12.29 Zn(OH)2

Zn(OH)2(gamma) -2.37 9.37 11.73 Zn(OH)2

Zn2(OH)2SO4 -9.87 -2.37 7.50 Zn2(OH)2SO4

Zn2(OH)3Cl -9.20 5.99 15.19 Zn2(OH)3Cl

Zn3(AsO4)2:2.5H2O -19.30 -5.65 13.65 Zn3(AsO4)2:2.5H2O

Zn3O(SO4)2 -35.41 -14.10 21.31 Zn3O(SO4)2

Zn4(OH)6SO4 -12.03 16.37 28.40 Zn4(OH)6SO4

Zn5(OH)8Cl2 -17.15 21.35 38.50 Zn5(OH)8Cl2

ZnCl2 -23.85 -16.12 7.72 ZnCl2

ZnCO3:1H2O -1.99 -12.25 -10.26 ZnCO3:1H2O

Znmetal -43.36 -16.15 27.21 Zn

ZnO(active) -2.64 9.37 12.01 ZnO

ZnSO4:1H2O -11.51 -11.74 -0.23 ZnSO4:1H2O

**For a gas, SI = log10(fugacity). Fugacity = pressure * phi / 1 atm.

For ideal gases, phi = 1.

Initial solution 19.

----------------------------Distribution of species----------------------------

Log Log Log mole V

Species Molality Activity Molality Activity Gamma cm?mol

OH- 3.163e-07 2.917e-07 -6.500 -6.535 -0.035 (0)

H+ 1.134e-08 1.047e-08 -7.945 -7.980 -0.035 0.00

H2O 5.551e+01 9.999e-01 1.744 -0.000 0.000 18.02

As(3) 1.198e-19

H3AsO3 1.163e-19 1.163e-19 -18.934 -18.934 0.000 (0)

H2AsO3- 3.463e-21 3.172e-21 -20.461 -20.499 -0.038 (0)

HAsO3-2 1.982e-25 1.394e-25 -24.703 -24.856 -0.153 (0)

H4AsO3+ 6.589e-28 6.035e-28 -27.181 -27.219 -0.038 (0)

AsO3-3 6.592e-31 2.988e-31 -30.181 -30.525 -0.344 (0)

As(5) 8.823e-09

HAsO4-2 8.180e-09 5.755e-09 -8.087 -8.240 -0.153 (0)

H2AsO4- 6.398e-10 5.859e-10 -9.194 -9.232 -0.038 (0)

AsO4-3 2.666e-12 1.208e-12 -11.574 -11.918 -0.344 (0)

H3AsO4 9.149e-16 9.161e-16 -15.039 -15.038 0.001 (0)

C(4) 3.937e-03

HCO3- 3.764e-03 3.484e-03 -2.424 -2.458 -0.034 (0)

H2CO3 9.978e-05 9.978e-05 -4.001 -4.001 0.000 (0)

CaHCO3+ 3.380e-05 3.131e-05 -4.471 -4.504 -0.033 (0)

CO3-2 1.570e-05 1.142e-05 -4.804 -4.942 -0.138 (0)

MgHCO3+ 1.068e-05 9.857e-06 -4.972 -5.006 -0.035 (0)

CaCO3 9.514e-06 9.514e-06 -5.022 -5.022 0.000 (0)

MgCO3 2.213e-06 2.213e-06 -5.655 -5.655 0.000 (0)

NaHCO3 1.052e-06 1.052e-06 -5.978 -5.978 0.000 (0)

NaCO3- 1.421e-07 1.315e-07 -6.848 -6.881 -0.034 (0)

CuCO3 3.288e-08 3.288e-08 -7.483 -7.483 0.000 (0)

ZnCO3 2.607e-08 2.607e-08 -7.584 -7.584 0.000 (0)

ZnHCO3+ 3.494e-09 3.200e-09 -8.457 -8.495 -0.038 (0)

CdCO3 3.108e-09 3.108e-09 -8.508 -8.508 0.000 (0)

PbCO3 1.950e-09 1.950e-09 -8.710 -8.710 0.000 (0)

Cu(CO3)2-2 1.436e-09 1.010e-09 -8.843 -8.996 -0.153 (0)

PbHCO3+ 1.176e-10 1.077e-10 -9.930 -9.968 -0.038 (0)

Pb(CO3)2-2 9.127e-11 6.421e-11 -10.040 -10.192 -0.153 (0)

CuHCO3+ 8.592e-11 7.869e-11 -10.066 -10.104 -0.038 (0)

CdHCO3+ 7.571e-11 6.934e-11 -10.121 -10.159 -0.038 (0)

Cd(CO3)2-2 3.739e-11 2.630e-11 -10.427 -10.580 -0.153 (0)

Ca 1.095e-03

Ca+2 1.018e-03 7.401e-04 -2.992 -3.131 -0.138 (0)

CaHCO3+ 3.380e-05 3.131e-05 -4.471 -4.504 -0.033 (0)

CaSO4 3.303e-05 3.303e-05 -4.481 -4.481 0.000 (0)

CaCO3 9.514e-06 9.514e-06 -5.022 -5.022 0.000 (0)

CaNO3+ 6.370e-07 5.833e-07 -6.196 -6.234 -0.038 (0)

CaOH+ 3.895e-09 3.608e-09 -8.410 -8.443 -0.033 (0)

Cd 2.045e-08

Cd+2 1.642e-08 1.194e-08 -7.785 -7.923 -0.138 (0)

CdCO3 3.108e-09 3.108e-09 -8.508 -8.508 0.000 (0)

CdSO4 5.272e-10 5.272e-10 -9.278 -9.278 0.000 (0)

CdCl+ 2.212e-10 2.026e-10 -9.655 -9.693 -0.038 (0)

CdHCO3+ 7.571e-11 6.934e-11 -10.121 -10.159 -0.038 (0)

Cd(CO3)2-2 3.739e-11 2.630e-11 -10.427 -10.580 -0.153 (0)

CdOH+ 3.087e-11 2.828e-11 -10.510 -10.549 -0.038 (0)

CdNO3+ 1.435e-11 1.314e-11 -10.843 -10.881 -0.038 (0)

CdOHCl 5.529e-12 5.529e-12 -11.257 -11.257 0.000 (0)

Cd(SO4)2-2 2.760e-12 1.942e-12 -11.559 -11.712 -0.153 (0)

Cd(OH)2 5.534e-13 5.534e-13 -12.257 -12.257 0.000 (0)

CdCl2 1.468e-13 1.468e-13 -12.833 -12.833 0.000 (0)

Cd(NO3)2 9.336e-16 9.336e-16 -15.030 -15.030 0.000 (0)

Cd(OH)3- 3.550e-17 3.251e-17 -16.450 -16.488 -0.038 (0)

CdCl3- 1.580e-17 1.447e-17 -16.801 -16.840 -0.038 (0)

Cd2OH+3 4.527e-18 2.052e-18 -17.344 -17.688 -0.344 (0)

Cd(OH)4-2 7.273e-24 5.116e-24 -23.138 -23.291 -0.153 (0)

Cl 1.965e-04

Cl- 1.965e-04 1.814e-04 -3.707 -3.741 -0.035 (0)

CdCl+ 2.212e-10 2.026e-10 -9.655 -9.693 -0.038 (0)

ZnOHCl 2.277e-11 2.277e-11 -10.643 -10.643 0.000 (0)

ZnCl+ 1.746e-11 1.611e-11 -10.758 -10.793 -0.035 (0)

CdOHCl 5.529e-12 5.529e-12 -11.257 -11.257 0.000 (0)

CuCl 4.721e-12 4.721e-12 -11.326 -11.326 0.000 (0)

PbCl+ 3.316e-13 3.037e-13 -12.479 -12.518 -0.038 (0)

CuCl2- 2.013e-13 1.858e-13 -12.696 -12.731 -0.035 (0)

CdCl2 1.468e-13 1.468e-13 -12.833 -12.833 0.000 (0)

CuCl+ 1.276e-13 1.178e-13 -12.894 -12.929 -0.035 (0)

ZnCl2 2.359e-15 2.359e-15 -14.627 -14.627 0.000 (0)

CrCl+2 7.699e-16 5.416e-16 -15.114 -15.266 -0.153 (0)

PbCl2 2.294e-16 2.294e-16 -15.639 -15.639 0.000 (0)

CdCl3- 1.580e-17 1.447e-17 -16.801 -16.840 -0.038 (0)

CuCl3-2 9.270e-18 6.782e-18 -17.033 -17.169 -0.136 (0)

CuCl2 3.441e-18 3.441e-18 -17.463 -17.463 0.000 (0)

ZnCl3- 3.453e-19 3.188e-19 -18.462 -18.496 -0.035 (0)

CrOHCl2 1.962e-19 1.962e-19 -18.707 -18.707 0.000 (0)

PbCl3- 2.146e-20 1.965e-20 -19.668 -19.707 -0.038 (0)

CrCl2+ 2.571e-21 2.355e-21 -20.590 -20.628 -0.038 (0)

ZnCl4-2 3.488e-23 2.552e-23 -22.457 -22.593 -0.136 (0)

CuCl3- 4.771e-24 4.404e-24 -23.321 -23.356 -0.035 (0)

PbCl4-2 1.841e-24 1.295e-24 -23.735 -23.888 -0.153 (0)

CrO3Cl- 9.682e-28 8.867e-28 -27.014 -27.052 -0.038 (0)

CuCl4-2 9.286e-30 6.793e-30 -29.032 -29.168 -0.136 (0)

Cr(2) 7.469e-24

Cr+2 7.469e-24 5.254e-24 -23.127 -23.279 -0.153 (0)

Cr(3) 9.616e-07

Cr(OH)2+ 6.771e-07 6.201e-07 -6.169 -6.208 -0.038 (0)

Cr(OH)3 2.240e-07 2.240e-07 -6.650 -6.650 0.000 (0)

Cr(OH)+2 3.982e-08 2.801e-08 -7.400 -7.553 -0.153 (0)

CrO2- 1.109e-08 1.016e-08 -7.955 -7.993 -0.038 (0)

Cr(OH)4- 9.361e-09 8.573e-09 -8.029 -8.067 -0.038 (0)

CrOHSO4 2.852e-10 2.852e-10 -9.545 -9.545 0.000 (0)

Cr+3 8.871e-12 4.021e-12 -11.052 -11.396 -0.344 (0)

CrSO4+ 1.198e-12 1.098e-12 -11.921 -11.960 -0.038 (0)

CrCl+2 7.699e-16 5.416e-16 -15.114 -15.266 -0.153 (0)

Cr2(OH)2SO4+2 1.942e-16 1.366e-16 -15.712 -15.864 -0.153 (0)

CrNO3+2 1.408e-17 9.903e-18 -16.851 -17.004 -0.153 (0)

Cr2(OH)2(SO4)2 1.840e-18 1.840e-18 -17.735 -17.735 0.000 (0)

CrOHCl2 1.962e-19 1.962e-19 -18.707 -18.707 0.000 (0)

CrCl2+ 2.571e-21 2.355e-21 -20.590 -20.628 -0.038 (0)

Cr(6) 3.094e-15

CrO4-2 3.012e-15 2.190e-15 -14.521 -14.660 -0.138 (0)

HCrO4- 7.764e-17 7.110e-17 -16.110 -16.148 -0.038 (0)

NaCrO4- 4.757e-18 4.357e-18 -17.323 -17.361 -0.038 (0)

KCrO4- 3.822e-19 3.500e-19 -18.418 -18.456 -0.038 (0)

H2CrO4 2.737e-25 2.737e-25 -24.563 -24.563 0.000 (0)

CrO3SO4-2 7.629e-26 5.367e-26 -25.118 -25.270 -0.153 (0)

CrO3Cl- 9.682e-28 8.867e-28 -27.014 -27.052 -0.038 (0)

Cr2O7-2 3.740e-31 2.631e-31 -30.427 -30.580 -0.153 (0)

Cu(1) 2.749e-11

Cu+ 2.257e-11 2.067e-11 -10.646 -10.685 -0.038 (0)

CuCl 4.721e-12 4.721e-12 -11.326 -11.326 0.000 (0)

CuCl2- 2.013e-13 1.858e-13 -12.696 -12.731 -0.035 (0)

CuCl3-2 9.270e-18 6.782e-18 -17.033 -17.169 -0.136 (0)

Cu(2) 3.613e-08

CuCO3 3.288e-08 3.288e-08 -7.483 -7.483 0.000 (0)

Cu(CO3)2-2 1.436e-09 1.010e-09 -8.843 -8.996 -0.153 (0)

CuOH+ 7.494e-10 6.918e-10 -9.125 -9.160 -0.035 (0)

Cu+2 6.726e-10 4.891e-10 -9.172 -9.311 -0.138 (0)

Cu(OH)2 2.853e-10 2.853e-10 -9.545 -9.545 0.000 (0)

CuHCO3+ 8.592e-11 7.869e-11 -10.066 -10.104 -0.038 (0)

CuSO4 2.109e-11 2.109e-11 -10.676 -10.676 0.000 (0)

Cu(OH)3- 6.143e-13 5.626e-13 -12.212 -12.250 -0.038 (0)

CuNO3+ 4.094e-13 3.749e-13 -12.388 -12.426 -0.038 (0)

CuCl+ 1.276e-13 1.178e-13 -12.894 -12.929 -0.035 (0)

Cu2(OH)2+2 1.536e-14 1.080e-14 -13.814 -13.966 -0.153 (0)

Cu(NO3)2 9.603e-18 9.603e-18 -17.018 -17.018 0.000 (0)

Cu(OH)4-2 6.052e-18 4.258e-18 -17.218 -17.371 -0.153 (0)

CuCl2 3.441e-18 3.441e-18 -17.463 -17.463 0.000 (0)

CuCl3- 4.771e-24 4.404e-24 -23.321 -23.356 -0.035 (0)

CuCl4-2 9.286e-30 6.793e-30 -29.032 -29.168 -0.136 (0)

H(0) 1.814e-27

H2 9.072e-28 9.085e-28 -27.042 -27.042 0.001 (0)

K 4.665e-05

K+ 4.658e-05 4.302e-05 -4.332 -4.366 -0.035 (0)

KSO4- 6.836e-08 6.326e-08 -7.165 -7.199 -0.034 (0)

KCrO4- 3.822e-19 3.500e-19 -18.418 -18.456 -0.038 (0)

Mg 4.381e-04

Mg+2 4.142e-04 3.012e-04 -3.383 -3.521 -0.138 (0)

MgSO4 1.098e-05 1.098e-05 -4.959 -4.959 0.000 (0)

MgHCO3+ 1.068e-05 9.857e-06 -4.972 -5.006 -0.035 (0)

MgCO3 2.213e-06 2.213e-06 -5.655 -5.655 0.000 (0)

MgOH+ 2.920e-08 2.707e-08 -7.535 -7.568 -0.033 (0)

N(5) 2.411e-04

NO3- 2.405e-04 2.221e-04 -3.619 -3.653 -0.035 (0)

CaNO3+ 6.370e-07 5.833e-07 -6.196 -6.234 -0.038 (0)

ZnNO3+ 2.667e-11 2.442e-11 -10.574 -10.612 -0.038 (0)

CdNO3+ 1.435e-11 1.314e-11 -10.843 -10.881 -0.038 (0)

CuNO3+ 4.094e-13 3.749e-13 -12.388 -12.426 -0.038 (0)

PbNO3+ 1.953e-13 1.788e-13 -12.709 -12.748 -0.038 (0)

Zn(NO3)2 9.810e-16 9.810e-16 -15.008 -15.008 0.000 (0)

Cd(NO3)2 9.336e-16 9.336e-16 -15.030 -15.030 0.000 (0)

Pb(NO3)2 8.105e-17 8.105e-17 -16.091 -16.091 0.000 (0)

CrNO3+2 1.408e-17 9.903e-18 -16.851 -17.004 -0.153 (0)

Cu(NO3)2 9.603e-18 9.603e-18 -17.018 -17.018 0.000 (0)

Na 4.352e-04

Na+ 4.335e-04 4.003e-04 -3.363 -3.398 -0.035 (0)

NaHCO3 1.052e-06 1.052e-06 -5.978 -5.978 0.000 (0)

NaSO4- 5.156e-07 4.772e-07 -6.288 -6.321 -0.034 (0)

NaCO3- 1.421e-07 1.315e-07 -6.848 -6.881 -0.034 (0)

NaCrO4- 4.757e-18 4.357e-18 -17.323 -17.361 -0.038 (0)

O(0) 0.000e+00

O2 0.000e+00 0.000e+00 -42.540 -42.539 0.001 (0)

Pb 2.398e-09

PbCO3 1.950e-09 1.950e-09 -8.710 -8.710 0.000 (0)

PbOH+ 1.498e-10 1.372e-10 -9.824 -9.863 -0.038 (0)

PbHCO3+ 1.176e-10 1.077e-10 -9.930 -9.968 -0.038 (0)

Pb(CO3)2-2 9.127e-11 6.421e-11 -10.040 -10.192 -0.153 (0)

Pb+2 7.814e-11 5.682e-11 -10.107 -10.246 -0.138 (0)

PbSO4 6.310e-12 6.310e-12 -11.200 -11.200 0.000 (0)

Pb(OH)2 4.172e-12 4.172e-12 -11.380 -11.380 0.000 (0)

PbCl+ 3.316e-13 3.037e-13 -12.479 -12.518 -0.038 (0)

PbNO3+ 1.953e-13 1.788e-13 -12.709 -12.748 -0.038 (0)

Pb(SO4)2-2 1.226e-14 8.621e-15 -13.912 -14.064 -0.153 (0)

Pb(OH)3- 4.381e-15 4.012e-15 -14.358 -14.397 -0.038 (0)

PbCl2 2.294e-16 2.294e-16 -15.639 -15.639 0.000 (0)

Pb(NO3)2 8.105e-17 8.105e-17 -16.091 -16.091 0.000 (0)

Pb(OH)4-2 1.343e-18 9.447e-19 -17.872 -18.025 -0.153 (0)

Pb2OH+3 2.727e-19 1.236e-19 -18.564 -18.908 -0.344 (0)

PbCl3- 2.146e-20 1.965e-20 -19.668 -19.707 -0.038 (0)

Pb3(OH)4+2 2.391e-24 1.682e-24 -23.621 -23.774 -0.153 (0)

PbCl4-2 1.841e-24 1.295e-24 -23.735 -23.888 -0.153 (0)

Pb4(OH)4+4 5.519e-30 1.352e-30 -29.258 -29.869 -0.611 (0)

S(6) 3.564e-04

SO4-2 3.118e-04 2.267e-04 -3.506 -3.644 -0.138 (0)

CaSO4 3.303e-05 3.303e-05 -4.481 -4.481 0.000 (0)

MgSO4 1.098e-05 1.098e-05 -4.959 -4.959 0.000 (0)

NaSO4- 5.156e-07 4.772e-07 -6.288 -6.321 -0.034 (0)

KSO4- 6.836e-08 6.326e-08 -7.165 -7.199 -0.034 (0)

ZnSO4 1.724e-09 1.724e-09 -8.763 -8.763 0.000 (0)

CdSO4 5.272e-10 5.272e-10 -9.278 -9.278 0.000 (0)

CrOHSO4 2.852e-10 2.852e-10 -9.545 -9.545 0.000 (0)

HSO4- 1.569e-10 1.450e-10 -9.804 -9.839 -0.034 (0)

CuSO4 2.109e-11 2.109e-11 -10.676 -10.676 0.000 (0)

PbSO4 6.310e-12 6.310e-12 -11.200 -11.200 0.000 (0)

Zn(SO4)2-2 5.527e-12 3.888e-12 -11.258 -11.410 -0.153 (0)

Cd(SO4)2-2 2.760e-12 1.942e-12 -11.559 -11.712 -0.153 (0)

CrSO4+ 1.198e-12 1.098e-12 -11.921 -11.960 -0.038 (0)

Pb(SO4)2-2 1.226e-14 8.621e-15 -13.912 -14.064 -0.153 (0)

Cr2(OH)2SO4+2 1.942e-16 1.366e-16 -15.712 -15.864 -0.153 (0)

Cr2(OH)2(SO4)2 1.840e-18 1.840e-18 -17.735 -17.735 0.000 (0)

CrO3SO4-2 7.629e-26 5.367e-26 -25.118 -25.270 -0.153 (0)

Zn 8.779e-08

Zn+2 5.457e-08 3.969e-08 -7.263 -7.401 -0.138 (0)

ZnCO3 2.607e-08 2.607e-08 -7.584 -7.584 0.000 (0)

ZnHCO3+ 3.494e-09 3.200e-09 -8.457 -8.495 -0.038 (0)

ZnSO4 1.724e-09 1.724e-09 -8.763 -8.763 0.000 (0)

ZnOH+ 1.264e-09 1.158e-09 -8.898 -8.936 -0.038 (0)

Zn(OH)2 5.815e-10 5.815e-10 -9.235 -9.235 0.000 (0)

ZnNO3+ 2.667e-11 2.442e-11 -10.574 -10.612 -0.038 (0)

ZnOHCl 2.277e-11 2.277e-11 -10.643 -10.643 0.000 (0)

ZnCl+ 1.746e-11 1.611e-11 -10.758 -10.793 -0.035 (0)

Zn(SO4)2-2 5.527e-12 3.888e-12 -11.258 -11.410 -0.153 (0)

Zn(OH)3- 3.060e-12 2.802e-12 -11.514 -11.553 -0.038 (0)

ZnCl2 2.359e-15 2.359e-15 -14.627 -14.627 0.000 (0)

Zn(NO3)2 9.810e-16 9.810e-16 -15.008 -15.008 0.000 (0)

Zn(OH)4-2 1.525e-16 1.073e-16 -15.817 -15.970 -0.153 (0)

ZnCl3- 3.453e-19 3.188e-19 -18.462 -18.496 -0.035 (0)

ZnCl4-2 3.488e-23 2.552e-23 -22.457 -22.593 -0.136 (0)

------------------------------Saturation indices-------------------------------

Phase SI** log IAP log K(283 K, 1 atm)

Anglesite -5.99 -13.89 -7.90 PbSO4

Anhydrite -2.48 -6.78 -4.29 CaSO4

Antlerite -8.44 0.34 8.79 Cu3(OH)4SO4

Aragonite 0.12 -8.07 -8.19 CaCO3

Arsenolite -72.42 -75.74 -3.32 As4O6

Artinite -6.74 3.97 10.72 MgCO3:Mg(OH)2:3H2O

As2O5 -36.99 -30.08 6.92 As2O5

Atacamite -6.68 1.58 8.26 Cu2(OH)3Cl

Azurite -5.83 -21.86 -16.02 Cu3(OH)2(CO3)2

Bianchite -9.29 -11.05 -1.76 ZnSO4:6H2O

Brochantite -10.11 6.99 17.10 Cu4(OH)6SO4

Brucite -5.46 12.44 17.90 Mg(OH)2

Ca3(AsO4)2:4H2O -13.89 8.41 22.30 Ca3(AsO4)2:4H2O

CaCrO4 -15.77 -17.79 -2.02 CaCrO4

Calcite 0.33 -8.07 -8.41 CaCO3

Cd(OH)2 -6.49 8.04 14.52 Cd(OH)2

Cd(OH)2(am) -6.50 8.04 14.54 Cd(OH)2

Cd3(OH)2(SO4)2 -21.81 -15.10 6.71 Cd3(OH)2(SO4)2

Cd3(OH)4SO4 -18.05 4.51 22.56 Cd3(OH)4SO4

Cd4(OH)6SO4 -15.86 12.54 28.40 Cd4(OH)6SO4

CdCl2 -14.92 -15.41 -0.49 CdCl2

CdCl2:1H2O -13.78 -15.41 -1.62 CdCl2:1H2O

CdCl2:2.5H2O -13.42 -15.41 -1.98 CdCl2:2.5H2O

Cdmetal(alpha) -30.14 -15.92 14.21 Cd

Cdmetal(gamma) -30.25 -15.92 14.32 Cd

CdOHCl -7.51 -3.68 3.82 CdOHCl

CdSO4 -11.88 -11.57 0.31 CdSO4

CdSO4:1H2O -10.13 -11.57 -1.43 CdSO4:1H2O

CdSO4:2.67H2O -9.86 -11.57 -1.71 CdSO4:2.67H2O

Cerussite -1.83 -15.19 -13.36 PbCO3

CH4(g) -73.31 -116.74 -43.43 CH4

Chalcanthite -10.26 -12.96 -2.70 CuSO4:5H2O

Claudetite -72.16 -75.74 -3.58 As4O6

CO2(g) -2.72 -20.90 -18.18 CO2

Cotunnite -12.71 -17.73 -5.02 PbCl2

Cr(OH)2 -18.47 -7.32 11.15 Cr(OH)2

Cr(OH)3 0.16 1.77 1.61 Cr(OH)3

Cr(OH)3(am) 2.52 1.77 -0.75 Cr(OH)3

Cr2O3 5.43 3.54 -1.89 Cr2O3

CrCl2 -45.88 -30.76 15.12 CrCl2

CrCl3 -49.63 -33.39 16.24 CrCl3

Crmetal -63.36 -31.28 32.08 Cr

CrO3 -27.46 -30.62 -3.16 CrO3

Cu(OH)2 -2.55 6.65 9.20 Cu(OH)2

Cu2(OH)3NO3 -8.26 1.67 9.92 Cu2(OH)3NO3

Cu2SO4 -23.24 -25.01 -1.77 Cu2SO4

Cu3(AsO4)2:2H2O -16.23 -10.13 6.10 Cu3(AsO4)2:2H2O

CuCO3 -2.75 -14.25 -11.50 CuCO3

CuCrO4 -18.53 -23.97 -5.44 CuCrO4

Cumetal -5.26 -14.68 -9.42 Cu

CuOCuSO4 -17.89 -6.31 11.58 CuOCuSO4

Cuprite -5.15 -5.41 -0.26 Cu2O

CuSO4 -16.57 -12.96 3.62 CuSO4

Dolomite(disordered) -0.43 -16.54 -16.11 CaMg(CO3)2

Dolomite(ordered) 0.19 -16.54 -16.72 CaMg(CO3)2

Epsomite -4.93 -7.17 -2.23 MgSO4:7H2O

Goslarite -8.90 -11.05 -2.14 ZnSO4:7H2O

Gypsum -2.16 -6.78 -4.62 CaSO4:2H2O

Halite -8.71 -7.14 1.57 NaCl

Huntite -4.50 -33.46 -28.97 CaMg3(CO3)4

Hydrocerussite -5.89 -24.66 -18.77 Pb3(OH)2(CO3)2

Hydromagnesite -14.68 -21.42 -6.74 Mg5(CO3)4(OH)2:4H2O

K2Cr2O7 -36.02 -54.01 -17.99 K2Cr2O7

K2CrO4 -22.71 -23.39 -0.68 K2CrO4

Langite -12.03 6.99 19.03 Cu4(OH)6SO4:H2O

Larnakite -7.94 -8.18 -0.23 PbO:PbSO4

Laurionite -6.63 -6.01 0.62 PbOHCl

Lime -21.67 12.83 34.50 CaO

Litharge -7.59 5.71 13.30 PbO

Magnesite -0.82 -8.46 -7.65 MgCO3

Malachite -1.59 -7.60 -6.01 Cu2(OH)2CO3

Massicot -7.80 5.71 13.51 PbO

Melanothallite -23.64 -16.79 6.85 CuCl2

Mg(OH)2(active) -6.36 12.44 18.79 Mg(OH)2

MgCr2O4 -1.88 15.98 17.87 MgCr2O4

MgCrO4 -24.39 -18.18 6.21 MgCrO4

Minium -36.33 41.10 77.44 Pb3O4

Mirabilite -8.59 -10.44 -1.85 Na2SO4:10H2O

Monteponite -8.03 8.04 16.06 CdO

Na2Cr2O7 -41.97 -52.07 -10.10 Na2Cr2O7

Na2CrO4 -24.57 -21.45 3.11 Na2CrO4

Nantokite -7.30 -14.43 -7.13 CuCl

Natron -9.82 -11.74 -1.92 Na2CO3:10H2O

Nesquehonite -4.02 -8.46 -4.45 MgCO3:3H2O

O2(g) -40.47 47.92 88.39 O2

Otavite -0.87 -12.87 -11.99 CdCO3

Pb(OH)2 -2.98 5.71 8.69 Pb(OH)2

Pb10(OH)6O(CO3)6 -59.51 -68.27 -8.76 Pb10(OH)6O(CO3)6

Pb2(OH)3Cl -9.09 -0.29 8.79 Pb2(OH)3Cl

Pb2O(OH)2 -14.76 11.43 26.19 Pb2O(OH)2

Pb2O3 -25.65 35.39 61.04 Pb2O3

Pb2OCO3 -9.29 -9.47 -0.18 Pb2OCO3

Pb3(AsO4)2 -18.73 -12.93 5.80 Pb3(AsO4)2

Pb3O2CO3 -15.81 -3.76 12.05 Pb3O2CO3

Pb3O2SO4 -13.88 -2.46 11.42 Pb3O2SO4

Pb4(OH)6SO4 -17.85 3.25 21.10 Pb4(OH)6SO4

Pb4O3SO4 -19.89 3.25 23.14 Pb4O3SO4

PbCrO4 -11.90 -24.91 -13.01 PbCrO4

Pbmetal -22.48 -18.25 4.24 Pb

PbO:0.3H2O -7.27 5.71 12.98 PbO:0.33H2O

Periclase -10.55 12.44 22.99 MgO

Phosgenite -13.11 -32.92 -19.81 PbCl2:PbCO3

Plattnerite -22.68 29.67 52.35 PbO2

Portlandite -11.17 12.83 24.00 Ca(OH)2

Smithsonite -2.49 -12.34 -9.85 ZnCO3

Tenorite -1.60 6.65 8.25 CuO

Thenardite -10.85 -10.44 0.41 Na2SO4

Thermonatrite -12.47 -11.74 0.73 Na2CO3:H2O

Zincite -3.61 8.56 12.17 ZnO

Zincosite -15.74 -11.05 4.70 ZnSO4

Zn(NO3)2:6H2O -17.80 -14.71 3.09 Zn(NO3)2:6H2O

Zn(OH)2 -3.64 8.56 12.20 Zn(OH)2

Zn(OH)2(am) -4.66 8.56 13.22 Zn(OH)2

Zn(OH)2(beta) -3.97 8.56 12.53 Zn(OH)2

Zn(OH)2(epsilon) -3.73 8.56 12.29 Zn(OH)2

Zn(OH)2(gamma) -3.18 8.56 11.73 Zn(OH)2

Zn2(OH)2SO4 -9.99 -2.49 7.50 Zn2(OH)2SO4

Zn2(OH)3Cl -9.80 5.40 15.19 Zn2(OH)3Cl

Zn3(AsO4)2:2.5H2O -18.05 -4.40 13.65 Zn3(AsO4)2:2.5H2O

Zn3O(SO4)2 -34.84 -13.53 21.31 Zn3O(SO4)2

Zn4(OH)6SO4 -13.77 14.63 28.40 Zn4(OH)6SO4

Zn5(OH)8Cl2 -19.15 19.35 38.50 Zn5(OH)8Cl2

ZnCl2 -22.61 -14.88 7.72 ZnCl2

ZnCO3:1H2O -2.08 -12.34 -10.26 ZnCO3:1H2O

Znmetal -42.61 -15.40 27.21 Zn

ZnO(active) -3.45 8.56 12.01 ZnO

ZnSO4:1H2O -10.82 -11.05 -0.23 ZnSO4:1H2O

**For a gas, SI = log10(fugacity). Fugacity = pressure * phi / 1 atm.

For ideal gases, phi = 1.

Initial solution 20.

----------------------------Distribution of species----------------------------

Log Log Log mole V

Species Molality Activity Molality Activity Gamma cm?mol

OH- 2.901e-07 2.661e-07 -6.537 -6.575 -0.038 (0)

H+ 1.250e-08 1.148e-08 -7.903 -7.940 -0.037 0.00

H2O 5.551e+01 9.999e-01 1.744 -0.000 0.000 18.02

As(3) 8.275e-20

H3AsO3 8.055e-20 8.055e-20 -19.094 -19.094 0.000 (0)

H2AsO3- 2.202e-21 2.003e-21 -20.657 -20.698 -0.041 (0)

HAsO3-2 1.173e-25 8.030e-26 -24.931 -25.095 -0.165 (0)

H4AsO3+ 5.038e-28 4.582e-28 -27.298 -27.339 -0.041 (0)

AsO3-3 3.685e-31 1.569e-31 -30.434 -30.804 -0.371 (0)

As(5) 4.368e-09

HAsO4-2 4.029e-09 2.757e-09 -8.395 -8.560 -0.165 (0)

H2AsO4- 3.384e-10 3.078e-10 -9.471 -9.512 -0.041 (0)

AsO4-3 1.240e-12 5.280e-13 -11.907 -12.277 -0.371 (0)

H3AsO4 5.268e-16 5.277e-16 -15.278 -15.278 0.001 (0)

C(4) 5.093e-03

HCO3- 4.864e-03 4.477e-03 -2.313 -2.349 -0.036 (0)

H2CO3 1.406e-04 1.406e-04 -3.852 -3.852 0.000 (0)

CaHCO3+ 3.803e-05 3.505e-05 -4.420 -4.455 -0.035 (0)

CO3-2 1.882e-05 1.338e-05 -4.725 -4.873 -0.148 (0)

MgHCO3+ 1.662e-05 1.525e-05 -4.779 -4.817 -0.037 (0)

CaCO3 9.712e-06 9.712e-06 -5.013 -5.013 0.000 (0)

MgCO3 3.123e-06 3.123e-06 -5.505 -5.505 0.000 (0)

NaHCO3 1.974e-06 1.974e-06 -5.705 -5.705 0.000 (0)

NaCO3- 2.444e-07 2.250e-07 -6.612 -6.648 -0.036 (0)

CuCO3 4.568e-08 4.568e-08 -7.340 -7.340 0.000 (0)

ZnCO3 8.835e-09 8.835e-09 -8.054 -8.054 0.000 (0)

CdCO3 5.860e-09 5.860e-09 -8.232 -8.232 0.000 (0)

Cu(CO3)2-2 2.404e-09 1.645e-09 -8.619 -8.784 -0.165 (0)

ZnHCO3+ 1.307e-09 1.189e-09 -8.884 -8.925 -0.041 (0)

PbCO3 1.799e-10 1.799e-10 -9.745 -9.745 0.000 (0)

CdHCO3+ 1.576e-10 1.433e-10 -9.802 -9.844 -0.041 (0)

CuHCO3+ 1.318e-10 1.199e-10 -9.880 -9.921 -0.041 (0)

Cd(CO3)2-2 8.494e-11 5.813e-11 -10.071 -10.236 -0.165 (0)

PbHCO3+ 1.198e-11 1.089e-11 -10.922 -10.963 -0.041 (0)

Pb(CO3)2-2 1.015e-11 6.944e-12 -10.994 -11.158 -0.165 (0)

Ca 9.929e-04

Ca+2 9.065e-04 6.446e-04 -3.043 -3.191 -0.148 (0)

CaHCO3+ 3.803e-05 3.505e-05 -4.420 -4.455 -0.035 (0)

CaSO4 3.792e-05 3.792e-05 -4.421 -4.421 0.000 (0)

CaCO3 9.712e-06 9.712e-06 -5.013 -5.013 0.000 (0)

CaNO3+ 6.727e-07 6.118e-07 -6.172 -6.213 -0.041 (0)

CaOH+ 3.110e-09 2.866e-09 -8.507 -8.543 -0.035 (0)

Cd 3.499e-08

Cd+2 2.702e-08 1.921e-08 -7.568 -7.716 -0.148 (0)

CdCO3 5.860e-09 5.860e-09 -8.232 -8.232 0.000 (0)

CdSO4 1.118e-09 1.118e-09 -8.952 -8.952 0.000 (0)

CdCl+ 6.533e-10 5.942e-10 -9.185 -9.226 -0.041 (0)

CdHCO3+ 1.576e-10 1.433e-10 -9.802 -9.844 -0.041 (0)

Cd(CO3)2-2 8.494e-11 5.813e-11 -10.071 -10.236 -0.165 (0)

CdOH+ 4.561e-11 4.148e-11 -10.341 -10.382 -0.041 (0)

CdNO3+ 2.798e-11 2.545e-11 -10.553 -10.594 -0.041 (0)

CdOHCl 1.479e-11 1.479e-11 -10.830 -10.830 0.000 (0)

Cd(SO4)2-2 7.930e-12 5.427e-12 -11.101 -11.265 -0.165 (0)

CdCl2 7.852e-13 7.852e-13 -12.105 -12.105 0.000 (0)

Cd(OH)2 7.404e-13 7.404e-13 -12.131 -12.131 0.000 (0)

Cd(NO3)2 2.178e-15 2.178e-15 -14.662 -14.662 0.000 (0)

CdCl3- 1.552e-16 1.411e-16 -15.809 -15.850 -0.041 (0)

Cd(OH)3- 4.361e-17 3.966e-17 -16.360 -16.402 -0.041 (0)

Cd2OH+3 1.137e-17 4.841e-18 -16.944 -17.315 -0.371 (0)

Cd(OH)4-2 8.319e-24 5.693e-24 -23.080 -23.245 -0.165 (0)

Cl 3.603e-04

Cl- 3.603e-04 3.308e-04 -3.443 -3.480 -0.037 (0)

CdCl+ 6.533e-10 5.942e-10 -9.185 -9.226 -0.041 (0)

CdOHCl 1.479e-11 1.479e-11 -10.830 -10.830 0.000 (0)

ZnOHCl 1.095e-11 1.095e-11 -10.961 -10.961 0.000 (0)

CuCl 1.020e-11 1.020e-11 -10.991 -10.991 0.000 (0)

ZnCl+ 9.255e-12 8.495e-12 -11.034 -11.071 -0.037 (0)

CuCl2- 7.978e-13 7.324e-13 -12.098 -12.135 -0.037 (0)

CdCl2 7.852e-13 7.852e-13 -12.105 -12.105 0.000 (0)

CuCl+ 2.773e-13 2.546e-13 -12.557 -12.594 -0.037 (0)

PbCl+ 4.794e-14 4.360e-14 -13.319 -13.361 -0.041 (0)

ZnCl2 2.267e-15 2.267e-15 -14.644 -14.644 0.000 (0)

CrCl+2 2.111e-15 1.445e-15 -14.675 -14.840 -0.165 (0)

CdCl3- 1.552e-16 1.411e-16 -15.809 -15.850 -0.041 (0)

CuCl3-2 6.810e-17 4.875e-17 -16.167 -16.312 -0.145 (0)

PbCl2 6.004e-17 6.004e-17 -16.222 -16.222 0.000 (0)

CuCl2 1.357e-17 1.357e-17 -16.868 -16.868 0.000 (0)

CrOHCl2 8.702e-19 8.702e-19 -18.060 -18.060 0.000 (0)

ZnCl3- 6.088e-19 5.589e-19 -18.215 -18.253 -0.037 (0)

CrCl2+ 1.259e-20 1.145e-20 -19.900 -19.941 -0.041 (0)

PbCl3- 1.032e-20 9.383e-21 -19.986 -20.028 -0.041 (0)

ZnCl4-2 1.140e-22 8.158e-23 -21.943 -22.088 -0.145 (0)

CuCl3- 3.449e-23 3.166e-23 -22.462 -22.499 -0.037 (0)

PbCl4-2 1.647e-24 1.127e-24 -23.783 -23.948 -0.165 (0)

CrO3Cl- 1.496e-27 1.361e-27 -26.825 -26.866 -0.041 (0)

CuCl4-2 1.244e-28 8.905e-29 -27.905 -28.050 -0.145 (0)

Cr(2) 1.123e-23

Cr+2 1.123e-23 7.686e-24 -22.950 -23.114 -0.165 (0)

Cr(3) 1.154e-06

Cr(OH)2+ 8.295e-07 7.544e-07 -6.081 -6.122 -0.041 (0)

Cr(OH)3 2.485e-07 2.485e-07 -6.605 -6.605 0.000 (0)

Cr(OH)+2 5.461e-08 3.737e-08 -7.263 -7.427 -0.165 (0)

CrO2- 1.130e-08 1.028e-08 -7.947 -7.988 -0.041 (0)

Cr(OH)4- 9.538e-09 8.675e-09 -8.021 -8.062 -0.041 (0)

CrOHSO4 5.015e-10 5.015e-10 -9.300 -9.300 0.000 (0)

Cr+3 1.381e-11 5.881e-12 -10.860 -11.231 -0.371 (0)

CrSO4+ 2.327e-12 2.116e-12 -11.633 -11.674 -0.041 (0)

CrCl+2 2.111e-15 1.445e-15 -14.675 -14.840 -0.165 (0)

Cr2(OH)2SO4+2 4.684e-16 3.205e-16 -15.329 -15.494 -0.165 (0)

CrNO3+2 2.549e-17 1.744e-17 -16.594 -16.758 -0.165 (0)

Cr2(OH)2(SO4)2 5.690e-18 5.690e-18 -17.245 -17.245 0.000 (0)

CrOHCl2 8.702e-19 8.702e-19 -18.060 -18.060 0.000 (0)

CrCl2+ 1.259e-20 1.145e-20 -19.900 -19.941 -0.041 (0)

Cr(6) 2.221e-15

CrO4-2 2.156e-15 1.533e-15 -14.666 -14.814 -0.148 (0)

HCrO4- 6.001e-17 5.458e-17 -16.222 -16.263 -0.041 (0)

NaCrO4- 4.897e-18 4.454e-18 -17.310 -17.351 -0.041 (0)

KCrO4- 3.157e-19 2.871e-19 -18.501 -18.542 -0.041 (0)

H2CrO4 2.304e-25 2.304e-25 -24.638 -24.638 0.000 (0)

CrO3SO4-2 8.701e-26 5.954e-26 -25.060 -25.225 -0.165 (0)

CrO3Cl- 1.496e-27 1.361e-27 -26.825 -26.866 -0.041 (0)

Cr2O7-2 2.266e-31 1.550e-31 -30.645 -30.810 -0.165 (0)

Cu(1) 3.794e-11

Cu+ 2.694e-11 2.450e-11 -10.570 -10.611 -0.041 (0)

CuCl 1.020e-11 1.020e-11 -10.991 -10.991 0.000 (0)

CuCl2- 7.978e-13 7.324e-13 -12.098 -12.135 -0.037 (0)

CuCl3-2 6.810e-17 4.875e-17 -16.167 -16.312 -0.145 (0)

Cu(2) 5.016e-08

CuCO3 4.568e-08 4.568e-08 -7.340 -7.340 0.000 (0)

Cu(CO3)2-2 2.404e-09 1.645e-09 -8.619 -8.784 -0.165 (0)

Cu+2 8.153e-10 5.797e-10 -9.089 -9.237 -0.148 (0)

CuOH+ 8.147e-10 7.479e-10 -9.089 -9.126 -0.037 (0)

Cu(OH)2 2.813e-10 2.813e-10 -9.551 -9.551 0.000 (0)

CuHCO3+ 1.318e-10 1.199e-10 -9.880 -9.921 -0.041 (0)

CuSO4 3.296e-11 3.296e-11 -10.482 -10.482 0.000 (0)

CuNO3+ 5.884e-13 5.352e-13 -12.230 -12.272 -0.041 (0)

Cu(OH)3- 5.562e-13 5.059e-13 -12.255 -12.296 -0.041 (0)

CuCl+ 2.773e-13 2.546e-13 -12.557 -12.594 -0.037 (0)

Cu2(OH)2+2 1.845e-14 1.263e-14 -13.734 -13.899 -0.165 (0)

Cu(NO3)2 1.651e-17 1.651e-17 -16.782 -16.782 0.000 (0)

CuCl2 1.357e-17 1.357e-17 -16.868 -16.868 0.000 (0)

Cu(OH)4-2 5.102e-18 3.491e-18 -17.292 -17.457 -0.165 (0)

CuCl3- 3.449e-23 3.166e-23 -22.462 -22.499 -0.037 (0)

CuCl4-2 1.244e-28 8.905e-29 -27.905 -28.050 -0.145 (0)

H(0) 2.181e-27

H2 1.090e-27 1.092e-27 -26.962 -26.962 0.001 (0)

K 5.499e-05

K+ 5.489e-05 5.040e-05 -4.261 -4.298 -0.037 (0)

KSO4- 1.061e-07 9.770e-08 -6.974 -7.010 -0.036 (0)

KCrO4- 3.157e-19 2.871e-19 -18.501 -18.542 -0.041 (0)

Mg 5.471e-04

Mg+2 5.099e-04 3.626e-04 -3.292 -3.441 -0.148 (0)

MgSO4 1.742e-05 1.742e-05 -4.759 -4.759 0.000 (0)

MgHCO3+ 1.662e-05 1.525e-05 -4.779 -4.817 -0.037 (0)

MgCO3 3.123e-06 3.123e-06 -5.505 -5.505 0.000 (0)

MgOH+ 3.222e-08 2.972e-08 -7.492 -7.527 -0.035 (0)

N(5) 2.919e-04

NO3- 2.912e-04 2.674e-04 -3.536 -3.573 -0.037 (0)

CaNO3+ 6.727e-07 6.118e-07 -6.172 -6.213 -0.041 (0)

CdNO3+ 2.798e-11 2.545e-11 -10.553 -10.594 -0.041 (0)

ZnNO3+ 9.349e-12 8.503e-12 -11.029 -11.070 -0.041 (0)

CuNO3+ 5.884e-13 5.352e-13 -12.230 -12.272 -0.041 (0)

PbNO3+ 1.864e-14 1.695e-14 -13.730 -13.771 -0.041 (0)

Cd(NO3)2 2.178e-15 2.178e-15 -14.662 -14.662 0.000 (0)

Zn(NO3)2 4.112e-16 4.112e-16 -15.386 -15.386 0.000 (0)

CrNO3+2 2.549e-17 1.744e-17 -16.594 -16.758 -0.165 (0)

Cu(NO3)2 1.651e-17 1.651e-17 -16.782 -16.782 0.000 (0)

Pb(NO3)2 9.253e-18 9.253e-18 -17.034 -17.034 0.000 (0)

Na 6.398e-04

Na+ 6.366e-04 5.846e-04 -3.196 -3.233 -0.037 (0)

NaHCO3 1.974e-06 1.974e-06 -5.705 -5.705 0.000 (0)

NaSO4- 9.977e-07 9.184e-07 -6.001 -6.037 -0.036 (0)

NaCO3- 2.444e-07 2.250e-07 -6.612 -6.648 -0.036 (0)

NaCrO4- 4.897e-18 4.454e-18 -17.310 -17.351 -0.041 (0)

O(0) 0.000e+00

O2 0.000e+00 0.000e+00 -42.700 -42.699 0.001 (0)

Pb 2.202e-10

PbCO3 1.799e-10 1.799e-10 -9.745 -9.745 0.000 (0)

PbHCO3+ 1.198e-11 1.089e-11 -10.922 -10.963 -0.041 (0)

PbOH+ 1.083e-11 9.852e-12 -10.965 -11.006 -0.041 (0)

Pb(CO3)2-2 1.015e-11 6.944e-12 -10.994 -11.158 -0.165 (0)

Pb+2 6.290e-12 4.473e-12 -11.201 -11.349 -0.148 (0)

PbSO4 6.548e-13 6.548e-13 -12.184 -12.184 0.000 (0)

Pb(OH)2 2.732e-13 2.732e-13 -12.564 -12.564 0.000 (0)

PbCl+ 4.794e-14 4.360e-14 -13.319 -13.361 -0.041 (0)

PbNO3+ 1.864e-14 1.695e-14 -13.730 -13.771 -0.041 (0)

Pb(SO4)2-2 1.723e-15 1.179e-15 -14.764 -14.928 -0.165 (0)

Pb(OH)3- 2.634e-16 2.396e-16 -15.579 -15.621 -0.041 (0)

PbCl2 6.004e-17 6.004e-17 -16.222 -16.222 0.000 (0)

Pb(NO3)2 9.253e-18 9.253e-18 -17.034 -17.034 0.000 (0)

Pb(OH)4-2 7.518e-20 5.145e-20 -19.124 -19.289 -0.165 (0)

PbCl3- 1.032e-20 9.383e-21 -19.986 -20.028 -0.041 (0)

Pb2OH+3 1.640e-21 6.984e-22 -20.785 -21.156 -0.371 (0)

PbCl4-2 1.647e-24 1.127e-24 -23.783 -23.948 -0.165 (0)

Pb3(OH)4+2 8.294e-28 5.676e-28 -27.081 -27.246 -0.165 (0)

Pb4(OH)4+4 1.638e-34 3.591e-35 -33.786 -34.445 -0.659 (0)

S(6) 4.768e-04

SO4-2 4.203e-04 2.989e-04 -3.376 -3.525 -0.148 (0)

CaSO4 3.792e-05 3.792e-05 -4.421 -4.421 0.000 (0)

MgSO4 1.742e-05 1.742e-05 -4.759 -4.759 0.000 (0)

NaSO4- 9.977e-07 9.184e-07 -6.001 -6.037 -0.036 (0)

KSO4- 1.061e-07 9.770e-08 -6.974 -7.010 -0.036 (0)

CdSO4 1.118e-09 1.118e-09 -8.952 -8.952 0.000 (0)

ZnSO4 6.571e-10 6.571e-10 -9.182 -9.182 0.000 (0)

CrOHSO4 5.015e-10 5.015e-10 -9.300 -9.300 0.000 (0)

HSO4- 2.281e-10 2.096e-10 -9.642 -9.679 -0.037 (0)

CuSO4 3.296e-11 3.296e-11 -10.482 -10.482 0.000 (0)

Cd(SO4)2-2 7.930e-12 5.427e-12 -11.101 -11.265 -0.165 (0)

Zn(SO4)2-2 2.854e-12 1.953e-12 -11.545 -11.709 -0.165 (0)

CrSO4+ 2.327e-12 2.116e-12 -11.633 -11.674 -0.041 (0)

PbSO4 6.548e-13 6.548e-13 -12.184 -12.184 0.000 (0)

Pb(SO4)2-2 1.723e-15 1.179e-15 -14.764 -14.928 -0.165 (0)

Cr2(OH)2SO4+2 4.684e-16 3.205e-16 -15.329 -15.494 -0.165 (0)

Cr2(OH)2(SO4)2 5.690e-18 5.690e-18 -17.245 -17.245 0.000 (0)

CrO3SO4-2 8.701e-26 5.954e-26 -25.060 -25.225 -0.165 (0)

Zn 2.744e-08

Zn+2 1.613e-08 1.147e-08 -7.792 -7.940 -0.148 (0)

ZnCO3 8.835e-09 8.835e-09 -8.054 -8.054 0.000 (0)

ZnHCO3+ 1.307e-09 1.189e-09 -8.884 -8.925 -0.041 (0)

ZnSO4 6.571e-10 6.571e-10 -9.182 -9.182 0.000 (0)

ZnOH+ 3.356e-10 3.053e-10 -9.474 -9.515 -0.041 (0)

Zn(OH)2 1.398e-10 1.398e-10 -9.854 -9.854 0.000 (0)

ZnOHCl 1.095e-11 1.095e-11 -10.961 -10.961 0.000 (0)

ZnNO3+ 9.349e-12 8.503e-12 -11.029 -11.070 -0.041 (0)

ZnCl+ 9.255e-12 8.495e-12 -11.034 -11.071 -0.037 (0)

Zn(SO4)2-2 2.854e-12 1.953e-12 -11.545 -11.709 -0.165 (0)

Zn(OH)3- 6.756e-13 6.145e-13 -12.170 -12.212 -0.041 (0)

ZnCl2 2.267e-15 2.267e-15 -14.644 -14.644 0.000 (0)

Zn(NO3)2 4.112e-16 4.112e-16 -15.386 -15.386 0.000 (0)

Zn(OH)4-2 3.135e-17 2.145e-17 -16.504 -16.669 -0.165 (0)

ZnCl3- 6.088e-19 5.589e-19 -18.215 -18.253 -0.037 (0)

ZnCl4-2 1.140e-22 8.158e-23 -21.943 -22.088 -0.145 (0)

------------------------------Saturation indices-------------------------------

Phase SI** log IAP log K(283 K, 1 atm)

Anglesite -6.97 -14.87 -7.90 PbSO4

Anhydrite -2.42 -6.72 -4.29 CaSO4

Antlerite -8.26 0.52 8.79 Cu3(OH)4SO4

Aragonite 0.12 -8.06 -8.19 CaCO3

Arsenolite -73.06 -76.38 -3.32 As4O6

Artinite -6.59 4.13 10.72 MgCO3:Mg(OH)2:3H2O

As2O5 -37.47 -30.56 6.92 As2O5

Atacamite -6.39 1.87 8.26 Cu2(OH)3Cl

Azurite -5.56 -21.58 -16.02 Cu3(OH)2(CO3)2

Bianchite -9.71 -11.47 -1.76 ZnSO4:6H2O

Brochantite -9.94 7.17 17.10 Cu4(OH)6SO4

Brucite -5.46 12.44 17.90 Mg(OH)2

Ca3(AsO4)2:4H2O -14.79 7.51 22.30 Ca3(AsO4)2:4H2O

CaCrO4 -15.99 -18.01 -2.02 CaCrO4

Calcite 0.34 -8.06 -8.41 CaCO3

Cd(OH)2 -6.36 8.16 14.52 Cd(OH)2

Cd(OH)2(am) -6.37 8.16 14.54 Cd(OH)2

Cd3(OH)2(SO4)2 -21.03 -14.32 6.71 Cd3(OH)2(SO4)2

Cd3(OH)4SO4 -17.47 5.09 22.56 Cd3(OH)4SO4

Cd4(OH)6SO4 -15.15 13.25 28.40 Cd4(OH)6SO4

CdCl2 -14.19 -14.68 -0.49 CdCl2

CdCl2:1H2O -13.05 -14.68 -1.62 CdCl2:1H2O

CdCl2:2.5H2O -12.70 -14.68 -1.98 CdCl2:2.5H2O

Cdmetal(alpha) -29.93 -15.72 14.21 Cd

Cdmetal(gamma) -30.04 -15.72 14.32 Cd

CdOHCl -7.08 -3.26 3.82 CdOHCl

CdSO4 -11.55 -11.24 0.31 CdSO4

CdSO4:1H2O -9.81 -11.24 -1.43 CdSO4:1H2O

CdSO4:2.67H2O -9.54 -11.24 -1.71 CdSO4:2.67H2O

Cerussite -2.86 -16.22 -13.36 PbCO3

CH4(g) -72.84 -116.27 -43.43 CH4

Chalcanthite -10.07 -12.76 -2.70 CuSO4:5H2O

Claudetite -72.79 -76.38 -3.58 As4O6

CO2(g) -2.57 -20.75 -18.18 CO2

Cotunnite -13.29 -18.31 -5.02 PbCl2

Cr(OH)2 -18.38 -7.23 11.15 Cr(OH)2

Cr(OH)3 0.21 1.82 1.61 Cr(OH)3

Cr(OH)3(am) 2.57 1.82 -0.75 Cr(OH)3

Cr2O3 5.52 3.64 -1.89 Cr2O3

CrCl2 -45.19 -30.08 15.12 CrCl2

CrCl3 -48.68 -32.44 16.24 CrCl3

Crmetal -63.19 -31.11 32.08 Cr

CrO3 -27.53 -30.69 -3.16 CrO3

Cu(OH)2 -2.55 6.64 9.20 Cu(OH)2

Cu2(OH)3NO3 -8.15 1.77 9.92 Cu2(OH)3NO3

Cu2SO4 -22.97 -24.75 -1.77 Cu2SO4

Cu3(AsO4)2:2H2O -16.73 -10.63 6.10 Cu3(AsO4)2:2H2O

CuCO3 -2.61 -14.11 -11.50 CuCO3

CuCrO4 -18.61 -24.05 -5.44 CuCrO4

Cumetal -5.19 -14.61 -9.42 Cu

CuOCuSO4 -17.70 -6.12 11.58 CuOCuSO4

Cuprite -5.09 -5.34 -0.26 Cu2O

CuSO4 -16.38 -12.76 3.62 CuSO4

Dolomite(disordered) -0.27 -16.38 -16.11 CaMg(CO3)2

Dolomite(ordered) 0.35 -16.38 -16.72 CaMg(CO3)2

Epsomite -4.73 -6.97 -2.23 MgSO4:7H2O

Goslarite -9.32 -11.47 -2.14 ZnSO4:7H2O

Gypsum -2.10 -6.72 -4.62 CaSO4:2H2O

Halite -8.28 -6.71 1.57 NaCl

Huntite -4.04 -33.01 -28.97 CaMg3(CO3)4

Hydrocerussite -9.14 -27.92 -18.77 Pb3(OH)2(CO3)2

Hydromagnesite -14.08 -20.82 -6.74 Mg5(CO3)4(OH)2:4H2O

K2Cr2O7 -36.11 -54.10 -17.99 K2Cr2O7

K2CrO4 -22.73 -23.41 -0.68 K2CrO4

Langite -11.86 7.17 19.03 Cu4(OH)6SO4:H2O

Larnakite -10.11 -10.34 -0.23 PbO:PbSO4

Laurionite -7.51 -6.89 0.62 PbOHCl

Lime -21.81 12.69 34.50 CaO

Litharge -8.77 4.53 13.30 PbO

Magnesite -0.67 -8.31 -7.65 MgCO3

Malachite -1.45 -7.47 -6.01 Cu2(OH)2CO3

Massicot -8.98 4.53 13.51 PbO

Melanothallite -23.04 -16.20 6.85 CuCl2

Mg(OH)2(active) -6.35 12.44 18.79 Mg(OH)2

MgCr2O4 -1.79 16.07 17.87 MgCr2O4

MgCrO4 -24.46 -18.25 6.21 MgCrO4

Minium -39.97 37.47 77.44 Pb3O4

Mirabilite -8.14 -9.99 -1.85 Na2SO4:10H2O

Monteponite -7.90 8.16 16.06 CdO

Na2Cr2O7 -41.87 -51.98 -10.10 Na2Cr2O7

Na2CrO4 -24.39 -21.28 3.11 Na2CrO4

Nantokite -6.97 -14.09 -7.13 CuCl

Natron -9.42 -11.34 -1.92 Na2CO3:10H2O

Nesquehonite -3.87 -8.31 -4.45 MgCO3:3H2O

O2(g) -40.63 47.76 88.39 O2

Otavite -0.60 -12.59 -11.99 CdCO3

Pb(OH)2 -4.16 4.53 8.69 Pb(OH)2

Pb10(OH)6O(CO3)6 -70.46 -79.22 -8.76 Pb10(OH)6O(CO3)6

Pb2(OH)3Cl -11.15 -2.36 8.79 Pb2(OH)3Cl

Pb2O(OH)2 -17.13 9.06 26.19 Pb2O(OH)2

Pb2O3 -28.10 32.94 61.04 Pb2O3

Pb2OCO3 -11.51 -11.69 -0.18 Pb2OCO3

Pb3(AsO4)2 -22.76 -16.96 5.80 Pb3(AsO4)2

Pb3O2CO3 -19.21 -7.16 12.05 Pb3O2CO3

Pb3O2SO4 -17.23 -5.81 11.42 Pb3O2SO4

Pb4(OH)6SO4 -22.38 -1.28 21.10 Pb4(OH)6SO4

Pb4O3SO4 -24.43 -1.28 23.14 Pb4O3SO4

PbCrO4 -13.15 -26.16 -13.01 PbCrO4

Pbmetal -23.59 -19.35 4.24 Pb

PbO:0.3H2O -8.45 4.53 12.98 PbO:0.33H2O

Periclase -10.55 12.44 22.99 MgO

Phosgenite -14.72 -34.53 -19.81 PbCl2:PbCO3

Plattnerite -23.94 28.41 52.35 PbO2

Portlandite -11.31 12.69 24.00 Ca(OH)2

Smithsonite -2.96 -12.81 -9.85 ZnCO3

Tenorite -1.60 6.64 8.25 CuO

Thenardite -10.40 -9.99 0.41 Na2SO4

Thermonatrite -12.07 -11.34 0.73 Na2CO3:H2O

Zincite -4.23 7.94 12.17 ZnO

Zincosite -16.16 -11.46 4.70 ZnSO4

Zn(NO3)2:6H2O -18.17 -15.09 3.09 Zn(NO3)2:6H2O

Zn(OH)2 -4.26 7.94 12.20 Zn(OH)2

Zn(OH)2(am) -5.28 7.94 13.22 Zn(OH)2

Zn(OH)2(beta) -4.59 7.94 12.53 Zn(OH)2

Zn(OH)2(epsilon) -4.35 7.94 12.29 Zn(OH)2

Zn(OH)2(gamma) -3.79 7.94 11.73 Zn(OH)2

Zn2(OH)2SO4 -11.03 -3.53 7.50 Zn2(OH)2SO4

Zn2(OH)3Cl -10.73 4.46 15.19 Zn2(OH)3Cl

Zn3(AsO4)2:2.5H2O -20.39 -6.74 13.65 Zn3(AsO4)2:2.5H2O

Zn3O(SO4)2 -36.30 -14.99 21.31 Zn3O(SO4)2

Zn4(OH)6SO4 -16.05 12.35 28.40 Zn4(OH)6SO4

Zn5(OH)8Cl2 -21.64 16.86 38.50 Zn5(OH)8Cl2

ZnCl2 -22.62 -14.90 7.72 ZnCl2

ZnCO3:1H2O -2.55 -12.81 -10.26 ZnCO3:1H2O

Znmetal -43.15 -15.94 27.21 Zn

ZnO(active) -4.07 7.94 12.01 ZnO

ZnSO4:1H2O -11.24 -11.46 -0.23 ZnSO4:1H2O

**For a gas, SI = log10(fugacity). Fugacity = pressure * phi / 1 atm.

For ideal gases, phi = 1.

Initial solution 21.

----------------------------Distribution of species----------------------------

Log Log Log mole V

Species Molality Activity Molality Activity Gamma cm?mol

OH- 5.649e-07 5.314e-07 -6.248 -6.275 -0.027 (0)

H+ 6.107e-09 5.749e-09 -8.214 -8.240 -0.026 0.00

H2O 5.551e+01 9.999e-01 1.744 -0.000 0.000 18.02

As(3) 1.239e-20

H3AsO3 1.176e-20 1.176e-20 -19.929 -19.929 0.000 (0)

H2AsO3- 6.235e-22 5.843e-22 -21.205 -21.233 -0.028 (0)

HAsO3-2 6.068e-26 4.678e-26 -25.217 -25.330 -0.113 (0)

H4AsO3+ 3.576e-29 3.351e-29 -28.447 -28.475 -0.028 (0)

AsO3-3 3.279e-31 1.826e-31 -30.484 -30.738 -0.254 (0)

As(5) 8.698e-09

HAsO4-2 8.311e-09 6.407e-09 -8.080 -8.193 -0.113 (0)

H2AsO4- 3.822e-10 3.582e-10 -9.418 -9.446 -0.028 (0)

AsO4-3 4.401e-12 2.451e-12 -11.356 -11.611 -0.254 (0)

H3AsO4 3.072e-16 3.074e-16 -15.513 -15.512 0.000 (0)

C(4) 2.119e-03

HCO3- 2.052e-03 1.935e-03 -2.688 -2.713 -0.026 (0)

H2CO3 3.042e-05 3.042e-05 -4.517 -4.517 0.000 (0)

CO3-2 1.471e-05 1.155e-05 -4.832 -4.937 -0.105 (0)

CaHCO3+ 1.046e-05 9.863e-06 -4.981 -5.006 -0.025 (0)

CaCO3 5.458e-06 5.458e-06 -5.263 -5.263 0.000 (0)

MgHCO3+ 3.621e-06 3.408e-06 -5.441 -5.467 -0.026 (0)

MgCO3 1.394e-06 1.394e-06 -5.856 -5.856 0.000 (0)

NaHCO3 7.955e-08 7.955e-08 -7.099 -7.099 0.000 (0)

ZnCO3 3.396e-08 3.396e-08 -7.469 -7.469 0.000 (0)

CuCO3 2.256e-08 2.256e-08 -7.647 -7.647 0.000 (0)

NaCO3- 1.922e-08 1.811e-08 -7.716 -7.742 -0.026 (0)

PbCO3 5.198e-09 5.198e-09 -8.284 -8.284 0.000 (0)

ZnHCO3+ 2.442e-09 2.288e-09 -8.612 -8.641 -0.028 (0)

Cu(CO3)2-2 9.094e-10 7.011e-10 -9.041 -9.154 -0.113 (0)

Pb(CO3)2-2 2.246e-10 1.731e-10 -9.649 -9.762 -0.113 (0)

CdCO3 2.009e-10 2.009e-10 -9.697 -9.697 0.000 (0)

PbHCO3+ 1.681e-10 1.576e-10 -9.774 -9.803 -0.028 (0)

CuHCO3+ 3.163e-11 2.964e-11 -10.500 -10.528 -0.028 (0)

CdHCO3+ 2.626e-12 2.461e-12 -11.581 -11.609 -0.028 (0)

Cd(CO3)2-2 2.231e-12 1.720e-12 -11.651 -11.764 -0.113 (0)

Ca 5.661e-04

Ca+2 5.346e-04 4.198e-04 -3.272 -3.377 -0.105 (0)

CaSO4 1.536e-05 1.536e-05 -4.814 -4.814 0.000 (0)

CaHCO3+ 1.046e-05 9.863e-06 -4.981 -5.006 -0.025 (0)

CaCO3 5.458e-06 5.458e-06 -5.263 -5.263 0.000 (0)

CaNO3+ 2.392e-07 2.241e-07 -6.621 -6.650 -0.028 (0)

CaOH+ 3.953e-09 3.728e-09 -8.403 -8.429 -0.025 (0)

Cd 1.211e-09

Cd+2 9.720e-10 7.633e-10 -9.012 -9.117 -0.105 (0)

CdCO3 2.009e-10 2.009e-10 -9.697 -9.697 0.000 (0)

CdSO4 2.762e-11 2.762e-11 -10.559 -10.559 0.000 (0)

CdOH+ 3.513e-12 3.292e-12 -11.454 -11.483 -0.028 (0)

CdHCO3+ 2.626e-12 2.461e-12 -11.581 -11.609 -0.028 (0)

Cd(CO3)2-2 2.231e-12 1.720e-12 -11.651 -11.764 -0.113 (0)

CdCl+ 1.516e-12 1.420e-12 -11.819 -11.848 -0.028 (0)

CdNO3+ 6.069e-13 5.687e-13 -12.217 -12.245 -0.028 (0)

Cd(OH)2 1.174e-13 1.174e-13 -12.930 -12.930 0.000 (0)

Cd(SO4)2-2 1.082e-13 8.339e-14 -12.966 -13.079 -0.113 (0)

CdOHCl 7.062e-14 7.062e-14 -13.151 -13.151 0.000 (0)

CdCl2 1.129e-16 1.129e-16 -15.947 -15.947 0.000 (0)

Cd(NO3)2 2.737e-17 2.737e-17 -16.563 -16.563 0.000 (0)

Cd(OH)3- 1.340e-17 1.256e-17 -16.873 -16.901 -0.028 (0)

Cd2OH+3 2.741e-20 1.526e-20 -19.562 -19.816 -0.254 (0)

CdCl3- 1.303e-21 1.221e-21 -20.885 -20.913 -0.028 (0)

Cd(OH)4-2 4.670e-24 3.600e-24 -23.331 -23.444 -0.113 (0)

Cl 2.114e-05

Cl- 2.114e-05 1.990e-05 -4.675 -4.701 -0.026 (0)

ZnOHCl 5.858e-12 5.858e-12 -11.232 -11.232 0.000 (0)

ZnCl+ 2.418e-12 2.276e-12 -11.616 -11.643 -0.026 (0)

CdCl+ 1.516e-12 1.420e-12 -11.819 -11.848 -0.028 (0)

CuCl 3.513e-13 3.513e-13 -12.454 -12.454 0.000 (0)

PbCl+ 9.371e-14 8.781e-14 -13.028 -13.056 -0.028 (0)

CdOHCl 7.062e-14 7.062e-14 -13.151 -13.151 0.000 (0)

CuCl+ 9.309e-15 8.762e-15 -14.031 -14.057 -0.026 (0)

CuCl2- 1.611e-15 1.517e-15 -14.793 -14.819 -0.026 (0)

CdCl2 1.129e-16 1.129e-16 -15.947 -15.947 0.000 (0)

ZnCl2 3.655e-17 3.655e-17 -16.437 -16.437 0.000 (0)

CrCl+2 1.545e-17 1.191e-17 -16.811 -16.924 -0.113 (0)

PbCl2 7.275e-18 7.275e-18 -17.138 -17.138 0.000 (0)

CuCl2 2.809e-20 2.809e-20 -19.551 -19.551 0.000 (0)

CuCl3-2 7.705e-21 6.073e-21 -20.113 -20.217 -0.103 (0)

CdCl3- 1.303e-21 1.221e-21 -20.885 -20.913 -0.028 (0)

CrOHCl2 8.623e-22 8.623e-22 -21.064 -21.064 0.000 (0)

ZnCl3- 5.758e-22 5.420e-22 -21.240 -21.266 -0.026 (0)

PbCl3- 7.299e-23 6.839e-23 -22.137 -22.165 -0.028 (0)

CrCl2+ 6.065e-24 5.683e-24 -23.217 -23.245 -0.028 (0)

ZnCl4-2 6.039e-27 4.759e-27 -26.219 -26.322 -0.103 (0)

CuCl3- 4.190e-27 3.944e-27 -26.378 -26.404 -0.026 (0)

CrO3Cl- 7.605e-28 7.126e-28 -27.119 -27.147 -0.028 (0)

PbCl4-2 6.411e-28 4.943e-28 -27.193 -27.306 -0.113 (0)

CuCl4-2 8.467e-34 6.673e-34 -33.072 -33.176 -0.103 (0)

Cr(2) 1.367e-24

Cr+2 1.367e-24 1.054e-24 -23.864 -23.977 -0.113 (0)

Cr(3) 7.693e-07

Cr(OH)2+ 4.403e-07 4.126e-07 -6.356 -6.384 -0.028 (0)

Cr(OH)3 2.715e-07 2.715e-07 -6.566 -6.566 0.000 (0)

CrO2- 2.394e-08 2.243e-08 -7.621 -7.649 -0.028 (0)

Cr(OH)4- 2.020e-08 1.893e-08 -7.695 -7.723 -0.028 (0)

Cr(OH)+2 1.327e-08 1.023e-08 -7.877 -7.990 -0.113 (0)

CrOHSO4 8.539e-11 8.539e-11 -10.069 -10.069 0.000 (0)

Cr+3 1.448e-12 8.062e-13 -11.839 -12.094 -0.254 (0)

CrSO4+ 1.925e-13 1.804e-13 -12.715 -12.744 -0.028 (0)

Cr2(OH)2SO4+2 1.938e-17 1.494e-17 -16.713 -16.826 -0.113 (0)

CrCl+2 1.545e-17 1.191e-17 -16.811 -16.924 -0.113 (0)

CrNO3+2 1.745e-18 1.345e-18 -17.758 -17.871 -0.113 (0)

Cr2(OH)2(SO4)2 1.650e-19 1.650e-19 -18.783 -18.783 0.000 (0)

CrOHCl2 8.623e-22 8.623e-22 -21.064 -21.064 0.000 (0)

CrCl2+ 6.065e-24 5.683e-24 -23.217 -23.245 -0.028 (0)

Cr(6) 6.882e-14

CrO4-2 6.779e-14 5.323e-14 -13.169 -13.274 -0.105 (0)

HCrO4- 1.013e-15 9.488e-16 -14.995 -15.023 -0.028 (0)

NaCrO4- 1.539e-17 1.442e-17 -16.813 -16.841 -0.028 (0)

KCrO4- 5.960e-18 5.585e-18 -17.225 -17.253 -0.028 (0)

H2CrO4 2.005e-24 2.005e-24 -23.698 -23.698 0.000 (0)

CrO3SO4-2 4.180e-25 3.223e-25 -24.379 -24.492 -0.113 (0)

CrO3Cl- 7.605e-28 7.126e-28 -27.119 -27.147 -0.028 (0)

Cr2O7-2 6.077e-29 4.685e-29 -28.216 -28.329 -0.113 (0)

Cu(1) 1.531e-11

Cu+ 1.496e-11 1.402e-11 -10.825 -10.853 -0.028 (0)

CuCl 3.513e-13 3.513e-13 -12.454 -12.454 0.000 (0)

CuCl2- 1.611e-15 1.517e-15 -14.793 -14.819 -0.026 (0)

CuCl3-2 7.705e-21 6.073e-21 -20.113 -20.217 -0.103 (0)

Cu(2) 2.548e-08

CuCO3 2.256e-08 2.256e-08 -7.647 -7.647 0.000 (0)

Cu(CO3)2-2 9.094e-10 7.011e-10 -9.041 -9.154 -0.113 (0)

CuOH+ 9.080e-10 8.547e-10 -9.042 -9.068 -0.026 (0)

Cu(OH)2 6.420e-10 6.420e-10 -9.192 -9.192 0.000 (0)

Cu+2 4.224e-10 3.317e-10 -9.374 -9.479 -0.105 (0)

CuHCO3+ 3.163e-11 2.964e-11 -10.500 -10.528 -0.028 (0)

CuSO4 1.173e-11 1.173e-11 -10.931 -10.931 0.000 (0)

Cu(OH)3- 2.461e-12 2.306e-12 -11.609 -11.637 -0.028 (0)

CuNO3+ 1.838e-13 1.722e-13 -12.736 -12.764 -0.028 (0)

Cu2(OH)2+2 2.139e-14 1.649e-14 -13.670 -13.783 -0.113 (0)

CuCl+ 9.309e-15 8.762e-15 -14.031 -14.057 -0.026 (0)

Cu(OH)4-2 4.124e-17 3.180e-17 -16.385 -16.498 -0.113 (0)

Cu(NO3)2 2.988e-18 2.988e-18 -17.525 -17.525 0.000 (0)

CuCl2 2.809e-20 2.809e-20 -19.551 -19.551 0.000 (0)

CuCl3- 4.190e-27 3.944e-27 -26.378 -26.404 -0.026 (0)

CuCl4-2 8.467e-34 6.673e-34 -33.072 -33.176 -0.103 (0)

H(0) 5.472e-28

H2 2.736e-28 2.738e-28 -27.563 -27.563 0.000 (0)

K 3.003e-05

K+ 3.000e-05 2.824e-05 -4.523 -4.549 -0.026 (0)

KSO4- 3.611e-08 3.404e-08 -7.442 -7.468 -0.026 (0)

KCrO4- 5.960e-18 5.585e-18 -17.225 -17.253 -0.028 (0)

Mg 2.495e-04

Mg+2 2.388e-04 1.875e-04 -3.622 -3.727 -0.105 (0)

MgSO4 5.603e-06 5.603e-06 -5.252 -5.252 0.000 (0)

MgHCO3+ 3.621e-06 3.408e-06 -5.441 -5.467 -0.026 (0)

MgCO3 1.394e-06 1.394e-06 -5.856 -5.856 0.000 (0)

MgOH+ 3.254e-08 3.070e-08 -7.488 -7.513 -0.025 (0)

N(5) 1.600e-04

NO3- 1.598e-04 1.504e-04 -3.796 -3.823 -0.026 (0)

CaNO3+ 2.392e-07 2.241e-07 -6.621 -6.650 -0.028 (0)

ZnNO3+ 2.273e-11 2.130e-11 -10.643 -10.672 -0.028 (0)

CdNO3+ 6.069e-13 5.687e-13 -12.217 -12.245 -0.028 (0)

PbNO3+ 3.407e-13 3.192e-13 -12.468 -12.496 -0.028 (0)

CuNO3+ 1.838e-13 1.722e-13 -12.736 -12.764 -0.028 (0)

Zn(NO3)2 5.795e-16 5.795e-16 -15.237 -15.237 0.000 (0)

Pb(NO3)2 9.799e-17 9.799e-17 -16.009 -16.009 0.000 (0)

Cd(NO3)2 2.737e-17 2.737e-17 -16.563 -16.563 0.000 (0)

Cu(NO3)2 2.988e-18 2.988e-18 -17.525 -17.525 0.000 (0)

CrNO3+2 1.745e-18 1.345e-18 -17.758 -17.871 -0.113 (0)

Na 5.808e-05

Na+ 5.792e-05 5.453e-05 -4.237 -4.263 -0.026 (0)

NaHCO3 7.955e-08 7.955e-08 -7.099 -7.099 0.000 (0)

NaSO4- 5.652e-08 5.327e-08 -7.248 -7.273 -0.026 (0)

NaCO3- 1.922e-08 1.811e-08 -7.716 -7.742 -0.026 (0)

NaCrO4- 1.539e-17 1.442e-17 -16.813 -16.841 -0.028 (0)

O(0) 0.000e+00

O2 0.000e+00 0.000e+00 -41.498 -41.497 0.000 (0)

Pb 6.535e-09

PbCO3 5.198e-09 5.198e-09 -8.284 -8.284 0.000 (0)

PbOH+ 7.031e-10 6.588e-10 -9.153 -9.181 -0.028 (0)

Pb(CO3)2-2 2.246e-10 1.731e-10 -9.649 -9.762 -0.113 (0)

Pb+2 1.907e-10 1.497e-10 -9.720 -9.825 -0.105 (0)

PbHCO3+ 1.681e-10 1.576e-10 -9.774 -9.803 -0.028 (0)

Pb(OH)2 3.649e-11 3.649e-11 -10.438 -10.438 0.000 (0)

PbSO4 1.363e-11 1.363e-11 -10.865 -10.865 0.000 (0)

PbNO3+ 3.407e-13 3.192e-13 -12.468 -12.496 -0.028 (0)

PbCl+ 9.371e-14 8.781e-14 -13.028 -13.056 -0.028 (0)

Pb(OH)3- 6.820e-14 6.391e-14 -13.166 -13.194 -0.028 (0)

Pb(SO4)2-2 1.980e-14 1.527e-14 -13.703 -13.816 -0.113 (0)

Pb(NO3)2 9.799e-17 9.799e-17 -16.009 -16.009 0.000 (0)

Pb(OH)4-2 3.556e-17 2.741e-17 -16.449 -16.562 -0.113 (0)

PbCl2 7.275e-18 7.275e-18 -17.138 -17.138 0.000 (0)

Pb2OH+3 2.807e-18 1.563e-18 -17.552 -17.806 -0.254 (0)

Pb3(OH)4+2 4.397e-22 3.390e-22 -21.357 -21.470 -0.113 (0)

PbCl3- 7.299e-23 6.839e-23 -22.137 -22.165 -0.028 (0)

Pb4(OH)4+4 2.032e-27 7.179e-28 -26.692 -27.144 -0.452 (0)

PbCl4-2 6.411e-28 4.943e-28 -27.193 -27.306 -0.113 (0)

S(6) 2.577e-04

SO4-2 2.367e-04 1.859e-04 -3.626 -3.731 -0.105 (0)

CaSO4 1.536e-05 1.536e-05 -4.814 -4.814 0.000 (0)

MgSO4 5.603e-06 5.603e-06 -5.252 -5.252 0.000 (0)

NaSO4- 5.652e-08 5.327e-08 -7.248 -7.273 -0.026 (0)

KSO4- 3.611e-08 3.404e-08 -7.442 -7.468 -0.026 (0)

ZnSO4 1.820e-09 1.820e-09 -8.740 -8.740 0.000 (0)

CrOHSO4 8.539e-11 8.539e-11 -10.069 -10.069 0.000 (0)

HSO4- 6.929e-11 6.525e-11 -10.159 -10.185 -0.026 (0)

CdSO4 2.762e-11 2.762e-11 -10.559 -10.559 0.000 (0)

PbSO4 1.363e-11 1.363e-11 -10.865 -10.865 0.000 (0)

CuSO4 1.173e-11 1.173e-11 -10.931 -10.931 0.000 (0)

Zn(SO4)2-2 4.363e-12 3.364e-12 -11.360 -11.473 -0.113 (0)

CrSO4+ 1.925e-13 1.804e-13 -12.715 -12.744 -0.028 (0)

Cd(SO4)2-2 1.082e-13 8.339e-14 -12.966 -13.079 -0.113 (0)

Pb(SO4)2-2 1.980e-14 1.527e-14 -13.703 -13.816 -0.113 (0)

Cr2(OH)2SO4+2 1.938e-17 1.494e-17 -16.713 -16.826 -0.113 (0)

Cr2(OH)2(SO4)2 1.650e-19 1.650e-19 -18.783 -18.783 0.000 (0)

CrO3SO4-2 4.180e-25 3.223e-25 -24.379 -24.492 -0.113 (0)

Zn 1.087e-07

Zn+2 6.507e-08 5.110e-08 -7.187 -7.292 -0.105 (0)

ZnCO3 3.396e-08 3.396e-08 -7.469 -7.469 0.000 (0)

ZnOH+ 2.898e-09 2.716e-09 -8.538 -8.566 -0.028 (0)

Zn(OH)2 2.484e-09 2.484e-09 -8.605 -8.605 0.000 (0)

ZnHCO3+ 2.442e-09 2.288e-09 -8.612 -8.641 -0.028 (0)

ZnSO4 1.820e-09 1.820e-09 -8.740 -8.740 0.000 (0)

Zn(OH)3- 2.327e-11 2.181e-11 -10.633 -10.661 -0.028 (0)

ZnNO3+ 2.273e-11 2.130e-11 -10.643 -10.672 -0.028 (0)

ZnOHCl 5.858e-12 5.858e-12 -11.232 -11.232 0.000 (0)

Zn(SO4)2-2 4.363e-12 3.364e-12 -11.360 -11.473 -0.113 (0)

ZnCl+ 2.418e-12 2.276e-12 -11.616 -11.643 -0.026 (0)

Zn(OH)4-2 1.972e-15 1.521e-15 -14.705 -14.818 -0.113 (0)

Zn(NO3)2 5.795e-16 5.795e-16 -15.237 -15.237 0.000 (0)

ZnCl2 3.655e-17 3.655e-17 -16.437 -16.437 0.000 (0)

ZnCl3- 5.758e-22 5.420e-22 -21.240 -21.266 -0.026 (0)

ZnCl4-2 6.039e-27 4.759e-27 -26.219 -26.322 -0.103 (0)

------------------------------Saturation indices-------------------------------

Phase SI** log IAP log K(283 K, 1 atm)

Anglesite -5.65 -13.56 -7.90 PbSO4

Anhydrite -2.81 -7.11 -4.29 CaSO4

Antlerite -7.99 0.79 8.79 Cu3(OH)4SO4

Aragonite -0.13 -8.31 -8.19 CaCO3

Arsenolite -76.40 -79.72 -3.32 As4O6

Artinite -6.63 4.09 10.72 MgCO3:Mg(OH)2:3H2O

As2O5 -37.94 -31.02 6.92 As2O5

Atacamite -7.20 1.06 8.26 Cu2(OH)3Cl

Azurite -5.81 -21.83 -16.02 Cu3(OH)2(CO3)2

Bianchite -9.26 -11.02 -1.76 ZnSO4:6H2O

Brochantite -9.31 7.79 17.10 Cu4(OH)6SO4

Brucite -5.15 12.75 17.90 Mg(OH)2

Ca3(AsO4)2:4H2O -14.01 8.29 22.30 Ca3(AsO4)2:4H2O

CaCrO4 -14.64 -16.65 -2.02 CaCrO4

Calcite 0.09 -8.31 -8.41 CaCO3

Cd(OH)2 -7.16 7.36 14.52 Cd(OH)2

Cd(OH)2(am) -7.17 7.36 14.54 Cd(OH)2

Cd3(OH)2(SO4)2 -25.04 -18.33 6.71 Cd3(OH)2(SO4)2

Cd3(OH)4SO4 -20.68 1.88 22.56 Cd3(OH)4SO4

Cd4(OH)6SO4 -19.16 9.24 28.40 Cd4(OH)6SO4

CdCl2 -18.03 -18.52 -0.49 CdCl2

CdCl2:1H2O -16.90 -18.52 -1.62 CdCl2:1H2O

CdCl2:2.5H2O -16.54 -18.52 -1.98 CdCl2:2.5H2O

Cdmetal(alpha) -31.33 -17.12 14.21 Cd

Cdmetal(gamma) -31.44 -17.12 14.32 Cd

CdOHCl -9.40 -5.58 3.82 CdOHCl

CdSO4 -13.16 -12.85 0.31 CdSO4

CdSO4:1H2O -11.41 -12.85 -1.43 CdSO4:1H2O

CdSO4:2.67H2O -11.14 -12.85 -1.71 CdSO4:2.67H2O

Cerussite -1.40 -14.76 -13.36 PbCO3

CH4(g) -75.91 -119.34 -43.43 CH4

Chalcanthite -10.51 -13.21 -2.70 CuSO4:5H2O

Claudetite -76.14 -79.72 -3.58 As4O6

CO2(g) -3.23 -21.42 -18.18 CO2

Cotunnite -14.20 -19.23 -5.02 PbCl2

Cr(OH)2 -18.65 -7.50 11.15 Cr(OH)2

Cr(OH)3 0.24 1.86 1.61 Cr(OH)3

Cr(OH)3(am) 2.61 1.86 -0.75 Cr(OH)3

Cr2O3 5.60 3.71 -1.89 Cr2O3

CrCl2 -48.50 -33.38 15.12 CrCl2

CrCl3 -53.21 -36.97 16.24 CrCl3

Crmetal -64.06 -31.98 32.08 Cr

CrO3 -26.59 -29.75 -3.16 CrO3

Cu(OH)2 -2.20 7.00 9.20 Cu(OH)2

Cu2(OH)3NO3 -7.98 1.94 9.92 Cu2(OH)3NO3

Cu2SO4 -23.66 -25.44 -1.77 Cu2SO4

Cu3(AsO4)2:2H2O -16.12 -10.02 6.10 Cu3(AsO4)2:2H2O

CuCO3 -2.92 -14.42 -11.50 CuCO3

CuCrO4 -17.31 -22.75 -5.44 CuCrO4

Cumetal -5.43 -14.85 -9.42 Cu

CuOCuSO4 -17.79 -6.21 11.58 CuOCuSO4

Cuprite -4.97 -5.23 -0.26 Cu2O

CuSO4 -16.83 -13.21 3.62 CuSO4

Dolomite(disordered) -0.87 -16.98 -16.11 CaMg(CO3)2

Dolomite(ordered) -0.26 -16.98 -16.72 CaMg(CO3)2

Epsomite -5.22 -7.46 -2.23 MgSO4:7H2O

Goslarite -8.88 -11.02 -2.14 ZnSO4:7H2O

Gypsum -2.49 -7.11 -4.62 CaSO4:2H2O

Halite -10.53 -8.96 1.57 NaCl

Huntite -5.34 -34.31 -28.97 CaMg3(CO3)4

Hydrocerussite -4.10 -22.87 -18.77 Pb3(OH)2(CO3)2

Hydromagnesite -15.17 -21.90 -6.74 Mg5(CO3)4(OH)2:4H2O

K2Cr2O7 -34.13 -52.13 -17.99 K2Cr2O7

K2CrO4 -21.69 -22.37 -0.68 K2CrO4

Langite -11.23 7.79 19.03 Cu4(OH)6SO4:H2O

Larnakite -6.67 -6.90 -0.23 PbO:PbSO4

Laurionite -6.91 -6.29 0.62 PbOHCl

Lime -21.40 13.10 34.50 CaO

Litharge -6.65 6.66 13.30 PbO

Magnesite -1.02 -8.66 -7.65 MgCO3

Malachite -1.40 -7.42 -6.01 Cu2(OH)2CO3

Massicot -6.86 6.66 13.51 PbO

Melanothallite -25.73 -18.88 6.85 CuCl2

Mg(OH)2(active) -6.04 12.75 18.79 Mg(OH)2

MgCr2O4 -1.40 16.47 17.87 MgCr2O4

MgCrO4 -23.21 -17.00 6.21 MgCrO4

Minium -32.99 44.45 77.44 Pb3O4

Mirabilite -10.41 -12.26 -1.85 Na2SO4:10H2O

Monteponite -8.70 7.36 16.06 CdO

Na2Cr2O7 -41.45 -51.56 -10.10 Na2Cr2O7

Na2CrO4 -24.91 -21.80 3.11 Na2CrO4

Nantokite -8.43 -15.55 -7.13 CuCl

Natron -11.54 -13.46 -1.92 Na2CO3:10H2O

Nesquehonite -4.22 -8.66 -4.45 MgCO3:3H2O

O2(g) -39.43 48.96 88.39 O2

Otavite -2.06 -14.05 -11.99 CdCO3

Pb(OH)2 -2.04 6.66 8.69 Pb(OH)2

Pb10(OH)6O(CO3)6 -53.19 -61.95 -8.76 Pb10(OH)6O(CO3)6

Pb2(OH)3Cl -8.42 0.37 8.79 Pb2(OH)3Cl

Pb2O(OH)2 -12.88 13.31 26.19 Pb2O(OH)2

Pb2O3 -23.25 37.79 61.04 Pb2O3

Pb2OCO3 -7.93 -8.11 -0.18 Pb2OCO3

Pb3(AsO4)2 -16.86 -11.06 5.80 Pb3(AsO4)2

Pb3O2CO3 -13.50 -1.45 12.05 Pb3O2CO3

Pb3O2SO4 -11.66 -0.24 11.42 Pb3O2SO4

Pb4(OH)6SO4 -14.69 6.41 21.10 Pb4(OH)6SO4

Pb4O3SO4 -16.73 6.41 23.14 Pb4O3SO4

PbCrO4 -10.09 -23.10 -13.01 PbCrO4

Pbmetal -22.06 -17.82 4.24 Pb

PbO:0.3H2O -6.32 6.66 12.98 PbO:0.33H2O

Periclase -10.23 12.75 22.99 MgO

Phosgenite -14.18 -33.99 -19.81 PbCl2:PbCO3

Plattnerite -21.21 31.14 52.35 PbO2

Portlandite -10.89 13.10 24.00 Ca(OH)2

Smithsonite -2.38 -12.23 -9.85 ZnCO3

Tenorite -1.24 7.00 8.25 CuO

Thenardite -12.66 -12.26 0.41 Na2SO4

Thermonatrite -14.20 -13.46 0.73 Na2CO3:H2O

Zincite -2.98 9.19 12.17 ZnO

Zincosite -15.72 -11.02 4.70 ZnSO4

Zn(NO3)2:6H2O -18.02 -14.94 3.09 Zn(NO3)2:6H2O

Zn(OH)2 -3.01 9.19 12.20 Zn(OH)2

Zn(OH)2(am) -4.03 9.19 13.22 Zn(OH)2

Zn(OH)2(beta) -3.34 9.19 12.53 Zn(OH)2

Zn(OH)2(epsilon) -3.10 9.19 12.29 Zn(OH)2

Zn(OH)2(gamma) -2.54 9.19 11.73 Zn(OH)2

Zn2(OH)2SO4 -9.33 -1.83 7.50 Zn2(OH)2SO4

Zn2(OH)3Cl -9.75 5.44 15.19 Zn2(OH)3Cl

Zn3(AsO4)2:2.5H2O -17.11 -3.46 13.65 Zn3(AsO4)2:2.5H2O

Zn3O(SO4)2 -34.16 -12.86 21.31 Zn3O(SO4)2

Zn4(OH)6SO4 -11.85 16.55 28.40 Zn4(OH)6SO4

Zn5(OH)8Cl2 -18.44 20.06 38.50 Zn5(OH)8Cl2

ZnCl2 -24.42 -16.69 7.72 ZnCl2

ZnCO3:1H2O -1.97 -12.23 -10.26 ZnCO3:1H2O

Znmetal -42.50 -15.29 27.21 Zn

ZnO(active) -2.82 9.19 12.01 ZnO

ZnSO4:1H2O -10.79 -11.02 -0.23 ZnSO4:1H2O

**For a gas, SI = log10(fugacity). Fugacity = pressure * phi / 1 atm.

For ideal gases, phi = 1.

Initial solution 22.

----------------------------Distribution of species----------------------------

Log Log Log mole V

Species Molality Activity Molality Activity Gamma cm?mol

OH- 5.651e-07 5.314e-07 -6.248 -6.275 -0.027 (0)

H+ 6.109e-09 5.749e-09 -8.214 -8.240 -0.026 0.00

H2O 5.551e+01 9.999e-01 1.744 -0.000 0.000 18.02

As(3) 9.490e-21

H3AsO3 9.012e-21 9.012e-21 -20.045 -20.045 0.000 (0)

H2AsO3- 4.779e-22 4.476e-22 -21.321 -21.349 -0.028 (0)

HAsO3-2 4.657e-26 3.584e-26 -25.332 -25.446 -0.114 (0)

H4AsO3+ 2.741e-29 2.567e-29 -28.562 -28.591 -0.028 (0)

AsO3-3 2.523e-31 1.399e-31 -30.598 -30.854 -0.256 (0)

As(5) 6.675e-09

HAsO4-2 6.379e-09 4.909e-09 -8.195 -8.309 -0.114 (0)

H2AsO4- 2.930e-10 2.744e-10 -9.533 -9.562 -0.028 (0)

AsO4-3 3.386e-12 1.878e-12 -11.470 -11.726 -0.256 (0)

H3AsO4 2.353e-16 2.355e-16 -15.628 -15.628 0.000 (0)

C(4) 2.297e-03

HCO3- 2.225e-03 2.097e-03 -2.653 -2.678 -0.026 (0)

H2CO3 3.297e-05 3.297e-05 -4.482 -4.482 0.000 (0)

CO3-2 1.596e-05 1.252e-05 -4.797 -4.903 -0.106 (0)

CaHCO3+ 1.146e-05 1.081e-05 -4.941 -4.966 -0.026 (0)

CaCO3 5.981e-06 5.981e-06 -5.223 -5.223 0.000 (0)

MgHCO3+ 4.052e-06 3.812e-06 -5.392 -5.419 -0.026 (0)

MgCO3 1.559e-06 1.559e-06 -5.807 -5.807 0.000 (0)

NaHCO3 9.305e-08 9.305e-08 -7.031 -7.031 0.000 (0)

ZnCO3 3.507e-08 3.507e-08 -7.455 -7.455 0.000 (0)

CuCO3 3.171e-08 3.171e-08 -7.499 -7.499 0.000 (0)

NaCO3- 2.249e-08 2.119e-08 -7.648 -7.674 -0.026 (0)

PbCO3 7.755e-09 7.755e-09 -8.110 -8.110 0.000 (0)

ZnHCO3+ 2.523e-09 2.363e-09 -8.598 -8.627 -0.028 (0)

Cu(CO3)2-2 1.388e-09 1.068e-09 -8.858 -8.971 -0.114 (0)

Pb(CO3)2-2 3.638e-10 2.799e-10 -9.439 -9.553 -0.114 (0)

PbHCO3+ 2.510e-10 2.351e-10 -9.600 -9.629 -0.028 (0)

CdCO3 1.349e-10 1.349e-10 -9.870 -9.870 0.000 (0)

CuHCO3+ 4.448e-11 4.166e-11 -10.352 -10.380 -0.028 (0)

CdHCO3+ 1.764e-12 1.653e-12 -11.753 -11.782 -0.028 (0)

Cd(CO3)2-2 1.627e-12 1.252e-12 -11.789 -11.902 -0.114 (0)

Ca 5.721e-04

Ca+2 5.414e-04 4.245e-04 -3.266 -3.372 -0.106 (0)

CaSO4 1.303e-05 1.303e-05 -4.885 -4.885 0.000 (0)

CaHCO3+ 1.146e-05 1.081e-05 -4.941 -4.966 -0.026 (0)

CaCO3 5.981e-06 5.981e-06 -5.223 -5.223 0.000 (0)

CaNO3+ 2.367e-07 2.217e-07 -6.626 -6.654 -0.028 (0)

CaOH+ 3.998e-09 3.769e-09 -8.398 -8.424 -0.026 (0)

Cd 7.596e-10

Cd+2 6.033e-10 4.730e-10 -9.219 -9.325 -0.106 (0)

CdCO3 1.349e-10 1.349e-10 -9.870 -9.870 0.000 (0)

CdSO4 1.436e-11 1.436e-11 -10.843 -10.843 0.000 (0)

CdOH+ 2.178e-12 2.040e-12 -11.662 -11.690 -0.028 (0)

CdHCO3+ 1.764e-12 1.653e-12 -11.753 -11.782 -0.028 (0)

Cd(CO3)2-2 1.627e-12 1.252e-12 -11.789 -11.902 -0.114 (0)

CdCl+ 9.721e-13 9.105e-13 -12.012 -12.041 -0.028 (0)

CdNO3+ 3.681e-13 3.447e-13 -12.434 -12.463 -0.028 (0)

Cd(OH)2 7.272e-14 7.272e-14 -13.138 -13.138 0.000 (0)

Cd(SO4)2-2 4.728e-14 3.639e-14 -13.325 -13.439 -0.114 (0)

CdOHCl 4.527e-14 4.527e-14 -13.344 -13.344 0.000 (0)

CdCl2 7.489e-17 7.489e-17 -16.126 -16.126 0.000 (0)

Cd(NO3)2 1.623e-17 1.623e-17 -16.790 -16.790 0.000 (0)

Cd(OH)3- 8.308e-18 7.781e-18 -17.081 -17.109 -0.028 (0)

Cd2OH+3 1.057e-20 5.860e-21 -19.976 -20.232 -0.256 (0)

CdCl3- 8.947e-22 8.379e-22 -21.048 -21.077 -0.028 (0)

Cd(OH)4-2 2.899e-24 2.231e-24 -23.538 -23.652 -0.114 (0)

Cl 2.189e-05

Cl- 2.189e-05 2.059e-05 -4.660 -4.686 -0.026 (0)

ZnOHCl 5.775e-12 5.775e-12 -11.238 -11.238 0.000 (0)

ZnCl+ 2.385e-12 2.244e-12 -11.622 -11.649 -0.026 (0)

CdCl+ 9.721e-13 9.105e-13 -12.012 -12.041 -0.028 (0)

CuCl 4.714e-13 4.714e-13 -12.327 -12.327 0.000 (0)

PbCl+ 1.335e-13 1.251e-13 -12.874 -12.903 -0.028 (0)

CdOHCl 4.527e-14 4.527e-14 -13.344 -13.344 0.000 (0)

CuCl+ 1.250e-14 1.176e-14 -13.903 -13.930 -0.026 (0)

CuCl2- 2.238e-15 2.106e-15 -14.650 -14.677 -0.026 (0)

CdCl2 7.489e-17 7.489e-17 -16.126 -16.126 0.000 (0)

ZnCl2 3.728e-17 3.728e-17 -16.428 -16.428 0.000 (0)

CrCl+2 1.602e-17 1.232e-17 -16.795 -16.909 -0.114 (0)

PbCl2 1.072e-17 1.072e-17 -16.970 -16.970 0.000 (0)

CuCl2 3.901e-20 3.901e-20 -19.409 -19.409 0.000 (0)

CuCl3-2 1.109e-20 8.725e-21 -19.955 -20.059 -0.104 (0)

CrOHCl2 9.229e-22 9.229e-22 -21.035 -21.035 0.000 (0)

CdCl3- 8.947e-22 8.379e-22 -21.048 -21.077 -0.028 (0)

ZnCl3- 6.080e-22 5.720e-22 -21.216 -21.243 -0.026 (0)

PbCl3- 1.113e-22 1.043e-22 -21.953 -21.982 -0.028 (0)

CrCl2+ 6.493e-24 6.082e-24 -23.188 -23.216 -0.028 (0)

ZnCl4-2 6.605e-27 5.197e-27 -26.180 -26.284 -0.104 (0)

CuCl3- 6.023e-27 5.667e-27 -26.220 -26.247 -0.026 (0)

PbCl4-2 1.013e-27 7.798e-28 -26.994 -27.108 -0.114 (0)

CrO3Cl- 7.870e-28 7.371e-28 -27.104 -27.132 -0.028 (0)

CuCl4-2 1.261e-33 9.921e-34 -32.899 -33.003 -0.104 (0)

Cr(2) 1.369e-24

Cr+2 1.369e-24 1.053e-24 -23.864 -23.977 -0.114 (0)

Cr(3) 7.693e-07

Cr(OH)2+ 4.404e-07 4.125e-07 -6.356 -6.385 -0.028 (0)

Cr(OH)3 2.714e-07 2.714e-07 -6.566 -6.566 0.000 (0)

CrO2- 2.394e-08 2.242e-08 -7.621 -7.649 -0.028 (0)

Cr(OH)4- 2.020e-08 1.892e-08 -7.695 -7.723 -0.028 (0)

Cr(OH)+2 1.329e-08 1.023e-08 -7.876 -7.990 -0.114 (0)

CrOHSO4 7.164e-11 7.164e-11 -10.145 -10.145 0.000 (0)

Cr+3 1.453e-12 8.060e-13 -11.838 -12.094 -0.256 (0)

CrSO4+ 1.616e-13 1.514e-13 -12.792 -12.820 -0.028 (0)

Cr2(OH)2SO4+2 1.629e-17 1.253e-17 -16.788 -16.902 -0.114 (0)

CrCl+2 1.602e-17 1.232e-17 -16.795 -16.909 -0.114 (0)

CrNO3+2 1.709e-18 1.315e-18 -17.767 -17.881 -0.114 (0)

Cr2(OH)2(SO4)2 1.161e-19 1.161e-19 -18.935 -18.935 0.000 (0)

CrOHCl2 9.229e-22 9.229e-22 -21.035 -21.035 0.000 (0)

CrCl2+ 6.493e-24 6.082e-24 -23.188 -23.216 -0.028 (0)

Cr(6) 6.891e-14

CrO4-2 6.788e-14 5.322e-14 -13.168 -13.274 -0.106 (0)

HCrO4- 1.013e-15 9.485e-16 -14.995 -15.023 -0.028 (0)

NaCrO4- 1.662e-17 1.556e-17 -16.779 -16.808 -0.028 (0)

KCrO4- 6.547e-18 6.132e-18 -17.184 -17.212 -0.028 (0)

H2CrO4 2.005e-24 2.005e-24 -23.698 -23.698 0.000 (0)

CrO3SO4-2 3.513e-25 2.704e-25 -24.454 -24.568 -0.114 (0)

CrO3Cl- 7.870e-28 7.371e-28 -27.104 -27.132 -0.028 (0)

Cr2O7-2 6.085e-29 4.682e-29 -28.216 -28.330 -0.114 (0)

Cu(1) 1.989e-11

Cu+ 1.941e-11 1.818e-11 -10.712 -10.740 -0.028 (0)

CuCl 4.714e-13 4.714e-13 -12.327 -12.327 0.000 (0)

CuCl2- 2.238e-15 2.106e-15 -14.650 -14.677 -0.026 (0)

CuCl3-2 1.109e-20 8.725e-21 -19.955 -20.059 -0.104 (0)

Cu(2) 3.572e-08

CuCO3 3.171e-08 3.171e-08 -7.499 -7.499 0.000 (0)

Cu(CO3)2-2 1.388e-09 1.068e-09 -8.858 -8.971 -0.114 (0)

CuOH+ 1.178e-09 1.109e-09 -8.929 -8.955 -0.026 (0)

Cu(OH)2 8.328e-10 8.328e-10 -9.079 -9.079 0.000 (0)

Cu+2 5.488e-10 4.302e-10 -9.261 -9.366 -0.106 (0)

CuHCO3+ 4.448e-11 4.166e-11 -10.352 -10.380 -0.028 (0)

CuSO4 1.277e-11 1.277e-11 -10.894 -10.894 0.000 (0)

Cu(OH)3- 3.194e-12 2.992e-12 -11.496 -11.524 -0.028 (0)

CuNO3+ 2.333e-13 2.185e-13 -12.632 -12.660 -0.028 (0)

Cu2(OH)2+2 3.605e-14 2.774e-14 -13.443 -13.557 -0.114 (0)

CuCl+ 1.250e-14 1.176e-14 -13.903 -13.930 -0.026 (0)

Cu(OH)4-2 5.360e-17 4.124e-17 -16.271 -16.385 -0.114 (0)

Cu(NO3)2 3.709e-18 3.709e-18 -17.431 -17.431 0.000 (0)

CuCl2 3.901e-20 3.901e-20 -19.409 -19.409 0.000 (0)

CuCl3- 6.023e-27 5.667e-27 -26.220 -26.247 -0.026 (0)

CuCl4-2 1.261e-33 9.921e-34 -32.899 -33.003 -0.104 (0)

H(0) 5.472e-28

H2 2.736e-28 2.738e-28 -27.563 -27.563 0.000 (0)

K 3.299e-05

K+ 3.296e-05 3.101e-05 -4.482 -4.508 -0.026 (0)

KSO4- 3.330e-08 3.137e-08 -7.478 -7.503 -0.026 (0)

KCrO4- 6.547e-18 6.132e-18 -17.184 -17.212 -0.028 (0)

Mg 2.574e-04

Mg+2 2.469e-04 1.935e-04 -3.608 -3.713 -0.106 (0)

MgSO4 4.853e-06 4.853e-06 -5.314 -5.314 0.000 (0)

MgHCO3+ 4.052e-06 3.812e-06 -5.392 -5.419 -0.026 (0)

MgCO3 1.559e-06 1.559e-06 -5.807 -5.807 0.000 (0)

MgOH+ 3.359e-08 3.168e-08 -7.474 -7.499 -0.025 (0)

N(5) 1.566e-04

NO3- 1.564e-04 1.472e-04 -3.806 -3.832 -0.026 (0)

CaNO3+ 2.367e-07 2.217e-07 -6.626 -6.654 -0.028 (0)

ZnNO3+ 2.120e-11 1.986e-11 -10.674 -10.702 -0.028 (0)

PbNO3+ 4.590e-13 4.299e-13 -12.338 -12.367 -0.028 (0)

CdNO3+ 3.681e-13 3.447e-13 -12.434 -12.463 -0.028 (0)

CuNO3+ 2.333e-13 2.185e-13 -12.632 -12.660 -0.028 (0)

Zn(NO3)2 5.284e-16 5.284e-16 -15.277 -15.277 0.000 (0)

Pb(NO3)2 1.291e-16 1.291e-16 -15.889 -15.889 0.000 (0)

Cd(NO3)2 1.623e-17 1.623e-17 -16.790 -16.790 0.000 (0)

Cu(NO3)2 3.709e-18 3.709e-18 -17.431 -17.431 0.000 (0)

CrNO3+2 1.709e-18 1.315e-18 -17.767 -17.881 -0.114 (0)

Na 6.271e-05

Na+ 6.255e-05 5.886e-05 -4.204 -4.230 -0.026 (0)

NaHCO3 9.305e-08 9.305e-08 -7.031 -7.031 0.000 (0)

NaSO4- 5.122e-08 4.826e-08 -7.291 -7.316 -0.026 (0)

NaCO3- 2.249e-08 2.119e-08 -7.648 -7.674 -0.026 (0)

NaCrO4- 1.662e-17 1.556e-17 -16.779 -16.808 -0.028 (0)

O(0) 0.000e+00

O2 0.000e+00 0.000e+00 -41.498 -41.497 0.000 (0)

Pb 9.668e-09

PbCO3 7.755e-09 7.755e-09 -8.110 -8.110 0.000 (0)

PbOH+ 9.683e-10 9.069e-10 -9.014 -9.042 -0.028 (0)

Pb(CO3)2-2 3.638e-10 2.799e-10 -9.439 -9.553 -0.114 (0)

Pb+2 2.629e-10 2.061e-10 -9.580 -9.686 -0.106 (0)

PbHCO3+ 2.510e-10 2.351e-10 -9.600 -9.629 -0.028 (0)

Pb(OH)2 5.023e-11 5.023e-11 -10.299 -10.299 0.000 (0)

PbSO4 1.575e-11 1.575e-11 -10.803 -10.803 0.000 (0)

PbNO3+ 4.590e-13 4.299e-13 -12.338 -12.367 -0.028 (0)

PbCl+ 1.335e-13 1.251e-13 -12.874 -12.903 -0.028 (0)

Pb(OH)3- 9.393e-14 8.798e-14 -13.027 -13.056 -0.028 (0)

Pb(SO4)2-2 1.923e-14 1.480e-14 -13.716 -13.830 -0.114 (0)

Pb(NO3)2 1.291e-16 1.291e-16 -15.889 -15.889 0.000 (0)

Pb(OH)4-2 4.904e-17 3.774e-17 -16.309 -16.423 -0.114 (0)

PbCl2 1.072e-17 1.072e-17 -16.970 -16.970 0.000 (0)

Pb2OH+3 5.342e-18 2.963e-18 -17.272 -17.528 -0.256 (0)

Pb3(OH)4+2 1.149e-21 8.842e-22 -20.940 -21.053 -0.114 (0)

PbCl3- 1.113e-22 1.043e-22 -21.953 -21.982 -0.028 (0)

Pb4(OH)4+4 7.352e-27 2.578e-27 -26.134 -26.589 -0.455 (0)

PbCl4-2 1.013e-27 7.798e-28 -26.994 -27.108 -0.114 (0)

S(6) 2.169e-04

SO4-2 1.989e-04 1.560e-04 -3.701 -3.807 -0.106 (0)

CaSO4 1.303e-05 1.303e-05 -4.885 -4.885 0.000 (0)

MgSO4 4.853e-06 4.853e-06 -5.314 -5.314 0.000 (0)

NaSO4- 5.122e-08 4.826e-08 -7.291 -7.316 -0.026 (0)

KSO4- 3.330e-08 3.137e-08 -7.478 -7.503 -0.026 (0)

ZnSO4 1.455e-09 1.455e-09 -8.837 -8.837 0.000 (0)

CrOHSO4 7.164e-11 7.164e-11 -10.145 -10.145 0.000 (0)

HSO4- 5.817e-11 5.476e-11 -10.235 -10.262 -0.026 (0)

PbSO4 1.575e-11 1.575e-11 -10.803 -10.803 0.000 (0)

CdSO4 1.436e-11 1.436e-11 -10.843 -10.843 0.000 (0)

CuSO4 1.277e-11 1.277e-11 -10.894 -10.894 0.000 (0)

Zn(SO4)2-2 2.933e-12 2.257e-12 -11.533 -11.646 -0.114 (0)

CrSO4+ 1.616e-13 1.514e-13 -12.792 -12.820 -0.028 (0)

Cd(SO4)2-2 4.728e-14 3.639e-14 -13.325 -13.439 -0.114 (0)

Pb(SO4)2-2 1.923e-14 1.480e-14 -13.716 -13.830 -0.114 (0)

Cr2(OH)2SO4+2 1.629e-17 1.253e-17 -16.788 -16.902 -0.114 (0)

Cr2(OH)2(SO4)2 1.161e-19 1.161e-19 -18.935 -18.935 0.000 (0)

CrO3SO4-2 3.513e-25 2.704e-25 -24.454 -24.568 -0.114 (0)

Zn 1.063e-07

Zn+2 6.210e-08 4.869e-08 -7.207 -7.313 -0.106 (0)

ZnCO3 3.507e-08 3.507e-08 -7.455 -7.455 0.000 (0)

ZnOH+ 2.763e-09 2.588e-09 -8.559 -8.587 -0.028 (0)

ZnHCO3+ 2.523e-09 2.363e-09 -8.598 -8.627 -0.028 (0)

Zn(OH)2 2.367e-09 2.367e-09 -8.626 -8.626 0.000 (0)

ZnSO4 1.455e-09 1.455e-09 -8.837 -8.837 0.000 (0)

Zn(OH)3- 2.219e-11 2.078e-11 -10.654 -10.682 -0.028 (0)

ZnNO3+ 2.120e-11 1.986e-11 -10.674 -10.702 -0.028 (0)

ZnOHCl 5.775e-12 5.775e-12 -11.238 -11.238 0.000 (0)

Zn(SO4)2-2 2.933e-12 2.257e-12 -11.533 -11.646 -0.114 (0)

ZnCl+ 2.385e-12 2.244e-12 -11.622 -11.649 -0.026 (0)

Zn(OH)4-2 1.883e-15 1.449e-15 -14.725 -14.839 -0.114 (0)

Zn(NO3)2 5.284e-16 5.284e-16 -15.277 -15.277 0.000 (0)

ZnCl2 3.728e-17 3.728e-17 -16.428 -16.428 0.000 (0)

ZnCl3- 6.080e-22 5.720e-22 -21.216 -21.243 -0.026 (0)

ZnCl4-2 6.605e-27 5.197e-27 -26.180 -26.284 -0.104 (0)

------------------------------Saturation indices-------------------------------

Phase SI** log IAP log K(283 K, 1 atm)

Anglesite -5.59 -13.49 -7.90 PbSO4

Anhydrite -2.89 -7.18 -4.29 CaSO4

Antlerite -7.73 1.06 8.79 Cu3(OH)4SO4

Aragonite -0.09 -8.27 -8.19 CaCO3

Arsenolite -76.86 -80.18 -3.32 As4O6

Artinite -6.56 4.15 10.72 MgCO3:Mg(OH)2:3H2O

As2O5 -38.17 -31.26 6.92 As2O5

Atacamite -6.96 1.30 8.26 Cu2(OH)3Cl

Azurite -5.40 -21.42 -16.02 Cu3(OH)2(CO3)2

Bianchite -9.36 -11.12 -1.76 ZnSO4:6H2O

Brochantite -8.93 8.17 17.10 Cu4(OH)6SO4

Brucite -5.13 12.77 17.90 Mg(OH)2

Ca3(AsO4)2:4H2O -14.23 8.07 22.30 Ca3(AsO4)2:4H2O

CaCrO4 -14.63 -16.65 -2.02 CaCrO4

Calcite 0.13 -8.27 -8.41 CaCO3

Cd(OH)2 -7.37 7.16 14.52 Cd(OH)2

Cd(OH)2(am) -7.38 7.16 14.54 Cd(OH)2

Cd3(OH)2(SO4)2 -25.82 -19.11 6.71 Cd3(OH)2(SO4)2

Cd3(OH)4SO4 -21.38 1.18 22.56 Cd3(OH)4SO4

Cd4(OH)6SO4 -20.07 8.33 28.40 Cd4(OH)6SO4

CdCl2 -18.21 -18.70 -0.49 CdCl2

CdCl2:1H2O -17.07 -18.70 -1.62 CdCl2:1H2O

CdCl2:2.5H2O -16.72 -18.70 -1.98 CdCl2:2.5H2O

Cdmetal(alpha) -31.54 -17.33 14.21 Cd

Cdmetal(gamma) -31.65 -17.33 14.32 Cd

CdOHCl -9.60 -5.77 3.82 CdOHCl

CdSO4 -13.44 -13.13 0.31 CdSO4

CdSO4:1H2O -11.70 -13.13 -1.43 CdSO4:1H2O

CdSO4:2.67H2O -11.43 -13.13 -1.71 CdSO4:2.67H2O

Cerussite -1.23 -14.59 -13.36 PbCO3

CH4(g) -75.88 -119.31 -43.43 CH4

Chalcanthite -10.48 -13.17 -2.70 CuSO4:5H2O

Claudetite -76.60 -80.18 -3.58 As4O6

CO2(g) -3.20 -21.38 -18.18 CO2

Cotunnite -14.04 -19.06 -5.02 PbCl2

Cr(OH)2 -18.65 -7.50 11.15 Cr(OH)2

Cr(OH)3 0.24 1.86 1.61 Cr(OH)3

Cr(OH)3(am) 2.61 1.86 -0.75 Cr(OH)3

Cr2O3 5.60 3.71 -1.89 Cr2O3

CrCl2 -48.47 -33.35 15.12 CrCl2

CrCl3 -53.16 -36.92 16.24 CrCl3

Crmetal -64.06 -31.98 32.08 Cr

CrO3 -26.59 -29.75 -3.16 CrO3

Cu(OH)2 -2.08 7.11 9.20 Cu(OH)2

Cu2(OH)3NO3 -7.77 2.16 9.92 Cu2(OH)3NO3

Cu2SO4 -23.51 -25.29 -1.77 Cu2SO4

Cu3(AsO4)2:2H2O -16.01 -9.91 6.10 Cu3(AsO4)2:2H2O

CuCO3 -2.77 -14.27 -11.50 CuCO3

CuCrO4 -17.20 -22.64 -5.44 CuCrO4

Cumetal -5.32 -14.74 -9.42 Cu

CuOCuSO4 -17.64 -6.06 11.58 CuOCuSO4

Cuprite -4.74 -5.00 -0.26 Cu2O

CuSO4 -16.79 -13.17 3.62 CuSO4

Dolomite(disordered) -0.78 -16.89 -16.11 CaMg(CO3)2

Dolomite(ordered) -0.17 -16.89 -16.72 CaMg(CO3)2

Epsomite -5.29 -7.52 -2.23 MgSO4:7H2O

Goslarite -8.98 -11.12 -2.14 ZnSO4:7H2O

Gypsum -2.56 -7.18 -4.62 CaSO4:2H2O

Halite -10.48 -8.92 1.57 NaCl

Huntite -5.15 -34.12 -28.97 CaMg3(CO3)4

Hydrocerussite -3.61 -22.38 -18.77 Pb3(OH)2(CO3)2

Hydromagnesite -14.96 -21.70 -6.74 Mg5(CO3)4(OH)2:4H2O

K2Cr2O7 -34.05 -52.05 -17.99 K2Cr2O7

K2CrO4 -21.61 -22.29 -0.68 K2CrO4

Langite -10.85 8.17 19.03 Cu4(OH)6SO4:H2O

Larnakite -6.47 -6.70 -0.23 PbO:PbSO4

Laurionite -6.75 -6.13 0.62 PbOHCl

Lime -21.39 13.11 34.50 CaO

Litharge -6.51 6.79 13.30 PbO

Magnesite -0.97 -8.62 -7.65 MgCO3

Malachite -1.14 -7.15 -6.01 Cu2(OH)2CO3

Massicot -6.72 6.79 13.51 PbO

Melanothallite -25.58 -18.74 6.85 CuCl2

Mg(OH)2(active) -6.03 12.77 18.79 Mg(OH)2

MgCr2O4 -1.39 16.48 17.87 MgCr2O4

MgCrO4 -23.19 -16.99 6.21 MgCrO4

Minium -32.57 44.87 77.44 Pb3O4

Mirabilite -10.42 -12.27 -1.85 Na2SO4:10H2O

Monteponite -8.91 7.16 16.06 CdO

Na2Cr2O7 -41.39 -51.49 -10.10 Na2Cr2O7

Na2CrO4 -24.85 -21.73 3.11 Na2CrO4

Nantokite -8.30 -15.43 -7.13 CuCl

Natron -11.44 -13.36 -1.92 Na2CO3:10H2O

Nesquehonite -4.17 -8.62 -4.45 MgCO3:3H2O

O2(g) -39.43 48.96 88.39 O2

Otavite -2.23 -14.23 -11.99 CdCO3

Pb(OH)2 -1.90 6.79 8.69 Pb(OH)2

Pb10(OH)6O(CO3)6 -51.59 -60.35 -8.76 Pb10(OH)6O(CO3)6

Pb2(OH)3Cl -8.13 0.66 8.79 Pb2(OH)3Cl

Pb2O(OH)2 -12.60 13.59 26.19 Pb2O(OH)2

Pb2O3 -22.97 38.07 61.04 Pb2O3

Pb2OCO3 -7.61 -7.79 -0.18 Pb2OCO3

Pb3(AsO4)2 -16.67 -10.87 5.80 Pb3(AsO4)2

Pb3O2CO3 -13.04 -1.00 12.05 Pb3O2CO3

Pb3O2SO4 -11.32 0.10 11.42 Pb3O2SO4

Pb4(OH)6SO4 -14.21 6.89 21.10 Pb4(OH)6SO4

Pb4O3SO4 -16.25 6.89 23.14 Pb4O3SO4

PbCrO4 -9.95 -22.96 -13.01 PbCrO4

Pbmetal -21.92 -17.69 4.24 Pb

PbO:0.3H2O -6.19 6.79 12.98 PbO:0.33H2O

Periclase -10.22 12.77 22.99 MgO

Phosgenite -13.84 -33.65 -19.81 PbCl2:PbCO3

Plattnerite -21.07 31.28 52.35 PbO2

Portlandite -10.89 13.11 24.00 Ca(OH)2

Smithsonite -2.36 -12.22 -9.85 ZnCO3

Tenorite -1.13 7.11 8.25 CuO

Thenardite -12.67 -12.27 0.41 Na2SO4

Thermonatrite -14.10 -13.36 0.73 Na2CO3:H2O

Zincite -3.00 9.17 12.17 ZnO

Zincosite -15.82 -11.12 4.70 ZnSO4

Zn(NO3)2:6H2O -18.06 -14.98 3.09 Zn(NO3)2:6H2O

Zn(OH)2 -3.03 9.17 12.20 Zn(OH)2

Zn(OH)2(am) -4.05 9.17 13.22 Zn(OH)2

Zn(OH)2(beta) -3.36 9.17 12.53 Zn(OH)2

Zn(OH)2(epsilon) -3.12 9.17 12.29 Zn(OH)2

Zn(OH)2(gamma) -2.57 9.17 11.73 Zn(OH)2

Zn2(OH)2SO4 -9.45 -1.95 7.50 Zn2(OH)2SO4

Zn2(OH)3Cl -9.78 5.41 15.19 Zn2(OH)3Cl

Zn3(AsO4)2:2.5H2O -17.40 -3.75 13.65 Zn3(AsO4)2:2.5H2O

Zn3O(SO4)2 -34.38 -13.07 21.31 Zn3O(SO4)2

Zn4(OH)6SO4 -12.01 16.39 28.40 Zn4(OH)6SO4

Zn5(OH)8Cl2 -18.51 19.99 38.50 Zn5(OH)8Cl2

ZnCl2 -24.41 -16.69 7.72 ZnCl2

ZnCO3:1H2O -1.96 -12.22 -10.26 ZnCO3:1H2O

Znmetal -42.52 -15.31 27.21 Zn

ZnO(active) -2.84 9.17 12.01 ZnO

ZnSO4:1H2O -10.89 -11.12 -0.23 ZnSO4:1H2O

**For a gas, SI = log10(fugacity). Fugacity = pressure * phi / 1 atm.

For ideal gases, phi = 1.

Initial solution 23.

----------------------------Distribution of species----------------------------

Log Log Log mole V

Species Molality Activity Molality Activity Gamma cm?mol

OH- 1.599e-07 1.496e-07 -6.796 -6.825 -0.029 (0)

H+ 2.180e-08 2.042e-08 -7.662 -7.690 -0.028 0.00

H2O 5.551e+01 9.999e-01 1.744 -0.000 0.000 18.02

As(3) 3.430e-18

H3AsO3 3.379e-18 3.379e-18 -17.471 -17.471 0.000 (0)

H2AsO3- 5.072e-20 4.725e-20 -19.295 -19.326 -0.031 (0)

HAsO3-2 1.414e-24 1.065e-24 -23.850 -23.973 -0.123 (0)

H4AsO3+ 3.669e-26 3.418e-26 -25.435 -25.466 -0.031 (0)

AsO3-3 2.215e-30 1.171e-30 -29.655 -29.932 -0.277 (0)

As(5) 1.782e-08

HAsO4-2 1.535e-08 1.157e-08 -7.814 -7.937 -0.123 (0)

H2AsO4- 2.465e-09 2.296e-09 -8.608 -8.639 -0.031 (0)

AsO4-3 2.356e-12 1.246e-12 -11.628 -11.905 -0.277 (0)

H3AsO4 6.994e-15 7.000e-15 -14.155 -14.155 0.000 (0)

C(4) 2.328e-03

HCO3- 2.188e-03 2.052e-03 -2.660 -2.688 -0.028 (0)

H2CO3 1.146e-04 1.146e-04 -3.941 -3.941 0.000 (0)

CaHCO3+ 1.470e-05 1.380e-05 -4.833 -4.860 -0.027 (0)

CO3-2 4.481e-06 3.450e-06 -5.349 -5.462 -0.114 (0)

MgHCO3+ 3.923e-06 3.674e-06 -5.406 -5.435 -0.028 (0)

CaCO3 2.151e-06 2.151e-06 -5.667 -5.667 0.000 (0)

MgCO3 4.230e-07 4.230e-07 -6.374 -6.374 0.000 (0)

NaHCO3 3.056e-07 3.056e-07 -6.515 -6.515 0.000 (0)

NaCO3- 2.089e-08 1.959e-08 -7.680 -7.708 -0.028 (0)

CuCO3 8.177e-09 8.177e-09 -8.087 -8.087 0.000 (0)

ZnCO3 5.012e-09 5.012e-09 -8.300 -8.300 0.000 (0)

ZnHCO3+ 1.288e-09 1.200e-09 -8.890 -8.921 -0.031 (0)

PbCO3 5.685e-10 5.685e-10 -9.245 -9.245 0.000 (0)

Cu(CO3)2-2 1.008e-10 7.592e-11 -9.997 -10.120 -0.123 (0)

PbHCO3+ 6.569e-11 6.120e-11 -10.182 -10.213 -0.031 (0)

CuHCO3+ 4.096e-11 3.816e-11 -10.388 -10.418 -0.031 (0)

CdCO3 1.052e-11 1.052e-11 -10.978 -10.978 0.000 (0)

Pb(CO3)2-2 7.509e-12 5.656e-12 -11.124 -11.247 -0.123 (0)

CdHCO3+ 4.913e-13 4.577e-13 -12.309 -12.339 -0.031 (0)

Cd(CO3)2-2 3.572e-14 2.691e-14 -13.447 -13.570 -0.123 (0)

Ca 7.575e-04

Ca+2 7.194e-04 5.538e-04 -3.143 -3.257 -0.114 (0)

CaSO4 2.079e-05 2.079e-05 -4.682 -4.682 0.000 (0)

CaHCO3+ 1.470e-05 1.380e-05 -4.833 -4.860 -0.027 (0)

CaCO3 2.151e-06 2.151e-06 -5.667 -5.667 0.000 (0)

CaNO3+ 5.103e-07 4.754e-07 -6.292 -6.323 -0.031 (0)

CaOH+ 1.475e-09 1.385e-09 -8.831 -8.859 -0.027 (0)

Cd 1.907e-10

Cd+2 1.738e-10 1.338e-10 -9.760 -9.873 -0.114 (0)

CdCO3 1.052e-11 1.052e-11 -10.978 -10.978 0.000 (0)

CdSO4 4.967e-12 4.967e-12 -11.304 -11.304 0.000 (0)

CdHCO3+ 4.913e-13 4.577e-13 -12.309 -12.339 -0.031 (0)

CdCl+ 4.860e-13 4.528e-13 -12.313 -12.344 -0.031 (0)

CdOH+ 1.744e-13 1.625e-13 -12.758 -12.789 -0.031 (0)

CdNO3+ 1.721e-13 1.603e-13 -12.764 -12.795 -0.031 (0)

Cd(CO3)2-2 3.572e-14 2.691e-14 -13.447 -13.570 -0.123 (0)

Cd(SO4)2-2 2.043e-14 1.539e-14 -13.690 -13.813 -0.123 (0)

CdOHCl 6.340e-15 6.340e-15 -14.198 -14.198 0.000 (0)

Cd(OH)2 1.631e-15 1.631e-15 -14.788 -14.788 0.000 (0)

CdCl2 6.547e-17 6.547e-17 -16.184 -16.184 0.000 (0)

Cd(NO3)2 1.241e-17 1.241e-17 -16.906 -16.906 0.000 (0)

Cd(OH)3- 5.275e-20 4.914e-20 -19.278 -19.309 -0.031 (0)

CdCl3- 1.382e-21 1.287e-21 -20.860 -20.890 -0.031 (0)

Cd2OH+3 2.499e-22 1.321e-22 -21.602 -21.879 -0.277 (0)

Cd(OH)4-2 5.265e-27 3.966e-27 -26.279 -26.402 -0.123 (0)

Cl 3.864e-05

Cl- 3.864e-05 3.620e-05 -4.413 -4.441 -0.028 (0)

ZnCl+ 2.184e-12 2.046e-12 -11.661 -11.689 -0.028 (0)

ZnOHCl 1.482e-12 1.482e-12 -11.829 -11.829 0.000 (0)

CuCl 7.753e-13 7.753e-13 -12.111 -12.111 0.000 (0)

CdCl+ 4.860e-13 4.528e-13 -12.313 -12.344 -0.031 (0)

PbCl+ 6.276e-14 5.847e-14 -13.202 -13.233 -0.031 (0)

CuCl+ 2.065e-14 1.934e-14 -13.685 -13.714 -0.028 (0)

CuCl2- 6.500e-15 6.087e-15 -14.187 -14.216 -0.028 (0)

CdOHCl 6.340e-15 6.340e-15 -14.198 -14.198 0.000 (0)

CrCl+2 3.664e-16 2.760e-16 -15.436 -15.559 -0.123 (0)

CdCl2 6.547e-17 6.547e-17 -16.184 -16.184 0.000 (0)

ZnCl2 5.974e-17 5.974e-17 -16.224 -16.224 0.000 (0)

PbCl2 8.810e-18 8.810e-18 -17.055 -17.055 0.000 (0)

CuCl2 1.128e-19 1.128e-19 -18.948 -18.948 0.000 (0)

CuCl3-2 5.734e-20 4.433e-20 -19.242 -19.353 -0.112 (0)

CrOHCl2 1.023e-20 1.023e-20 -19.990 -19.990 0.000 (0)

ZnCl3- 1.720e-21 1.611e-21 -20.764 -20.793 -0.028 (0)

CdCl3- 1.382e-21 1.287e-21 -20.860 -20.890 -0.031 (0)

CrCl2+ 2.570e-22 2.394e-22 -21.590 -21.621 -0.031 (0)

PbCl3- 1.617e-22 1.506e-22 -21.791 -21.822 -0.031 (0)

ZnCl4-2 3.328e-26 2.573e-26 -25.478 -25.590 -0.112 (0)

CuCl3- 3.074e-26 2.879e-26 -25.512 -25.541 -0.028 (0)

PbCl4-2 2.628e-27 1.980e-27 -26.580 -26.703 -0.123 (0)

CrO3Cl- 8.827e-30 8.224e-30 -29.054 -29.085 -0.031 (0)

CuCl4-2 1.146e-32 8.859e-33 -31.941 -32.053 -0.112 (0)

Cr(2) 1.782e-23

Cr+2 1.782e-23 1.342e-23 -22.749 -22.872 -0.123 (0)

Cr(3) 5.770e-07

Cr(OH)2+ 4.472e-07 4.166e-07 -6.350 -6.380 -0.031 (0)

Cr(OH)3 7.718e-08 7.718e-08 -7.112 -7.112 0.000 (0)

Cr(OH)+2 4.871e-08 3.670e-08 -7.312 -7.435 -0.123 (0)

CrO2- 1.927e-09 1.795e-09 -8.715 -8.746 -0.031 (0)

Cr(OH)4- 1.626e-09 1.515e-09 -8.789 -8.820 -0.031 (0)

CrOHSO4 3.142e-10 3.142e-10 -9.503 -9.503 0.000 (0)

Cr+3 1.943e-11 1.027e-11 -10.712 -10.988 -0.277 (0)

CrSO4+ 2.531e-12 2.358e-12 -11.597 -11.628 -0.031 (0)

CrCl+2 3.664e-16 2.760e-16 -15.436 -15.559 -0.123 (0)

Cr2(OH)2SO4+2 2.618e-16 1.972e-16 -15.582 -15.705 -0.123 (0)

CrNO3+2 3.657e-17 2.755e-17 -16.437 -16.560 -0.123 (0)

Cr2(OH)2(SO4)2 2.234e-18 2.234e-18 -17.651 -17.651 0.000 (0)

CrOHCl2 1.023e-20 1.023e-20 -19.990 -19.990 0.000 (0)

CrCl2+ 2.570e-22 2.394e-22 -21.590 -21.621 -0.031 (0)

Cr(6) 3.663e-17

CrO4-2 3.478e-17 2.678e-17 -16.459 -16.572 -0.114 (0)

HCrO4- 1.820e-18 1.695e-18 -17.740 -17.771 -0.031 (0)

NaCrO4- 2.821e-20 2.628e-20 -19.550 -19.580 -0.031 (0)

KCrO4- 4.110e-21 3.829e-21 -20.386 -20.417 -0.031 (0)

H2CrO4 1.272e-26 1.272e-26 -25.895 -25.895 0.000 (0)

CrO3SO4-2 2.785e-27 2.098e-27 -26.555 -26.678 -0.123 (0)

CrO3Cl- 8.827e-30 8.224e-30 -29.054 -29.085 -0.031 (0)

Cr2O7-2 1.985e-34 1.495e-34 -33.702 -33.825 -0.123 (0)

Cu(1) 1.904e-11

Cu+ 1.826e-11 1.701e-11 -10.738 -10.769 -0.031 (0)

CuCl 7.753e-13 7.753e-13 -12.111 -12.111 0.000 (0)

CuCl2- 6.500e-15 6.087e-15 -14.187 -14.216 -0.028 (0)

CuCl3-2 5.734e-20 4.433e-20 -19.242 -19.353 -0.112 (0)

Cu(2) 9.230e-09

CuCO3 8.177e-09 8.177e-09 -8.087 -8.087 0.000 (0)

Cu+2 5.229e-10 4.026e-10 -9.282 -9.395 -0.114 (0)

CuOH+ 3.118e-10 2.920e-10 -9.506 -9.535 -0.028 (0)

Cu(CO3)2-2 1.008e-10 7.592e-11 -9.997 -10.120 -0.123 (0)

Cu(OH)2 6.177e-11 6.177e-11 -10.209 -10.209 0.000 (0)

CuHCO3+ 4.096e-11 3.816e-11 -10.388 -10.418 -0.031 (0)

CuSO4 1.460e-11 1.460e-11 -10.836 -10.836 0.000 (0)

CuNO3+ 3.608e-13 3.361e-13 -12.443 -12.473 -0.031 (0)

Cu(OH)3- 6.706e-14 6.248e-14 -13.174 -13.204 -0.031 (0)

CuCl+ 2.065e-14 1.934e-14 -13.685 -13.714 -0.028 (0)

Cu2(OH)2+2 2.556e-15 1.925e-15 -14.592 -14.715 -0.123 (0)

Cu(NO3)2 9.378e-18 9.378e-18 -17.028 -17.028 0.000 (0)

Cu(OH)4-2 3.219e-19 2.425e-19 -18.492 -18.615 -0.123 (0)

CuCl2 1.128e-19 1.128e-19 -18.948 -18.948 0.000 (0)

CuCl3- 3.074e-26 2.879e-26 -25.512 -25.541 -0.028 (0)

CuCl4-2 1.146e-32 8.859e-33 -31.941 -32.053 -0.112 (0)

H(0) 6.902e-27

H2 3.451e-27 3.454e-27 -26.462 -26.462 0.000 (0)

K 4.114e-05

K+ 4.109e-05 3.849e-05 -4.386 -4.415 -0.028 (0)

KSO4- 5.074e-08 4.760e-08 -7.295 -7.322 -0.028 (0)

KCrO4- 4.110e-21 3.829e-21 -20.386 -20.417 -0.031 (0)

Mg 2.577e-04

Mg+2 2.475e-04 1.905e-04 -3.606 -3.720 -0.114 (0)

MgSO4 5.841e-06 5.841e-06 -5.234 -5.234 0.000 (0)

MgHCO3+ 3.923e-06 3.674e-06 -5.406 -5.435 -0.028 (0)

MgCO3 4.230e-07 4.230e-07 -6.374 -6.374 0.000 (0)

MgOH+ 9.350e-09 8.782e-09 -8.029 -8.056 -0.027 (0)

N(5) 2.588e-04

NO3- 2.582e-04 2.419e-04 -3.588 -3.616 -0.028 (0)

CaNO3+ 5.103e-07 4.754e-07 -6.292 -6.323 -0.031 (0)

ZnNO3+ 1.817e-11 1.693e-11 -10.741 -10.771 -0.031 (0)

CuNO3+ 3.608e-13 3.361e-13 -12.443 -12.473 -0.031 (0)

PbNO3+ 2.018e-13 1.880e-13 -12.695 -12.726 -0.031 (0)

CdNO3+ 1.721e-13 1.603e-13 -12.764 -12.795 -0.031 (0)

Zn(NO3)2 7.405e-16 7.405e-16 -15.130 -15.130 0.000 (0)

Pb(NO3)2 9.279e-17 9.279e-17 -16.032 -16.032 0.000 (0)

CrNO3+2 3.657e-17 2.755e-17 -16.437 -16.560 -0.123 (0)

Cd(NO3)2 1.241e-17 1.241e-17 -16.906 -16.906 0.000 (0)

Cu(NO3)2 9.378e-18 9.378e-18 -17.028 -17.028 0.000 (0)

Na 2.114e-04

Na+ 2.108e-04 1.975e-04 -3.676 -3.704 -0.028 (0)

NaHCO3 3.056e-07 3.056e-07 -6.515 -6.515 0.000 (0)

NaSO4- 2.110e-07 1.980e-07 -6.676 -6.703 -0.028 (0)

NaCO3- 2.089e-08 1.959e-08 -7.680 -7.708 -0.028 (0)

NaCrO4- 2.821e-20 2.628e-20 -19.550 -19.580 -0.031 (0)

O(0) 0.000e+00

O2 0.000e+00 0.000e+00 -43.699 -43.699 0.000 (0)

Pb 7.923e-10

PbCO3 5.685e-10 5.685e-10 -9.245 -9.245 0.000 (0)

PbOH+ 7.290e-11 6.791e-11 -10.137 -10.168 -0.031 (0)

Pb+2 7.122e-11 5.483e-11 -10.147 -10.261 -0.114 (0)

PbHCO3+ 6.569e-11 6.120e-11 -10.182 -10.213 -0.031 (0)

Pb(CO3)2-2 7.509e-12 5.656e-12 -11.124 -11.247 -0.123 (0)

PbSO4 5.121e-12 5.121e-12 -11.291 -11.291 0.000 (0)

Pb(OH)2 1.059e-12 1.059e-12 -11.975 -11.975 0.000 (0)

PbNO3+ 2.018e-13 1.880e-13 -12.695 -12.726 -0.031 (0)

PbCl+ 6.276e-14 5.847e-14 -13.202 -13.233 -0.031 (0)

Pb(SO4)2-2 7.811e-15 5.884e-15 -14.107 -14.230 -0.123 (0)

Pb(OH)3- 5.606e-16 5.223e-16 -15.251 -15.282 -0.031 (0)

Pb(NO3)2 9.279e-17 9.279e-17 -16.032 -16.032 0.000 (0)

PbCl2 8.810e-18 8.810e-18 -17.055 -17.055 0.000 (0)

Pb2OH+3 1.116e-19 5.901e-20 -18.952 -19.229 -0.277 (0)

Pb(OH)4-2 8.373e-20 6.308e-20 -19.077 -19.200 -0.123 (0)

PbCl3- 1.617e-22 1.506e-22 -21.791 -21.822 -0.031 (0)

Pb3(OH)4+2 1.388e-25 1.046e-25 -24.858 -24.981 -0.123 (0)

PbCl4-2 2.628e-27 1.980e-27 -26.580 -26.703 -0.123 (0)

Pb4(OH)4+4 2.518e-31 8.108e-32 -30.599 -31.091 -0.492 (0)

S(6) 2.746e-04

SO4-2 2.477e-04 1.907e-04 -3.606 -3.720 -0.114 (0)

CaSO4 2.079e-05 2.079e-05 -4.682 -4.682 0.000 (0)

MgSO4 5.841e-06 5.841e-06 -5.234 -5.234 0.000 (0)

NaSO4- 2.110e-07 1.980e-07 -6.676 -6.703 -0.028 (0)

KSO4- 5.074e-08 4.760e-08 -7.295 -7.322 -0.028 (0)

ZnSO4 9.227e-10 9.227e-10 -9.035 -9.035 0.000 (0)

CrOHSO4 3.142e-10 3.142e-10 -9.503 -9.503 0.000 (0)

HSO4- 2.537e-10 2.378e-10 -9.596 -9.624 -0.028 (0)

CuSO4 1.460e-11 1.460e-11 -10.836 -10.836 0.000 (0)

PbSO4 5.121e-12 5.121e-12 -11.291 -11.291 0.000 (0)

CdSO4 4.967e-12 4.967e-12 -11.304 -11.304 0.000 (0)

CrSO4+ 2.531e-12 2.358e-12 -11.597 -11.628 -0.031 (0)

Zn(SO4)2-2 2.323e-12 1.750e-12 -11.634 -11.757 -0.123 (0)

Cd(SO4)2-2 2.043e-14 1.539e-14 -13.690 -13.813 -0.123 (0)

Pb(SO4)2-2 7.811e-15 5.884e-15 -14.107 -14.230 -0.123 (0)

Cr2(OH)2SO4+2 2.618e-16 1.972e-16 -15.582 -15.705 -0.123 (0)

Cr2(OH)2(SO4)2 2.234e-18 2.234e-18 -17.651 -17.651 0.000 (0)

CrO3SO4-2 2.785e-27 2.098e-27 -26.555 -26.678 -0.123 (0)

Zn 4.055e-08

Zn+2 3.280e-08 2.525e-08 -7.484 -7.598 -0.114 (0)

ZnCO3 5.012e-09 5.012e-09 -8.300 -8.300 0.000 (0)

ZnHCO3+ 1.288e-09 1.200e-09 -8.890 -8.921 -0.031 (0)

ZnSO4 9.227e-10 9.227e-10 -9.035 -9.035 0.000 (0)

ZnOH+ 4.056e-10 3.778e-10 -9.392 -9.423 -0.031 (0)

Zn(OH)2 9.732e-11 9.732e-11 -10.012 -10.012 0.000 (0)

ZnNO3+ 1.817e-11 1.693e-11 -10.741 -10.771 -0.031 (0)

Zn(SO4)2-2 2.323e-12 1.750e-12 -11.634 -11.757 -0.123 (0)

ZnCl+ 2.184e-12 2.046e-12 -11.661 -11.689 -0.028 (0)

ZnOHCl 1.482e-12 1.482e-12 -11.829 -11.829 0.000 (0)

Zn(OH)3- 2.582e-13 2.405e-13 -12.588 -12.619 -0.031 (0)

Zn(NO3)2 7.405e-16 7.405e-16 -15.130 -15.130 0.000 (0)

ZnCl2 5.974e-17 5.974e-17 -16.224 -16.224 0.000 (0)

Zn(OH)4-2 6.269e-18 4.722e-18 -17.203 -17.326 -0.123 (0)

ZnCl3- 1.720e-21 1.611e-21 -20.764 -20.793 -0.028 (0)

ZnCl4-2 3.328e-26 2.573e-26 -25.478 -25.590 -0.112 (0)

------------------------------Saturation indices-------------------------------

Phase SI** log IAP log K(283 K, 1 atm)

Anglesite -6.08 -13.98 -7.90 PbSO4

Anhydrite -2.68 -6.98 -4.29 CaSO4

Antlerite -9.93 -1.15 8.79 Cu3(OH)4SO4

Aragonite -0.53 -8.72 -8.19 CaCO3

Arsenolite -66.57 -69.88 -3.32 As4O6

Artinite -8.24 2.48 10.72 MgCO3:Mg(OH)2:3H2O

As2O5 -35.23 -28.31 6.92 As2O5

Atacamite -8.42 -0.16 8.26 Cu2(OH)3Cl

Azurite -7.71 -23.73 -16.02 Cu3(OH)2(CO3)2

Bianchite -9.56 -11.32 -1.76 ZnSO4:6H2O

Brochantite -12.27 4.84 17.10 Cu4(OH)6SO4

Brucite -6.24 11.66 17.90 Mg(OH)2

Ca3(AsO4)2:4H2O -14.24 8.06 22.30 Ca3(AsO4)2:4H2O

CaCrO4 -17.81 -19.83 -2.02 CaCrO4

Calcite -0.31 -8.72 -8.41 CaCO3

Cd(OH)2 -9.02 5.51 14.52 Cd(OH)2

Cd(OH)2(am) -9.03 5.51 14.54 Cd(OH)2

Cd3(OH)2(SO4)2 -28.39 -21.68 6.71 Cd3(OH)2(SO4)2

Cd3(OH)4SO4 -25.14 -2.58 22.56 Cd3(OH)4SO4

Cd4(OH)6SO4 -25.47 2.93 28.40 Cd4(OH)6SO4

CdCl2 -18.27 -18.76 -0.49 CdCl2

CdCl2:1H2O -17.13 -18.76 -1.62 CdCl2:1H2O

CdCl2:2.5H2O -16.78 -18.76 -1.98 CdCl2:2.5H2O

Cdmetal(alpha) -32.09 -17.87 14.21 Cd

Cdmetal(gamma) -32.20 -17.87 14.32 Cd

CdOHCl -10.45 -6.62 3.82 CdOHCl

CdSO4 -13.90 -13.59 0.31 CdSO4

CdSO4:1H2O -12.16 -13.59 -1.43 CdSO4:1H2O

CdSO4:2.67H2O -11.89 -13.59 -1.71 CdSO4:2.67H2O

Cerussite -2.36 -15.72 -13.36 PbCO3

CH4(g) -70.93 -114.36 -43.43 CH4

Chalcanthite -10.42 -13.11 -2.70 CuSO4:5H2O

Claudetite -66.30 -69.88 -3.58 As4O6

CO2(g) -2.66 -20.84 -18.18 CO2

Cotunnite -14.12 -19.14 -5.02 PbCl2

Cr(OH)2 -18.64 -7.49 11.15 Cr(OH)2

Cr(OH)3 -0.30 1.31 1.61 Cr(OH)3

Cr(OH)3(am) 2.06 1.31 -0.75 Cr(OH)3

Cr2O3 4.51 2.62 -1.89 Cr2O3

CrCl2 -46.87 -31.75 15.12 CrCl2

CrCl3 -51.32 -35.08 16.24 CrCl3

Crmetal -62.95 -30.87 32.08 Cr

CrO3 -28.79 -31.95 -3.16 CrO3

Cu(OH)2 -3.21 5.98 9.20 Cu(OH)2

Cu2(OH)3NO3 -9.26 0.66 9.92 Cu2(OH)3NO3

Cu2SO4 -23.49 -25.26 -1.77 Cu2SO4

Cu3(AsO4)2:2H2O -16.46 -10.36 6.10 Cu3(AsO4)2:2H2O

CuCO3 -3.36 -14.86 -11.50 CuCO3

CuCrO4 -20.53 -25.97 -5.44 CuCrO4

Cumetal -5.35 -14.77 -9.42 Cu

CuOCuSO4 -18.71 -7.13 11.58 CuOCuSO4

Cuprite -5.90 -6.16 -0.26 Cu2O

CuSO4 -16.73 -13.11 3.62 CuSO4

Dolomite(disordered) -1.79 -17.90 -16.11 CaMg(CO3)2

Dolomite(ordered) -1.18 -17.90 -16.72 CaMg(CO3)2

Epsomite -5.21 -7.44 -2.23 MgSO4:7H2O

Goslarite -9.17 -11.32 -2.14 ZnSO4:7H2O

Gypsum -2.36 -6.98 -4.62 CaSO4:2H2O

Halite -9.71 -8.15 1.57 NaCl

Huntite -7.30 -36.27 -28.97 CaMg3(CO3)4

Hydrocerussite -7.56 -26.33 -18.77 Pb3(OH)2(CO3)2

Hydromagnesite -18.33 -25.07 -6.74 Mg5(CO3)4(OH)2:4H2O

K2Cr2O7 -39.36 -57.35 -17.99 K2Cr2O7

K2CrO4 -24.72 -25.40 -0.68 K2CrO4

Langite -14.19 4.84 19.03 Cu4(OH)6SO4:H2O

Larnakite -8.63 -8.86 -0.23 PbO:PbSO4

Laurionite -7.64 -7.01 0.62 PbOHCl

Lime -22.38 12.12 34.50 CaO

Litharge -8.18 5.12 13.30 PbO

Magnesite -1.54 -9.18 -7.65 MgCO3

Malachite -2.86 -8.87 -6.01 Cu2(OH)2CO3

Massicot -8.40 5.12 13.51 PbO

Melanothallite -25.12 -18.28 6.85 CuCl2

Mg(OH)2(active) -7.13 11.66 18.79 Mg(OH)2

MgCr2O4 -3.59 14.28 17.87 MgCr2O4

MgCrO4 -26.50 -20.29 6.21 MgCrO4

Minium -38.70 38.74 77.44 Pb3O4

Mirabilite -9.28 -11.13 -1.85 Na2SO4:10H2O

Monteponite -10.56 5.51 16.06 CdO

Na2Cr2O7 -45.83 -55.93 -10.10 Na2Cr2O7

Na2CrO4 -27.09 -23.98 3.11 Na2CrO4

Nantokite -8.08 -15.21 -7.13 CuCl

Natron -10.95 -12.87 -1.92 Na2CO3:10H2O

Nesquehonite -4.74 -9.18 -4.45 MgCO3:3H2O

O2(g) -41.63 46.76 88.39 O2

Otavite -3.34 -15.34 -11.99 CdCO3

Pb(OH)2 -3.57 5.12 8.69 Pb(OH)2

Pb10(OH)6O(CO3)6 -65.10 -73.86 -8.76 Pb10(OH)6O(CO3)6

Pb2(OH)3Cl -10.69 -1.89 8.79 Pb2(OH)3Cl

Pb2O(OH)2 -15.95 10.24 26.19 Pb2O(OH)2

Pb2O3 -27.42 33.62 61.04 Pb2O3

Pb2OCO3 -10.43 -10.60 -0.18 Pb2OCO3

Pb3(AsO4)2 -18.75 -12.95 5.80 Pb3(AsO4)2

Pb3O2CO3 -17.53 -5.49 12.05 Pb3O2CO3

Pb3O2SO4 -15.16 -3.74 11.42 Pb3O2SO4

Pb4(OH)6SO4 -19.72 1.38 21.10 Pb4(OH)6SO4

Pb4O3SO4 -21.77 1.38 23.14 Pb4O3SO4

PbCrO4 -13.82 -26.83 -13.01 PbCrO4

Pbmetal -22.50 -18.26 4.24 Pb

PbO:0.3H2O -7.86 5.12 12.98 PbO:0.33H2O

Periclase -11.33 11.66 22.99 MgO

Phosgenite -15.06 -34.87 -19.81 PbCl2:PbCO3

Plattnerite -23.85 28.50 52.35 PbO2

Portlandite -11.87 12.12 24.00 Ca(OH)2

Smithsonite -3.21 -13.06 -9.85 ZnCO3

Tenorite -2.26 5.98 8.25 CuO

Thenardite -11.53 -11.13 0.41 Na2SO4

Thermonatrite -13.61 -12.87 0.73 Na2CO3:H2O

Zincite -4.38 7.78 12.17 ZnO

Zincosite -16.01 -11.32 4.70 ZnSO4

Zn(NO3)2:6H2O -17.92 -14.83 3.09 Zn(NO3)2:6H2O

Zn(OH)2 -4.42 7.78 12.20 Zn(OH)2

Zn(OH)2(am) -5.44 7.78 13.22 Zn(OH)2

Zn(OH)2(beta) -4.74 7.78 12.53 Zn(OH)2

Zn(OH)2(epsilon) -4.51 7.78 12.29 Zn(OH)2

Zn(OH)2(gamma) -3.95 7.78 11.73 Zn(OH)2

Zn2(OH)2SO4 -11.04 -3.54 7.50 Zn2(OH)2SO4

Zn2(OH)3Cl -11.76 3.43 15.19 Zn2(OH)3Cl

Zn3(AsO4)2:2.5H2O -18.61 -4.96 13.65 Zn3(AsO4)2:2.5H2O

Zn3O(SO4)2 -36.16 -14.85 21.31 Zn3O(SO4)2

Zn4(OH)6SO4 -16.37 12.03 28.40 Zn4(OH)6SO4

Zn5(OH)8Cl2 -23.85 14.65 38.50 Zn5(OH)8Cl2

ZnCl2 -24.20 -16.48 7.72 ZnCl2

ZnCO3:1H2O -2.80 -13.06 -10.26 ZnCO3:1H2O

Znmetal -42.81 -15.60 27.21 Zn

ZnO(active) -4.23 7.78 12.01 ZnO

ZnSO4:1H2O -11.09 -11.32 -0.23 ZnSO4:1H2O

**For a gas, SI = log10(fugacity). Fugacity = pressure * phi / 1 atm.

For ideal gases, phi = 1.

Initial solution 24.

----------------------------Distribution of species----------------------------

Log Log Log mole V

Species Molality Activity Molality Activity Gamma cm?mol

OH- 2.622e-07 2.427e-07 -6.581 -6.615 -0.034 (0)

H+ 1.359e-08 1.259e-08 -7.867 -7.900 -0.033 0.00

H2O 5.551e+01 9.999e-01 1.744 -0.000 0.000 18.02

As(3) 5.030e-19

H3AsO3 4.909e-19 4.909e-19 -18.309 -18.309 0.000 (0)

H2AsO3- 1.211e-20 1.113e-20 -19.917 -19.953 -0.036 (0)

HAsO3-2 5.697e-25 4.070e-25 -24.244 -24.390 -0.146 (0)

H4AsO3+ 3.330e-27 3.062e-27 -26.478 -26.514 -0.036 (0)

AsO3-3 1.546e-30 7.256e-31 -29.811 -30.139 -0.328 (0)

As(5) 1.782e-08

HAsO4-2 1.627e-08 1.162e-08 -7.789 -7.935 -0.146 (0)

H2AsO4- 1.548e-09 1.423e-09 -8.810 -8.847 -0.036 (0)

AsO4-3 4.326e-12 2.030e-12 -11.364 -11.692 -0.328 (0)

H3AsO4 2.672e-15 2.675e-15 -14.573 -14.573 0.001 (0)

C(4) 3.407e-03

HCO3- 3.247e-03 3.014e-03 -2.489 -2.521 -0.032 (0)

H2CO3 1.038e-04 1.038e-04 -3.984 -3.984 0.000 (0)

CaHCO3+ 2.934e-05 2.726e-05 -4.533 -4.565 -0.032 (0)

CO3-2 1.115e-05 8.215e-06 -4.953 -5.085 -0.133 (0)

MgHCO3+ 7.119e-06 6.594e-06 -5.148 -5.181 -0.033 (0)

CaCO3 6.888e-06 6.888e-06 -5.162 -5.162 0.000 (0)

MgCO3 1.231e-06 1.231e-06 -5.910 -5.910 0.000 (0)

NaHCO3 8.526e-07 8.526e-07 -6.069 -6.069 0.000 (0)

NaCO3- 9.551e-08 8.866e-08 -7.020 -7.052 -0.032 (0)

CuCO3 1.975e-08 1.975e-08 -7.704 -7.704 0.000 (0)

ZnCO3 1.648e-08 1.648e-08 -7.783 -7.783 0.000 (0)

CdCO3 2.824e-09 2.824e-09 -8.549 -8.549 0.000 (0)

ZnHCO3+ 2.646e-09 2.432e-09 -8.577 -8.614 -0.036 (0)

PbCO3 2.018e-09 2.018e-09 -8.695 -8.695 0.000 (0)

Cu(CO3)2-2 6.112e-10 4.367e-10 -9.214 -9.360 -0.146 (0)

PbHCO3+ 1.457e-10 1.339e-10 -9.837 -9.873 -0.036 (0)

CdHCO3+ 8.239e-11 7.575e-11 -10.084 -10.121 -0.036 (0)

Pb(CO3)2-2 6.691e-11 4.781e-11 -10.175 -10.321 -0.146 (0)

CuHCO3+ 6.181e-11 5.683e-11 -10.209 -10.245 -0.036 (0)

Cd(CO3)2-2 2.407e-11 1.720e-11 -10.619 -10.765 -0.146 (0)

Ca 1.080e-03

Ca+2 1.011e-03 7.447e-04 -2.995 -3.128 -0.133 (0)

CaSO4 3.135e-05 3.135e-05 -4.504 -4.504 0.000 (0)

CaHCO3+ 2.934e-05 2.726e-05 -4.533 -4.565 -0.032 (0)

CaCO3 6.888e-06 6.888e-06 -5.162 -5.162 0.000 (0)

CaNO3+ 9.785e-07 8.996e-07 -6.009 -6.046 -0.036 (0)

CaOH+ 3.250e-09 3.020e-09 -8.488 -8.520 -0.032 (0)

Cd 2.428e-08

Cd+2 2.048e-08 1.508e-08 -7.689 -7.822 -0.133 (0)

CdCO3 2.824e-09 2.824e-09 -8.549 -8.549 0.000 (0)

CdSO4 6.279e-10 6.279e-10 -9.202 -9.202 0.000 (0)

CdCl+ 1.762e-10 1.620e-10 -9.754 -9.791 -0.036 (0)

CdHCO3+ 8.239e-11 7.575e-11 -10.084 -10.121 -0.036 (0)

CdOH+ 3.230e-11 2.970e-11 -10.491 -10.527 -0.036 (0)

CdNO3+ 2.766e-11 2.543e-11 -10.558 -10.595 -0.036 (0)

Cd(CO3)2-2 2.407e-11 1.720e-11 -10.619 -10.765 -0.146 (0)

CdOHCl 3.678e-12 3.678e-12 -11.434 -11.434 0.000 (0)

Cd(SO4)2-2 3.054e-12 2.182e-12 -11.515 -11.661 -0.146 (0)

Cd(OH)2 4.835e-13 4.835e-13 -12.316 -12.316 0.000 (0)

CdCl2 7.434e-14 7.434e-14 -13.129 -13.129 0.000 (0)

Cd(NO3)2 2.769e-15 2.769e-15 -14.558 -14.558 0.000 (0)

Cd(OH)3- 2.569e-17 2.362e-17 -16.590 -16.627 -0.036 (0)

Cd2OH+3 5.797e-18 2.721e-18 -17.237 -17.565 -0.328 (0)

CdCl3- 5.047e-18 4.640e-18 -17.297 -17.333 -0.036 (0)

Cd(OH)4-2 4.328e-24 3.092e-24 -23.364 -23.510 -0.146 (0)

Cl 1.240e-04

Cl- 1.240e-04 1.149e-04 -3.907 -3.940 -0.033 (0)

CdCl+ 1.762e-10 1.620e-10 -9.754 -9.791 -0.036 (0)

ZnOHCl 1.054e-11 1.054e-11 -10.977 -10.977 0.000 (0)

ZnCl+ 9.681e-12 8.966e-12 -11.014 -11.047 -0.033 (0)

CdOHCl 3.678e-12 3.678e-12 -11.434 -11.434 0.000 (0)

CuCl 2.496e-12 2.496e-12 -11.603 -11.603 0.000 (0)

PbCl+ 3.009e-13 2.766e-13 -12.522 -12.558 -0.036 (0)

CdCl2 7.434e-14 7.434e-14 -13.129 -13.129 0.000 (0)

CuCl+ 6.723e-14 6.226e-14 -13.172 -13.206 -0.033 (0)

CuCl2- 6.717e-14 6.221e-14 -13.173 -13.206 -0.033 (0)

ZnCl2 8.311e-16 8.311e-16 -15.080 -15.080 0.000 (0)

CrCl+2 5.787e-16 4.135e-16 -15.238 -15.383 -0.146 (0)

PbCl2 1.323e-16 1.323e-16 -15.878 -15.878 0.000 (0)

CdCl3- 5.047e-18 4.640e-18 -17.297 -17.333 -0.036 (0)

CuCl3-2 1.941e-18 1.438e-18 -17.712 -17.842 -0.130 (0)

CuCl2 1.152e-18 1.152e-18 -17.938 -17.938 0.000 (0)

CrOHCl2 7.888e-20 7.888e-20 -19.103 -19.103 0.000 (0)

ZnCl3- 7.681e-20 7.114e-20 -19.115 -19.148 -0.033 (0)

PbCl3- 7.808e-21 7.179e-21 -20.107 -20.144 -0.036 (0)

CrCl2+ 1.238e-21 1.138e-21 -20.907 -20.944 -0.036 (0)

ZnCl4-2 4.869e-24 3.606e-24 -23.313 -23.443 -0.130 (0)

CuCl3- 1.008e-24 9.338e-25 -23.996 -24.030 -0.033 (0)

PbCl4-2 4.192e-25 2.995e-25 -24.378 -24.524 -0.146 (0)

CrO3Cl- 2.438e-28 2.242e-28 -27.613 -27.649 -0.036 (0)

CuCl4-2 1.232e-30 9.121e-31 -29.910 -30.040 -0.130 (0)

Cr(2) 8.866e-24

Cr+2 8.866e-24 6.335e-24 -23.052 -23.198 -0.146 (0)

Cr(3) 7.693e-07

Cr(OH)2+ 5.625e-07 5.172e-07 -6.250 -6.286 -0.036 (0)

Cr(OH)3 1.554e-07 1.554e-07 -6.809 -6.809 0.000 (0)

Cr(OH)+2 3.931e-08 2.809e-08 -7.405 -7.551 -0.146 (0)

CrO2- 6.376e-09 5.862e-09 -8.195 -8.232 -0.036 (0)

Cr(OH)4- 5.381e-09 4.947e-09 -8.269 -8.306 -0.036 (0)

CrOHSO4 2.698e-10 2.698e-10 -9.569 -9.569 0.000 (0)

Cr+3 1.033e-11 4.847e-12 -10.986 -11.314 -0.328 (0)

CrSO4+ 1.358e-12 1.248e-12 -11.867 -11.904 -0.036 (0)

CrCl+2 5.787e-16 4.135e-16 -15.238 -15.383 -0.146 (0)

Cr2(OH)2SO4+2 1.814e-16 1.296e-16 -15.741 -15.887 -0.146 (0)

CrNO3+2 2.561e-17 1.830e-17 -16.592 -16.738 -0.146 (0)

Cr2(OH)2(SO4)2 1.647e-18 1.647e-18 -17.783 -17.783 0.000 (0)

CrOHCl2 7.888e-20 7.888e-20 -19.103 -19.103 0.000 (0)

CrCl2+ 1.238e-21 1.138e-21 -20.907 -20.944 -0.036 (0)

Cr(6) 8.483e-16

CrO4-2 8.212e-16 6.049e-16 -15.086 -15.218 -0.133 (0)

HCrO4- 2.568e-17 2.361e-17 -16.590 -16.627 -0.036 (0)

NaCrO4- 1.227e-18 1.128e-18 -17.911 -17.948 -0.036 (0)

KCrO4- 1.130e-19 1.039e-19 -18.947 -18.983 -0.036 (0)

H2CrO4 1.093e-25 1.093e-25 -24.961 -24.961 0.000 (0)

CrO3SO4-2 2.829e-26 2.021e-26 -25.548 -25.694 -0.146 (0)

CrO3Cl- 2.438e-28 2.242e-28 -27.613 -27.649 -0.036 (0)

Cr2O7-2 4.061e-32 2.901e-32 -31.391 -31.537 -0.146 (0)

Cu(1) 2.133e-11

Cu+ 1.877e-11 1.726e-11 -10.727 -10.763 -0.036 (0)

CuCl 2.496e-12 2.496e-12 -11.603 -11.603 0.000 (0)

CuCl2- 6.717e-14 6.221e-14 -13.173 -13.206 -0.033 (0)

CuCl3-2 1.941e-18 1.438e-18 -17.712 -17.842 -0.130 (0)

Cu(2) 2.168e-08

CuCO3 1.975e-08 1.975e-08 -7.704 -7.704 0.000 (0)

Cu(CO3)2-2 6.112e-10 4.367e-10 -9.214 -9.360 -0.146 (0)

Cu+2 5.543e-10 4.083e-10 -9.256 -9.389 -0.133 (0)

CuOH+ 5.187e-10 4.804e-10 -9.285 -9.318 -0.033 (0)

Cu(OH)2 1.648e-10 1.648e-10 -9.783 -9.783 0.000 (0)

CuHCO3+ 6.181e-11 5.683e-11 -10.209 -10.245 -0.036 (0)

CuSO4 1.661e-11 1.661e-11 -10.780 -10.780 0.000 (0)

CuNO3+ 5.218e-13 4.797e-13 -12.283 -12.319 -0.036 (0)

Cu(OH)3- 2.940e-13 2.703e-13 -12.532 -12.568 -0.036 (0)

CuCl+ 6.723e-14 6.226e-14 -13.172 -13.206 -0.033 (0)

Cu2(OH)2+2 7.291e-15 5.209e-15 -14.137 -14.283 -0.146 (0)

Cu(NO3)2 1.883e-17 1.883e-17 -16.725 -16.725 0.000 (0)

Cu(OH)4-2 2.381e-18 1.701e-18 -17.623 -17.769 -0.146 (0)

CuCl2 1.152e-18 1.152e-18 -17.938 -17.938 0.000 (0)

CuCl3- 1.008e-24 9.338e-25 -23.996 -24.030 -0.033 (0)

CuCl4-2 1.232e-30 9.121e-31 -29.910 -30.040 -0.130 (0)

H(0) 2.623e-27

H2 1.311e-27 1.313e-27 -26.882 -26.882 0.001 (0)

K 4.998e-05

K+ 4.992e-05 4.624e-05 -4.302 -4.335 -0.033 (0)

KSO4- 6.910e-08 6.415e-08 -7.160 -7.193 -0.032 (0)

KCrO4- 1.130e-19 1.039e-19 -18.947 -18.983 -0.036 (0)

Mg 3.326e-04

Mg+2 3.162e-04 2.329e-04 -3.500 -3.633 -0.133 (0)

MgSO4 8.008e-06 8.008e-06 -5.096 -5.096 0.000 (0)

MgHCO3+ 7.119e-06 6.594e-06 -5.148 -5.181 -0.033 (0)

MgCO3 1.231e-06 1.231e-06 -5.910 -5.910 0.000 (0)

MgOH+ 1.872e-08 1.741e-08 -7.728 -7.759 -0.032 (0)

N(5) 3.684e-04

NO3- 3.674e-04 3.404e-04 -3.435 -3.468 -0.033 (0)

CaNO3+ 9.785e-07 8.996e-07 -6.009 -6.046 -0.036 (0)

ZnNO3+ 3.577e-11 3.289e-11 -10.446 -10.483 -0.036 (0)

CdNO3+ 2.766e-11 2.543e-11 -10.558 -10.595 -0.036 (0)

CuNO3+ 5.218e-13 4.797e-13 -12.283 -12.319 -0.036 (0)

PbNO3+ 4.287e-13 3.942e-13 -12.368 -12.404 -0.036 (0)

Cd(NO3)2 2.769e-15 2.769e-15 -14.558 -14.558 0.000 (0)

Zn(NO3)2 2.025e-15 2.025e-15 -14.694 -14.694 0.000 (0)

Pb(NO3)2 2.738e-16 2.738e-16 -15.563 -15.563 0.000 (0)

CrNO3+2 2.561e-17 1.830e-17 -16.592 -16.738 -0.146 (0)

Cu(NO3)2 1.883e-17 1.883e-17 -16.725 -16.725 0.000 (0)

Na 4.064e-04

Na+ 4.050e-04 3.752e-04 -3.393 -3.426 -0.033 (0)

NaHCO3 8.526e-07 8.526e-07 -6.069 -6.069 0.000 (0)

NaSO4- 4.545e-07 4.219e-07 -6.343 -6.375 -0.032 (0)

NaCO3- 9.551e-08 8.866e-08 -7.020 -7.052 -0.032 (0)

NaCrO4- 1.227e-18 1.128e-18 -17.911 -17.948 -0.036 (0)

O(0) 0.000e+00

O2 0.000e+00 0.000e+00 -42.860 -42.859 0.001 (0)

Pb 2.533e-09

PbCO3 2.018e-09 2.018e-09 -8.695 -8.695 0.000 (0)

PbOH+ 1.786e-10 1.642e-10 -9.748 -9.785 -0.036 (0)

PbHCO3+ 1.457e-10 1.339e-10 -9.837 -9.873 -0.036 (0)

Pb+2 1.109e-10 8.172e-11 -9.955 -10.088 -0.133 (0)

Pb(CO3)2-2 6.691e-11 4.781e-11 -10.175 -10.321 -0.146 (0)

PbSO4 8.561e-12 8.561e-12 -11.067 -11.067 0.000 (0)

Pb(OH)2 4.152e-12 4.152e-12 -11.382 -11.382 0.000 (0)

PbNO3+ 4.287e-13 3.942e-13 -12.368 -12.404 -0.036 (0)

PbCl+ 3.009e-13 2.766e-13 -12.522 -12.558 -0.036 (0)

Pb(SO4)2-2 1.544e-14 1.103e-14 -13.811 -13.957 -0.146 (0)

Pb(OH)3- 3.611e-15 3.320e-15 -14.442 -14.479 -0.036 (0)

Pb(NO3)2 2.738e-16 2.738e-16 -15.563 -15.563 0.000 (0)

PbCl2 1.323e-16 1.323e-16 -15.878 -15.878 0.000 (0)

Pb(OH)4-2 9.102e-19 6.503e-19 -18.041 -18.187 -0.146 (0)

Pb2OH+3 4.529e-19 2.126e-19 -18.344 -18.672 -0.328 (0)

PbCl3- 7.808e-21 7.179e-21 -20.107 -20.144 -0.036 (0)

Pb3(OH)4+2 3.351e-24 2.395e-24 -23.475 -23.621 -0.146 (0)

PbCl4-2 4.192e-25 2.995e-25 -24.378 -24.524 -0.146 (0)

Pb4(OH)4+4 1.062e-29 2.768e-30 -28.974 -29.558 -0.584 (0)

S(6) 3.303e-04

SO4-2 2.904e-04 2.139e-04 -3.537 -3.670 -0.133 (0)

CaSO4 3.135e-05 3.135e-05 -4.504 -4.504 0.000 (0)

MgSO4 8.008e-06 8.008e-06 -5.096 -5.096 0.000 (0)

NaSO4- 4.545e-07 4.219e-07 -6.343 -6.375 -0.032 (0)

KSO4- 6.910e-08 6.415e-08 -7.160 -7.193 -0.032 (0)

ZnSO4 1.429e-09 1.429e-09 -8.845 -8.845 0.000 (0)

CdSO4 6.279e-10 6.279e-10 -9.202 -9.202 0.000 (0)

CrOHSO4 2.698e-10 2.698e-10 -9.569 -9.569 0.000 (0)

HSO4- 1.774e-10 1.644e-10 -9.751 -9.784 -0.033 (0)

CuSO4 1.661e-11 1.661e-11 -10.780 -10.780 0.000 (0)

PbSO4 8.561e-12 8.561e-12 -11.067 -11.067 0.000 (0)

Zn(SO4)2-2 4.254e-12 3.040e-12 -11.371 -11.517 -0.146 (0)

Cd(SO4)2-2 3.054e-12 2.182e-12 -11.515 -11.661 -0.146 (0)

CrSO4+ 1.358e-12 1.248e-12 -11.867 -11.904 -0.036 (0)

Pb(SO4)2-2 1.544e-14 1.103e-14 -13.811 -13.957 -0.146 (0)

Cr2(OH)2SO4+2 1.814e-16 1.296e-16 -15.741 -15.887 -0.146 (0)

Cr2(OH)2(SO4)2 1.647e-18 1.647e-18 -17.783 -17.783 0.000 (0)

CrO3SO4-2 2.829e-26 2.021e-26 -25.548 -25.694 -0.146 (0)

Zn 6.923e-08

Zn+2 4.734e-08 3.487e-08 -7.325 -7.458 -0.133 (0)

ZnCO3 1.648e-08 1.648e-08 -7.783 -7.783 0.000 (0)

ZnHCO3+ 2.646e-09 2.432e-09 -8.577 -8.614 -0.036 (0)

ZnSO4 1.429e-09 1.429e-09 -8.845 -8.845 0.000 (0)

ZnOH+ 9.203e-10 8.461e-10 -9.036 -9.073 -0.036 (0)

Zn(OH)2 3.535e-10 3.535e-10 -9.452 -9.452 0.000 (0)

ZnNO3+ 3.577e-11 3.289e-11 -10.446 -10.483 -0.036 (0)

ZnOHCl 1.054e-11 1.054e-11 -10.977 -10.977 0.000 (0)

ZnCl+ 9.681e-12 8.966e-12 -11.014 -11.047 -0.033 (0)

Zn(SO4)2-2 4.254e-12 3.040e-12 -11.371 -11.517 -0.146 (0)

Zn(OH)3- 1.541e-12 1.417e-12 -11.812 -11.849 -0.036 (0)

Zn(NO3)2 2.025e-15 2.025e-15 -14.694 -14.694 0.000 (0)

ZnCl2 8.311e-16 8.311e-16 -15.080 -15.080 0.000 (0)

Zn(OH)4-2 6.313e-17 4.511e-17 -16.200 -16.346 -0.146 (0)

ZnCl3- 7.681e-20 7.114e-20 -19.115 -19.148 -0.033 (0)

ZnCl4-2 4.869e-24 3.606e-24 -23.313 -23.443 -0.130 (0)

------------------------------Saturation indices-------------------------------

Phase SI** log IAP log K(283 K, 1 atm)

Anglesite -5.86 -13.76 -7.90 PbSO4

Anhydrite -2.50 -6.80 -4.29 CaSO4

Antlerite -9.03 -0.24 8.79 Cu3(OH)4SO4

Aragonite -0.02 -8.21 -8.19 CaCO3

Arsenolite -69.92 -73.24 -3.32 As4O6

Artinite -7.27 3.45 10.72 MgCO3:Mg(OH)2:3H2O

As2O5 -36.06 -29.15 6.92 As2O5

Atacamite -7.28 0.98 8.26 Cu2(OH)3Cl

Azurite -6.52 -22.54 -16.02 Cu3(OH)2(CO3)2

Bianchite -9.37 -11.13 -1.76 ZnSO4:6H2O

Brochantite -10.93 6.17 17.10 Cu4(OH)6SO4

Brucite -5.73 12.17 17.90 Mg(OH)2

Ca3(AsO4)2:4H2O -13.43 8.87 22.30 Ca3(AsO4)2:4H2O

CaCrO4 -16.33 -18.35 -2.02 CaCrO4

Calcite 0.19 -8.21 -8.41 CaCO3

Cd(OH)2 -6.54 7.98 14.52 Cd(OH)2

Cd(OH)2(am) -6.56 7.98 14.54 Cd(OH)2

Cd3(OH)2(SO4)2 -21.71 -15.00 6.71 Cd3(OH)2(SO4)2

Cd3(OH)4SO4 -18.09 4.47 22.56 Cd3(OH)4SO4

Cd4(OH)6SO4 -15.96 12.44 28.40 Cd4(OH)6SO4

CdCl2 -15.21 -15.70 -0.49 CdCl2

CdCl2:1H2O -14.08 -15.70 -1.62 CdCl2:1H2O

CdCl2:2.5H2O -13.72 -15.70 -1.98 CdCl2:2.5H2O

Cdmetal(alpha) -30.04 -15.82 14.21 Cd

Cdmetal(gamma) -30.14 -15.82 14.32 Cd

CdOHCl -7.69 -3.86 3.82 CdOHCl

CdSO4 -11.80 -11.49 0.31 CdSO4

CdSO4:1H2O -10.06 -11.49 -1.43 CdSO4:1H2O

CdSO4:2.67H2O -9.79 -11.49 -1.71 CdSO4:2.67H2O

Cerussite -1.81 -15.17 -13.36 PbCO3

CH4(g) -72.65 -116.09 -43.43 CH4

Chalcanthite -10.36 -13.06 -2.70 CuSO4:5H2O

Claudetite -69.65 -73.24 -3.58 As4O6

CO2(g) -2.70 -20.89 -18.18 CO2

Cotunnite -12.94 -17.97 -5.02 PbCl2

Cr(OH)2 -18.55 -7.40 11.15 Cr(OH)2

Cr(OH)3 0.00 1.61 1.61 Cr(OH)3

Cr(OH)3(am) 2.36 1.61 -0.75 Cr(OH)3

Cr2O3 5.11 3.23 -1.89 Cr2O3

CrCl2 -46.20 -31.08 15.12 CrCl2

CrCl3 -50.14 -33.91 16.24 CrCl3

Crmetal -63.28 -31.20 32.08 Cr

CrO3 -27.86 -31.02 -3.16 CrO3

Cu(OH)2 -2.79 6.41 9.20 Cu(OH)2

Cu2(OH)3NO3 -8.47 1.45 9.92 Cu2(OH)3NO3

Cu2SO4 -23.42 -25.20 -1.77 Cu2SO4

Cu3(AsO4)2:2H2O -16.01 -9.91 6.10 Cu3(AsO4)2:2H2O

CuCO3 -2.97 -14.47 -11.50 CuCO3

CuCrO4 -19.17 -24.61 -5.44 CuCrO4

Cumetal -5.34 -14.76 -9.42 Cu

CuOCuSO4 -18.23 -6.65 11.58 CuOCuSO4

Cuprite -5.47 -5.73 -0.26 Cu2O

CuSO4 -16.68 -13.06 3.62 CuSO4

Dolomite(disordered) -0.82 -16.93 -16.11 CaMg(CO3)2

Dolomite(ordered) -0.21 -16.93 -16.72 CaMg(CO3)2

Epsomite -5.07 -7.30 -2.23 MgSO4:7H2O

Goslarite -8.98 -11.13 -2.14 ZnSO4:7H2O

Gypsum -2.18 -6.80 -4.62 CaSO4:2H2O

Halite -8.93 -7.37 1.57 NaCl

Huntite -5.40 -34.37 -28.97 CaMg3(CO3)4

Hydrocerussite -5.86 -24.63 -18.77 Pb3(OH)2(CO3)2

Hydromagnesite -15.97 -22.71 -6.74 Mg5(CO3)4(OH)2:4H2O

K2Cr2O7 -36.91 -54.91 -17.99 K2Cr2O7

K2CrO4 -23.21 -23.89 -0.68 K2CrO4

Langite -12.85 6.17 19.03 Cu4(OH)6SO4:H2O

Larnakite -7.81 -8.05 -0.23 PbO:PbSO4

Laurionite -6.75 -6.13 0.62 PbOHCl

Lime -21.83 12.67 34.50 CaO

Litharge -7.59 5.71 13.30 PbO

Magnesite -1.07 -8.72 -7.65 MgCO3

Malachite -2.05 -8.06 -6.01 Cu2(OH)2CO3

Massicot -7.80 5.71 13.51 PbO

Melanothallite -24.11 -17.27 6.85 CuCl2

Mg(OH)2(active) -6.63 12.17 18.79 Mg(OH)2

MgCr2O4 -2.47 15.39 17.87 MgCr2O4

MgCrO4 -25.06 -18.85 6.21 MgCrO4

Minium -36.50 40.94 77.44 Pb3O4

Mirabilite -8.67 -10.52 -1.85 Na2SO4:10H2O

Monteponite -8.08 7.98 16.06 CdO
[truncated: 599,012 more chars]
